# iTRAQ-based protein profiling provides insights into the central metabolism changes driving grape berry development and ripening

**DOI:** 10.1186/1471-2229-13-167

**Published:** 2013-10-24

**Authors:** María José Martínez-Esteso, María Teresa Vilella-Antón, María Ángeles Pedreño, María Luz Valero, Roque Bru-Martínez

**Affiliations:** 1 Grupo de Proteómica y Genómica Funcional de Plantas, Dept. Agroquímica y Bioquímica, Facultad de Ciencias, Universidad de Alicante, Apartado 99, E-03080 Alicante, Spain; 2 Grupo de Peroxidasas Vegetales, Department Fisiología Vegetal, Facultad de Biología, Universidad de Murcia, Campus de Espinardo, E-30100 Murcia, Spain; 3 Laboratorio de Proteómica, Centro de Investigación Príncipe Felipe, Av. Autopista del Saler, 16, 46012 Valencia, Spain

**Keywords:** Development, Grape berry, iTRAQ, Mesocarp, Proteomics, Quantitative, *Vitis vinifera*, Functional annotation

## Abstract

**Background:**

Grapevine (*Vitis vinifera* L.) is an economically important fruit crop. Quality-determining grape components such as sugars, acids, flavors, anthocyanins, tannins, etc., accumulate in the different grape berry development stages. Thus, correlating the proteomic profiles with the biochemical and physiological changes occurring in grape is of paramount importance to advance in our understanding of berry development and ripening processes.

**Results:**

We report the developmental analysis of *Vitis vinifera* cv. Muscat Hamburg berries at the protein level from fruit set to full ripening. An iTRAQ-based bottom-up proteomic approach followed by tandem mass spectrometry led to the identification and quantitation of 411 and 630 proteins in the green and ripening phases, respectively. Two key points in development relating to changes in protein level were detected: end of the first growth period (7 mm-to-15 mm) and onset of ripening (15 mm-to-V100, V100-to-110). A functional analysis was performed using the Blast2GO software based on the enrichment of GO terms during berry growth.

**Conclusions:**

The study of the proteome contributes to decipher the biological processes and metabolic pathways involved in the development and quality traits of fruit and its derived products. These findings lie mainly in metabolism and storage of sugars and malate, energy-related pathways such as respiration, photosynthesis and fermentation, and the synthesis of polyphenolics as major secondary metabolites in grape berry. In addition, some key steps in carbohydrate and malate metabolism have been identified in this study, i.e., PFP-PFK or SuSy-INV switches among others, which may influence the final sugar and acid balance in ripe fruit. In conclusion, some proteins not reported to date have been detected to be deregulated in specific tissues and developmental stages, leading to formulate new hypotheses on the metabolic processes underlying grape berry development. These results open up new lines to decipher the processes controlling grape berry development and ripening.

## Background

Grapevine (*Vitis vinifera* L.) is the most important fruit crop in the world, and viticulture and enology play an important role in the economy of many developed and emerging countries. In 2007, the Food and Agriculture Organization (FAO) of the United Nations estimated that the gross world production of grape berries was over 67 million tons per year, with land occupancy of 7,272,583 ha (FAO, 2007 [1]). In the food industry, grape berries are widely commercialized raw, as a fresh and dried fruit, or are processed mainly as grape juice or wine. Extracts from grape berry seeds, skin and also grape plant leaves are used as commodities in nutraceutical and cosmetic industries.

The grape berry is a non climacteric fruit that exhibits a double sigmoid growth pattern [2]. The first growth phase after fruit set is characterized by not only rapid cell division, which increases the number of cells, but also by an expansion of existing cells. It is followed by a lag phase with little or no growth. The second growth phase coincides with the onset of ripening, called *véraison*, which is characterized by important biochemical and physiological changes such as softening, coloring and engustment of berry. As grape berries develop, they change in size and composition. These transformations range from small, firm and acidic with little sugar, desirable flavors or aroma, to becoming larger, softened, sweet, highly flavored, less acidic and highly colored fruit. The biochemical changes underlying grape berry ripening have been recently reviewed [3]. The development of these traits is subject to genetic, environmental and viticultural conditions, which determine the final quality of the berry and its derived products. During the first growth period, chlorophyll is the main pigment present in fruit and cells are rich in organic acids; the most prevalent compounds are tartaric and malic acids, which accumulate mainly in skin and flesh. Compounds vastly increase starting at *véraison*, and the major ones are glucose and fructose [4], in addition to phenolic and aromatic compounds [5], while malate concentration lowers [6]. Flavor development in grapes is partly due to the acid/sugar balance [7], which is particularly important in table grapes. Muscat Hamburg is a classical cultivar of black table grape that is grown in many parts of Europe, and is greatly appreciated for its pleasant Muscat flavour [8,9].

Correct grape ripening is fundamental for both commercial fruit use and wine quality. For this reason, grape berry development has been investigated by monitoring the expression of quality trait-related genes in a classic targeted approach [4,10-13], and also by global mRNA expression profiling using cDNA or oligonucleotide microarrays [14-19] since genomic resources for *Vitis vinifera* and related species have been generated on a large scale.

In recent years, proteomics-based technologies have been successfully applied to grapevine to analyze stress responses [20-23] and berry development [24-28]. These studies have improved the understanding of changes in grape berries at the proteome level, but are limited by the intrinsic inaccuracy of single staining-based quantitative two-dimensional gel electrophoresis (2-DE) [29]. The application of more robust quantitative proteomic approaches, such as difference gel electrophoresis (DIGE) or MS-based stable isotopic labeling, should lead to a better coverage and accuracy of protein changes. DIGE has been applied to study grape berry development. It has led to a better coverage and accuracy of the protein changes reported from a gel-based approach [30,31]. Lücker *et al.*[32] studied grape berry ripening initiation by the quantitative MS-based iTRAQ approach, and reported a significantly larger number of total protein identification and protein changes than single-stain 2-DE-based studies. These authors noticed the difficulty of protein identification as no finished genome sequence data for grapevine were available. Thus, they built a protein database by combining in-house generated [33] and publicly available grapevine nucleotide sequences. To date, this limitation has been greatly reduced thanks to grapevine genome sequencing initiatives [34,35], which have released the translated ORFs found in the genome to public protein databases. Nevertheless, the extent of sequence description and annotation of grapevine proteins, including genome-derived ones, is still far from complete. The exocarp of grape berries has been analyzed throughout ripening by the iTRAQ technology to offer better proteome coverage [36] and by making full use of recent grapevine genome sequencing initiatives [34,35].

Stable isotopic labeling followed by a reverse phase high-performance liquid chromatography coupled with tandem mass spectrometry (HPLC-MS/MS) analysis is a relatively recent quantitative technique, and was first described by Ross *et al*. [37]. Isobaric tags for relative and absolute protein quantitation (iTRAQ) are currently being used successfully to characterize and quantify changes in protein levels in complex biological samples [38]. The use of iTRAQ has become a consolidated technique in quantitative proteomics since large fold changes of protein expression within broad dynamic ranges of protein abundance can be measured quite accurately [39]. The iTRAQ method allows the multiplexed identification and quantitation of proteins in four different samples (4-plex) and has been recently scaled up to measure protein changes in up to eight different samples (8-plex) [40].

The tissue-specific analysis of grape berry is critical if information on its individual contribution to final fruit quality is sought. Quality traits relating to the organoleptic properties of berry and wine are linked to specific berry tissue: skin composition plays an important role in determining color, aroma, astringency and bitterness, while the pulp contributes to the sugar/acid balance, crispiness, juiciness and alcoholic potential. The seed also contributes to the astringency and bitterness of wines.

Grimplet *et al*. [41], used a large-scale transcriptomic analysis to study the differential expression of genes among main tissues, seed, flesh and skin, and reported that 60% of these genes exhibited a significant differential expression in at least one of the three major tissue types. Several proteomic analyses at the tissue-specific level have been carried out [24,26,31,32,36,42]. Nevertheless on the whole, a gap still remains either because one only tissue is analyzed or berry development is not comprehensively covered.

In this study, the issue of protein expression changes is addressed in the pericarp/mesocarp tissues in grape berry and all the developmental phases are covered. For the purpose of gaining a better understanding of the protein composition in grape berry and its changes during development, seven different stages from fruit set to fully ripened were analyzed. The aim is to improve coverage and to complement the proteomic analysis done in previous DIGE experiments [31] with the high-throughput proteomic analysis presented herein using an alternative approach, namely the top-down quantitative proteomics iTRAQ technique. The use of annotation and analysis tools [43], along with recent literature findings, has also led to gain insights into the biological significance of the proteomic data in the search for useful protein biomarkers to track the final quality traits associated with grape berry development.

## Results and discussion

**Figure 1:**
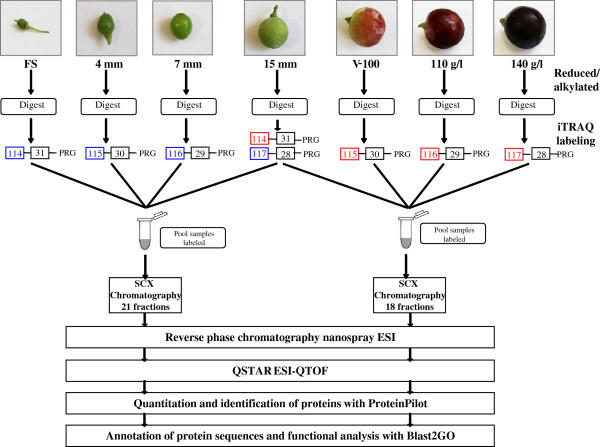
**Workflow of the iTRAQ experiments for both the first growth period and grape berry ripening.** The first growth period, analyzed in the first iTRAQ experiment, covers four developmental stages: fruit set (FS), 4 mm, 7 mm and 15 mm labeled with the 114, 115, 116 and 117 iTRAQ tags, respectively. The ripening period covers three developmental stages, 100% *véraison* (V-100), 110 g/l and 140 g/l, and a fourth point, the 15 mm stage, which is common with the previous experiment, and was co-analyzed in a second iTRAQ experiment. For each experiment, the extracted proteins were trypsin-digested and peptides were labeled with iTRAQ tags. After labeling, peptides were pooled and fractionated by strong cation exchange (SCX) chromatography. Each fraction was resolved by reverse phase chromatography and was analyzed directly by mass spectrometry. Searches and quantitation analysis were carried out using the ProteinPilot v1.0 software (Applied Biosystems). Finally, the identified proteins in the set were annotated and functionally analyzed using the Blast2GO v2.4.0 application [43] based on gene ontology (GO) terms.

Grape berry development was analyzed by the iTRAQ technique. One iTRAQ experiment covered four time points of the first growth period, which spanned the stages from fruit set to pre-*véraison* (FS-to-15 mm). Another iTRAQ experiment covered three time points of the second growth period from onset to full ripening (V100-to-140 g/l), and a pre-*véraison* fourth time point (15 mm), shared with the first experiment. Figure 1 describes the experimental design and workflow for both experiments.

### Protein identification and quantitation

In the green stages, 542 proteins were identified, which is similar to the number of proteins identified with at least two peptides in an iTRAQ experiment of the grape berry exocarp [36] (Additional file 1). Of these, 524 proteins were quantified and, after applying a p<0.05 filter, the list shortened to 411 (Additional file 2). All the 524 quantified sequences were assigned a description and 508 were annotated with an average number of five annotations per sequence and an average annotation level depth of 5.8.

In the ripe stages, 1117 proteins were identified, which is almost twice the number of those identified in the experiment for green stages (Additional file 3). Of these, 1034 proteins were quantified and, after applying a p<0.05 filter, the list shortened to 630 (Additional file 4). Of the 1034 sequences, 1010 were assigned a description and 977 were annotated with an average number of five annotations per sequence and an average annotation level depth of 5.5.

As a summary of the annotation results, pie charts for the biological function and cellular component gene ontology (GO) terms are shown for each experiment in Additional file 5, respectively. The resulting annotation files are provided as Additional file 6 (green) and Additional file 7 (ripe).

### Proteome changes during development

**Figure 2:**
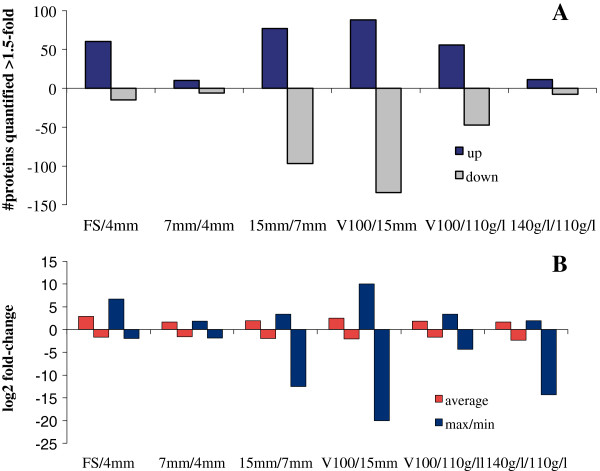
**Overall changes in the protein level throughout berry development.** Each value represents **(A)** the number of sequences quantified between two consecutive developmental stages, for all the proteins that were up- (dark blue bars) and down-regulated (gray bars), and **(B)** the fold-change of the proteins, represented as mean (red) and max/min (blue) changes. Transitions were: fruit set to 15 mm (FS-to-15 mm), 4 mm to 15 mm (4-to-15 mm), and 7 mm to 15 mm (7-to-15 mm), respectively. The experiment linked to the ripe stages was stated as follows: V100 to 15 mm (V100-to-15 mm), 110 g/l to 15 mm (110-to-15 mm) and 140 g/l to 15 mm (140-to-15 mm). An arbitrary fold change cutoff of ± 1.5 was used to select the protein subsets.

To better characterize the most active stages as regards proteome changes, the relative abundance of the deregulated proteins was computed between contiguous stages (reporter ion ratios 115/114, 116/115 and 117/116 per experiment) and these values were used to rank the selected protein lists. For each stage transition, two subsets of proteins were established: those whose fold change was above 1.5 and those with a fold change under −1.5. Figure 2 provides a global picture of the proteome changes between each pair of contiguous sampled time points of the grape berry formation phase. Figure 2A illustrates the number of up- and down-regulated proteins passing the filter, whereas Figure 2B depicts the average and the highest fold change of that set of proteins. Of the number of deregulated proteins, three classes of development stages can be formed: stages with very few changes (transitions from 4 mm-to-7 mm and from 110 g/l-to-140 g/l); stages with many changes (transitions from 7 mm-to-15 mm and from 15 mm-to-V100); stages with an intermediate number of changes (transitions FS-to-4 mm and V100-to-110 g/l). If the amplitude of changes was considered, the average fold change among the paired stages was not so different. However, there were few very major changes at certain transitions that revealed potential marker proteins for the developmental stages. The results obtained herein agree with those reported in a previous proteomic experiment, which analyzed the same time points using the DIGE platform [31]. The additional time point included in the present study, the transition from V100-to-15 mm, resulted in the development stage where the largest number of proteins underwent changes and where the largest fold-changes occurred. These results suggest that the most important changes at the protein level took place at the end of green period and at the onset of ripening, as previous data from genomic [44], oligo/microarray transcriptomic [14,15] and proteomic [27,31,32] studies have also indicated.

### Functional analysis

The present study contributes to significantly increase the coverage of the differential proteome of the whole development of grape berries and confidently allows the detection of putative marker proteins based on a previous proteomic experiment, which revealed that the protein changes observed were developmentally associated with the analyzed biological individuals [31]. The good agreement between most of the profiles of the proteins identified in common herein and in Martínez-Esteso *et al.*[31], serves as validation the present study using the non gel accurate quantitative proteomic iTRAQ approach.

#### Functional annotation analysis

**Figure 3:**
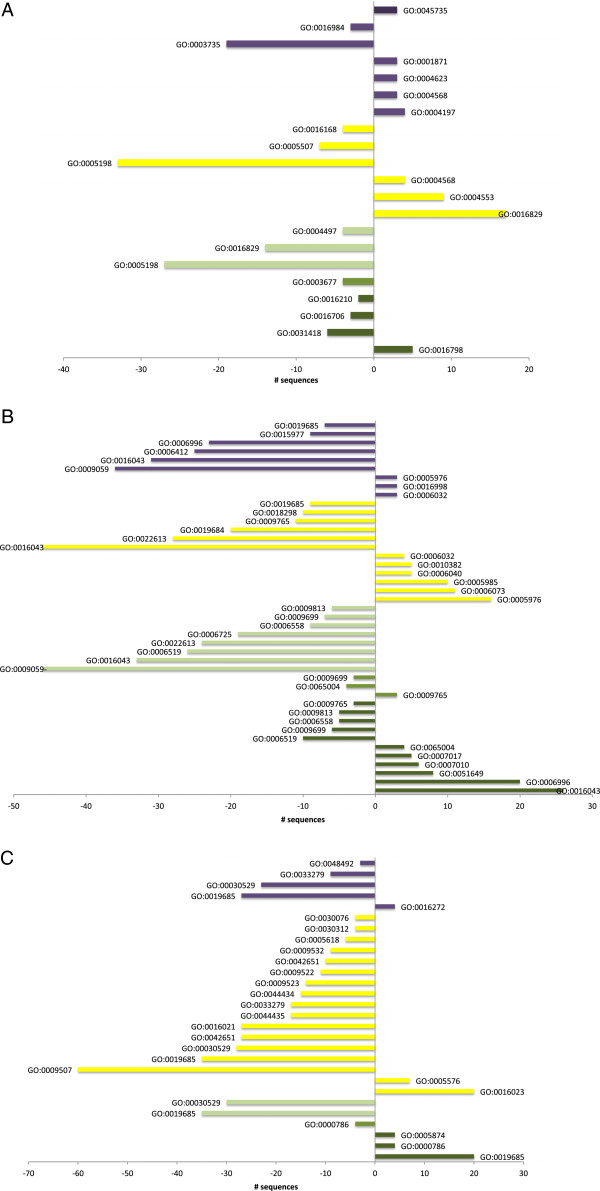
**Enriched GO terms (Molecular function (F term) (A), Biological process (P term) (B) and Cellular component (C term) (C)) for the sequences annotated in the up- or down-regulated subsets of proteins in each grape berry developmental stage analyzed.** Bar diagrams indicate the number of sequences that were up- and down-regulated (X-axis), annotated with each enriched GO term (Y-axis) in the consecutive paired stages. GO terms descriptions are detailed in Additional file 8. Color code for the consecutive pair stages: FS-to-4 mm transitions (dark green bars), 4 mm-to-7 mm transitions (medium intensity green), 15 mm-to-7 mm (light green), V100-to-15 mm transitions (yellow), 110 g/l-to-15 mm transitions (light purple), 140 g/l-to-110 g/l (dark purple).

A Fischer’s enrichment analysis [45] was carried out by comparing the frequencies of the annotation terms in the up- and down-regulated protein subsets with those of the whole list of quantified proteins as reference list. As a result, a series of GO terms, which were statistically overrepresented in the subset, was obtained (p<0.005). Figure 3 shows the result of the Fischer’s enrichment analysis for the molecular function (F term) (Figure 3A), biological processes (P term) (Figure 3B) and the cellular components (C term) (Figure 3C) -selected GO terms whose level in most cases was 4 or deeper. Additional file 8 provides a list of the proteins annotated with each enriched GO term.

The proteins annotated with the GO-terms enriched from the up-regulated list of proteins in FS-to-4 mm were mainly different isoforms of the histones, tubulins and proteins involved in cytoskeleton organization and biogenesis. Several ribosomal proteins, which were annotated with the terms ‘intracellular nonmembrane-bound organelle-GO:0019685’ (C term) and ‘cellular component organization and biogenesis- GO:0016043’ (P term), appeared to be up-regulated in FS-to-4 mm. In contrast, these terms were down-regulated from 7 mm onwards, showing those as leading processes during the early stages of development, when fruit cells were very active in division and expansion, and new proteins were being synthesized. The term ‘hydrolase activity, acting on glycosyl bonds-GO:0016798’ (F term), which appeared to be significantly up-regulated, related to different glycosyl hydrolases and an acid vacuolar invertase, which started to accumulate from the 4 mm stage and was possibly involved in the carbohydrate catabolism of sucrose unloaded in fruit by acting as a sink organ in the vine. The proteins involved in ‘flavonoid biosynthetic processes-GO:0009813’ and ‘aromatic amino acid synthesis-GO:0006725’ (P term) appeared to be down-regulated at this point of development, including the ‘phenylalanine metabolic process-GO:0006558’ GO term (P term), a precursor of all the phenylpropanoid-derived compounds. This finding reflects an accumulation of flavonoids in early fruit development. The 4 mm-to-7 mm transition was characterized by a few terms enriched in the down- and up-subsets of proteins, which reflect that most of the processes involved in development occurred earlier than or after this time point. Only the term ‘photosynthesis light harvesting-GO:0009765’ was up-regulated and some terms annotating several isoforms of histones were down-regulated (‘nucleosome-GO:0000786’ (C term) ‘protein-DNA complex assembly-GO:0065004’ (P term) and ‘DNA binding-GO:0003677’ (F term)), which displayed greater accumulation earlier in grape berry development. In the next transition (7 mm-to-15 mm), only the down-regulated subset of proteins contained GO-enriched terms. The most remarkable processes, these being the ‘macromolecule biosynthetic process-GO:0009059’ (P term) and ‘cellular component organization and biogenesis- GO:0016043’ (C term), were linked with proteins like elongation factors, tubulins, histones and ribosomal proteins, which agrees with the state of cells at this point of development. These results tie in with the fact that fruit entered a lag phase, with little or no division or expansion, and most processes were switched off before triggering the *véraison*. Another important down-regulated term was the phenylpropanoid metabolism (GO:0009699), involving flavonoids and amino acid precursors, which included the enzymes of the general phenylpropanoid pathway. The processes down-regulated at 7 mm-to-15 mm occurred mainly in the cytosol, unlike those down-regulated from 15 mm-to-V100, and which also occurred mainly in the chloroplast. Several proteins belonging to the photosynthetic machinery (‘Photosystem I-GO:0009522’ (C term), ‘Photosystem II-GO:0009523’(C term)) and those involved in photosynthesis, both light (GO:0019684) and dark reactions (GO:0019685) (phosphoribulokinase, rubisco), were strongly down-regulated before *véraison* was triggered. Some related processes occurring in the chloroplast such as starch metabolism (4-alpha-glucanotransferase), redox state (thioredoxin, peroxiredoxin) and protein folding (chaperonin 60), are linked with the terms enriched in the down-regulated subset, thus also indicating a decrease of the photosynthetic-dependent processes. In parallel, a significant number of ribosomal proteins was down-regulated (the term ‘structural molecule activity-GO:0005198’ (F term)). The term ‘integral to membrane-GO:0016021’ (C term) included an aquaporin and plasma membrane H^+^-ATPase, indicating a decrease in water-associated specific transporters prior to *véraison*. Likewise, cell wall-associated processes (endo-xyloglucan tranferase, pectinesterase) appeared to be deregulated, indicating that these profiles play a relevant role at *véraison* as this stage was characterized by fruit softening. This is a complex regulated process as inferred by the terms deregulated at this transition, 15 mm-to-V100, in which some specific isoforms related to cell wall biogenesis (the terms ‘cytoplasmic membrane-bound vesicle-GO:0016023’(C term), ‘extracellular region-GO:0005576’ (C term), endo-xyloglucan transglycosylase, expansin, polygalacturonase, pectin methylesterase, invertase pectin methylesterase inhibitor family) were strongly up-regulated, while other isoforms were down-regulated at this point. Interestingly, several stress-related proteins annotated with the term ‘copper ion binding-GO:0005507’ (F term) were down-regulated (polyphenol oxidase, diphenol oxidase, superoxide dismutase). The terms which up-regulated at *véraison* were ‘cytoplasmic membrane-bound vesicle-GO:0016023’, ‘extracellular region-GO:0005576’ and, apart from the proteins indicated above, they involved ripening-related proteins (grip22), plasma membrane proteins and some proteases (subtilisin, cysteine proteinase inhibitor). Nevertheless, the most relevant terms during this period were ‘cell wall metabolic process-GO:0010382’ (P term), ‘chitin catabolic process-GO:0006032’ (P term), involving defense proteins (PR-4, chitinase IV), carbohydrate-related processes, which included the proteins involved in sucrose metabolism (sucrose synthase, sucrose-phosphate synthase) and, behind the term ‘lyase activity-GO:0016829’ (F term), hexose and pyruvate metabolism (enolase, aldolase, pyruvate decarboxylase) was found. Altogether, this reflects the intense activity around carbohydrate metabolism at the onset of ripening, presumably in the biosynthetic direction, to convert existing pools of malate into sugars for their storage since glycolysis is supposed to be inhibited after the onset of ripening [6].

In the V100-to-110 transition, the down-regulated process predominated the up-regulated ones. Among the up-regulated ones, the terms ‘chitin catabolic process’ and ‘polysaccharide metabolic process-GO:0005976’ (P term), including defense proteins, were kept, and new terms appear such as ‘prefolding complex-GO:0016272’ (C term) (needed for the stabilization of the newly synthetized proteins) and ‘cysteine-type endopeptidase activity-GO:0004197’ (F term) (cysteine proteinase, the cysteine protease component of the protease-inhibitor complex and cathepsin b-like cysteine protease). Among the down-regulated proteins, the most remarkable feature was the diminished abundance of proteins synthesis, as interpreted by the enriched terms ‘intracellular non-membrane-bound organelle’ , (C term) ‘cellular component organization and biogenesis’ (P term), which included an important representation of different ribosomal proteins, and the term ‘translation-GO:0006412’ (P term) (t-RNA synthetases). Apart from these, other terms of the cellular component and molecular function categories associated with the ribosomal function were enriched in the down-regulated subset (‘macromolecule biosynthetic process’ (P term), ‘structural constituent of ribosome’-GO:0003735 (C term)). Another remarkable feature was that the proteins involved in the photosynthetic dark reaction were strongly down-regulated, which reflects that grape berries lose their photosynthetic capability as they ripen. Finally in the 110-to-140 transition, only the term ‘nutrient reservoir activity-GO:0045735’ (F term) was enriched in the up-regulated subset of proteins. This term included three seed storage proteins that are considerably accumulated.

#### Protein profile analysis

As shown in Additional file 9, the behaviour of the time course of the functional protein groups, which lie beneath important quality traits of the pericarp/mesocarp during fruit development, was observed in detail. The functional protein groups of ‘transporters’, ‘polyphenols’, ‘photosynthesis, respiration and fermentation’ and ‘carbohydrate and malate metabolism’ are discussed herein. The remaining functional protein groups, including ‘nitrogen and amino acid (Additional file 10) ‘terpenoid metabolism’, ‘signaling and hormone’, ‘stress’, ‘protein synthesis’, ‘protein degradation’, ‘protein processing’, ‘cell division and growth, biogenesis’ ‘defense proteins’ and ‘other proteins of interest’, are discussed in Additional file 11.

#### 1. Transporters

**Figure 4:**
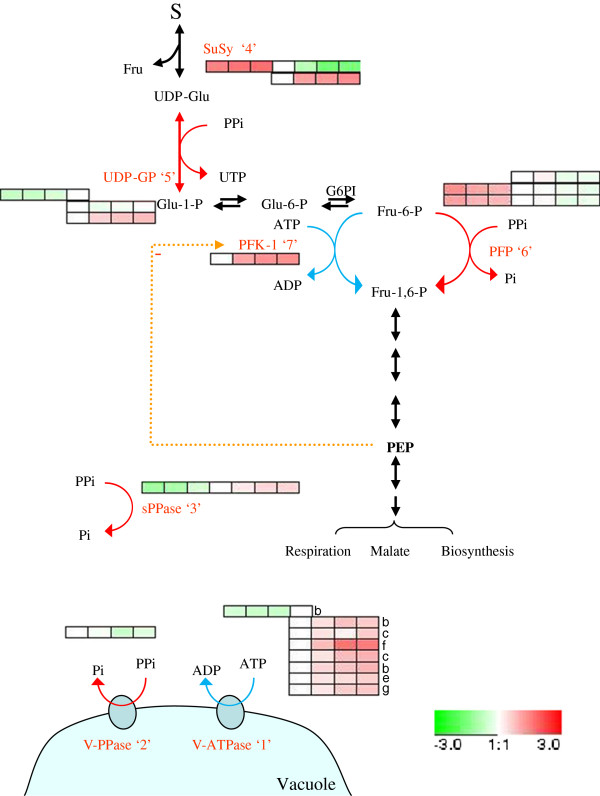
**Scheme of the pyrophosphate (PPi) metabolism.** The PPi-dependent (red line) and ATP-dependent (blue line) reactions are highlighted. The protein levels of the regulated enzymes are shown in coloured squares, indicating the change of expression (log_2_ ratio) for each developmental stage in relation to the 15 mm stage. In sequence order (left to right), stages are displayed from FS, 4 mm, 7 mm, 15 mm, V-100, 110 g/l, and 140 g/l. Different proteins isoforms or subunits are shown as different rows. SuSy, sucrose synthase; UDP-GP, UDP-glucose pyrophosphorylase; PFK-1, phosphofructokinase 1; PFP, pyrophosphate-dependent phosphofructokinase; sPPase, soluble pyrophosphatase; V-ATPase, vacuolar ATPase; PP-ase, vacuolar pyrophosphatase.

Here we will focus on transport phenomena involved in tonoplast and plasma membrane energization for its special relevance in sugar transport and accumulation. The vacuole is a very important organelle in grape berry cells because of its dominant cytoplasm occupancy [46], its predominant role as a storage compartment of organic acids, sugars, secondary metabolites and amino acids, and because of its central cell function in cytosolic pH regulation. To date, knowledge of the transporter proteins in the tonoplast is surprisingly scarce. Thus, the high acidity of grapes and the changes that occur during ripening still mean that important questions are raised. V-ATPase and V-PPase are two tonoplast primary proton pumps that are widely distributed in plants cells. Here, the protein levels for both pumps were quantified in the mesocarp during ripening. Several V-ATPase subunits were found up-regulated during development starting at the onset of ripening (Figure 4, ‘1’). This agrees with the profiles of subunits C and F reported in previous studies [31]. The protein level of V-PPase proved constant from the onset of ripening to 100% *véraison,* but then decreased until the end of ripening (Figure 4, ‘2’), which agrees with reported transcript and protein profiles [47]. On the basis of V-PPase and V-ATPase proton pumping activities, a predominant role of V-PPase in the energization of the grape tonoplast during development has been proposed despite its poorer stoichometry efficiency as compared to V-ATPase [47] while the role of V-ATPase became relevant throughout the ripening phase [48]. Thus, the protein profiles found herein for both the H^+^ pumps were in accordance with their reported activities [48]. Although V-PPase was not detected during the first growth period, the profiles found showed more abundance at *véraison* than at ripening; such decrease combined with the emergency of a putative cytosolic sPPase (Figure 4, ‘3’) along ripening, points to pyrophosphate (PPi) as an energy source to pump protons across the tonoplast during green development, but not during ripening.

Furthermore, the induction of ripening is known to be associated with the shift of phloem unloading from the symplastic to the apoplastic pathway [28]. The latter involves the participation of several plasma-membrane sugar transporters which are induced at *véraison*[3]. Sugar translocation is a secondary transport driven by a proton gradient established by plasma membrane H^+^-ATPase (PM-ATPase). Here the abundance of PM-ATPase (Additional file 9A) peaked at the onset of ripening and became down-regulated in the following stages, which is consistent with previous findings [49]. This profile is consistent with the sugar concentrations in the phloem and berry apoplast; that is, high at *véraison* and early ripening, but lower as the berry ripens [50], and it responds to the PM energization need. Thus, PM-ATPase can play a role relevant in triggering sugar accumulation in berries. Unlike PM, the energy demand at the tonoplast level continuously increased for use in both sugar translocation to the vacuole and the maintenance of the high sugar gradient concentration between the cytosol and the vacuole. Indeed the induction of proton-sugars antiporters supported vacuolar sugar accumulation [3]. The specific tonoplast antiporter carrier for hexoses, VvHT6, was up-regulated during ripening (Additional file 9A) in accordance with reported transcript levels [14,17]. In addition, vacuolar H^+^ pumps must play a key role in generating a proton motive force to accumulate sugars. As discussed above, both V-PPase and V-ATPase can play this role, but considering their respective profiles and the progressive accumulation of soluble PPase, the importance of the ATP-driven pump increases as the berry ripens.

As note above, PPi may play key functions during berry development although its metabolism is still a largely unknown process. PPi is released as a by-product during several anabolic reactions, which are highly active during young development, such as the synthesis of DNA, RNA, proteins, carbohydrates, etc., and it can be simply hydrolyzed by soluble pyrophosphatase (sPPase) to pull up biosynthetic reactions or used in other processes. According to the abundance profiles of the PPi-utilizing cytosolic enzymes (Figure 4), in the developing grape berry it might act as an energy donor *per se*[51] for tonoplast energization through the V-PPase pump, but also as a phosphoryl group donor in sucrose degradation via sucrose synthase (SuSy) (Figure 4, ‘4’), and UDP-glucose pyrophosphorylase (UDP-GP) (Figure 4, ‘5’) and in glycolysis through pyrophosphate-dependent phosphofructokinase (PFP) (Figure 4, ‘6’) (reviewed Plaxton, [52]). Besides the profiles for these enzymes, it is interesting to note that the profile for sPPase correlates positively to that of phosphofructokinase 1 (PFK-1) (Figure 4, ‘7’) and V-ATPase, but negatively to that of PPi-utilizing enzymes such as PFP and V-PPase, therefore, sPPase is the candidate to control the cytosolic PPi pools in the ripening berry development phase.

#### 2. Polyphenols

**Figure 5:**
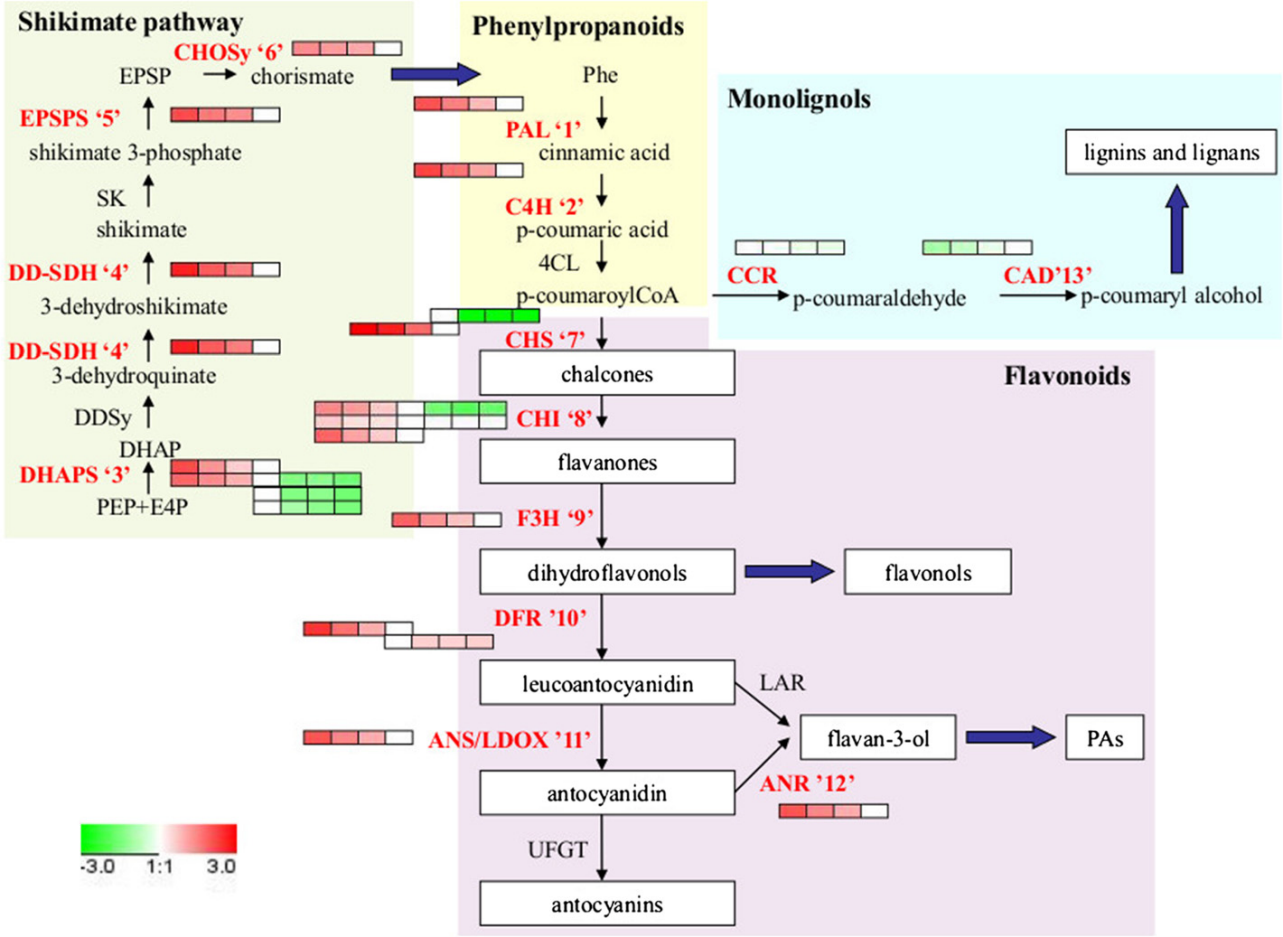
**Scheme of the polyphenol biosynthetic pathways in grape berries during development and ripening.** These include shikimate pathway as the phenylalanine (Phe) source, the general phenylpropanoid pathway and the branches of monolignols and flavonoids, including flavonols, proanthocyanidins (PAs) and anthocyanins (ACs) The protein levels of the regulated enzymes are shown in coloured squares, indicating the change of expression (log_2_ ratio) for each developmental stage in relation to the 15 mm stage. In sequence order (left to right), stages are displayed from FS, 4 mm, 7 mm, 15 mm, V-100, 110 g/l, and 140 g/l. Different isoforms or subunits of proteins are shown as different rows. The enzyme names for each catalytic step are indicated in red if differentially expressed and identified, or in black if not detected. PAL, phenylalanine ammonia lyase; C4H, cinnamate-4-hydroxylase; 4CL, 4-coumarate CoA-ligase; CHS, chalcone synthase; CHI, chalcone isomerase; F3H, flavonoid 3-hydroxylase; DFR, dihydroflavonol 4-reductase; ANS/LDOX, anthocyanidin reductase/leucoanthocyanidin dioxigenase; UFGT, UDP-glucose flavonoid 3-O-glucosyltransferase; ANR, anthocyanidin reductase; LAR, leucoanthocyanidin reductase; CCR, cinnamoyl-CoA reductase; CAD, cinnamyl alcohol dehydrogenase; DHAPS, 3-deoxy-d-arabino-heptulosonate 7-phosphate synthase; DD-SDH, dehydroquinate dehydratase shikimate:nadp oxidoreductase; EPSPS, 5-enol-pyruvylshikimate-phosphate synthase; CHOSy, chorismate synthase.

From a wine quality perspective, the major phenolic compounds in grape berries are non flavonoid hydroxycinnamic acids (HCyAs), and flavonoid anthocyanins (ACs) and proanthocyanidins (PACs) [53]. The synthesis of phenolics share the phenylpropanoid pathway, from phenylalanine (Phe) to p-coumaric acid, from which several branches derive, leading in grapes to lignins, hydroxycinnamic acids, stilbenoids and flavonoids [3] (see Figure 5). The obtained results clearly show enhanced phenylpropanoid synthesis from very early development as the two first enzymes of the pathway, phenylalanine ammonia lyase (PAL, Figure 5 ‘1’) and cinnamate 4-hydroxylase (C4H, Figure 5 ‘2’), were at least 4-fold up-regulated toward fruit set in relation to 15 mm. Likewise, five enzymes of the shikimate pathway (3-deoxy-D-arabino heptulosonate 7-phosphate synthase (DHAPS, Figure 5 ‘3’), dehydroquinate dehydratase- shikimate dehydrogenase (DD-SDH, Figure 5 ‘4’), 5-enol-pyruvilshikimate-phosphate synthase (EPSPS, Figure 5 ‘5’) and chorismate synthase (CHOSy, Figure 5 ‘6’)), leading to aromatic amino acids, had the same profile as the phenylpropanoid enzymes during green development (see Figure 5). Flavonoids constituted a significant portion of phenolics and included ACs, flavonols, flavan-3-ols and PACs or condensed tannins (polymers of flavan-3-ols and flavan-3,4-diols), and were the most abundant class of soluble polyphenolics in grape berries. Common enzymes for the flavonoid pathway (chalcone synthase (CHS, Figure 5 ‘7’), chalcone isomerase (CHI, Figure 5 ‘8’), flavonol-3-hydroxylase (F3H, Figure 5 ‘9’), dihydroflavonol reductase (DFR, Figure 5 ‘10’), anthocyanidin synthase/leucoanthocyanidin dioxigenase (ANS/LDOX, Figure 5 ‘11’) and the branching point toward PACs biosynthesis (anthocyanidin reductase (ANR, Figure 5 ‘12’)) displayed the same abundance pattern as the shikimate and phenylpropanoid enzymes. Although no enzyme of stilbene or hydroxycinnamic acid synthesis branches was detected, a resveratrol/hydroxycinnamic acid O-glucosyltransferase (RHCA-GT) with an abundance profile like that above was identified. It shared a 94% homology with a bi-functional glucosyltransferase from *Vitis lambrusca* cv. Concord (VLRSgt), which produced glycosides of stilbenes and glucose esters of HCyAs *in vitro*[54]. Conversely, cinnamyl alcohol dehydrogenase (CAD, Figure 5 ‘13’), which is involved in lignin biosynthesis, displayed quite an opposite profile. No other enzymes acting at the branching points of the flavonoid pathway were detected. Together all these results provide strong evidence that in pre-*véraison* stages, carbon flows mainly from glycolysis precursors toward not only the production of PAC precursors but, and based on weaker evidence, also hydroxycinnamic acid and stilbene derivatives. Alternatively, the production of other pathway end products, such as lignins, flavones, flavonols and ACs, seemed blocked in the mesocarp by the absence of the corresponding enzymes. These results are highly consistent with the type of Phe-derived secondary metabolites accumulation in the developing grape berry [3,55-60].

Besides ANR, leucoanthocyanidin reductase (LAR) also produces PAC precursors; both were expressed during PACs accumulation in grapes and were regulated in a temporal- and tissue-specific manner [59]. Although LAR was not detected in the pericarp of green berries, ANR (Figure 5 ‘12’) could play a relevant role in driving flavonoid biosynthesis in the epicatechin production in the tissue during early berry development (see Figure 5). The general trend observed for flavonoid-derived compound synthesis was that it was down-regulated in the ripening phase. This stems from the observation that CHI and CHS were strongly down-regulated at *véraison,* and remained down-regulated until full ripening. Of the remaining flavonoid pathway enzymes, only DFR was detected and displayed almost unchanged levels from 15 mm to full ripening. In grape berry skins, where ACs heavily accumulated at the onset of ripening, no new synthesis of precursors from p-coumaric acid apparently occurred in the skin, as observed by the decreasing profiles of the downsteam enzymes [36]. The presence of DFR in the mesocarp during ripening would warrant a supply of ACs precursors for synthesis in the skin via ANS and UDP-glucose: flavonoid 3-O-glucosyltransferase (UFGT), as suggested by Martínez-Esteso *et al*. [36].

Although flavonoid compounds were synthesized in the cytosol, they accumulated in vacuoles. It has been thought that the mechanism underlying their transport is mediated through gluthatione-S-transferase (GST) and ABC transporters [61,62], although GST-independent transport also occurs (reviewed by Terrier *et al.*, [63]). High-throughput studies during development in skin-colored cultivars have revealed the expression of a GST associated with ACs accumulation [14,36,64]. Here, seven GSTs belonging to the tau, phi and lambda classes were found to be deregulated in flesh during berry development. As Additional file 12 depicts, only one tau class, associated with AC accumulation, was also detected in berry skin, and none coincided with the four tau class isoforms associated with stilbenoid accumulation in elicited cell cultures [65]. The tau and phi GSTs, in addition to flavonoid binding, also perform known auxin and cytokinin binding activities [66,67], and a role in plant growth and development has been suggested [62].

Three isoenzymes of an isoflavone reductase-like protein (IFRL) have been reported as being up-regulated during ripening in the mesocarp, which confirms previous results obtained in the grape berry mesocarp and skin [31,36]. The deregulated IFRLs showed a high sequence homology with Eugenol synthase2 from *Clarkia brevweri* (CbEGS2) (79%) and with phenylcoumaran benzylic ether reductase (PCBER) from *Populus trichocarpa* (PtPCBER) (82%). Enzymatic activities reported for CbEGS2 show a preference on coniferyl acetate over dehydrodiconiferyl alcohol (DDC) as a substrate [68] which suggests that the sequences currently characterized as PCBER enzymes, as well as other phylogenetically related sequences, might prefer coniferyl acetate or other related substrates than DDC. In fact, increased metabolite eugenol during ripening, one of the volatiles present in grape berries, has been reported in berries of cv. Muscat Hamburg [69]. This increase ran in parallel to our quantitative protein profiles for IFRLs. Further experiments are required to test these hypotheses.

#### 3. Photosynthesis, respiration and fermentation

Apart from the photosynthates which translocate from leaves, grape berries are capable of local photosynthetic assimilation. A large number of them was detected and quantified through these iTRAQ experiments, thus providing quite a complete picture of the grape berry photosynthetic machinery during berry development at the protein level for the first time (Additional file 13). Almost every enzyme involved in the Calvin-Benson cycle was identified and quantified. They displayed decreasing abundance profiles throughout the berry formation and ripening processes, with a sharper decrease from 7-to-15 mm or 15 mm-to-V100. Moreover, a broad representation of the light reactions proteins was also detected and quantified (Additional file 13). The general profile trend for these proteins differed for those involved in Calvin-Benson during berry formation, with light reaction proteins displaying a moderate increase in abundance during the first growth period which leveling off from 7 mm-to-15 mm. Abundance reduced during fruit ripening in the same way it did for the carbon fixation proteins. These results are consistent with studies reported at the physiological, transcriptional and enzyme activity levels, which indicated that the capability of grapes to perform photosynthesis diminished before *véraison* and during the ripening process [47,70,71]. Likewise, they can correlate largely with transcriptomic studies [14,15,17]. The phasing-out of the Calvin-Benson cycle in relation to light reaction proteins during the first growth period suggests that an important part of the energy and reducing power can be used for processes other than carbon assimilation, such as the biosynthesis of aromatic amino acids and flavonoids, synthesis of proteins or cell division, which are highly active during berry formation. Indeed, grape berries cannot achieve a net carbon assimilation rate through photosynthesis at any development stage, while photosynthesis would serve to balance the loss of CO_2_ by respiration [72].

As noted in the DIGE experiments, oxygen-evolving enhancer (OEE) proteins were up-regulated from 7 mm-to-15 mm [31]. Subunits of 33 KDa (OEE33) and 16 KDa (OEE3) increased more markedly than most PSI, PSII, LHCI and LHCII proteins. As hypothesized in a previous proteomic study [31], an increase in the OEE components could respond to a redirection of unused electrons toward O_2_ modulating H_2_O_2_ formation, which is a widely accepted theory when NADP^+^ is in short supply [73,74] and may occur at the end of the berry formation phase when many of the aforementioned biosynthetic processes slow down, or even stop. Interestingly, two fibrillin isoforms (see Additional file 11, ‘Other proteins of interest’) displayed that the protein profiles ran in parallel to those of photosynthetic machinery proteins (see Additional file 13) with a 1.5- and 2-fold increase for each one from 7 mm-to-15 mm, which decreased after *véraison*. Fibrillins are lipid-binding proteins involved in the ABA-mediated photoprotection of PSII [75]. The fibrillin profile in the grape berry supports the PSII stabilization theory for the redirection of electrons and H_2_O_2_ formation control when it diminishes carbon reduction dramatically through the Calvin cycle. In line with these findings, antioxidant chloroplastic enzymes were simultaneously enhanced (see Additional file 11, ‘Stress’).

**Figure 6:**
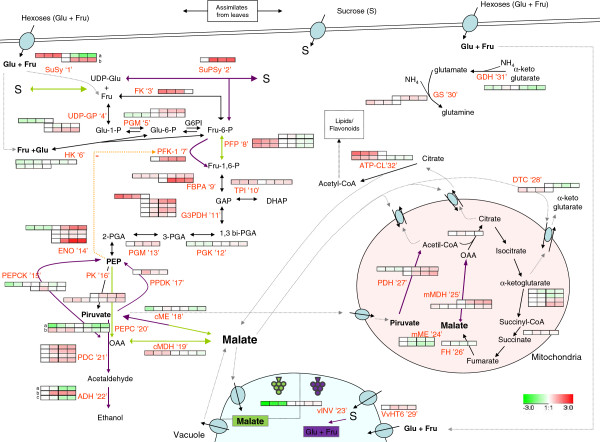
**Metabolic pathways involved in malate metabolism in grape fruits during development.** The protein levels of deregulated enzymes are shown in coloured squares, indicating the change of expression (log_2_ ratio) for each developmental stage in relation to the 15 mm stage. In sequence order (left to right), stages are displayed from FS, 4 mm, 7 mm, 15 mm, V-100, 110 g/l, and 140 g/l. Different isoforms or subunits of proteins are shown as different rows. The enzymes names for each biochemical step are indicated in red if differentially expressed and identified, or in black if not detected. Black solid and dashed arrows indicate enzymatic reactions and metabolite movement, respectively. SuSy, sucrose synthase; SuPSy, sucrose phosphate synthase; UDP-GP, UDP- glucose pyrophosphorylase; PFK-1: Phosphofructokinase-1; PFP, pyrophosphate-dependent phosphofructokinase; FK, fructokinase; HK, hexokinase; PGM, phosphoglucomutase; FBPA, fructose bisphosphate aldolase; TPI, triose phosphate isomerase; G3PDH, glyceraldehide-3-phosphate dehydrogenase; PGK, phosphoglycerate kinase; ENO, enolase; PGM, phosphoglycerate mutase; PK, pyruvate kinase; PEPC, phosphoenolpyruvate carboxylase; PEPCK, phosphoenolpyruvate carboxykinase; PPDK, pyruvate orthophosphate dikinase; cMDH, cytosolic malate dehydrogenase; cME, cytosolic malic enzyme; PDC, pyruvate decarboxylase; ADH, alcohol dehydrogenase; vINV, vacuolar acid invertase; VvHT6, hexose transporter 6; PDH, pyruvate dehydrogenase; mMDH, malate dehydrogenase mitochondrial; mME, mitochondrial malic enzyme; FH, fumarate hydratase; ATP-CL, ATP-citrate lyase; GS, glutamine synthase; GDH, glutamate dehydrogenase; DTC, dicarboxylate-tricarboxylate carrier.

As regards respiratory activity, several proteins involved in the mitochondrial respiratory chain and oxidative phosphorylation have been detected to be deregulated during development (Additional file 9C). The profiles for these proteins, along with several subunits for the ATP synthase and Complex I NADH:UQ reductase, increased during the first growth period, but decreased from the onset of ripening, which agrees with the respiratory activities described during development [71,72]. The adenylate and phosphate transporters also displayed similar protein profiles from the onset of ripening (see Additional file 9A). Other proteins of the respiratory chain from Complex III and cytochrome c remained unchanged or only increased slightly. Profiles were consistent with progressive electron transport deceleration during ripening. When mitochondrial respiration is compromised, the ATP synthase is able to reverse and consume ATP, which serves to maintain the mitochondrial membrane potential. This activity can deplete ATP and precipitate cell death. This process can be mitigated by mitochondrial protein IF_1_, an endogenous ATP synthase inhibitor [76]. An ATPase inhibitor protein was found to be strongly up-regulated from 7 mm-to-15 mm and during ripening. Here the results also reveal that the protein levels of NAD-ME (mME, Figure 6 ‘24’, see group 4 ‘Carbohydrate metabolism’) peaked at pre-*véraison,* but declined during ripening. mME was able to supply high levels of NADH to the mitochondrial matrix when malate was abundant. This reducing environment could activate non-phosphorylating dehydrogenases (reviewed by Sweetman *et al*. [77]) when the phosphorylating pathway is limited, which coincides with the oxidative burst observed at *véraison*[78]. Instead of the alternative oxidase as a bypass of proton translocating Complex-I, a mitochondrial uncoupling protein (MUP) was high at pre-*véraison* and down-regulated after the onset of ripening, which could contribute to the re-oxidization of coenzymes irrespectively of the energy status of berry cells.

These data provide further evidence for the decreasing respiration rates of the mitochondria as ripening proceeds. In such a situation, the TCA cycle is expected to become progressively inhibited by respiratory control, thus triggering the accumulation of malate in the cytosol. Consequently, part of the malate would be diverted toward ethanol fermentation, as supported by pyruvate decarboxylase accumulation (PDC, Figure 6 ‘21’, see group 4 ‘Carbohydrate metabolism’) and alcohol dehydrogenase 2 (ADH2) during ripening, (Figure 6 ‘22b’, see Group 4 ‘Carbohydrate metabolism’). ADH2 is the isoform responsible for ethanol fermentation at ripening [79]. In strawberry, ethanol fermentation can occur aerobically in ripening fruit if acidity increases due to, for instance, limited respiratory activity, with malate as a putative carbon source [80]. The above situation may lead to a shift in the cytosolic NAD(P)H:NAD(P)^+^ balance to a reduced form. Interestingly, NADP-dependent FMN quinone reductase (BQR) has been reported to markedly increase by up to 4-fold from V-100-to-110 g/l and to maintain its levels during ripening. BQR enzymes have not been characterized in grape berries and only poorly so in plants [81]. BQRs allow for the two-electron reduction of quinones to the hydroquinone form to avoid the generation of one-electron reduced semiquinone, which is known to cause oxidative stress [82], while an isoform in yeast has been shown to act as a NAD(P)-redox sensor for oxidative stress [83]. Hence, BQR becomes an interesting protein to study its putative implication in complex redox regulation in the ripening grape berry. In addition, BQR has been shown to be one of the most abundant proteins in the grape berry mesocarp [31].

#### 4. Carbohydrate and malate metabolism

Efforts have been made in recent decades to explain the metabolic enzymes controlling the sugar/acid balance during grape development, a major quality trait of grape berry flesh. Sucrose produced through photosynthesis in the mesophyll of mature leaves is loaded into the phloem and unloaded into sink berries throughout their development and ripening stages (reviewed by Boss and Davies, [7]). From fruit set to *véraison,* malate is stored in vacuoles as a major end product of imported sucrose; thereafter malate is consumed, while large amounts of sucrose-derived glucose and fructose are stored in the vacuole. As demonstrated by Zhang *et al*. [50], the unloading pathway shifts from symplastic to apoplastic at the onset of ripening, and is accompanied by a concominant increase in the expression and activity of cell wall invertases (cwINV). Proteomic data [31] have provided evidence for a switch between sucrose synthase (SuSy) and vacuolar acid invertase (vINV) before *véraison*, thus supporting a supposed functional mechanism between SuSy and INV for unloading sugars during grape berry development and ripening, respectively [84], as demonstrated in tomato [85-87]. In the current experiments, cwINV was not identified, but a vINV (Figure 6, ‘23’) was strongly up-regulated (14-fold) from 7-to-15 mm and protein levels were maintained during ripening. The transcripts and protein levels of two vINV (VvGIN1 and VvGIN2) accumulated before *véraison* and decreased during fruit ripening [4,17], although vINV activity peaked before *véraison* and remained constant on a per berry basis [4]. Moreover, the natural depletion of vINV in the Steuven grapevine hybrid contributed to increase sucrose in the maturing berry [88]. The above cumulative evidence indicates that, apart from the important role of cwINV during ripening [50], vINV also plays a relevant role in driving the import of sugars during ripening. Furthermore, SuSy (Figure 6, ‘1’) has been identified as two isoforms. One was abundant since the beginning of development to 7 mm, but then decreased abruptly before *véraison*, displaying the opposite trend to that of vINV (Figure 6, ‘1a’). The second SuSy isoform (Figure 6 ‘1b’) appeared to be up-regulated at the onset of ripening, while the levels were maintained thereafter until full ripening. Since the SuSy expression was induced by a sugar-sensing mechanism [89], its decrease before *véraison* suggests a lower sucrose concentration in the cytoplasm of sink cells, probably due to plasmodesmata blocking taking place at the shift of the unloading pathway [50]. The first isoform, which was also detected in a previous study [31], is a clear candidate to be involved in both sugar unloading and metabolism during the first growth period, while the second isoform would be responsible for the function during ripening, together with vINV. The present iTRAQ experiments revealed a third enzyme involved in sucrose unloading and sink strength, sucrose phosphate synthase (SuPSy) (Figure 6 ‘2’), which was up-regulated almost 4-fold at the onset of *véraison* and remained at constant levels during ripening. The joint action of SuSy, SuPSy and INV would result in dynamic sucrose synthesis and degradation cycles, referred to as ‘futile cycles’, which occurred in the plant cells regulating flow intensity and direction. Sucrose futile cycles are key mechanisms for unloading and storing sugars into ripening tomato fruit [87] that, as in grapes, stops the symplastic unloading pathway at the onset of ripening [50].

Malate is the main organic acid in grape berries and shows an accumulation pattern, which peaks in the pre-*véraison* stage before ripening is triggered [90]. Its metabolism operates tightly in parallel with sugar fate and carbohydrate metabolism in grape berry cells in the mesocarp. The abundance profiles for the enzymes involved in carbohydrate metabolism tie in with those found in a previous DIGE proteomic study [31]. Moreover, this iTRAQ large-scale study has allowed to increase the coverage of the metabolic pathways driving the sugar/acid balance during development, and more importantly, also of those enzymes controlling the glycolysis/gluconeogenesis and synthesis/degradation of malate. A previous study has highlighted the relevance of pyrophosphate-dependent phosphofructokinase (PFP, Figure 6 ‘8’) as the catalyst of the phosphorylation of fructose-6 phosphate during green development. As discussed before (Figure 4), the low levels of a sPPase and the occurrence of PPi releasing reactions during green development support the availability of PPi for PFP. PFP, unlike phosphofructokinase-1 (PFK-1, Figure 6 ‘7’), is not negatively regulated by end products such as PEP or ATP, thus allowing for the glycolytic flow to proceed driven simply by substrate availability. Here the profile of PFP reveals that, after dropping just before *véraison*, the levels are no longer recovered. PFK-1, detected in the ripening phase only, was strongly up-regulated at *véraison* and its levels were held until the end of ripening. Hence, during ripening glycolysis would be controlled strictly by accumulated PEP and by the energy status of mesocarp cells.

The next key event was the usage of PEP during green development. Several lines of evidence support two major uses: conversion into malate via oxalacetate (OAA) for storage in vacuoles [17,69,91] and respiration [72]. The profile of the corresponding enzymes acting on PEP indicated that both pathways are open (Figure 6).

The two enzymes converting PEP into OAA, PEP carboxylase (PEPC, Figure 6 ‘20’) irreversibly and PEP carboxykinase (PEPCK, Figure 6 ‘15’) reversibly, were detected here, but with quite different profiles. One isoform of PEPC was abundant during early development, but dropped before *véraison,* while another isoform displayed an abundance peak at *véraison*. Such profiles are consistent with those for gene expression [14,77,92] and enzyme activity [93,94] in developing grape berries, thus providing evidence for its role in malate synthesis at the protein level. Instead, the abundance of PEPCK isoforms started to increase in the last green development stages, and their level was held until the end of ripening. Despite the detection of transcripts and enzyme activity in pre-*véraison* berries [14,77,95], the profiles found herein do not support a key role of PEPCK in malate synthesis, as previously suggested [96], rather they would be important during ripening, as discussed below. The subsequent conversion of OAA into malate in the green stages by cytosolic malate dehydrogenase (cMDH) is also well supported by the cMDH profile (Figure 6 ‘19’), by the active glycolysis and PEPC in this phase as continuous sources of NADH and OAA, respectively, and by the thermodynamics of the reaction itself. Although no individual measurements of cMDH activity have been reported, the profiles found herein, as compared to those of mitochondrial MDH (mMDH), suggest that it may contribute largely to the high levels of total MDH activity reported during early development [97]. Taken together, the present results support the notion that a major carbon flow in malate synthesis in the green phase of grape berry development occurs via PEPC and cMDH until its maximum accumulation at pre-*véraison*.

In respiratory usage, PEP is directly converted into pyruvate through pyruvate kinase (PK, Figure 6 ‘16’) and is transported to mitochondria for complete oxidation. The respiratory use of PEP is supported by the PK profile, which is more abundant during early development than at pre-*véraison,* when it is minimal. The profiles of pyruvate dehydrogenase (PDH, Figure 6 ‘27’) and MDHm in that phase are flat. The CO_2_ evolution profiles in grape berries, higher early on in development to decrease at the beginning of ripening [72], correlate better with glycolytic enzymes, including PK, than with mitochondrial enzymes PDH and mMDH, which suggests that the respiratory machinery is not the rate-limiting step. Since both glycolysis and photosynthesis are operative, the carbon flow through the former and the intensity of the latter (see the profiles above) may be major determinant factors of CO_2_ evolution.

Pyruvate might potentially be converted into malate by the cytosolic NADP-dependent malic enzyme (cME, Figure 6 ‘18’). Yet despite cME catalyzing a reversible reaction, malate decarboxylation is thermodynamically favored. So this enzyme is considered to be involved in malate degradation during ripening [98]. cME levels rose halfway through green development, while malate accumulated and dropped at mid-ripening. In this sense, cME may not be assigned a unique role in malate degradation during ripening, when large amounts of this compound are released from the vacuole. One such role can be speculated to form part of a biochemical pH-stat, which also involves MDH, PEPC and PEPCK [99].

At the inception of ripening, net malate accumulation switches to net degradation [8] due to its release from the vacuole and its use in different pathways. Gluconeogenesis has been suggested to occur in grapes in ripening stages [100]. As mentioned above, cMDH abundance decreased notably at pre-*véraison,* while two PEPCK isoforms showed a moderate accumulation from the pre-*véraison* stage. The increase in the PEPCK transcripts [14,77], enzyme activity during ripening [95] and the proteins found herein supports its role in gluconeogenesis. However another pathway involving cME and pyruvate diphosphate dikinase (PPDK, Figure 6 ‘17’) may also operate in gluconeogenesis in plants. A transcript encoding a putative PPDK has been seen to increase throughout berry development, while no changes in the expression of the ME isoforms has been detected [77]. The accumulation profiles of cME found herein, from mid-green development to mid-ripening, and PPDK, which peaked at *véraison*, are consistent with this role in a transient gluconeogenic phase at the beginning of ripening. If we bear in mind the switch of the sugar unloading pathway at pre-*véraison*[50], it can be hypothesized that gluconeogenesis at early ripening stages is required to compensate the transient decay of sugars from the time that plasmodesmata are blocked to the full operation of the transporter-mediated apoplastic sugar unloading. The accumulation of PFK-1 and down-stream glycolytic enzymes, including PK, from *véraison* to full ripening indicates that the pathway may flow toward pyruvate once the sugar import is restored once again. Early biochemical and physiological studies provided evidence that gluconeogenesis occurs, but that it is not a major pathway of malate degradation throughout whole berry ripening [90]. Accordingly, the profiles of cMDH, PEPCK, cME and PPDK support gluconeogenesis in early, but not late, ripening stages.

Respiration during ripening is strongly supported by the corresponding profiles of the TCA cycle enzymes and, at least during the first half of the ripening phase, by the profiles of respiratory complexes (see group 3 above about ‘Respiration’). Large quantities of malate released from vacuoles from *véraison* and pyruvate, obtained from either malate or glycolysis, may be transported directly to mitochondria to feed the TCA cycle, to produce ATP and to maintain the respiratory flux in fruit cells. The up-regulation of mMDH (Figure 6 ‘25’) ties in with increasing protein levels and activity at post-*véraison* for MDH [97,101]. The use of pyruvate as a respiratory substrate is supported by the increasing PDH profile (Figure 6 ‘27’). This scenario is in agreement with our hypothesis for a change in the malate degradation metabolism from being supported mainly by respiration at early ripening stages to then occur by fermentation in the cytosol at late ripening. In the late ripening stages, the respiration rate may decrease and induce ethanol synthesis (see group 3 above about ‘Fermentation’).

## Conclusion

Applying iTRAQ to study grape berry development has allowed the identification and quantitation of 411 and 630 proteins in the green and ripe growing phases, respectively. These longer lists of proteins complement a previous gel-based proteomic study with a better proteome coverage and they better connect the two growing phases by analyzing a common time point in development, particularly 15 mm. This technique allowed the detection of another key point in development, 15 mm-to-V100, where most of the dramatic changes at the protein level occurred. The obtained results led to a comprehensive study of grape berry development that supports and complements a previous proteomic analysis [31]. This large-scale proteomic study as a hypothesis-free approach provides quite a complete view of the major and important pathways which evolve during fruit development, thus providing a better understanding of berry development and ripening physiology. These findings help provide an understanding of the metabolism and storage of sugars and malate, energy-related pathways such as respiration, photosynthesis and fermentation, and the synthesis of polyphenolics as major secondary metabolites in grape berries. They all largely determine final berry quality and are of paramount importance for the viticultural industry. A similar approach at the protein level is now feasible to study the effect of other variables of interest (environmental factors and cultural practices) on these pathways. Alternatively, the key steps identified in this study, such as the PFP-PFK or SuSy-INV switches, among others, can be targeted under multiple conditions to finely characterize their influence on the final sugar/acid balance in ripe fruit. Finally, some proteins underwent major changes at specific developmental stages; thus, they can be used as novel protein biomarkers of berry development, which have not been detected to date. Consequently, this may help open up new lines to explore the parameters controlling grape berry development and ripening.

## Methods

### Plant material

Grape berries (*Vitis vinifera* L. cv. Muscat Hamburg) were collected from the experimental vineyard at the Instituto Murciano de Investigación y Desarrollo Agrario (Torrepacheco, Murcia, Spain) in 2006. Berries were sampled from four selected vines and were collected at seven different developmental stages from fruit set until full ripening during both growing seasons. Individual grapes were developmentally staged according to the different berry growth pattern parameters. Green berries were classified into fruit set (stage FS) and then according to the equatorial diameter of fruit in 4, 7 and 15 mm. The beginning of the second berry growth phase, labeled 100% *véraison* (stage V-100), was assessed visually as 100% berry surfaces turned pink in. Ripening berries were classified according to their estimated density by flotation in different NaCl solutions: stage 110 was berries that sink in 110 g/l, but float in 120 g/l, while stage 140 was berries that sink in 140 g/l. Since berries in a bunch do not develop uniformly, berries were harvested from different bunches in the same plant on each sampling day and were then sorted according to their developmental stage, considered to be a sample. Four selected vines were sampled during berry development and ripening to make four biological replicates per stage. Sampled berries were immediately frozen in liquid nitrogen after detaching, except for the 110- and 140-staged berries, which were classified previously according to density. All samples were stored at −80°C until use. A parallel set of sampled berries was refrigerated after detaching and was transported to the laboratory to determine color index, juice pH, total acidity and °Brix, which showed typical profiles for grapevine berries. The methods and results are reported elsewhere [69].

### Protein extraction

The three main berry tissues, seed or endocarp, flesh or mesocarp and skin or exocarp, were differentiated at the different developmental stages, and their dissection was only possible after certain stages. Prior to total protein extraction, seeds were removed from berries from stage 4 mm onward, while exocarp tissue was peeled away in berries from stages V-100 onward. Thus, green berry proteins and ripening berries proteins were extracted from the pericarp and the mesocarp, respectively. The tissue of a pool of berries from the same vine was ground to a fine powder in a mortar with liquid nitrogen and 4 g of ground tissue were used to prepare a protein extract. Protein extracts were obtained as described in Martínez-Esteso *et al*. [31].

The protein was quantified by the Bradford dye-binding method [102] with bovine serum albumin used as a standard and an equal amount of protein from each staged replicate was pooled for isobaric labeling.

### Isobaric peptide labeling

For each developmental stage, a volume corresponding to 100 μg of protein was precipitated with 10 volumes of acetone at −20°C overnight. After centrifugation for 10 min at 15300 × *g*, the protein pellet was dissolved in 60 μl of iTRAQ dissolution buffer (Applied Biosystems) containing 0.2% (w/v) SDS. Proteins were reduced in 3 mM tris-(2-carboxyethyl) phosphine (TCEP) and were incubated for 1 h at 60°C. After cooling samples to RT, cysteine residues were blocked with 2 μl 200 mM methylmethanethiosulfate (MMTS) by incubating at RT for 10 min. Samples were diluted by adding 250 μl of iTRAQ dissolution buffer. Then, 10 μg of proteomics grade modified trypsin (Sigma), dissolved in the same buffer, were added to each vial and digestion was allowed to proceed overnight at 37°C. Afterward, a small pellet remained and 5 μg of proteomics grade modified trypsin (Sigma) dissolved in iTRAQ dissolution buffer were added to each vial and allowed to digest at 37°C for 3 h. The resulting tryptic peptides were vacuum-concentrated and re-suspended in 30 μl of iTRAQ dissolution buffer. The labeling reactions were done following the manufacturer’s recommendations by adding one iTRAQ reagent, 114.11123, 115.10826, 116.11162 or 117.11497, previously dissolved in 70 μl of pure ethanol, to each protein sample vial. The labeling reaction was stopped after 1 h of incubation at RT by adding 1 ml of a buffer containing 10 mM K_2_HPO_4_ and 25% acetonitrile (ACN), pH 2.7, to each vial. In one iTRAQ experiment, reagents were used to label the FS stages 4 mm, 7 mm and 15 mm, and to label the stages 15 mm, V-100, 110 g/l and 140 g/l in another. Then, the four samples were pooled and adjusted to pH 3.0 with concentrated phosphoric acid. As all these experiments were carried out in a different laboratory, the downstream workflow procedures differed slightly.

### Peptide fractionation by strong cation exchange

The pool of labeled samples was fractionated by strong cation exchange chromatography (SCX). Samples were separated using an Äkta Purifier (GE Healthcare) medium pressure liquid chromatography system equipped with a Mono S PC, 1.6 mm × 50 mm column (GE Healthcare) at a flow rate of 0.1 ml/min (green stages), or a BioCAD workstation (Applied Biosystems) utilizing a 100 × 4.6 mm polysulfoethyl aspartamide column (PolyLC Inc, Columbia, MD, USA) at a flow rate of 0.5 ml/min (ripe stages). First, the sample was loaded into the column, 5-fold diluted in buffer A (10 mM K_2_HPO_4_/25% ACN pH 2.7). Afterward, the column was washed with buffer A for 20 min and peptides were eluted with a two-step gradient: first a linear gradient of 5-35% buffer B (0.5 M KCl in 10 mM K_2_HPO_4_/25% ACN pH 2.7) for 30 min, followed by a linear gradient of 35-100% buffer B for 60 min. The elution of peptides was monitored at 280 nm and fractioned into 0.1 ml (green stages) or 214 nm and 0.5 ml (ripe stages) throughout the chromatographic run. For the green stages, the 77 collected fractions were further reduced to 21 by pooling sets of three or four consecutive fractions. The 21 resulting fractions were vacuum-concentrated, resuspended in 100 μl 5% ACN/0.5% trifluoroacetic acid (TFA) and desalted with PepClean™ C-18 Spin Columns (Thermo Fisher Scientific, Rockford, IL) according to the manufacturer’s recommendations. The peptides eluted in 40 μl 70% ACN/0.1% formic acid (FA) were dried under vacuum and re-suspended in 18 μl 0.1% FA. For the ripe stages, 61 fractions were collected, but only 18 containing the eluted labeled peptides measured by optical density monitoring at 214 nm were chosen for the analysis in a 2-hour LC-MS/MS program. The fractionated samples were reduced to 150 μl in a speed-vac (Thermo-Savant, Holbrook, NY, USA) and were transferred to autosampler tubes (LC Packings, Amsterdam, The Netherlands).

### Reverse phase chromatography

An integrated system consisting of a Famos Autosampler, a Switchos switching pump and a UltiMate micropump (LC Packings, Amsterdam, Netherlands) was used for the reverse phase chromatography on the selected SCX fractions. For the green stages, 5 μl of the tryptic peptides were pre-concentrated in a C18 PepMap guard column (300 μm i.d. × 5 mm 5 μm, 100 Å, LC Packings, Amsterdam, The Netherlands) at 40 μl/min for 3 min in 0.1% FA, followed by elution in a C18 PepMap (75 μm i.d. × 15 cm, 3 μm, 100 Å, LC Packings, Amsterdam, The Netherlands) using a 120 min linear gradient from 15 to 50% solvent B; solvent A was 0.1% FA in water and solvent B was 0.1% FA in 95% ACN. For the ripe stages, one fifth of each SCX fraction was desalted in a C18 PepMap guard column (300 μm i.d. × 5 mm 5 μm, 100 Å, LC Packings, Amsterdam, The Netherlands) at 50 μl/min for 15 minutes. HPLC buffers consisted of Buffer A −2% ACN, 0.1% FA and Buffer B −98% ACN and 0.1% FA. Peptides were separated ina manually packed 75 μm × 15 cm C18 column (Magic C18Aq, 5 μm, 100 Å, Michrom Bioresources Inc., Auburn CA, USA) using an 85 min gradient of 5%-75% buffer B flowing at 250 nl/min for fractions 37–48 and a 35 min gradient of 5%-75% buffer B.

### Mass spectrometry

The eluent was sprayed directly into either a QSTAR XL System (green stages) or a QSTAR Pulsar I (ripe stages) mass spectrometer equipped with a nanospray source (Applied Biosystems/MDS SCIEX Concord, ON Canada). The QSTAR operating software Analyst QS v1.1 employed an information dependent acquisition (IDA) method for optimized MS/MS spectra acquisition over a 6-second cycle, which was repeated throughout gradient duration. The MS-TOF survey scan lasted 1 second over the range of 400–1200 *m/z* targeting ions of charge state 2-4+, which exceeded a threshold of 20 counts. The former target ions within 100 ppm were excluded for the next 180 seconds. Each product ion scan lasted 2.5 seconds over a range of 100–1500 *m/z*. Enhance all was turned on for the product ion scans.

### Database search and protein quantitation

Raw data files were processed using Protein Pilot v1.0 with the Paragon Search and ProGroup Algorithms™ (Applied Biosystems/MDS Sciex Foster City, CA USA) for both tryptic peptide identification and quantitation. The peptides and corresponding relative abundances were obtained in ProteinPilot using a confidence cutoff (called a ‘Prot Score’) of >1.0 (>90%) and >1.3 (>95%) for the experiments of the green and ripe stages, respectively. Database searching for each sample was done against the NCBInr protein database without taxonomical restrictions, trypsin, MMTS as a fixed modification and the iTRAQ label as a variable modification. Only the proteins identified with at least 2 different peptides and p<0.05, and quantified with a ratio of >1.5 and p<0.05, were considered. The former p-value related to the protein score cutoff in the identification, while the latter p-value related to the iTRAQ ratio for each quantified protein and was computed from the ProGroup Algorithm in the ProteinPilot software as a measure of its statistical significance. The sequences the from keratins, trypsin and species other than plants were not considered.

### Bioinformatic functional analysis

Since the translated ORFs from the grapevine genome projects [34,35] have been deployed in public databases without descriptions and annotations to date, searches often match an undescribed amino acid sequence. The Blast2GO v2.4.0 application [43] has been used to automatically assign protein description and take-up annotations from homologous sequences of public databases, which have then been manually reviewed and enriched, if possible. A file of the FASTA format sequences of the identified and/or quantified protein set was batch-retrieved from the NCBI website. Blast2GO was fed with the FASTA file and was run to: first incorporate the sequence description by performing a BLASTp search against NCBInr (e-value cutoff of 1 × 10^-50^, 20 for the retrieved number of BLAST hits, 33 for the HSP (highest scoring pair) length cutoff); second to map the GO, EC and Interpro terms; then to annotate the sequences (E-Value Hit-Filter of 1 × 10^-6^, a Hsp-Hit Coverage Cutoff of 0, an Annotation Cutoff of 55, and a GO Weight of 5). The automatic annotation performed by Blast2GO was manually revised to guarantee accurate assignment. Fisher exact tests [45] were performed to find the significantly enriched GO terms (FDR <5%). Briefly, the frequencies of the annotation terms were compared between a reference list containing all the quantified sequences in the experiment and a subset list of sequences selected through an arbitrary quantitation ratio cutoff of 1.5-fold (ratio <0.6 for the down-regulated and >1.5 for the up-regulated proteins). Subsets were selected at each developmental stage transition analyzed: FS-to-4 mm, 4 mm-to-7 mm, 7 mm-to 15 mm, 15 mm-to-V100, V100-to-110 and 110-to-140.

## Abbreviations

ACs: Anthocyanins; DIGE: Difference gel electrophoresis; FS: Fruit set; V100: Full color change at véraison; GO: Gene ontology; GST: Gluthatione-S-transferase; HSPs: Heat shock proteins; iTRAQ: Isobaric tags for relative and absolute protein quantitation; HPLC-MS/MS: Liquid chromatography coupled with tandem mass spectrometry; cMDH: Malate dehydrogenase cytosolic; mMDH: Malate dehydrogenase mitochondrial; PFK-1: Phosphofructokinase 1; PACs: Proanthocyanidins; PFP: Pyrophosphate-dependent phosphofructokinase; sHSP: Small HSP; SCX: Strong cation exchange chromatography; SuSy: Sucrose synthase; TFA: Trifluoroacetic acid; 2-DE: Two-dimensional gel electrophoresis; UDP-GP: UDP-glucose pyrophosphorylase; vINV: Vacuolar acid invertase.

## Authors’ contributions

MJME contributed to the experimental design, grape sampling, protein extraction, labeling and SCX fractionation, bioinformatic analysis and biological and data interpretation. MTV contributed to grape sampling, protein extraction and labeling. MLV carried out the mass spectrometric analysis for the protein identification and quantification in the iTRAQ experiment of the green stages. MAP contributed to the experimental design and data interpretation. RBM defined the work objectives and technical approach, and contributed to the experimental design, bioinformatic analysis and data interpretation. This article is part of María José Martínez-Esteso’s PhD Thesis. All authors read and approved the final manuscript.

## Supplementary Material

Additional file 1 List of proteins identified and quantified for the iTRAQ experiment of the green stages, including the protein and peptide output files. Click here for file

Additional file 2 **Processed output file (Additional file ****1****), including the protein description annotation by Blast2GO v2.4.0 application**[43]. Click here for file

Additional file 3 List of proteins identified and quantified for the iTRAQ experiment of the ripe stages, including the protein and peptide output files. Click here for file

Additional file 4 **Processed output file (Additional file **3**), including the protein description annotation by Blast2GO v2.4.0 application**[43]. Click here for file

Additional file 5 Distribution of the annotations of biological processes and cellular components. Click here for file

Additional file 6 **File containing the GO-terms annotated by the Blast2GO v2.4.0 application **[42]** for the proteins identified in the iTRAQ experiment of the green stages.** Click here for file

Additional file 7 **File containing the GO-terms annotated by Blast2GO v2.4.0 application **[42]** for the proteins identified in the iTRAQ experiment of the ripe stages.** Click here for file

Additional file 8 The GO terms enriched and sequences annotated with such terms in the up- or down-regulated subsets of proteins in each grape berry developmental stage step analyzed. Click here for file

Additional file 9 **Profiles of protein functional clusters during berry development **[103]**.** Click here for file

Additional file 10 Methione synthesis and connection with folate metabolism in grape berries during development. Click here for file

Additional file 11 Results and discussion about the functional groups ‘Nitrogen and amino acid metabolism’, ‘Terpenoid metabolism’, ‘Signaling and hormone’, ‘Stress’, ‘Protein synthesis’, ‘Protein degradation’, ‘Protein processing’, ‘Cell division and growth, biogenesis’, ‘Defense proteins’, ‘Other proteins of interest’. Click here for file

Additional file 12 **Phylogram of the grapevine GSTs identified in grape berry during development **[104]**.** Click here for file

Additional file 13 Scenario of the photosynthetic machinery of both the light and dark phases during grape berry development. Click here for file
