# Supplementary material for: iTRAQ-based protein profiling provides insights into the central metabolism changes driving grape berry development and ripening
Source: BMC Plant Biol. 2013 Oct 24;13:167. doi: 10.1186/1471-2229-13-167 (PMC4016569; doi:10.1186/1471-2229-13-167)
Supplement: Additional file 5 — Distribution of the annotations of biological processes and cellular components. [file 1471-2229-13-167-S5.pdf]

Additional File 5. Distribution of the annotations of biological processes and cellular components. Only the GO terms annotating a minimum number of sequences are shown. Thus, the biological process (A) and the cellular components (B) charts are constructed with GO terms annotating at least a 10% and 5% of the whole set of sequences, respectively.

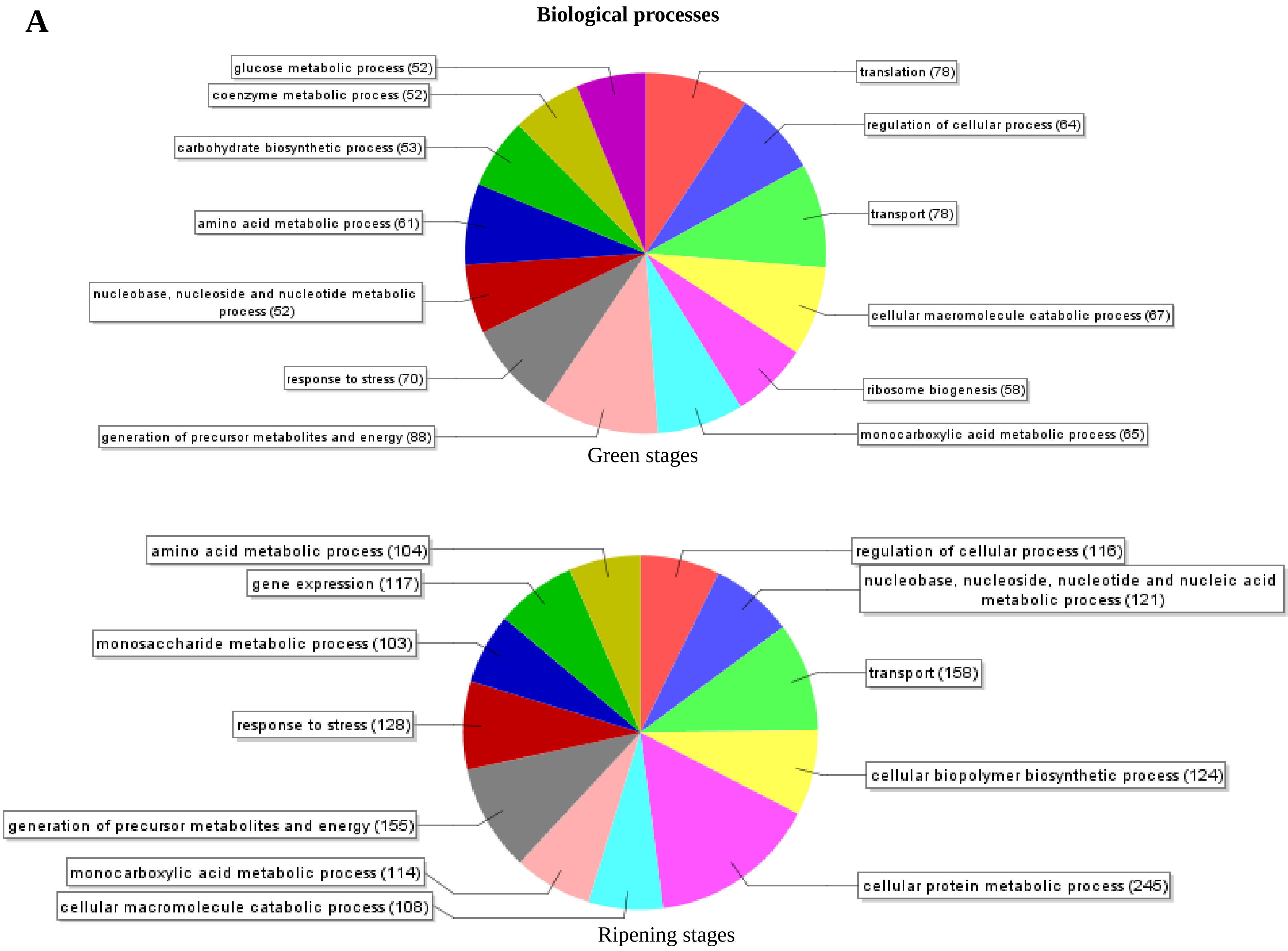
A
Biological processes
glucose metabolic process (52)
coenzyme metabolic process (52)
carbohydrate biosynthetic process (53)
amino acid metabolic process (61)
nucleobase, nucleoside and nucleotide metabolic process (52)
response to stress (70)
generation of precursor metabolites and energy (88)
translation (78)
regulation of cellular process (64)
transport (78)
cellular macromolecule catabolic process (67)
ribosome biogenesis (58)
monocarboxylic acid metabolic process (65)
Green stages
amino acid metabolic process (104)
gene expression (117)
monosaccharide metabolic process (103)
response to stress (128)
generation of precursor metabolites and energy (155)
monocarboxylic acid metabolic process (114)
cellular macromolecule catabolic process (108)
regulation of cellular process (116)
nucleobase, nucleoside, nucleotide and nucleic acid metabolic process (121)
transport (158)
cellular biopolymer biosynthetic process (124)
cellular protein metabolic process (245)
Ripening stages

B

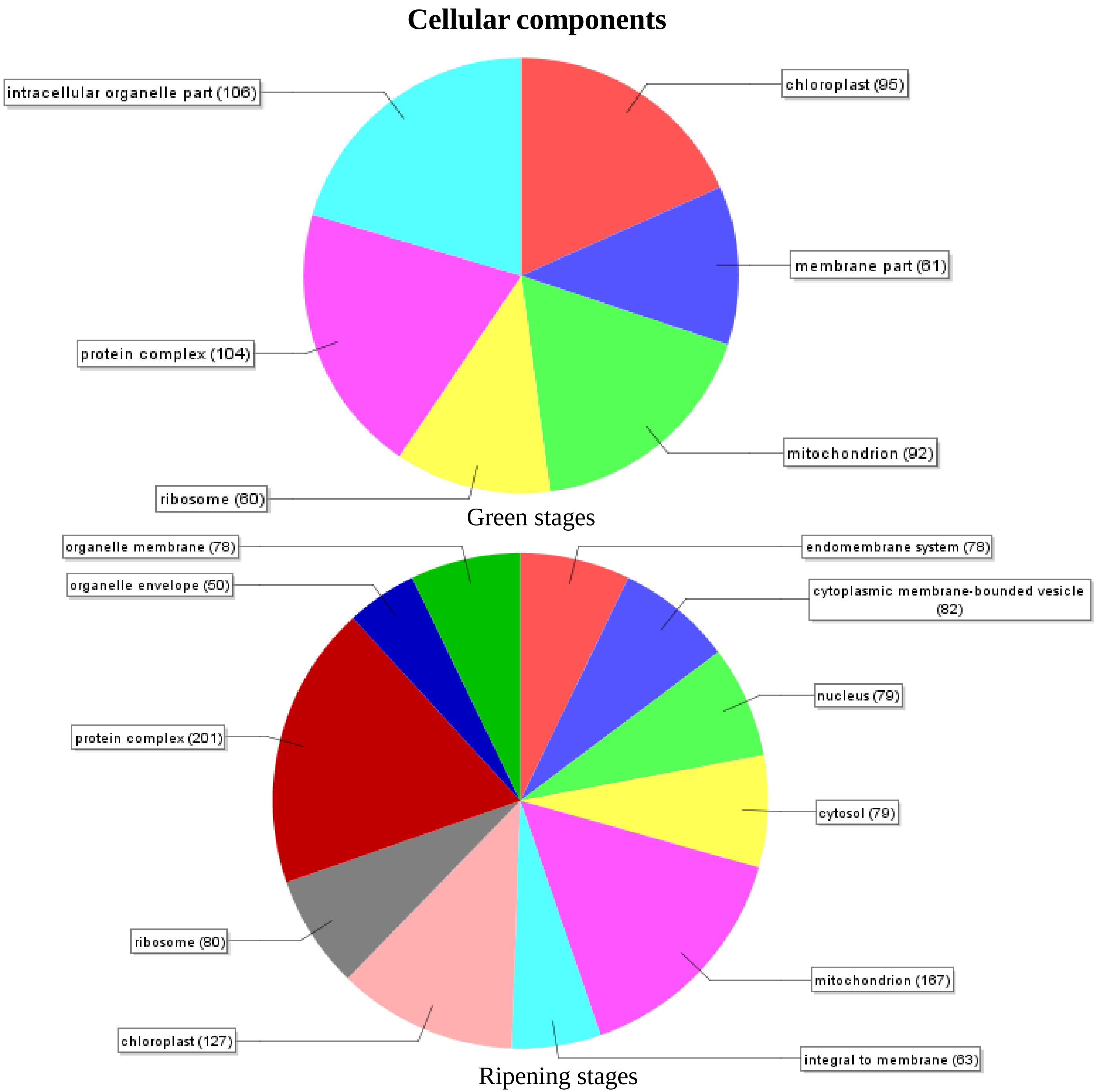
Cellular components
intracellular organelle part (106)
chloroplast (95)
membrane part (61)
protein complex (104)
mitochondrion (92)
ribosome (60)
Green stages
organelle membrane (78)
endomembrane system (78)
organelle envelope (50)
cytoplasmic membrane-bounded vesicle (82)
nucleus (79)
protein complex (201)
cytosol (79)
ribosome (80)
mitochondrion (167)
chloroplast (127)
integral to membrane (63)
Ripening stages

As seen in Additional file 5, major cellular components (C terms) and biological processes (P terms) are well-represented. The proteins identified in both experiments represent a broad range of biological processes, although specific GO terms are particularly enriched in each development phase. The terms ‘translation,’ ‘ribosome biogenesis’ and ‘glucose metabolic process’ appear in the green stages, which reflects the main processes taking place in early development stages characterized by intense cell division activity, protein synthesis and the conversion of sugar into organic acids. Conversely in the ripe stages, terms related with carbohydrate metabolism appear as this phase is characterized by the accumulation of sugars during ripening. Additional figure 5 shows the sequence distribution according to the GO annotation. Additional figure 5A depicts the biological processes annotated in at least 10% of the sequences, namely 103 sequences for the ripening stages experiment and 52 for the green ones. It can be seen that the term ‘cellular protein metabolic process’, associated with protein fate and protein folding, is enriched in the ripe stages. In fact the onset of ripening is characterized by the up-regulation of numerous heat shock proteins (HSPs), which is consistent with the major redirection of development and metabolism occurring in this stage and the need to stabilize pre-existing and newly synthesized proteins with this changing physiochemical environment (da Silva *et al*., 2005).
