# Supplementary material for: iTRAQ-based protein profiling provides insights into the central metabolism changes driving grape berry development and ripening
Source: BMC Plant Biol. 2013 Oct 24;13:167. doi: 10.1186/1471-2229-13-167 (PMC4016569; doi:10.1186/1471-2229-13-167)
Supplement: Additional file 8 — The GO terms enriched and sequences annotated with such terms in the up- or down-regulated subsets of proteins in each grape berry developmental stage step analyzed. [file 1471-2229-13-167-S8.zip › Additional file 8/4 mm to 7 down.doc]

| **Cellular component** | **GO:0005634** : **nucleous** | 0.0439348 | gi\|147781362\|emb\|CAN67222.1\|,  gi\|156322163\|ref\|XP_001618301.1\|,  gi\|147841321\|emb\|CAN60181.1\|, gi\|62286683\|sp\|Q9M531.1\|H2A_EUPES, gi\|157341729\|emb\|CAO62284.1\| **sar1 gtp-binding secretory factor (___)** |
| --- | --- | --- | --- |
|  | **GO:0000786: nucleosome**  **4mm to 7-down**  **GO: 0000785: chromatin**  **GO:0005694: chromosome GO:0044427: chromosomal part** | 4.66052E-5 | gi\|147781362\|emb\|CAN67222.1\| **histone h2b (0.60)**  gi\|156322163\|ref\|XP_001618301.1\| **protein (0.63)**  gi\|147841321\|emb\|CAN60181.1\| **histone h2a (0.58)**  gi\|62286683\|sp\|Q9M531.1\|H2A_EUPES **histone h2a (0.55)** |
| **Biological process** | **GO:0065004-protein-DNA complex assembly**  **GO:0006334-nucleosome assembly**  **GO: 0031497-chromatin assembly**  **GO: 0051276- chromosome organization and biogenesis**  **GO:0007001- chromosome organization and biogenesis (sensu Eukaryota)**  **GO:006323- DNA packaging**  **GO:006259- DNA metabolic process**  **GO:0065003- macromolecular complex assembly**  **GO:0022607- cellular component assembly** | 4.66052E-5 | gi\|147781362\|emb\|CAN67222.1\|,  gi\|156322163\|ref\|XP_001618301.1\|,  gi\|147841321\|emb\|CAN60181.1\|, gi\|62286683\|sp\|Q9M531.1\|H2A_EUPES |
|  | **GO:0009698- phenylpropanoid metabolic process** | 0.0229821 | gi\|147801854\|emb\|CAN74850.1\| **phenylalanine ammonia-lyaseo (0.67)**  gi\|147799448\|emb\|CAN61338.1\| **dihydroflavonol 4-reductase (0.61)**  gi\|18376655\|dbj\|BAB84112.1\| **chalcone synthase (0.54)** |
|  | **GO:0019748-**  **secondary metabolic process** | 0.0229821 | gi\|118406886\|gb\|ABK81651.1\| **glutathione s-transferase (0.62)**  gi\|147801854\|emb\|CAN74850.1\|,  gi\|147799448\|emb\|CAN61338.1\|,  gi\|18376655\|dbj\|BAB84112.1\| |
| **Molecular function** | **GO:0003677:**  **DNA binding** | 0.00275792 | gi\|147781362\|emb\|CAN67222.1\|, gi\|156322163\|ref\|XP_001618301.1\|, gi\|147841321\|emb\|CAN60181.1\|, gi\|62286683\|sp\|Q9M531.1\|H2A_EUPES |
