# Supplementary material for: iTRAQ-based protein profiling provides insights into the central metabolism changes driving grape berry development and ripening
Source: BMC Plant Biol. 2013 Oct 24;13:167. doi: 10.1186/1471-2229-13-167 (PMC4016569; doi:10.1186/1471-2229-13-167)

Additional File 9. Profiles of protein functional clusters during berry development. A heat map of the relative abundance in relation to the 15mm stage (the log_2-normalized ratio) of proteins during development was created by Genesis v1.0 [103]. For each protein, the gene index accession number and the sequence description assigned with Blast2GO are provided. Proteins were grouped according to their known or putative role in metabolic pathways or cellular processes. A) Transporters; B) Polyphenols (I, Phenylpropanoids; II, Flavonoids; III, Others; IV, aromatic amino acids); C) Photosynthesis, respiration and fermentation (I, Photosynthetic; II, Respiratory); D) Carbohydrate and malate metabolism (I, Sucrose-glycolysis/gluconeogenesis; II, Organic acid and TCA cycle; III, Pentose phosphate; IV, Carbon fixation; V, Nucleotide-sugars; VI, Glycosidases, glycosyltransferases); E) Nitrogen and amino acid metabolism (I, Asp, Glu, Gln; II, Ser, Gly, Met, Cys; III, Other amino acids; IV, Nucleotides; V, Coenzymes; VI, GABA); F) Lipid metabolism (I, Fatty acid; II, Phospholipid; III, Isoprenoid); G) Signaling and Hormone (I, Plasma membrane; II, Nucleous; III, Hormone synthesis; IV, Others); H) Stress (I, Oxidative; II, General); I) Protein synthesis (I, Gene expression and RNA; II, Translation; III, Ribosome; IV, Folding); J) Protein degradation (I, Proteasome-UB; II, Other); K) Protein processing (I, Trafficking; II, PTMs); L) Cell division and growth, biogenesis (I, replication; II, Vesicular trafficking; III, Cell wall biogenesis and modification; IV, Cell shape); M) Defense proteins; N) Other proteins of interest; O) Unknown proteins.

Transporters (Additional file 9A)

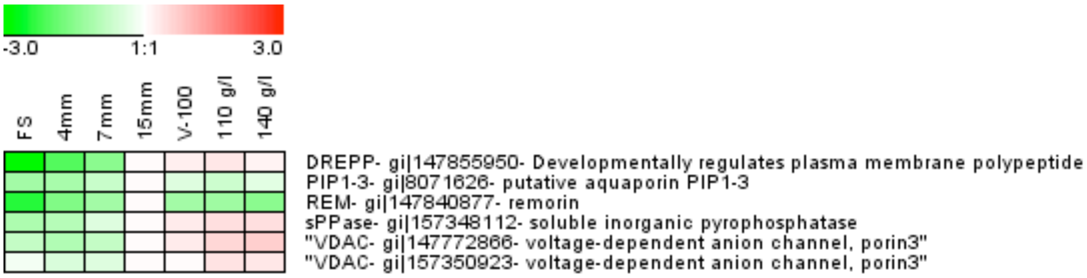

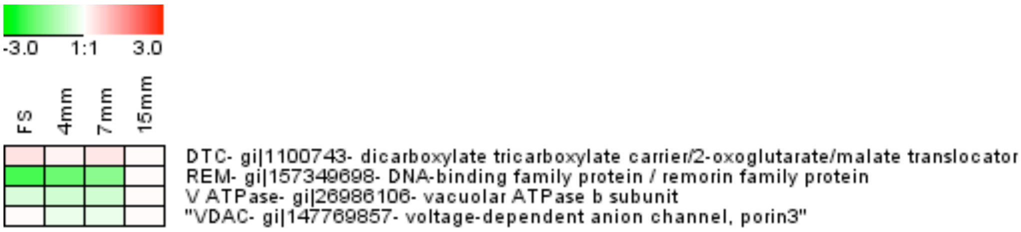

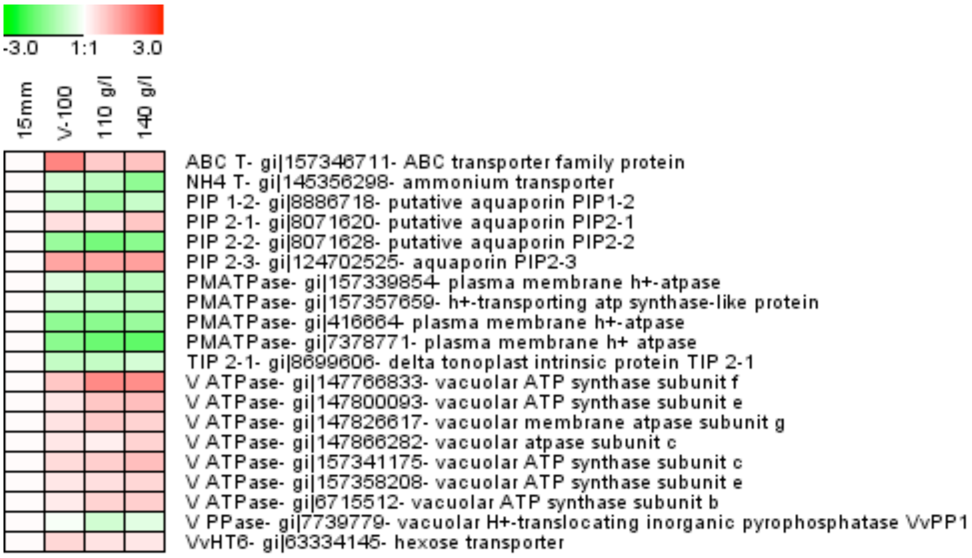

Polyphenols (Additional file 9B)

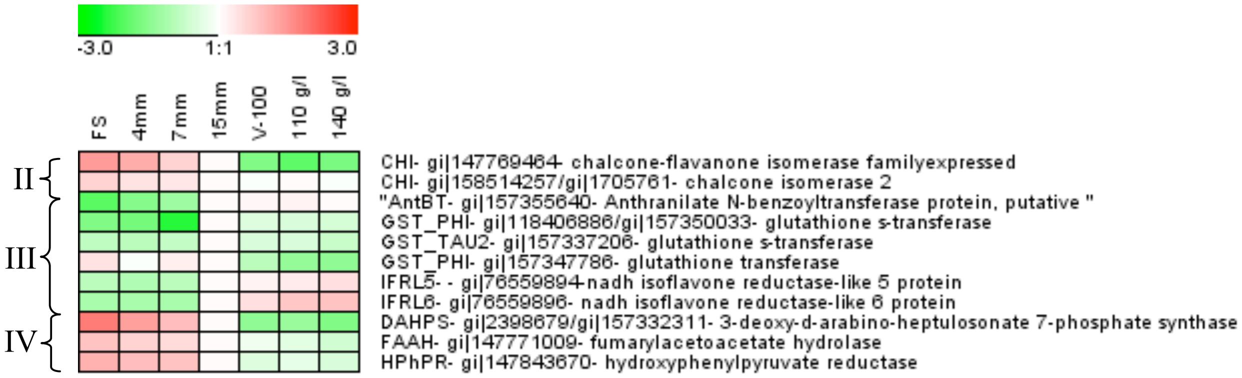

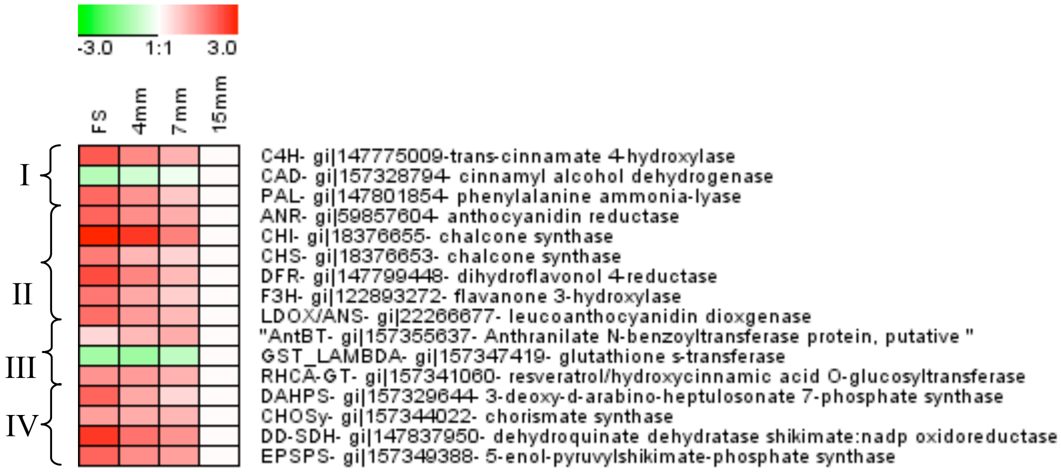

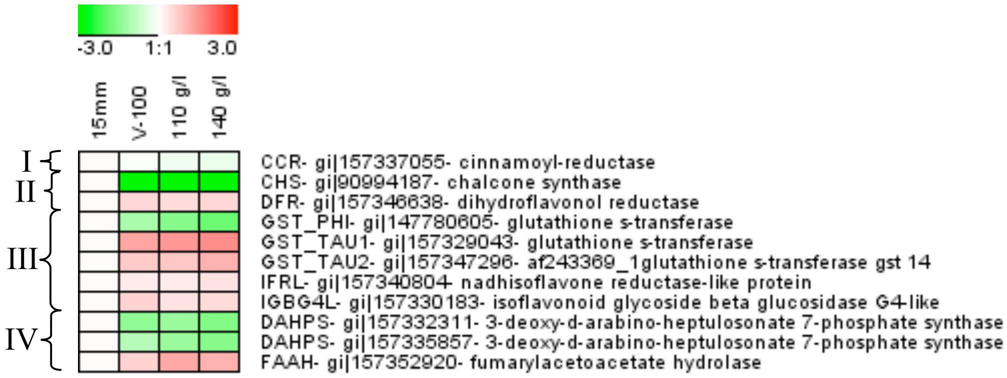

Photosynthesis, respiration and fermentation (Additional file 9C)

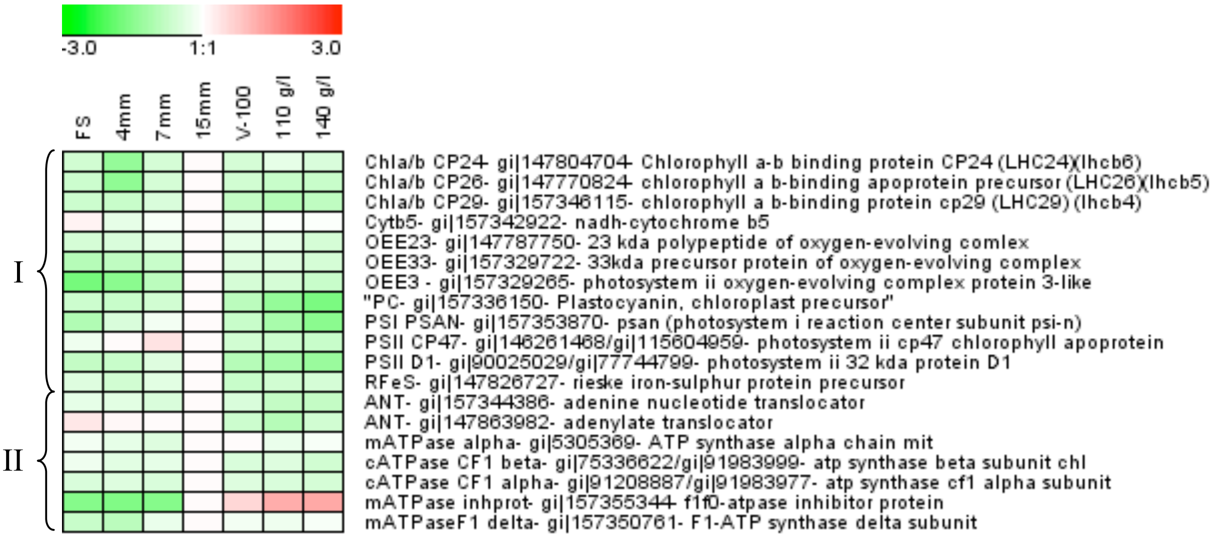

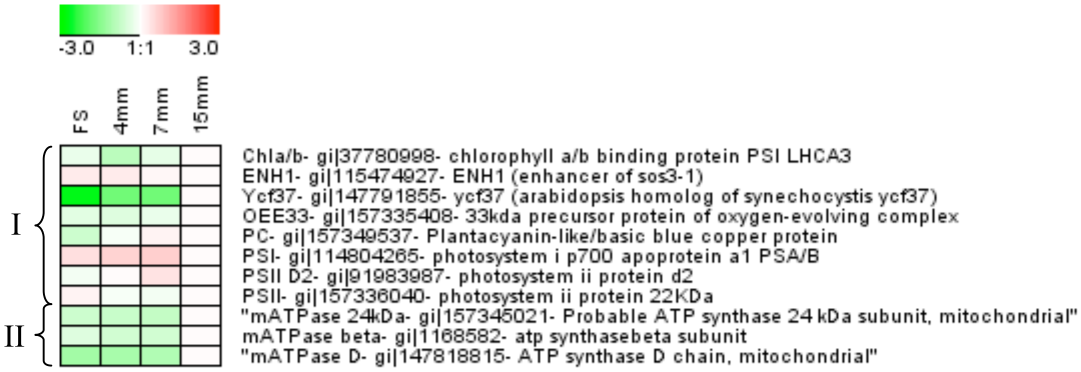

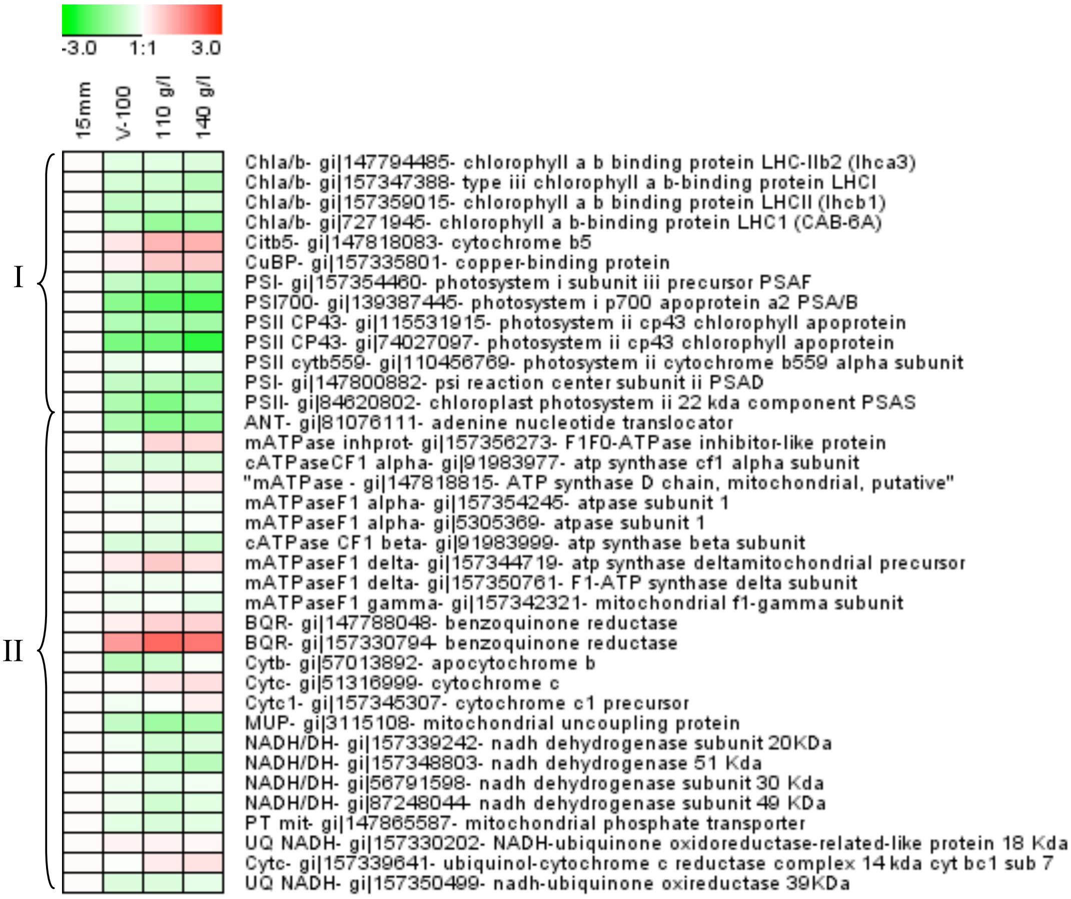

Carbohydrate and malate metabolism (Additional file 9D)

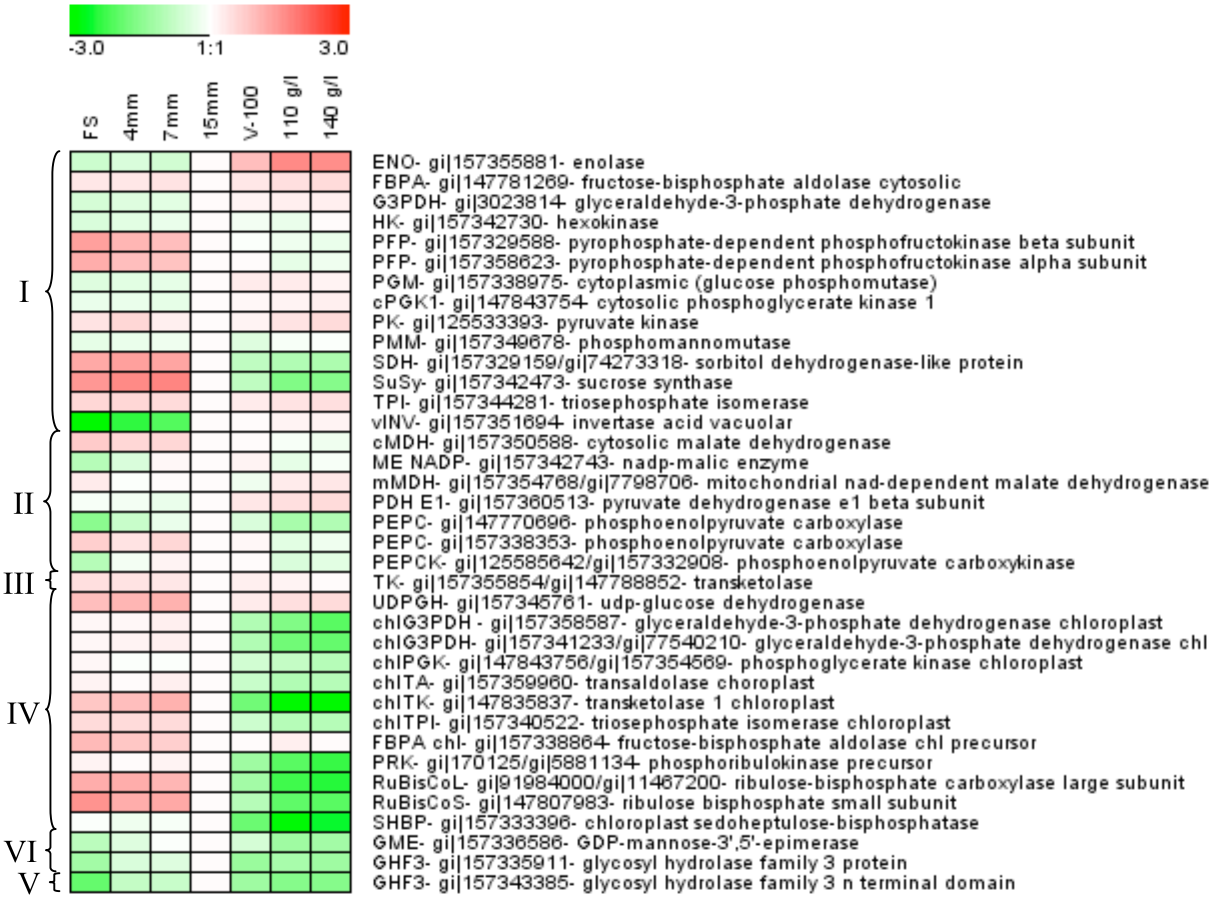

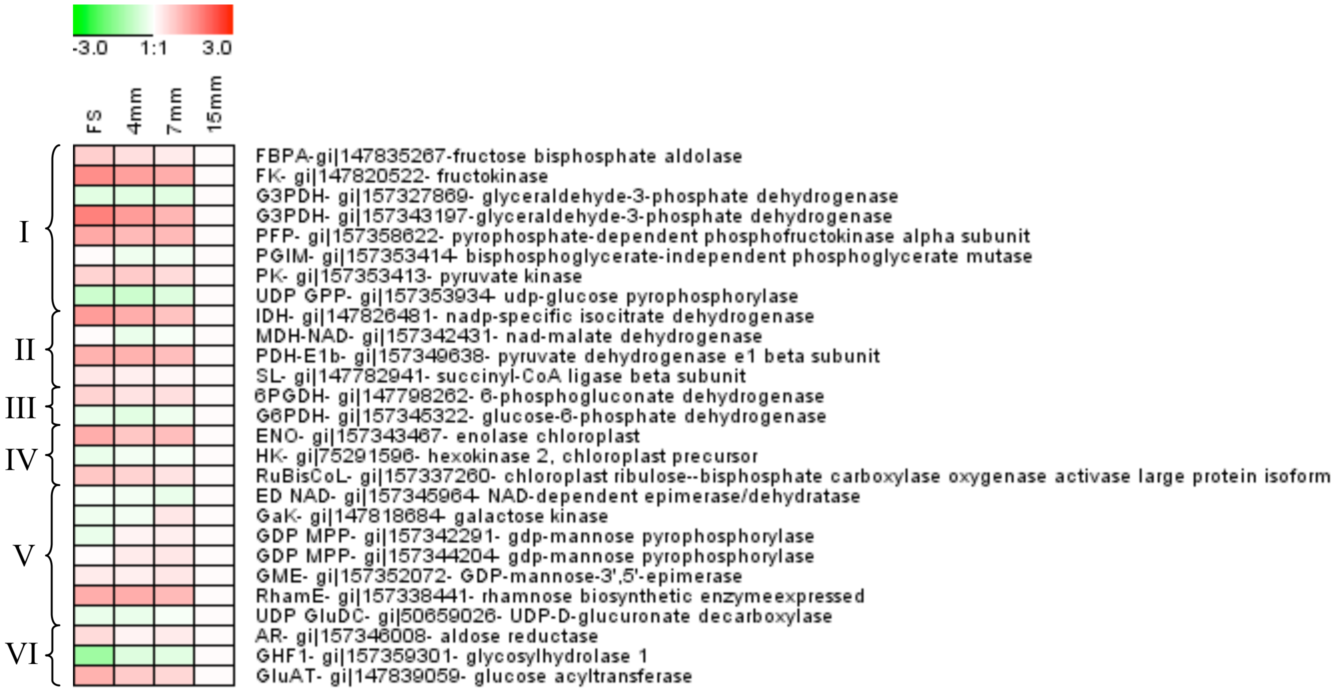

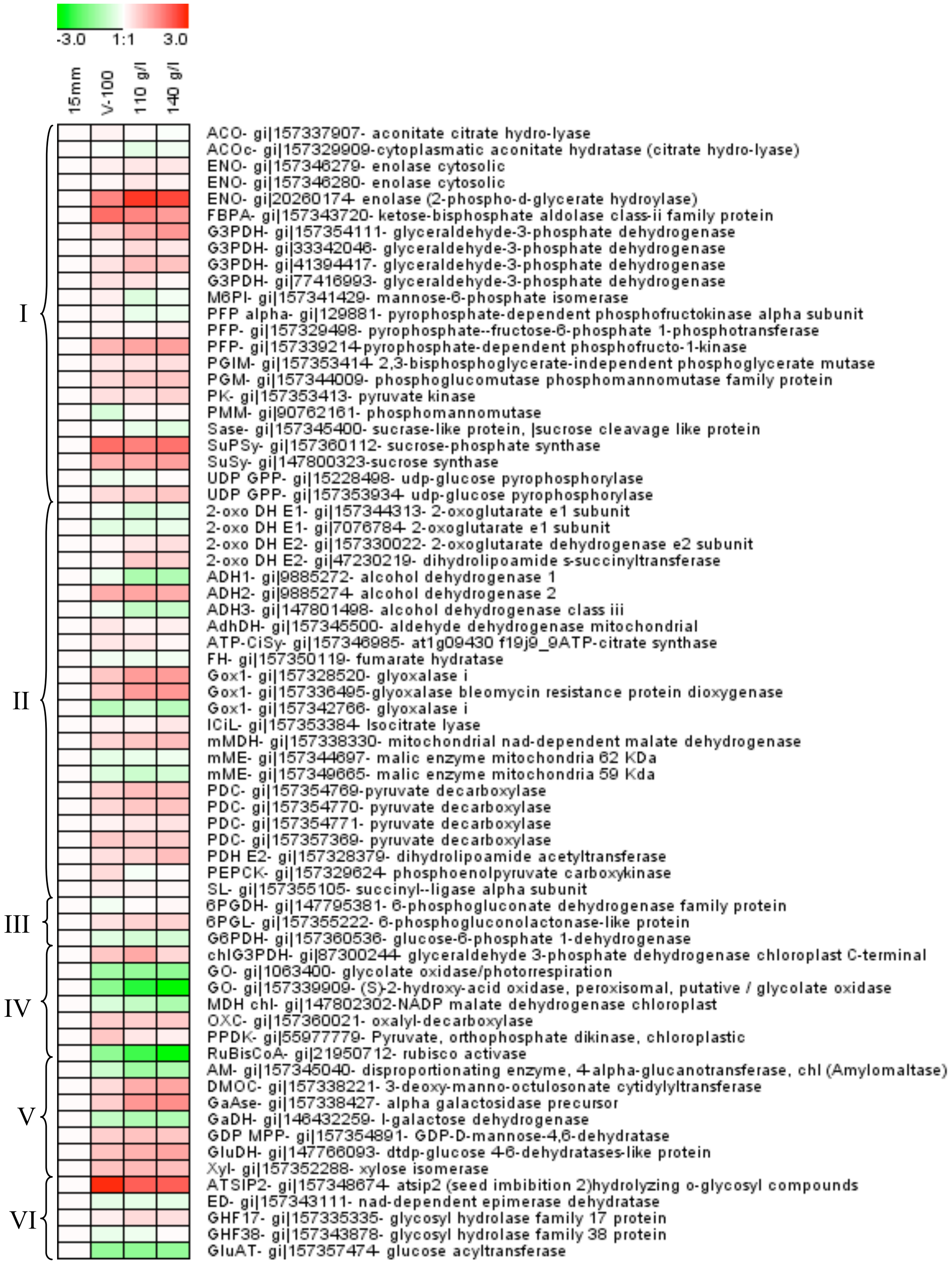
-3.0
1:1
3.0
15mm
V-100
110 g/l
140 g/l
I
II
III
IV
V
VI
ACO- gi|157337907- aconitate citrate hydro-lyase
ACOc- gi|157329909-cytoplasmatic aconitate hydratase (citrate hydro-lyase)
ENO- gi|157346279- enolase cytosolic
ENO- gi|157346280- enolase cytosolic
ENO- gi|20260174- enolase (2-phospho-d-glycerate hydroylase)
FBPA- gi|157343720- ketose-bisphosphate aldolase class-ii family protein
G3PDH- gi|157354111- glyceraldehyde-3-phosphate dehydrogenase
G3PDH- gi|33342046- glyceraldehyde-3-phosphate dehydrogenase
G3PDH- gi|41394417- glyceraldehyde-3-phosphate dehydrogenase
G3PDH- gi|77416993- glyceraldehyde-3-phosphate dehydrogenase
M6PI- gi|157341429- mannose-6-phosphate isomerase
PFP alpha- gi|129881- pyrophosphate-dependent phosphofructokinase alpha subunit
PFP- gi|157329498- pyrophosphate--fructose-6-phosphate 1-phosphotransferase
PFP- gi|157339214-pyrophosphate-dependent phosphofructo-1-kinase
PGIM- gi|157353414- 2,3-bisphosphoglycerate-independent phosphoglycerate mutase
PGM- gi|157344009- phosphoglucomutase phosphomannomutase family protein
PK- gi|157353413- pyruvate kinase
PMM- gi|90762161- phosphomannomutase
Sase- gi|157345400- sucrase-like protein, |sucrose cleavage like protein
SuPSy- gi|157360112- sucrose-phosphate synthase
SuSy- gi|147800323-sucrose synthase
UDP GPP- gi|15228498- udp-glucose pyrophosphorylase
UDP GPP- gi|157353934- udp-glucose pyrophosphorylase
2-oxo DH E1- gi|157344313- 2-oxoglutarate e1 subunit
2-oxo DH E1- gi|7076784- 2-oxoglutarate e1 subunit
2-oxo DH E2- gi|157330022- 2-oxoglutarate dehydrogenase e2 subunit
2-oxo DH E2- gi|47230219- dihydrolipoamide s-succinyltransferase
ADH1- gi|9885272- alcohol dehydrogenase 1
ADH2- gi|9885274- alcohol dehydrogenase 2
ADH3- gi|147801498- alcohol dehydrogenase class iii
AdhDH- gi|157345500- aldehyde dehydrogenase mitochondrial
ATP-CiSy- gi|157346985- at1g09430 f19j9_9ATP-citrate synthase
FH- gi|157350119- fumarate hydratase
Gox1- gi|157328520- glyoxalase i
Gox1- gi|157336495-glyoxalase bleomycin resistance protein dioxygenase
Gox1- gi|157342766- glyoxalase i
ICiL- gi|157353384- Isocitrate lyase
mMDH- gi|157338330- mitochondrial nad-dependent malate dehydrogenase
mME- gi|157344697- malic enzyme mitochondria 62 KDa
mME- gi|157349665- malic enzyme mitochondria 59 Kda
PDC- gi|157354769-pyruvate decarboxylase
PDC- gi|157354770- pyruvate decarboxylase
PDC- gi|157354771- pyruvate decarboxylase
PDC- gi|157357369- pyruvate decarboxylase
PDH E2- gi|157328379- dihydrolipoamide acetyltransferase
PEPCK- gi|157329624- phosphoenolpyruvate carboxykinase
SL- gi|157355105- succinyl--ligase alpha subunit
6PGDH- gi|147795381- 6-phosphogluconate dehydrogenase family protein
6PGL- gi|157355222- 6-phosphogluconolactonase-like protein
G6PDH- gi|157360536- glucose-6-phosphate 1-dehydrogenase
chlG3PDH- gi|87300244- glyceraldehyde 3-phosphate dehydrogenase chloroplast C-terminal
GO- gi|1063400- glycolate oxidase/photorrespiration
GO- gi|157339909- (S)-2-hydroxy-acid oxidase, peroxisomal, putative / glycolate oxidase
MDH chl- gi|147802302-NADP malate dehydrogenase chloroplast
OXC- gi|157360021- oxalyl-decarboxylase
PPDK- gi|55977779- Pyruvate, orthophosphate dikinase, chloroplastic
RuBisCoA- gi|21950712- rubisco activase
AM- gi|157345040- disproportionating enzyme, 4-alpha-glucanotransferase, chl (Amylomaltase)
DMOC- gi|157338221- 3-deoxy-manno-octulosonate cytidylyltransferase
GaAse- gi|157338427- alpha galactosidase precursor
GaDH- gi|146432259- l-galactose dehydrogenase
GDP MPP- gi|157354891- GDP-D-mannose-4,6-dehydratase
GluDH- gi|147766093- dtdp-glucose 4-6-dehydratases-like protein
Xyl- gi|157352288- xylose isomerase
ATSIP2- gi|157348674- atsip2 (seed imbibition 2)hydrolyzing o-glycosyl compounds
ED- gi|157343111- nad-dependent epimerase dehydratase
GHF17- gi|157335335- glycosyl hydrolase family 17 protein
GHF38- gi|157343878- glycosyl hydrolase family 38 protein
GluAT- gi|157357474- glucose acyltransferase

Nitrogen and amino acid metabolism (Additional file 9E)

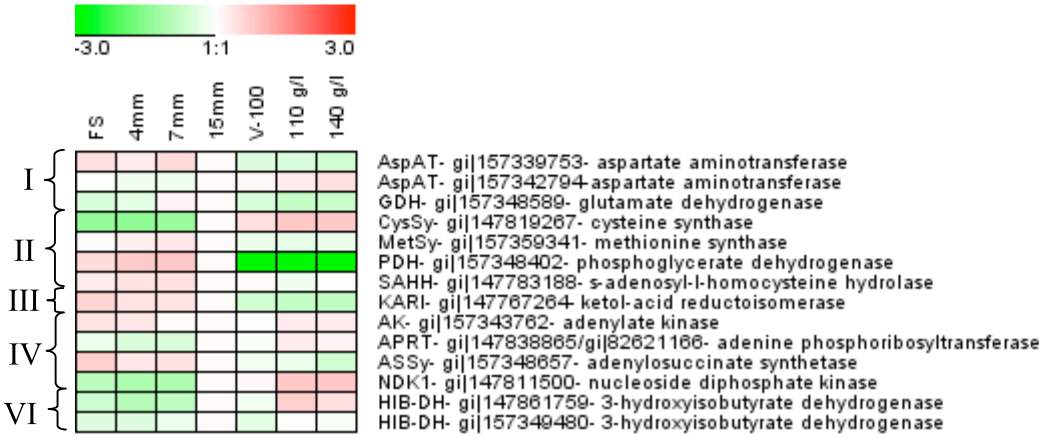

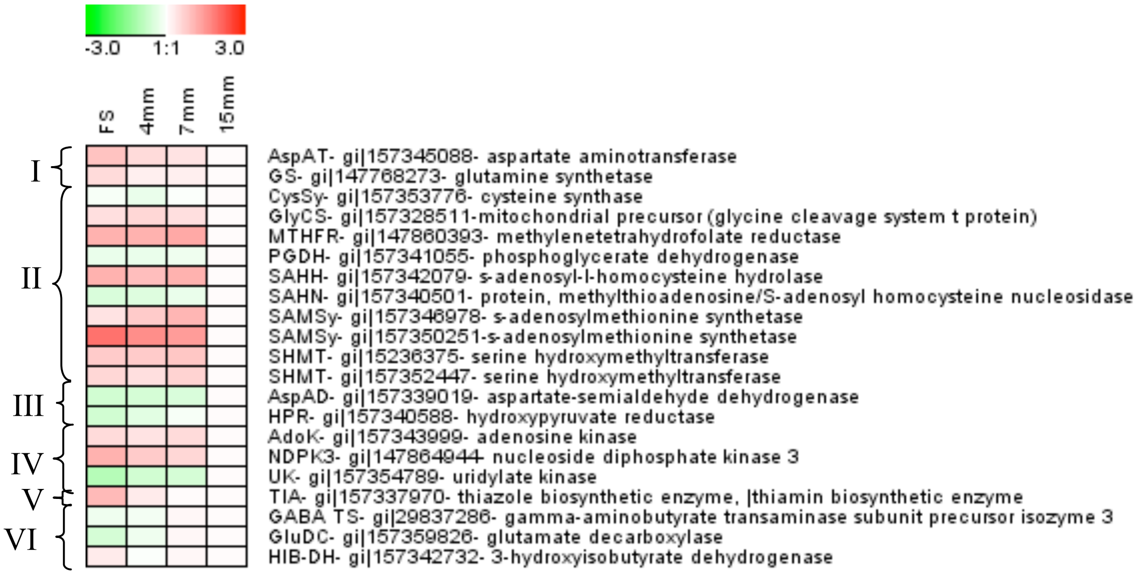

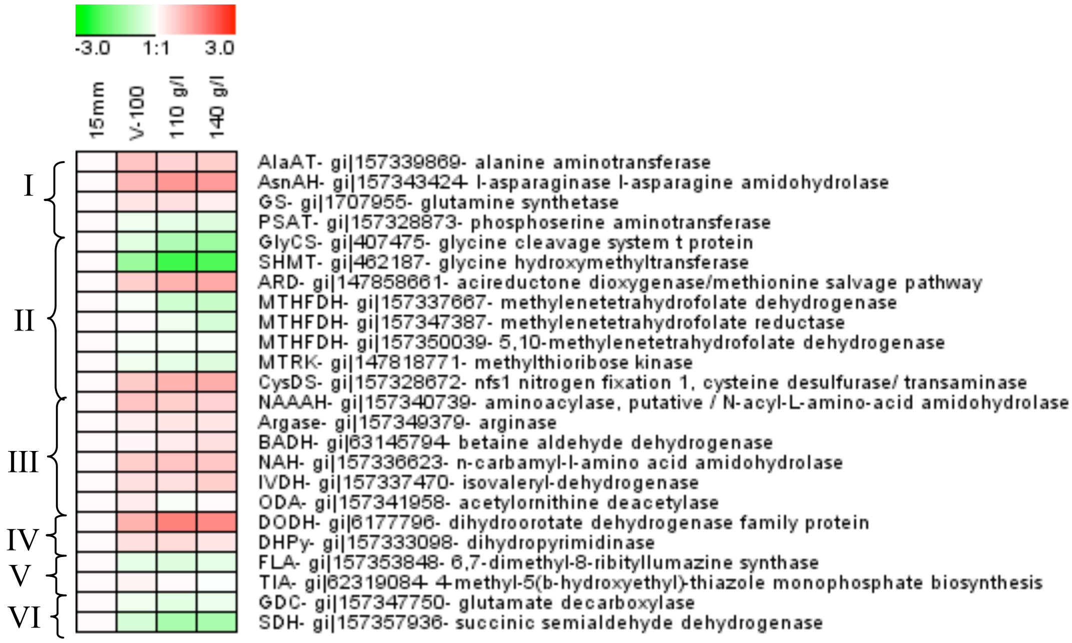

Lipid metabolism (Additional file 9F)

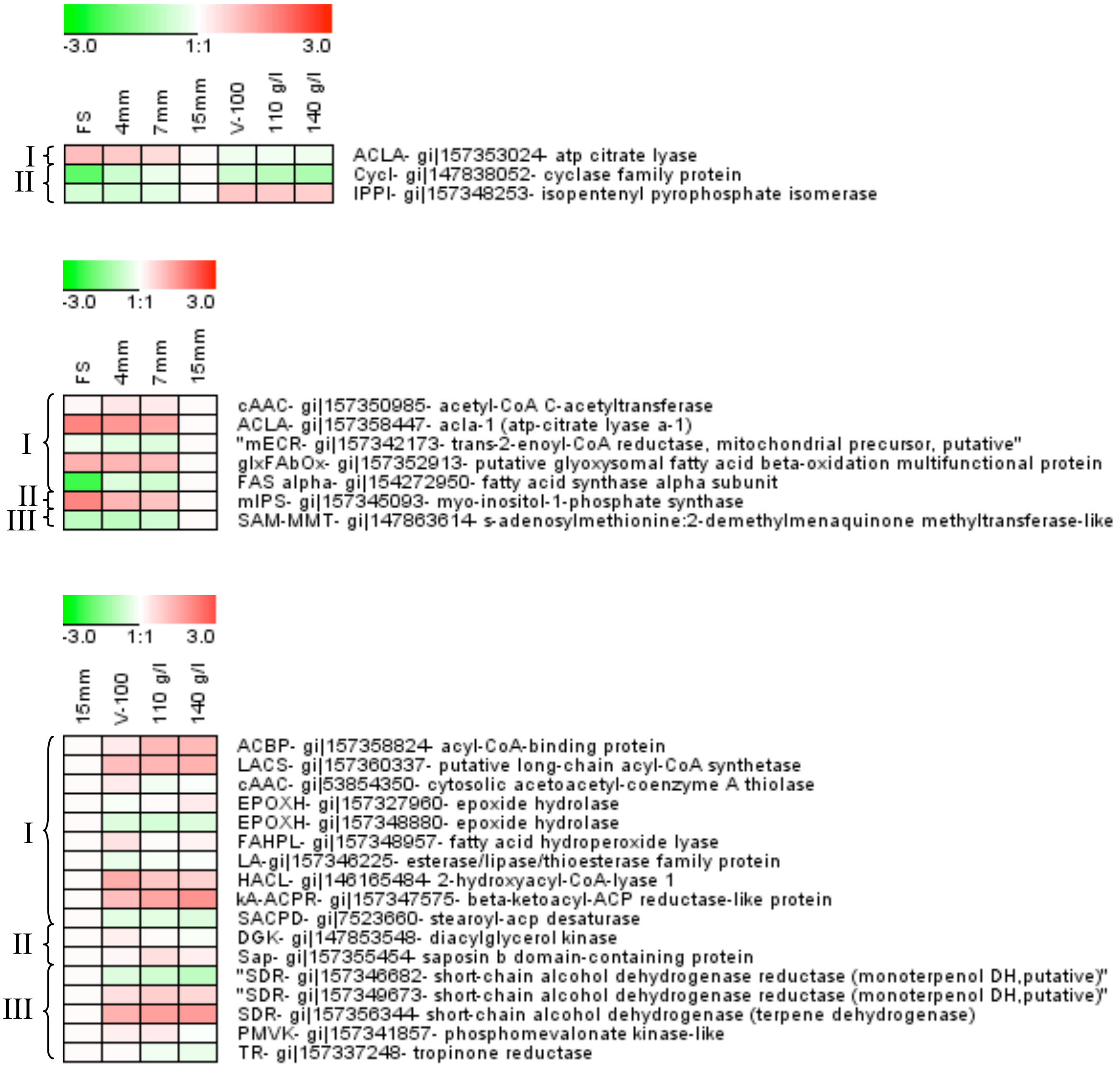

Signaling and Hormone (Additional file 9G)

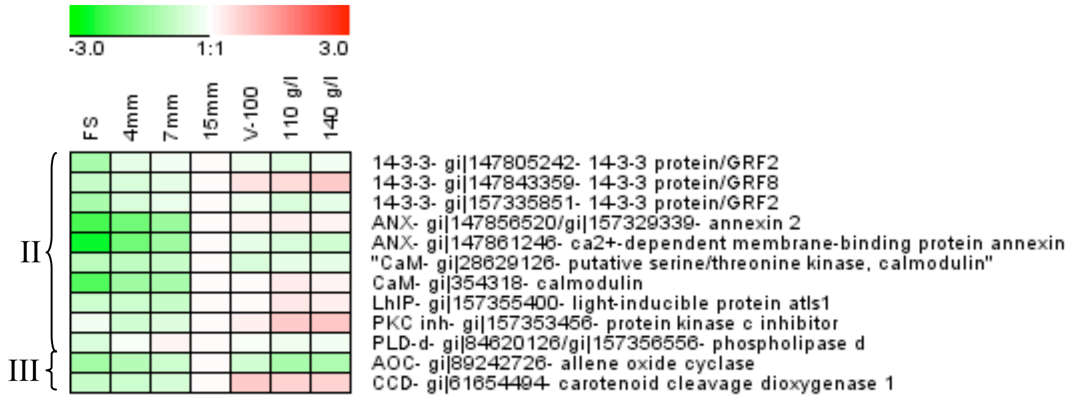

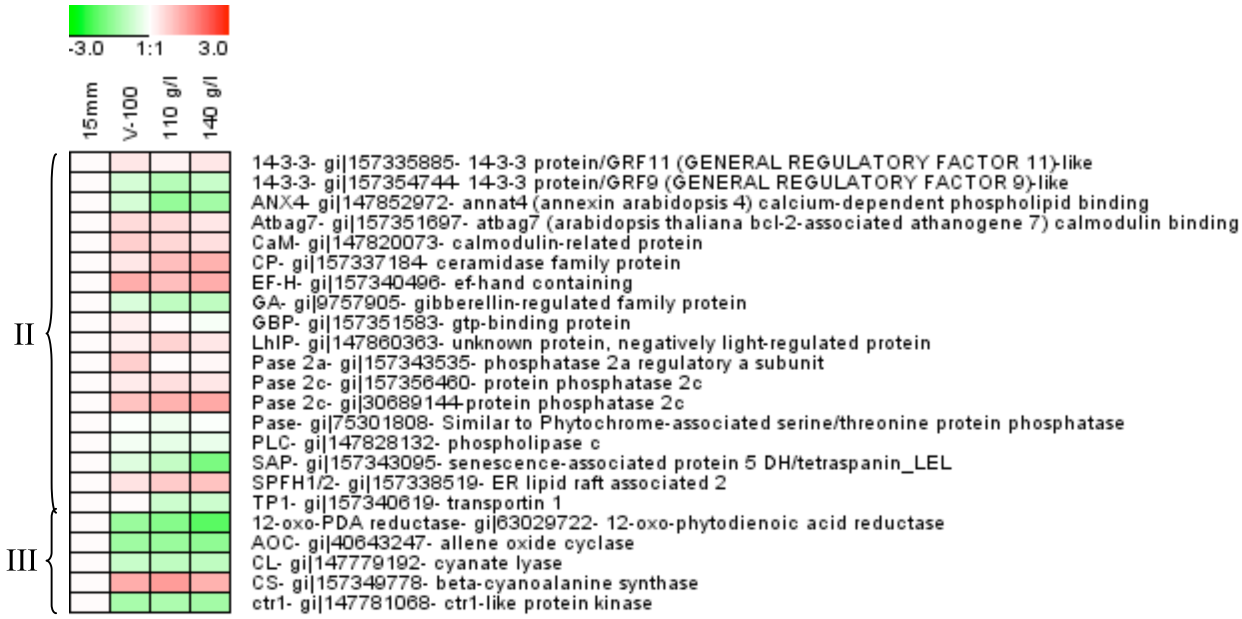

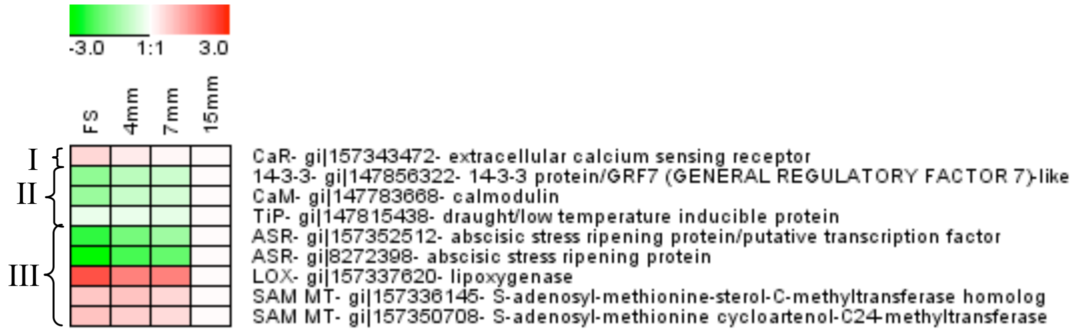

Stress (Additional file 9H)

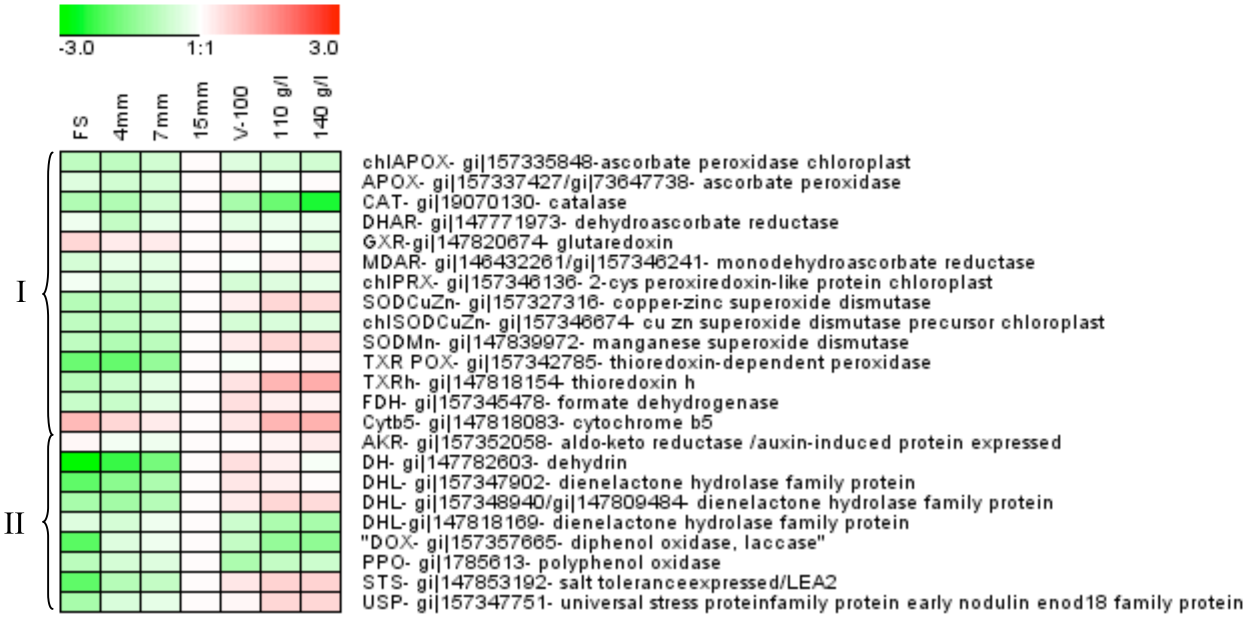

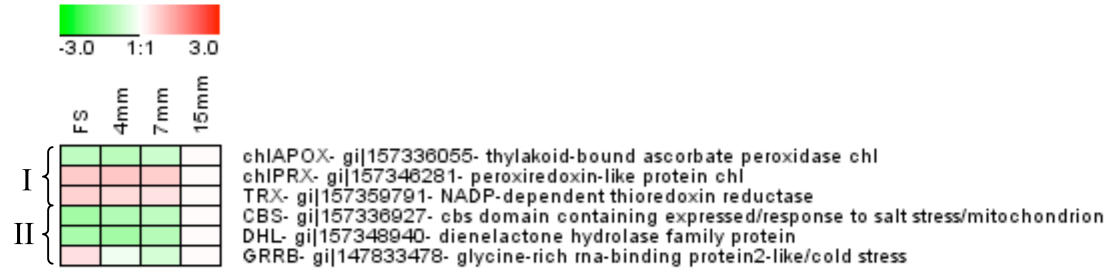

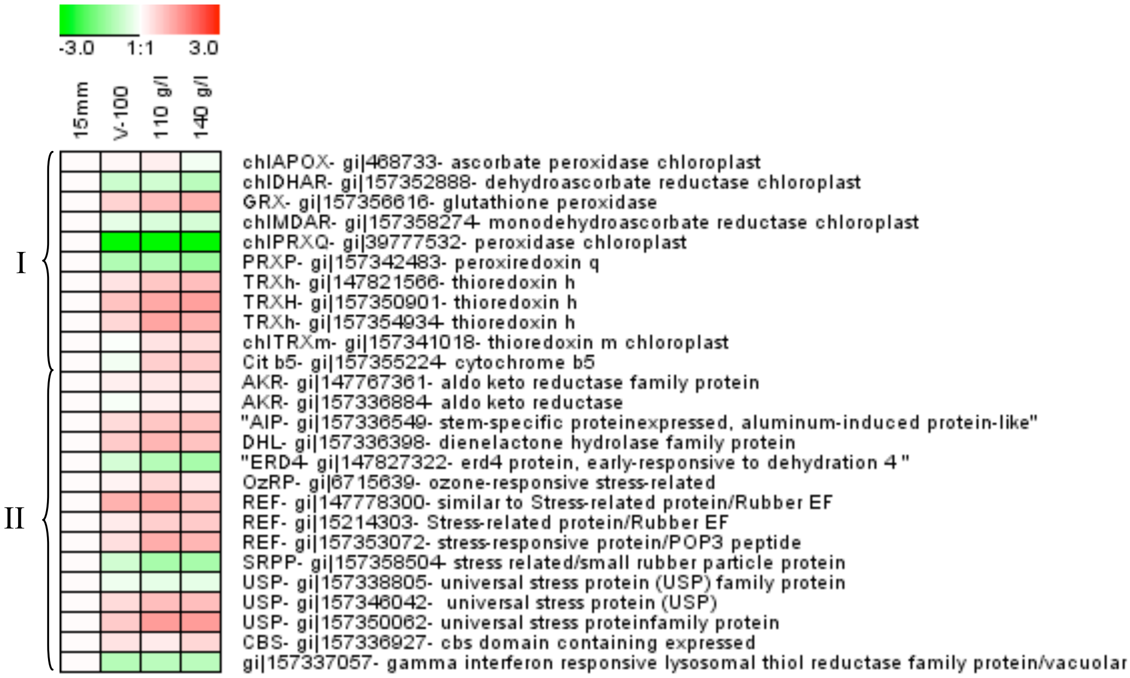

Protein synthesis (Additional file 9I)

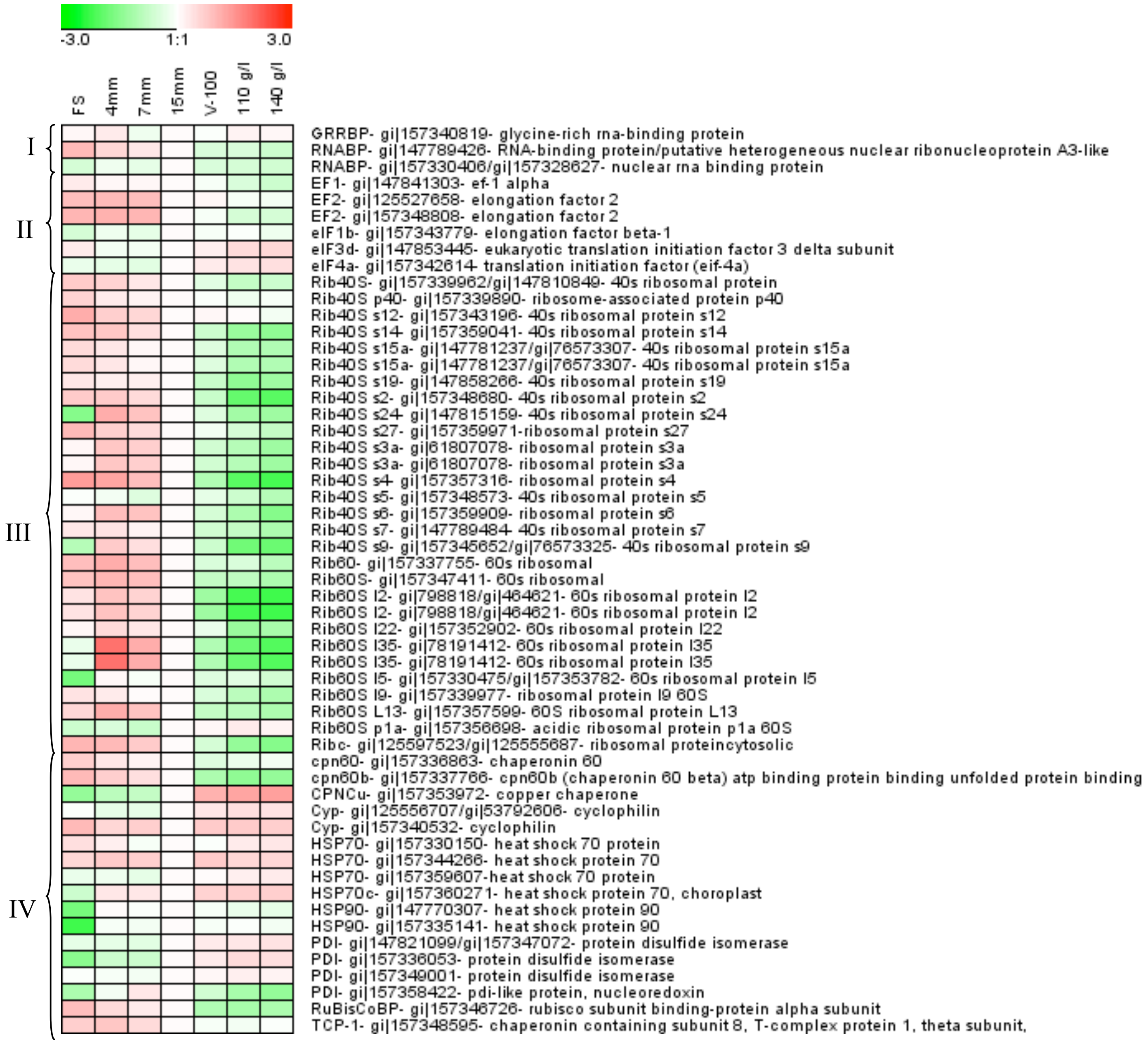

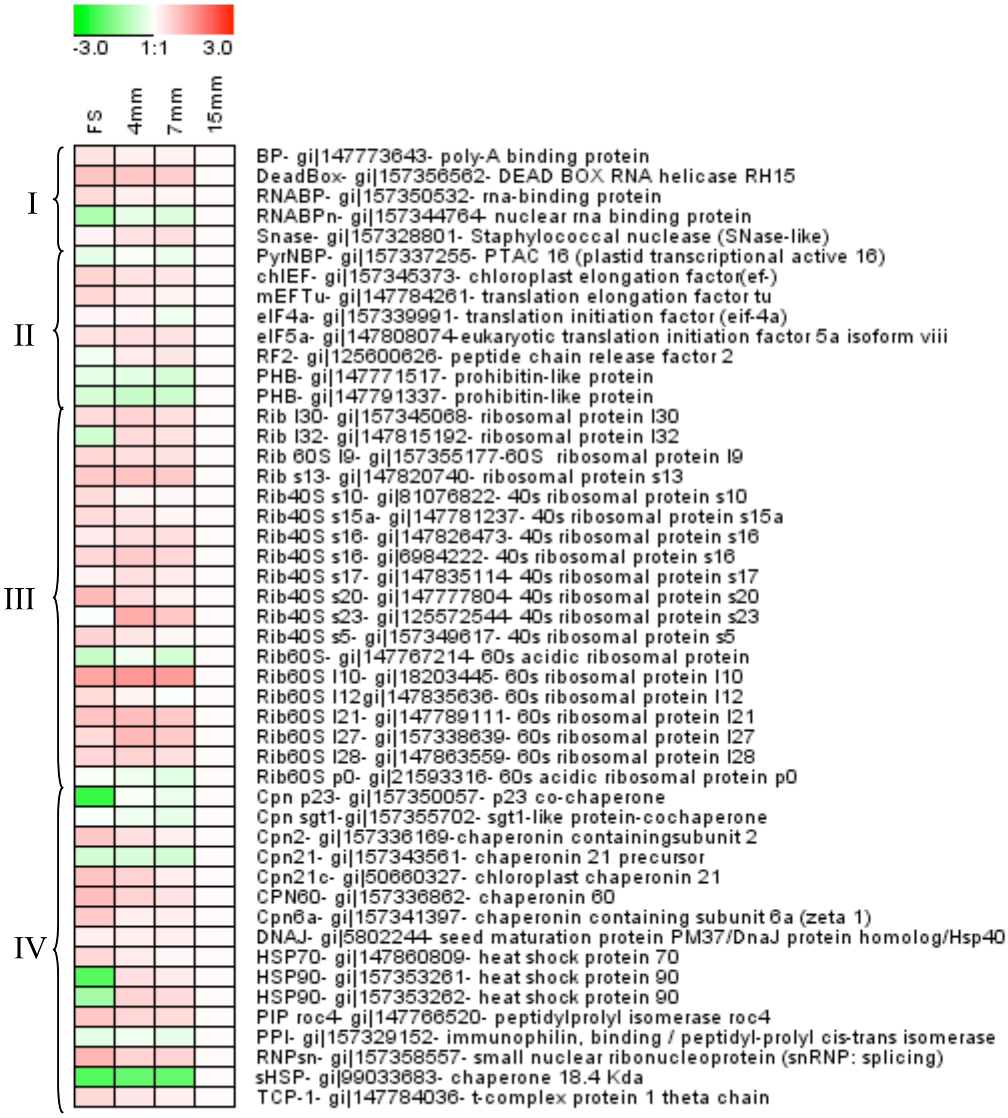
-3.0
1:1
3.0
FS
4mm
7mm
15mm
I
II
III
IV
BP- gi|147773643- poly-A binding protein
DeadBox- gi|157356562- DEAD BOX RNA helicase RH15
RNABP- gi|157350532- rna-binding protein
RNABPn- gi|157344764- nuclear rna binding protein
Snase- gi|157328801- Staphylococcal nuclease (SNase-like)
PyrNBP- gi|157337255- PTAC 16 (plastid transcriptional active 16)
chlEF- gi|157345373- chloroplast elongation factor(ef-)
mEFTu- gi|147784261- translation elongation factor tu
eIF4a- gi|157339991- translation initiation factor (eif-4a)
eIF5a- gi|147808074-eukaryotic translation initiation factor 5a isoform viii
RF2- gi|125600626- peptide chain release factor 2
PHB- gi|147771517- prohibitin-like protein
PHB- gi|147791337- prohibitin-like protein
Rib l30- gi|157345068- ribosomal protein l30
Rib l32- gi|147815192- ribosomal protein l32
Rib 60S l9- gi|157355177-60S ribosomal protein l9
Rib s13- gi|147820740- ribosomal protein s13
Rib40S s10- gi|81076822- 40s ribosomal protein s10
Rib40S s15a- gi|147781237- 40s ribosomal protein s15a
Rib40S s16- gi|147826473- 40s ribosomal protein s16
Rib40S s16- gi|6984222- 40s ribosomal protein s16
Rib40S s17- gi|147835114- 40s ribosomal protein s17
Rib40S s20- gi|147777804- 40s ribosomal protein s20
Rib40S s23- gi|125572544- 40s ribosomal protein s23
Rib40S s5- gi|157349617- 40s ribosomal protein s5
Rib60S- gi|147767214- 60s acidic ribosomal protein
Rib60S l10- gi|18203445- 60s ribosomal protein l10
Rib60S l12gi|147835636- 60s ribosomal protein l12
Rib60S l21- gi|147789111- 60s ribosomal protein l21
Rib60S l27- gi|157338639- 60s ribosomal protein l27
Rib60S l28- gi|147863559- 60s ribosomal protein l28
Rib60S p0- gi|21593316- 60s acidic ribosomal protein p0
Cpn p23- gi|157350057- p23 co-chaperone
Cpn sgt1-gi|157355702- sgt1-like protein-cochaperone
Cpn2- gi|157336169-chaperonin containingsubunit 2
Cpn21- gi|157343561- chaperonin 21 precursor
Cpn21c- gi|50660327- chloroplast chaperonin 21
CPN60- gi|157336862- chaperonin 60
Cpn6a- gi|157341397- chaperonin containing subunit 6a (zeta 1)
DNAJ- gi|5802244- seed maturation protein PM37/DnaJ protein homolog/Hsp40
HSP70- gi|147860809- heat shock protein 70
HSP90- gi|157353261- heat shock protein 90
HSP90- gi|157353262- heat shock protein 90
PIP roc4- gi|147766520- peptidylprolyl isomerase roc4
PPI- gi|157329152- immunophilin, binding / peptidyl-prolyl cis-trans isomerase
RNPsn- gi|157358557- small nuclear ribonucleoprotein (snRNP: splicing)
sHSP- gi|99033683- chaperone 18.4 Kda
TCP-1- gi|147784036- t-complex protein 1 theta chain

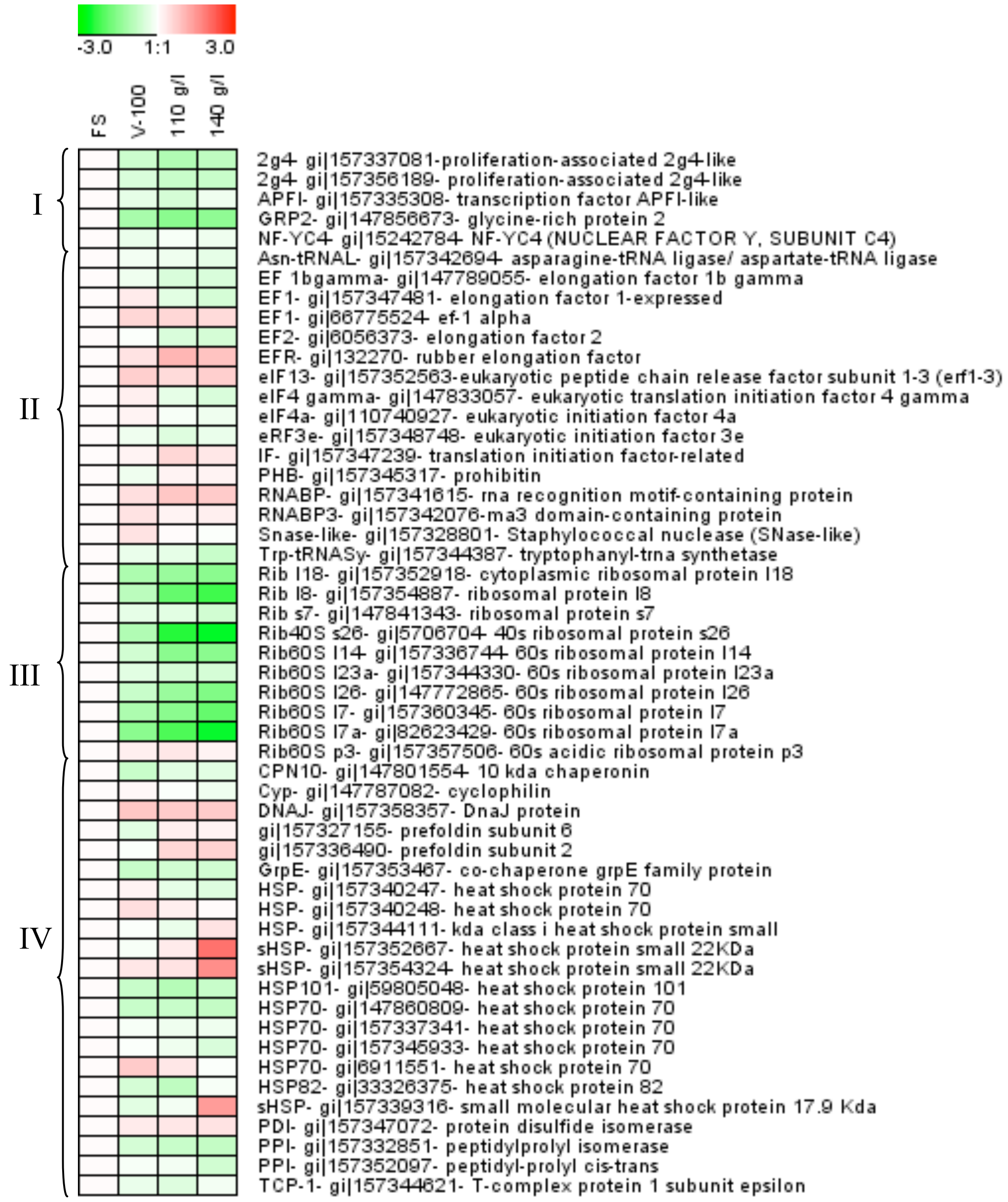
-3.0
1:1
3.0
FS
V-100
110 g/l
140 g/l
I
II
III
IV
2g4- gi|157337081-proliferation-associated 2g4-like
2g4- gi|157356189- proliferation-associated 2g4-like
APFI- gi|157335308- transcription factor APFI-like
GRP2- gi|147856673- glycine-rich protein 2
NF-YC4- gi|15242784- NF-YC4 (NUCLEAR FACTOR Y, SUBUNIT C4)
Asn-tRNAL- gi|157342694- asparagine-tRNA ligase/ aspartate-tRNA ligase
EF 1bgamma- gi|147789055- elongation factor 1b gamma
EF1- gi|157347481- elongation factor 1-expressed
EF1- gi|66775524- ef-1 alpha
EF2- gi|6056373- elongation factor 2
EFR- gi|132270- rubber elongation factor
eIF13- gi|157352563-eukaryotic peptide chain release factor subunit 1-3 (erf1-3)
eIF4 gamma- gi|147833057- eukaryotic translation initiation factor 4 gamma
eIF4a- gi|110740927- eukaryotic initiation factor 4a
eRF3e- gi|157348748- eukaryotic initiation factor 3e
IF- gi|157347239- translation initiation factor-related
PHB- gi|157345317- prohibitin
RNABP- gi|157341615- rna recognition motif-containing protein
RNABP3- gi|157342076-ma3 domain-containing protein
Snase-like- gi|157328801- Staphylococcal nuclease (SNase-like)
Trp-tRNASy- gi|157344387- tryptophanyl-trna synthetase
Rib l18- gi|157352918- cytoplasmic ribosomal protein l18
Rib l8- gi|157354887- ribosomal protein l8
Rib s7- gi|147841343- ribosomal protein s7
Rib40S s26- gi|5706704- 40s ribosomal protein s26
Rib60S l14- gi|157336744- 60s ribosomal protein l14
Rib60S l23a- gi|157344330- 60s ribosomal protein l23a
Rib60S l26- gi|147772865- 60s ribosomal protein l26
Rib60S l7- gi|157360345- 60s ribosomal protein l7
Rib60S l7a- gi|82623429- 60s ribosomal protein l7a
Rib60S p3- gi|157357506- 60s acidic ribosomal protein p3
CPN10- gi|147801554- 10 kda chaperonin
Cyp- gi|147787082- cyclophilin
DNAJ- gi|157358357- DnaJ protein
gi|157327155- prefoldin subunit 6
gi|157336490- prefoldin subunit 2
GrpE- gi|157353467- co-chaperone grpE family protein
HSP- gi|157340247- heat shock protein 70
HSP- gi|157340248- heat shock protein 70
HSP- gi|157344111- kda class i heat shock protein small
sHSP- gi|157352667- heat shock protein small 22KDa
sHSP- gi|157354324- heat shock protein small 22KDa
HSP101- gi|59805048- heat shock protein 101
HSP70- gi|147860809- heat shock protein 70
HSP70- gi|157337341- heat shock protein 70
HSP70- gi|157345933- heat shock protein 70
HSP70- gi|6911551- heat shock protein 70
HSP82- gi|33326375- heat shock protein 82
sHSP- gi|157339316- small molecular heat shock protein 17.9 Kda
PDI- gi|157347072- protein disulfide isomerase
PPI- gi|157332851- peptidylprolyl isomerase
PPI- gi|157352097- peptidyl-prolyl cis-trans
TCP-1- gi|157344621- T-complex protein 1 subunit epsilon

Protein degradation (Additional file 9J)

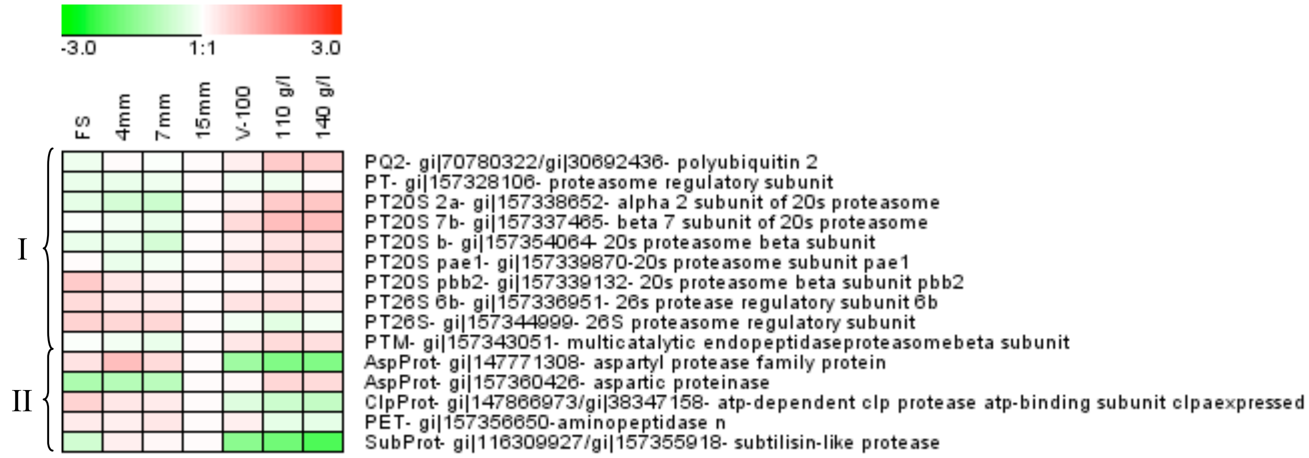

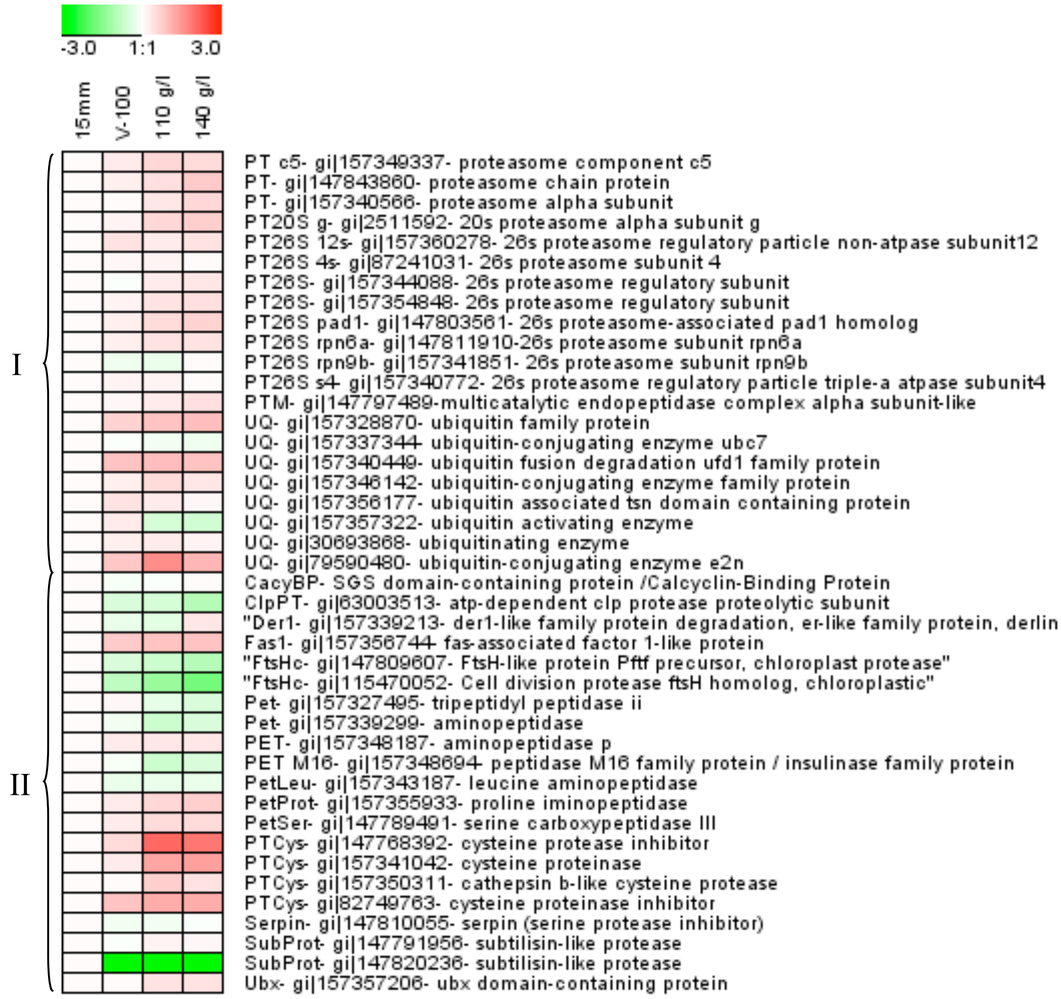

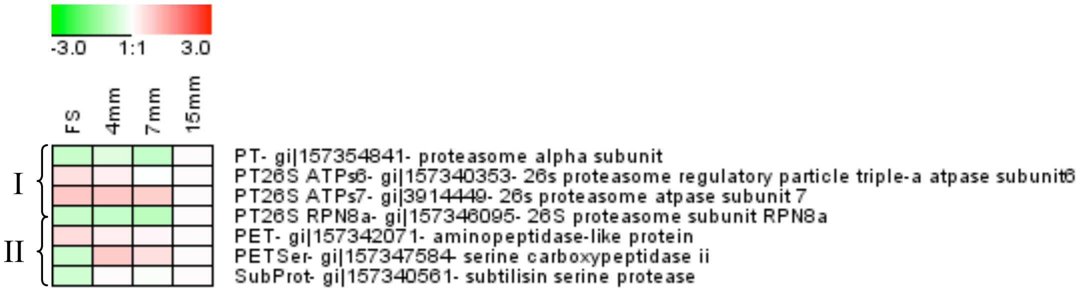
-3.0
1:1
3.0
FS
4mm
7mm
15mm
I
II
PT- gi|157354841- proteasome alpha subunit
PT26S ATPs6- gi|157340353- 26s proteasome regulatory particle triple-a atpase subunit6
PT26S ATPs7- gi|3914449- 26s proteasome atpase subunit 7
PT26S RPN8a- gi|157346095- 26S proteasome subunit RPN8a
PET- gi|157342071- aminopeptidase-like protein
PETSer- gi|157347584- serine carboxypeptidase ii
SubProt- gi|157340561- subtilisin serine protease

Protein processing (Additional file 9K)

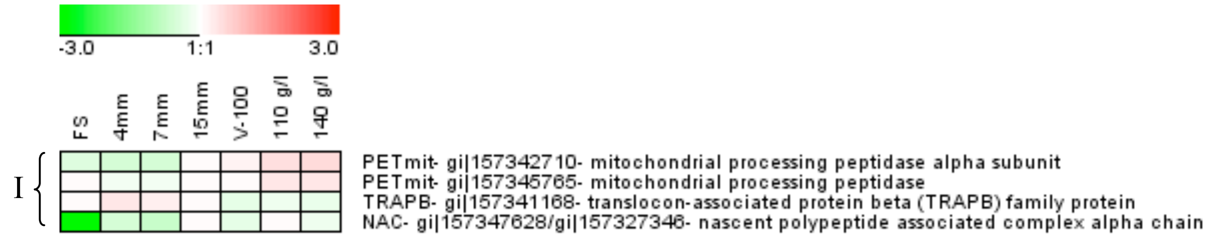

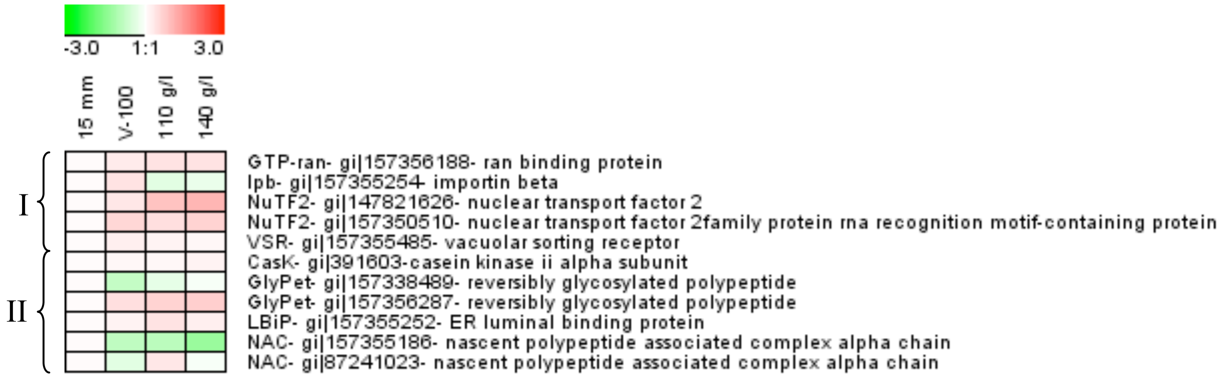

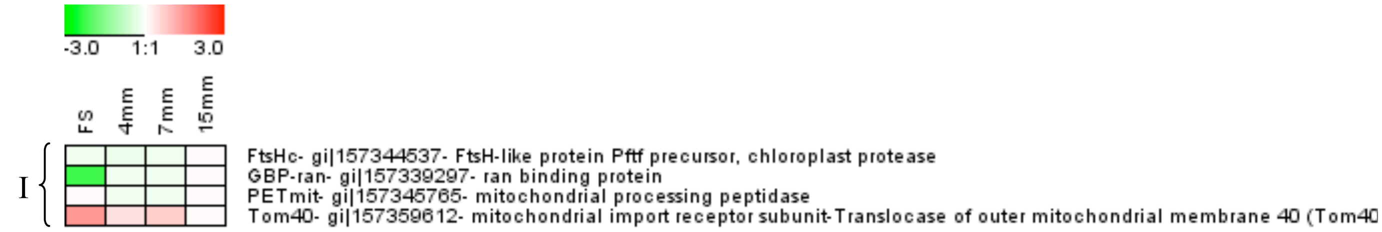

Cell division and growth, biogenesis (Additional file 9L)

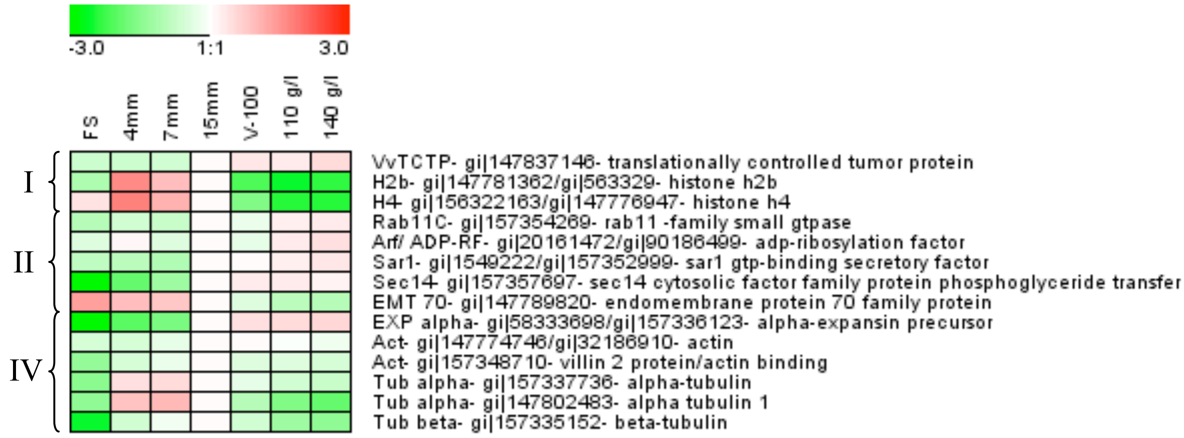

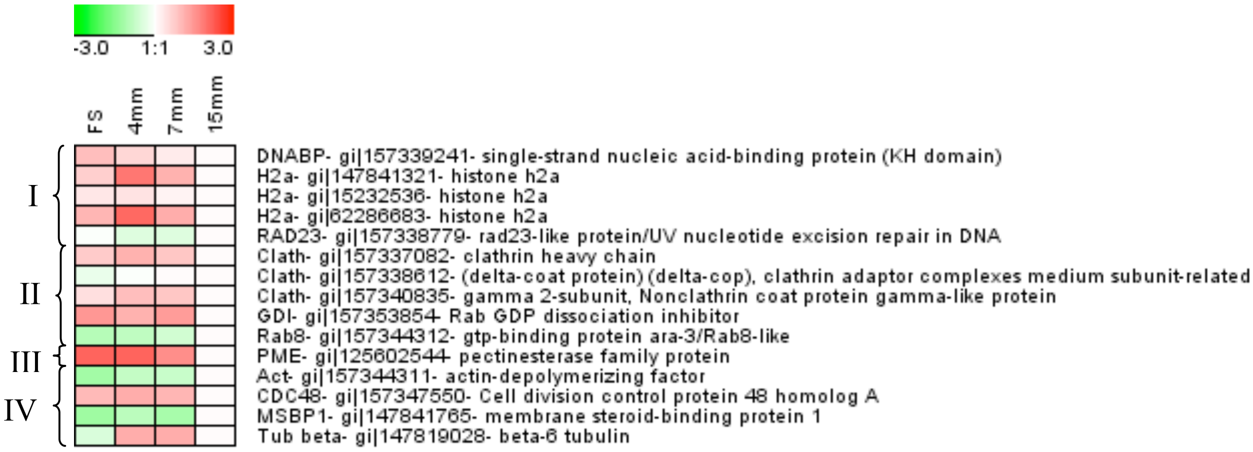

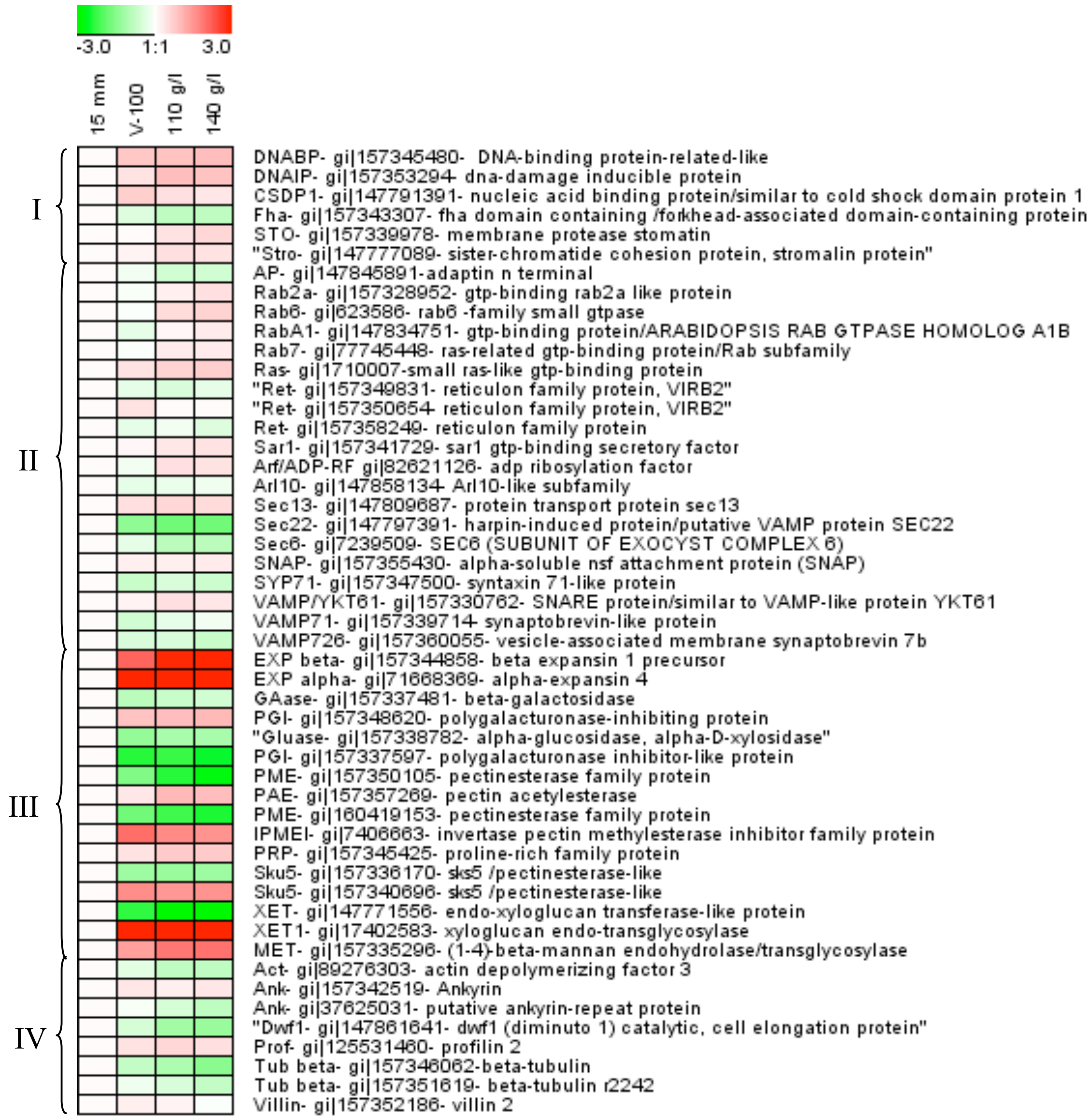
-3.0
1:1
3.0
15 mm
V-100
110 g/l
140 g/l
I
II
III
IV
DNABP- gi|157345480- DNA-binding protein-related-like
DNAIP- gi|157353294- dna-damage inducible protein
CSDP1- gi|147791391- nucleic acid binding protein/similar to cold shock domain protein 1
Fha- gi|157343307- fha domain containing /forkhead-associated domain-containing protein
STO- gi|157339978- membrane protease stomatin
"Stro- gi|147777089- sister-chromatide cohesion protein, stromalin protein"
AP- gi|147845891-adaptin n terminal
Rab2a- gi|157328952- gtp-binding rab2a like protein
Rab6- gi|623586- rab6 -family small gtpase
RabA1- gi|147834751- gtp-binding protein/ARABIDOPSIS RAB GTPASE HOMOLOG A1B
Rab7- gi|77745448- ras-related gtp-binding protein/Rab subfamily
Ras- gi|1710007-small ras-like gtp-binding protein
"Ret- gi|157349831- reticulon family protein, VIRB2"
"Ret- gi|157350654- reticulon family protein, VIRB2"
Ret- gi|157358249- reticulon family protein
Sar1- gi|157341729- sar1 gtp-binding secretory factor
Arf/ADP-RF gi|82621126- adp ribosylation factor
Arl10- gi|147858134- Arl10-like subfamily
Sec13- gi|147809687- protein transport protein sec13
Sec22- gi|147797391- harpin-induced protein/putative VAMP protein SEC22
Sec6- gi|7239509- SEC6 (SUBUNIT OF EXOCYST COMPLEX 6)
SNAP- gi|157355430- alpha-soluble nsf attachment protein (SNAP)
SYP71- gi|157347500- syntaxin 71-like protein
VAMP/YKT61- gi|157330762- SNARE protein/similar to VAMP-like protein YKT61
VAMP71- gi|157339714- synaptobrevin-like protein
VAMP726- gi|157360055- vesicle-associated membrane synaptobrevin 7b
EXP beta- gi|157344858- beta expansin 1 precursor
EXP alpha- gi|71668369- alpha-expansin 4
GAase- gi|157337481- beta-galactosidase
PGI- gi|157348620- polygalacturonase-inhibiting protein
"Gluase- gi|157338782- alpha-glucosidase, alpha-D-xylosidase"
PGI- gi|157337597- polygalacturonase inhibitor-like protein
PME- gi|157350105- pectinesterase family protein
PAE- gi|157357269- pectin acetylesterase
PME- gi|160419153- pectinesterase family protein
IPMEI- gi|7406663- invertase pectin methylesterase inhibitor family protein
PRP- gi|157345425- proline-rich family protein
Sku5- gi|157336170- sks5 /pectinesterase-like
Sku5- gi|157340696- sks5 /pectinesterase-like
XET- gi|147771556- endo-xyloglucan transferase-like protein
XET1- gi|17402583- xyloglucan endo-transglycosylase
MET- gi|157335296- (1-4)-beta-mannan endohydrolase/transglycosylase
Act- gi|89276303- actin depolymerizing factor 3
Ank- gi|157342519- Ankyrin
Ank- gi|37625031- putative ankyrin-repeat protein
"Dwf1- gi|147861641- dwf1 (diminuto 1) catalytic, cell elongation protein"
Prof- gi|125531460- profilin 2
Tub beta- gi|157346062-beta-tubulin
Tub beta- gi|157351619- beta-tubulin r2242
Villin- gi|157352186- villin 2

Defence protein (Additional file 9M)

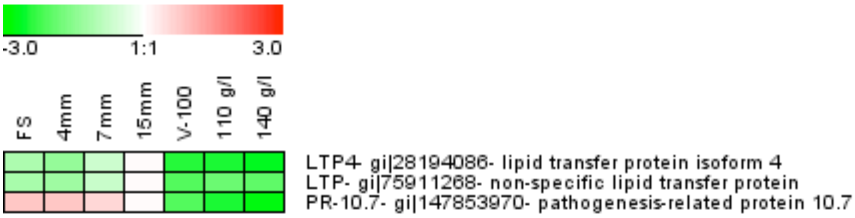

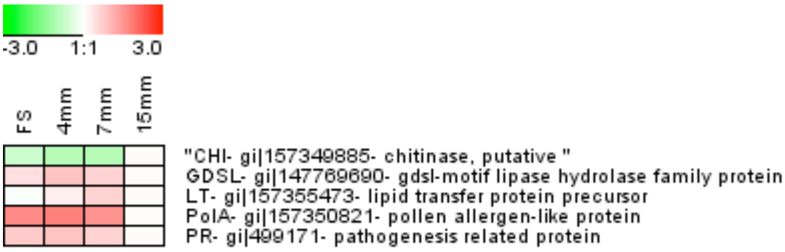

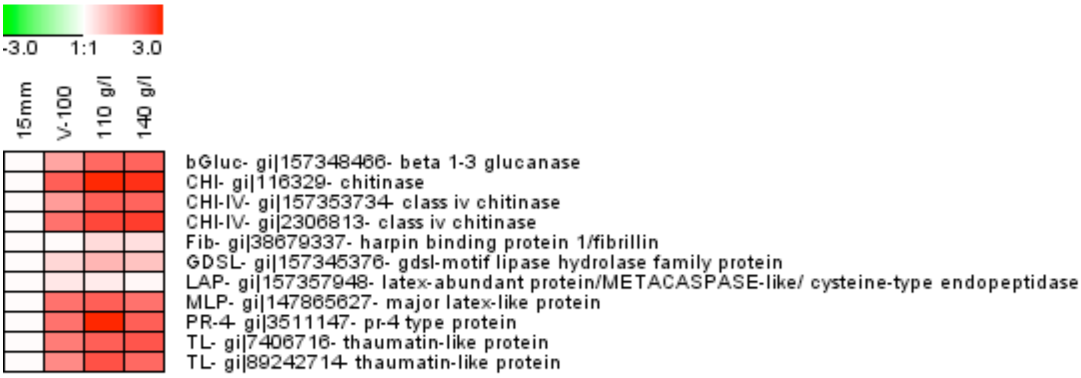

Other proteins of interest (Additional file 9N)

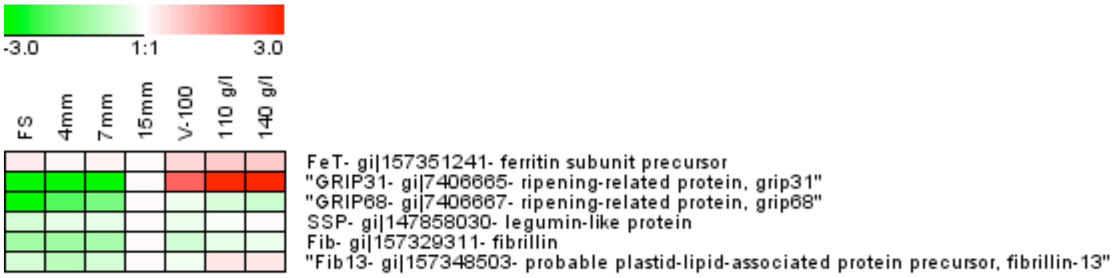

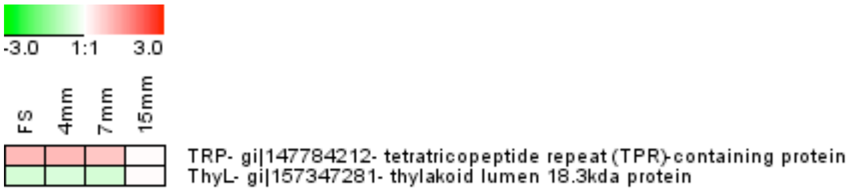

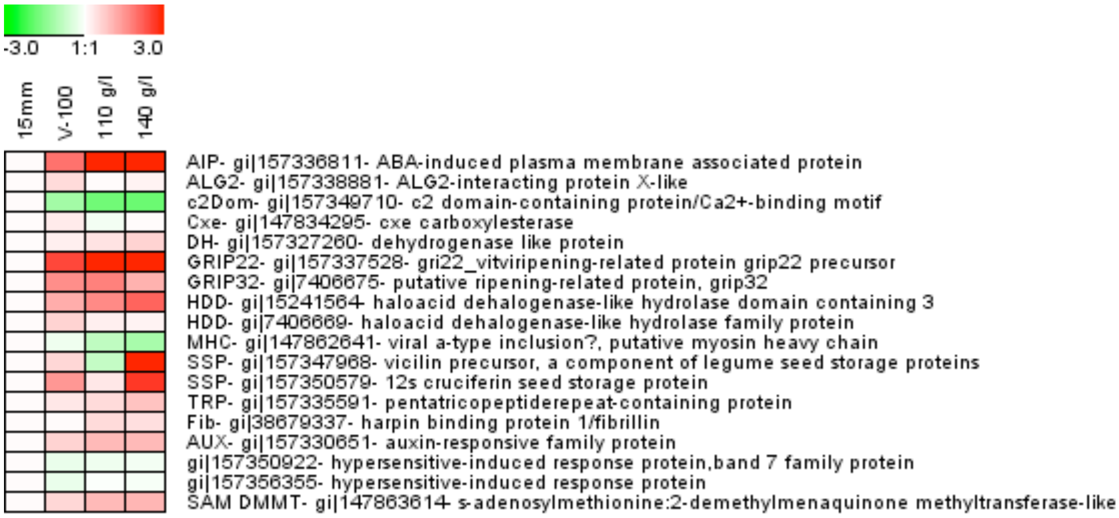

Unknown proteins (Additional file 9O)

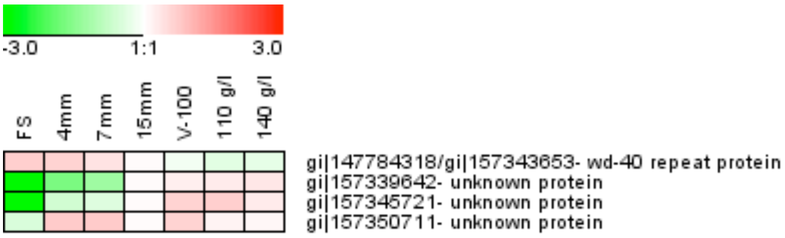

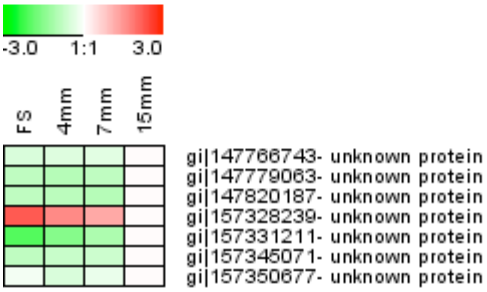

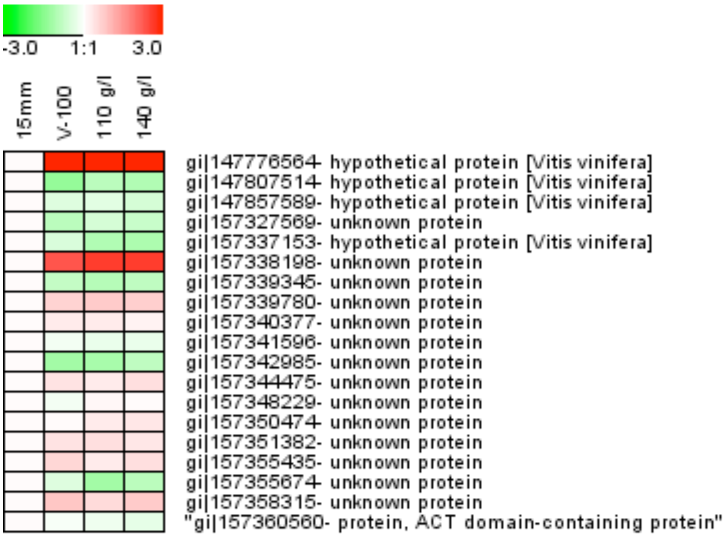

Supplement: Additional file 9 — Profiles of protein functional clusters during berry development [103]. [file 1471-2229-13-167-S9.pdf]
