# Supplementary material for: iTRAQ-based protein profiling provides insights into the central metabolism changes driving grape berry development and ripening
Source: BMC Plant Biol. 2013 Oct 24;13:167. doi: 10.1186/1471-2229-13-167 (PMC4016569; doi:10.1186/1471-2229-13-167)
Supplement: Additional file 10 — Methione synthesis and connection with folate metabolism in grape berries during development. [file 1471-2229-13-167-S10.pdf]

Additional Figure 10. Methione synthesis and connection with folate metabolism in grape berries during development. The protein levels of regulated enzymes are shown in colored squares, indicating the change of expression (log_2 ratio) for each developmental stage in relation to the 15mm stage. In sequence order (left to right), stages are displayed from FS, 4 mm, 7 mm, 15 mm, V-100, 110 g/l, and 140 g/l. Different protein isoforms are shown as different rows. The enzymes names for each biochemical step are indicated in red if differentially expressed and identified, or in black if not detected. MetSy, methionine synthase; SAMSy, S-adenosylmethionine synthase; SAHH, S-adenosyl-l-homocysteine hydrolase; SHMT, serine hydroxymethyltransferase; PGDH, 3-phosphoglycerate dehydrogenase; GlyCS, glycine decarboxylase, glycine cleavage system; MTHFR, methylenetetrahydrofolate reductase; MTHFDH, methylenetetrahydrofolate dehydrogenase; SAHN, S-adenosyl homocysteine nucleosidase; MTRK, methylthioribose kinase; ARD, acireductone dioxygenase.

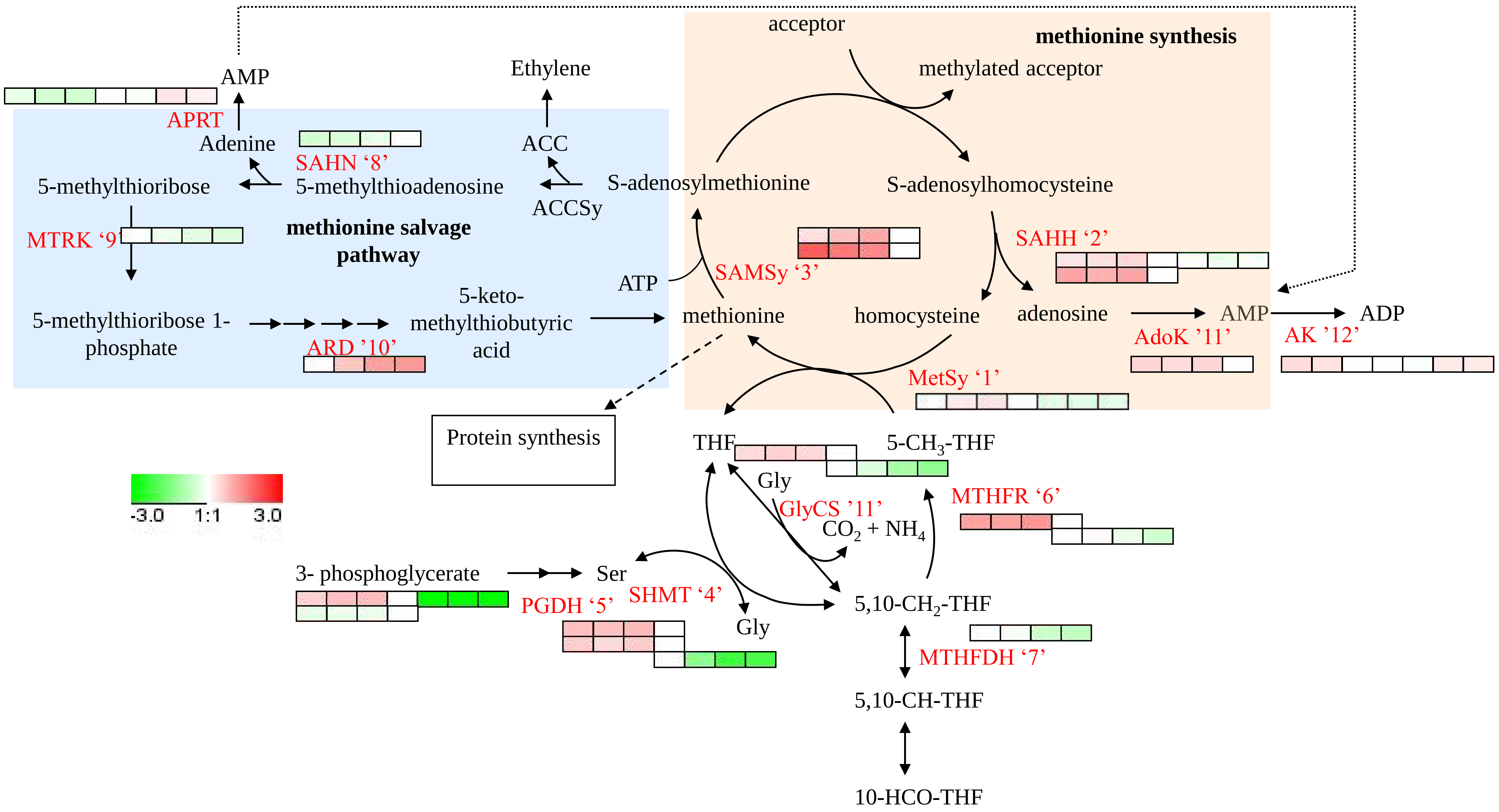

acceptor
methionine synthesis
methylated acceptor
AMP
Ethylene
APRT
Adenine
ACC
SAHN '8'
5-methylthioribose
5-methylthioadenosine
ACCSy
S-adenosylmethionine
S-adenosylhomocysteine
MTRK '9'
methionine salvage pathway
SAHH '2'
SAMSy '3'
ATP
5-keto-methylthiobutyric acid
5-methylthioribose 1-phosphate
methionine
homocysteine
adenosine
AMP
ADP
ARD '10'
AdoK '11'
AK '12'
MetSy '1'
Protein synthesis
THF
5-CH3-THF
Gly
GlyCS '11'
CO2 + NH4
MTHFR '6'
-3.0
1:1
3.0
3- phosphoglycerate
Ser
SHMT '4'
PGDH '5'
Gly
5,10-CH2-THF
MTHFDH '7'
5,10-CH-THF
10-HCO-THF
