# Supplementary material for: iTRAQ-based protein profiling provides insights into the central metabolism changes driving grape berry development and ripening
Source: BMC Plant Biol. 2013 Oct 24;13:167. doi: 10.1186/1471-2229-13-167 (PMC4016569; doi:10.1186/1471-2229-13-167)
Supplement: Additional file 11 — Results and discussion about the functional groups ‘Nitrogen and amino acid metabolism’, ‘Terpenoid metabolism’, ‘Signaling and hormone’, ‘Stress’, ‘Protein synthesis’, ‘Protein degradation’, ‘Protein processing’, ‘Cell division and growth, biogenesis’, ‘Defense proteins’, ‘Other proteins of interest’. [file 1471-2229-13-167-S11.pdf]

Protein Profile Analysis

1. Nitrogen and Amino Acid Metabolism **(**Additional File 9E**)**

In grape berries, ammonium ions can represent up to 80% of total nitrogen before *véraison,* while they can decrease to 5-10% after maturation; instead, amino acids can represent up to 50% of total nitrogen in the musts of ripe berries [1]. Thus, the major form of nitrogen switches from NH_4^+ to amino acids as the berry develops and ripens. Previous DIGE [2] and microarray studies [3] have reported profiles of glutamine synthase (GS) and aspartate aminotransferase (AspAT) during green development that are consistent with high levels of free ammonium and the drop in Gln and Glu observed before *véraison* in grape berries [4]. Here the decreasing profiles of one isoform of the cytosolic GS, the three isoforms of AspAT and the increase in glutamate dehydrogenase (GDH) support and complement the data presented above. In the ripening phase, another isoform of the cytosolic GS and two other isoforms of AspAT were up-regulated in parallel to the down-regulation of GDH. An activity peak for GDH before *véraison* [5] well matched the abundance profile found herein. In addition, a down-regulated ammonium transporter, an up-regulated alanine aminotransferase (AlaAT) and L-asparagine amidohydrolase (AsnAH) were identified. Asn utilization by plants plays an important role in the nitrogen metabolism of developing plant tissues [6] as an innocuous amino acid used to store and transport nitrogen from sources to sinks. Taken together, these profiles are consistent with the incorporation of ammonium into amino acids. Indeed, Ala, Glu and Gln are three of the seven most abundant amino acids in ripe berries [1]. These results confirm the previous study using DIGE [2] and indicate particular isoforms of GS and AspAT that are specific of pre- and post-*véraison* berry development.

Amino acids Met, Ser and Gly are involved in the synthesis of the universal methyl group donor S-adenosylmethionine (SAM). Several enzymes involved in this process have been described in a previous DIGE study [2], including methionine synthase (MetSy, Additional Figure 13 '1'), S-adenosyl-L-homocysteine hydrolase (SAHH, Additional figure 13 '2'), SAM synthase (SAMSy, Additional Figure 13 '3'), serine/glycine hydroxymethyl transferase (SHMT, Additional Figure 13 '4') and phosphoglycerate dehydrogenase (PGDH, Additional Figure 13 '5'). In addition, other enzymes involved in that and in related pathways were detected in this study, and help provide a more complete picture of this process during berry development (see Additional Figure 13). These enzymes are 5,10-methylene tetrahydrofolate reductase (MTHFR, Additional Figure 13 '6'), 5,10-methylene tetrahydrofolate dehydrogenase (MTHFDH, Additional Figure 13 '7'), involved in THF recycling; S-adenosyl-L-homocysteine nucleotidase (SAHN, Additional Figure 13 '8'), as an alternative reaction to SAHH; methyl-thioribose kinase (MTRK, Additional figure 13 '9') and acireductone dioxygenase (ARD, Additional Figure 13 '10'), from the Met salvage pathway; adenosine kinase (AdoK, Additional Figure 13 '11') and adenylate kinase (AK, Additional Figure 13 '12'), involved in recycling the adenosine released by SAHH. The abundance profiles between both experiments were consistent and suggest that the methylating capacity of the pathway is very high during early berry development phases, which likely satisfies the demand of intense processes such as DNA and flavonoid synthesis. From *véraison*, the capacity of SAM synthesis seemed to significantly reduce while the Met salvage pathway emerged. This change suggests that Met might be directed toward protein synthesis, which is known to increase at *véraison* [7]. Nevertheless, the use of the Met salvage pathway, particularly the up-regulation of ARD, linked the use of SAM with ethylene production. In rice, ARD1

has been discovered to be a primary response gene for ethylene, which is induced by low levels of this hormone [8]. The up-regulation of ARD in the mesocarp at *véraison* coincides with the release of a modest ethylene peak in grape berries [9], thus establishing a potential link between ARD and ethylene also in grape.

2. Terpenoid metabolism **(**Additional File 9F**)**

Isopentenyl pyrophosphate isomerase (IPPI) is a key enzyme in the biosynthesis of terpenes isomerizing isopentenyl diphosphate (IPP) into allylic isomer dimethylallyl diphosphate (DMAPP). Both IPP and DMAPP are precursors for the first condensation reaction to form the monoterpene intermediate geranyl diphosphate. Apart from IPPI, three proteins belonging to the short-chain alcohol dehydrogenase/reductase family protein (SDR) have been quantified, and present a poor homology with the well-characterized monoterpene dehydrogenases in *Mentha piperita* (isopiperitenol dehydrogenase, menthol dehydrogenase, neomenthol dehydrogenase) [10, 11]. These proteins quantified here from grape, which remain uncharacterized to date, can perform an analogous function to the homologous monoterpene reductase/dehydrogenases in *M. Piperita.* Thus they are involved in the synthesis of the vitaceae-specific monoterpene species in grape berries. The profile of IPPI and SDRs suggests a more active terpene synthesis from the onset of ripening. These results are in agreement with the metabolic profiles for the terpene and terpene-derived compounds in *V. vinifera* cv. Muscat Hamburg [12], which generally increased during grape development; in particular, the monoterpene fraction increased from the onset of ripening, from which linalool became the most accumulated monoterpene at the end of ripening. To a greater extent, the aroma of the Muscat flavor is due to the contribution of monoterpenes; thus it is interesting to consider the three uncharacterized SDRs identified herein, considered to be potential protein

markers, for a functional study to clarify the possible monoterpenes species that reach fruition.

3. Signaling and Hormone (*Additional File 9G****)***

The abundance and presence of certain hormones along development and ripening of grapes indicates a role of that hormone at that point of development. Although auxins, cytokinins and gibberellins (GAs) are known to control early berry development, no protein that would be directly related with the accumulation of these hormones was found. Only a GA-regulated family protein of unknown function dropped its levels from the onset of ripening, which agrees with the levels of GAs in berries established along development [13-15]. In turn, auxin-related proteins were found in grape berry skin during ripening with a peak of abundance at *véraison* [16].

On the other hand, berry maturation is thought to be under the control of ethylene, abscisic acid (ABA) and brassinosteroids (BRs). Some studies suggest that ethylene has a critical role in the ripening of the grape berries [9], in spite of their classification as a non-climacteric fruit [17], and is required for increasing diameter, decreasing acidity and enhancing anthocyanin accumulation in grape berries [9, 18]. In this study, changes in proteins directly related with the synthesis of ethylene (1-aminocyclopropane-1-carboxylate (ACC) synthase (ACCSy), ACC oxidase (ACCO)) were not detected and the profiles of enzymes involved in the metabolism of SAM (MetSy, SAHH, SAMSy) (see Additional File 9E), the precursor for ethylene formation, do not support a synthesis of ethylene neither in pericarp of green berries nor mesocarp along ripening. This would be consistent with the observation that ethylene synthesis may be skin-related [19]. However, enzymes indirectly involved in ethylene production such as β-cyanolalanine synthase (CAS), which is implicated in detoxification of the cyanide by-product of the conversion of ACC to ethylene [20],

and acireductone dioxigenase (ARD), from the methionine salvage pathway (Additional file 9E), were found up-regulated along ripening in mesocarp, suggesting that there may be a sustained accumulation of ethylene along ripening in this tissue. Previous studies at gene transcript and protein level [3, 21, 22] found that the expression of ACCO and ACCSy is elevated around *véraison*. Also, ctr1-like protein kinase, a protein involved in transduction of ethylene signal as a negative regulator of the ethylene response [23], has been identified. Down-regulation of ctr1 protein from onset of ripening indicates that from V-100 stage grape berries may become more sensitive to ethylene. This result supports the above proteomic data obtained, which point outs to a putative prolonged release of ethylene during ripening.

While there is still some debate regarding ethylene levels and accumulation in grape berries and its role on ripening, abscisic acid (ABA) accumulation was clearly established [14, 24-29]. The accumulation pattern of ABA, with an increase prior *véraison* to reach a peak two or three weeks later and declining as fruit reach ripeness, clearly points to its involvement in ripening [13, 30-33]. In correlation with the profile for ABA accumulation, a carotenoid cleavage dioxigenase has been quantified along development, which also ties in with reported data at gene transcript level [3]. The effect of ABA is involved with the timing of ripening for the increase of sugar levels and accumulation of anthocyanin [25, 34]. Two abscisic acid, stress and ripening (ASR) proteins (gi|157352512 and gi|8272398) are strongly up-regulated, with 5.3- and 9-fold change respectively, before *véraison*. The former is a putative transcription factor homologous to the ASR-like gene VvMSA having a 96% sequence similarity. VvMSA was shown to act as part of a transcription-regulating complex involved in sugar and ABA signaling [35]. Profiles found here are in agreement with the MSA,

the ASR protein identified in a previous proteomic study along development of grape berry [2].

Recently discovered, brassinosteroids (BRs) have been found to participate in a range of processes including the control of growth and response to stress. In grapevine, BRs have been shown to have roles in growth promotion, stress response and the control of berry ripening [15]. It seems that both processes could be important in berry ripening as is expanding and accumulating large amounts of osmotically active sugars during this time, which could induce a response to stress [36]. DREPP, a protein tightly related with the role of BRs, was a novel grape protein identified here in the developing and ripening berry. DREPP shows an up-regulation towards *véraison* up to 12.6-fold and then levelling off until full ripening (Additional File 9A). This protein was first reported in tobacco plasma membrane [37] and later demonstrated that it is a BR-inducible protein in Arabidopsis, having an important role in BR promotion of cell growth [38]. Thus, this is an interesting candidate protein to monitor as a potential developmental marker and worth to be further analyzed for its probable role in ripening in response to BR signaling.

In regards to the biosynthesis of BR, no proteins directly implicated were identified; only two proteins involved in the synthesis of the steroid cycloartenol (SAM MT) were identified. Their levels were stable along early stages of development and dropped before *véraison*. All sterols in plants are formed from the metabolite cycloartenol derived from squalene. The plausible precursors of various BRs derive from cycloartenol; thus, it seems that BRs precursors are at least produced in early stages of development. In fact, the levels of castasterone (CS) and its precursor 6-deoxoCS are high at early development of grapes and decline until *véraison* to

increase sharply again at this point [15]. Lücker *et al.* [39] detected two proteins peaking at *véraison* involved in BR biosynthetic pathway via squalene biosynthesis.

Jasmonic acid (JA) is another plant regulator whose endogenous levels stimulate cell division at early stages of development in climacteric apples and in non-climateric sweet cherries [23]. A lipoxygenase (LOX), detected with a strong decreased from 7mm-to-15mm here, shows the same profile as described in the earlier DIGE proteomic study [2]. LOX converts linoleic acid into hydroperoxylinoleic acid, which may derive to different end products including JA, but also volatile alcohols and aldehydes as potentially occurring in the skin of the grape berries at *véraison* [16]. Evidence that LOX can be involved in JA formation along green stages were found, while enzymes acting downstream in the octadecanoic pathway were detected; an allene oxide cyclase (AOC) peaking at *véraison*, and a 12-oxo-phytodienoic acid reductase (12-oxo-PDA reductase) with decreasing levels along ripening. Also 12-oxo-PDA reductase transcripts, besides LOX transcripts, had been described during early berry development [3]. Although the profiles of LOX and the JA-downstream enzymes are not synchronized, JA synthesis may occur at early development declining until *véraison*, which coincides with the levels for JA reported [40].

A considerable set of protein involved in signaling pathways were identified and quantified including Ca^{2+}-dependent and Ca^{2+}-independent pathways. Two of the principal elements in the signal transduction pathways are intracellular Ca^{2+} and protein kinases constituting a network of great complexity. Calmodulins, which are primary Ca^{2+} receptors that act as Ca^{2+}-calmodulin complexes that can activate many other enzymes, increase up to 4-fold at the end of green development stages. Such increase probably relates a more sensitive state of cells to small changes in the intracellular concentration of Ca^{2+} at that point of development. In the same way,

several Ca^{2+}-binding proteins have similar profiles. Another type of signaling proteins is the so called 14-3-3 proteins. These facilitate phosphorylation of other molecules acting as an intracellular messenger that enables cross-talking of signal transduction chains. All 14-3-3 protein isoforms identified here present a coordinate profile with previous described proteins showing an up-regulation towards *véraison*. Interestingly, these profiles correlate well with the identified ASR proteins (see discussion above). ABA transduction involves Ca^{2+}-dependent pathways and protein kinases as well as several 14-3-3 members [41, 42]. In fact, two annexins, a class of Ca^{2+}- and phospholipid-binding proteins, ANX1 and ANX4, have been identified as components in osmotic stress and abscisic acid signaling in Arabidopsis [43]. Actually, similar results were obtained at transcript level; a large set of genes were deregulated related to Ca^{2+} sequestration, transport and signaling, specifically around *véraison* [3].

Phospholipases can be activated in the plasma membrane by G-protein-coupled receptors. PLC participates with several kinases and phosphatases in an important cycle of inositol phospholipids synthesis and breakdown. PLD that hydrolyzes phosphatidylcholine to release phosphatidic acid has been implicated in plants in processes as fruit ripening, senescence, stress and wounding [44]. Both activation mechanisms by PLD and PLC are interrelated by a complex signaling network. Both proteins identified with modest deregulated levels of protein in grape development have a similar quantitation pattern.

4. Stress **(**Additional file 9H**)**

Proteins involved in oxidative stress present a general profile increasing along the first growth phase with a maximum from 7mm-to-15mm stage and then decreasing during the ripening phase, which suggests a higher expression of ROS-detoxifying

enzymes in green developing tissues, in agreement with previous proteomic studies [2, 16, 21]. The antioxidant system for detoxification of ROS species includes catalase (CAT), superoxide dismutase (SOD), peroxidases (POX) and the enzymes involved in the ascorbate-glutathione cycle: ascorbate peroxidase (APOX), monodehydroascorbate reductase (MDHAR), dehydroascorbate reductase (DHAR) and glutathione reductase (GRX), which scavenge H_2O_2 and the superoxide anion and regenerates ascorbate and glutathione [45, 46]. The profiles for all of them, including the chloroplastic Cu/Zn-superoxide dismutase (SOD Cu/Zn), APOX and the thylakoid-bound APOX and two peroxiredoxins (PRX), suggest its involvement in the removal of H_2O_2 during and especially at the end of the green development of the berry. Pilati *et al.*, [22] reported an oxidative burst starting from *véraison* characterized by a rapid accumulation of H_2O_2, which modulates the expression of a set of oxidative stress responsive genes. However, the source of such H_2O_2 remains unknown. The accumulation of OEE proteins towards pre-*véraison* as noticed above (see group 'Photosynthesis') was suggested to potentially play a role in H_2O_2 production at the chloroplast level by redirecting the unused electrons towards O_2 [2, 47]. Two late abundant embryogenesis proteins (LEA) have been identified. A dehydrin (DH) protein is strongly up-regulated along the first growth period with up to 20-fold change 7mm-to-15mm, and then levels remain unaltered along ripening. Dehydrins, also called LEA D11-family group II or RAB (responsive to ABA), accumulate in plant tissues in response to cellular dehydration resulting from developmental events or environmental stimuli such as osmotic stress and low temperature but also abscisic acid treatment [48-52]. Dehydrin profile agrees with the profile detected for the protein ASR, which is known to respond to ABA [35]. These findings suggest that dehydrin could be involved in the adaptation of the fruit to the

drastic changes that occur at *véraison* such as accumulation of sugars and water; thus, dehydrin could be acting as an osmoprotectant as these proteins have been suggested to play a biochemical role in the stabilization of macromolecules within cells [49]. The second LEA protein is a salt tolerance expressed protein (STS) belonging to LEA Group II, which has the same profile as dehydrin, increasing along the first growth period with up to 4-fold change from 7mm-to-15mm stages but its precise function is unknown.

Another protein probably involved in adaptation to osmotic stress is ERD4, which decreases from *véraison* along the ripening phase. An ERD4 protein from *Zea mays* has been suggested to play an important role in early stages of plant adaptation to stress conditions [53].

Several proteins homologous to a universal stress protein (USP) have been identified showing an increase in abundance throughout the berry development, in agreement with a previous proteomic study [2]. Members of this family may have specific functions although not yet characterized. USP homologues appear to be ubiquitous proteins in plants involved in stress response [54]. Up-regulation of a USP gene in *Gossypium arboretum* is produced by effect of the growth hormones GA and ABA and abiotic stresses as the water status [55]. Thus, profile of USP protein in grape berry is consistent with the ABA accumulation [56].

A polyphenol oxidase (PPO) was detected along development of grape berries with an increasing profile along the first growth period (1.92-fold from 7mm-to-15mm) to then decreases from *véraison* in mesocarp. The same trend was reported previously for mesocarp [2] and for skin [57] of ripe berries. The deregulated PPO detected here in green and ripe stages is identified with 25 and 17 different peptides covering 47% and 30% along the whole protein sequence. In these previous reports, although PPO is

a 60 kDa polypeptide, it was detected as the 16 kDa C-terminal domain fragment. In the present bottom-up approach study, it can not be ascertained if the protein is intact or fragmented in its N- and C-terminal portions. Nevertheless, along the green phase 4 out of the 6 most abundant unique peptides (over 15 spectra) belong to the C-terminal portion, thus contributing enormously to the quantitation of this protein. These data suggest that the C-terminal fragment may be particularly accumulated along this part of the development. PPO potentially regulates photosynthesis [58, 59] through its involvement in the Mehler reaction, photoreduction of oxygen by photosystem I, and metabolism of reactive oxygen species in the chloroplast [60]. This hypothesis supports the trend of other enzymes that seem to act in the photoprotection and stability of photosystems I and II (see group 'Photosynthesis').

5. Protein Synthesis (Additional file 9I)

A high number of proteins directly involved in protein synthesis such as several isoforms of the structural components of the ribosomal 40S and 60S subunits were identified and quantified. All of them present a common trend along development showing a biphasic decrease with *véraison* as the inflection point. Previous reports demonstrate the same two-phase pattern in the protein accumulation of the fruit [5, 7], which coincides with the abundant data obtained herein at protein level, and point to a steady state of the protein synthesis process around *véraison*. RNA binding proteins are important cellular regulatory proteins involved in the synthesis, processing, transport, translation and degradation of RNA although little it is known about the detailed roles of most of these proteins that share the occurrence of the RBD protein domain [61]. Some of the RNA binding proteins detected have marked profiles peaking during the first growth period at early or late stages and these results tie in

with previous studies that also suggest higher rates of RNA and protein synthesis when the fruit is forming [2, 21, 62, 63].

The nascent proteins must be correctly folded or processed to acquire its biological function. The main proteins that assist in the correct protein folding, the chaperones, have been detected with a diverse pattern of accumulation along the development of the grapes. HSP90 proteins increase abruptly from FS-to-4mm together with an associated co-chaperone p23; their levels are maintained until the end of the first growth period and then decrease along ripening, in agreement with previous results [2]. HSP90 is an essential molecular chaperone in plant cells with potential key roles in the folding and activation of proteins involved in the control of the cell cycle and signal transduction pathways controlling growth and development [64, 65]. It is right after fruit set when berry cells divide actively.

HSP70 proteins increase in abundance during early development stages and then increase again along ripening. The nascent chains in the cytosol interact with HSP70 together with their co-chaperones (DNAJ/HSP40 and GrpE), which are believed to play a crucial role in preventing aggregation of the unfolded polypeptides. Chaperonins, which acts later in the folding process, such as the GroEL and TRiC families, were also detected. HSP60 chaperonins (GroEL family) that are present in the mitochondrial matrix and the chloroplast stroma have decreasing protein levels along development. A specific class of HSP60, the Rubisco binding-protein that is involved in the Rubisco assembly, had a profile parallel to the proteins of the Calvin cycle (see group 'Carbohydrate and malate metabolism'). In regards to the TRiC family of chaperonins, the subunits theta and epsilon of TCP-1 protein were identified. The TRiC or CCP is a cytosolic complex known as the folding machinery for the cytoskeletal proteins actin, and α- and β-tubulins *in vivo* and *in vitro* [66-68].

Moser *et al.*, [69] determined that CCP is involved in the reorganization of plant microtubular networks. In line with these findings, TCP-1 profiles found here and also in a previous proteomic study [2], more abundant at early development stages, correlate with the profiles found for the cytoskeletal proteins, tubulins and actin, while are opposite to that found for the actin depolymerizing factor (see group 'Cell Division and Growth, Biogenesis').

Several small HSP (sHSP) were detected. An 18.4KDa sHSP exhibited a sharp increase before *véraison*, and two 22KDa and a 17.8KDa sHSP also increased sharply at the end of ripening; sHSP are low molecular mass eukaryotic HSP ubiquitously synthesized in response to multiple stresses and at certain developmental stages [70]. Although the sHSP are apparently not essential for basal cell functions as are the high molecular weight HSP such as HSP90, HSP70 and HSP60, their functions are likely to be critical for survival and recovery from heat stress as well as for specific developmental processes such as fruit ripening [71].

Individual members of each class of chaperones have particular functions but a co-operation between them must be the central principle of the integrated HSP/chaperone machinery [70]. This machinery acts under normal growth conditions or in response to stress. In case of the development of grape berries, a specific deregulation of particular HSPs isoforms seems to occur as was previously hypothesized by da Silva *et al.*, [72].

6. Protein Degradation **(**Additional File 9J**)**

The degradation of proteins is carried out by different complex proteolytic pathways among which the two forms of the proteasome, the 20S core proteasome and 26S proteasome ubiquitin-dependent, are widely distributed and act over cytoplasmic and nuclear proteins. Different subunits of the proteasomes and also subunits of the

ubiquitinating enzyme complex were identified showing a common increasing trend in the ripening phase. With respect to the protein profile along fruit forming phase a high number of proteins belonging to the main degradatory machinery is identified displaying different trends, making it difficult to interpret. The same protein trends were detected in a previous proteomic study using a DIGE approach [2]. The profile of fas1 was in accordance with the proteins involved in the ubiquitin-mediated pathway for protein degradation. Fas1 is a novel protein having multiubiquitin-related domains, which may serve as a scaffolding protein that regulates protein degradation in the ubiquitin-proteasome pathway [73]. However an increase of the protein degrading activity along ripening seems to exist, which might respond to the developmental program of the fruit. It is known that the total N content in the fruit increases particularly the amino acid free fraction [4] and these results tie in with the slowdown effect in the protein synthesis observed above.

A striking feature of this functional group of proteins is the profile of a group of proteases acting on cysteine residues and specifically acting in the secretory pathway. In this sense, two cysteine protease inhibitors or cystatins and two cysteine proteinases were up-regulated from onset of ripening in the same fashion as a cystatin was deregulated as described in a previous proteomic study [2]. It has been demonstrated that plant cysteine proteases and protease inhibitor genes are involved in the regulation of the programmed cell death (PCD) triggered by oxidative stress [74]. Interestingly, Pilati *et al.* [22] observed a modulation of a set of genes involved in enzymatic detoxification for ROS species and ten genes involved in PCD including cysteine proteases and a cystatin gene upon an oxidative burst characterized by the accumulation of H_2O_2 starting at *véraison*. Present results at the protein level suggest an occurrence of a PCD process as a part of the ripening-developmental process in

response to oxidative stress in grape as described for strawberry, a non-climateric fruit, where it is supposed to be linked to the tracheary elements differentiation [75]. In contrast two subtilisin-like proteases, a serine protease, are strongly down-regulated at *véraison,* which recently have been described as analogy to animal caspases participating in the regulation of the PCD process in plants [76].

7. ***Protein Processing*** **(**Additional File 9K**)**

Synthesized proteins must be relocated to the correct subcellular localization. The transport to the different organelles occurs through different import apparatus, which recognize specific targeting domains in the synthesized proteins. Three mitochondrial processing peptidases were identified. Among them, Tom40, the central protein channel in the machinery of the mitochondrial outer membrane that plays an active role in sorting imported proteins [77], undergoes a 2.6-fold increase from FS-to-4mm. In relation to the endoplasmic reticulum function as part of the protein secretory system, the beta-protein component of the 'translocon-associated protein' (TRAPB) complex was detected but not significantly deregulated. In contrast, three isoforms of the nascent polypeptide associated complex (NAC), which competes with the signal recognition particle (SRP) for the binding of the nascent polypeptide as it emerges from the ribosome, were identified; the unique isoform detected in the green developing stages have a strong up-regulation with up to 8-fold change from 7mm-to-15mm. It has been demonstrated that NAC acts as a negative regulator of translocation into the endoplasmic reticulum (ER) preventing the mistargeting of ribosomes containing nascent chains without signal peptide sequence to the ER [78]. The NAC increase along the first growth period and strongly towards *véraison*, which ties in with the general profiles for proteins involved in vesicle trafficking (see discussion group ‘Cell Division and Growth, Biogenesis’) that could be needed for

the secretion of cell wall components and proteins for the expanding cell walls. Moreover, an LBiP protein, involved in facilitating the assembly of multimeric protein complexes inside the ER, shows a moderate increasing profile at ripening, which suggests a possible increase of nascent proteins translocated to ER.

A wide variety of macromolecules are synthesized in the cytoplasm and then transported into the nucleus through the nuclear pore complexes located between the inner and outer nuclear membranes. The two import steps, binding and translocation need from soluble cytosolic factors and energy in form of GTP. Three soluble cytosolic factors were detected at ripening, an importin beta and two nuclear transport factor (NTF2) with increasing levels at *véraison*. Translocation of the complex soluble cytosolic factor-polypeptide with the recognized nuclear localization signal through the pore requires free GTP and a small GTPase, Ran. Two Ran proteins were detected up-regulated along development being the isoform gi|157339297 up-regulated 4.8-fold from FS until 15mm stage.

8. Cell Division and Growth, Biogenesis (Additional File 9L)

Several proteins involved in DNA replication and repair were identified. Among these, a single-strand nucleic acid-binding protein presented high levels in early stages [2]. Histone profiles exhibited a strong up-regulation in early development stages with a 6-fold change from FS-to-4mm, followed by a down-regulation from 4mm with levels decreasing from *véraison* to ripening by around 5.5-fold. These results are in accordance with the well-characterized cell division phases in early development and fruit growth stages at the expense of cell expansion during ripening. The abundance of a transcriptionally controlled tumor protein (VvTCTP) was seen to increase throughout the berry development phase. This VvTCTP presents a 78% similarity with a recently characterized Arabidopsis homologue [79] that acts as a key

protein for cell growth regulation over expressed in meristematic and expanding cells. Thus, VvTCTP might play an important role in the development of grape berry fruit. Cells use GTP-binding proteins and their GTP hydrolysis in diverse cell growth and biogenesis processes. Small GTPases are classified into five distinct families of which the Rab and Arf GTPase families are involved in vesicle trafficking [80]. A large number of them was identified, from which two Rab (Rab 8 and 11) and one Arf (Sar1) proteins were found to be significantly up-regulated during development until the *véraison* phase; in parallel, the abundance of a GDP dissociation inhibition (GDI) protein, a regulatory protein which maintains GTPases in the active state, decreased, thus enabling the GTPase turnover to proceed during this period. Although the functions of most of the Rab proteins from Arabidopsis have not yet been established, Rab GTPase functions have been extensively studied in mammals and yeast. Thus a role for its ortholog genes can be suggested. In this sense, Rab 8 GTPase is targeted to the protein secretion pathway by probably regulating protein excretion [80, 81, 82]. Rab 11C is an AtRABA subfamily member whose RABA isoforms have been suggested to play a role in the delivery of cell wall components. This has been inferred from the results of some antisense experiments in tomato which have produced complex developmental abnormalities and delayed fruit ripening [83]. Indeed the delivery of hemicelluloses, integral cell wall proteins and probably the cellulose synthase complex are likely to occur by the post-Golgi membrane trafficking membrane mechanism [80]. Sar 1 belongs to the Arf family whose isoforms act to recruit cytosolic coat proteins at vesicle budding sites, whereas Sar1 specifically regulates COP-II vesicle formation. All the other Rab and Arf GTPase isoforms detected during ripening were slightly up-regulated. Vesicle trafficking, apart from GTPase specific isoforms, needs associated integral-membrane proteins,

which allow the docking/fusion of vesicles to provide specificity to the mechanism; *e.g.*, SNARE, SNAP. Among other membrane trafficking proteins like sec mutants [84], Sec 14 was found to strongly up-regulate during development until *véraison*, with an overall 12.5-fold change by the end of the first growth period, then the protein levels were maintained throughout ripening. Sec 14 is a homolog to a Patellin-1 protein from *Cucurbita pepo*, a phosphoinositide-binding protein that plays a role in the membrane trafficking events associated with cell plate formation during cytokinesis in Arabidopsis [85]. The profile of Sec 14 is consistent with a similar role in developing grape berries. The fact that proteins levels held after the cell division stage of berries had finished [86] suggests a role for Sec 14 beyond cell plate formation during cell division, and of it being putatively involved in cell expansion through cell wall formation in the following stages until ripening.

Regarding cell growth, the MSBP1 protein was overexpressed at the end of the first growth period by 2.4-fold at 7mm-to-15mm, which coincides with the lag phase before *véraison*. MSBP1 acted as a negative regulator of cell elongation in Arabidopsis [87] and suppressed the brassinosteroid signaling pathway [88], so it could act as a negative regulator of berry growth. Conversely, CDC48, which is a positive regulator of plant cell growth during the cell division phase, was 2-fold down-regulated at same developmental point. Interestingly, the same phenomenon was observed in skin at the end of ripening [16], suggesting a tight involvement of MSBP1 and CDC48 in the control of cell growth.

The primary cell wall of plants is a complex structure of cellulose cross-linked by the hemicelluloses embedded in a pectin polysaccharides matrix. It is thought that homogalacturonan (HGA) is secreted as highly methyl-esterified polymers and then this methyl group is cleaved by the enzyme pectin methylesterase (PME) located in

the cell to permit the start of carboxylate binding to Ca^{2+}. The rhamnose (Rha) units of ramnogalacturonan I (RGI) interrupt the Ca^{2+} junctions and contribute to the cell wall pore definition Both pectic fraction characteristics seemed to be highlighted in the pre-*véraison* phase with a 4-fold up-regulation of PME and the rhamnose biosynthetic enzyme (RhamE) (see Additional File 9D). A similar profile has been detected for RhamE in a previous proteomic analysis [2]. Later, PME was strongly down-regulated in the post-*véraison* phase and, in parallel, was likely inhibitited by an INV/PME inhibitor protein, which was up-regulated, indicating the prevention of de-methylation. The same phenomenon has been observed in grape berry skin [16], indicating that similar processes take place in both tissues [89]. Although no major changes in cell wall composition took place during grape berry softening, a significant modification of specific polysaccharides was observed [90]. At *véraison*, it seems that some hydrolases such as galactosidase (GalAse), xylosidase and glycosidase could be released to act on polymers of the pectic backbone. GalAse activity increased during ripening [91] and seemed at least partly responsible for the loss of RGI, which occurred before softening [90]. It has been speculated that polygalacturonase (PG) can act by decreasing the size of pectic polysaccharides and by increasing their solubility [90]. Despite a PG protein not having been quantified, the levels of a polygalacturonase-inhibiting protein were seen to lower from *véraison*, which would support the above hypothesis.

The biosynthesis of non-cellulosic cell wall polysaccharides use several nucleotide sugars as substrates through several interconversion enzymes like epimerases and dehydratases; some up-regulated during ripening, such as GluDH, ManDH (see Group 'Carbohydrate metabolism').

Another important component of cell walls is structural proteins. These were developmentally regulated and could play a role in stabilizing the cell wall and in maintaining cell wall integrity during ripening. Having completed elongation, the primary cell wall locked the cell into a shape by becoming much less extensible. Proline-rich family proteins (PRPs), together with hydroxyproline-rich glycoproteins (HRGPs), became candidates as key actors in this mechanism by performing the cross-linking of cell wall components. A PRP protein accumulated during ripening, which is in accordance with the results reported of an increasing protein and amino acid content in cell walls during development [90].

Expansion of cells implies loosening the primary cell wall and synthesis, along with the reintegration of material. Fruit softening is one of the most remarkable characteristics to occur during ripening. In the light of the present results, the sharp increase noted in the glycan-modifying enzymes expansins (EXP) and xyloglucan endotransglycosylase (XET), also observed in skin [16], indicates an important role in restructuring tethering by cross-linking glycans, which modify primary cell wall properties and contribute to mesocarp softening, as observed in skin [16]. Two isoforms of XET were quantified with opposite trends. XET1 underwent a 10-fold increase from onset of ripening, in agreement with the expression in both in mesocarp and skin of Kyoho grapes from onset of ripening, which were suspected to be closely related to berry softening [92]. Another XET isoform (gi|147771556) decreased along ripening, showing a 5-fold change and was reported increasing at the end of the first growth period [2]. On the other hand, three expansin (EXP) isoforms were quantified. An alpha-EXP and a beta-EXP displayed a strong increasing trend during ripening, while another alpha-EXP presented very low levels from fruit set-to-7mm to then

significantly increase (10-fold) at pre-*véraison*. Such profiles support a major role of EXPs in fruit softening.

The hemicellulose fraction, apart from being constituted mainly by xyloglucan (XG) and arabinoxylan (AX) components, mannans as part of glucomannan (GM) and galactoglucomannan (GGM) polysaccharides, was also found in the cross-linking glycans of primary cell walls. GM and GGM were characterized by a beta-(1,4)-mannose backbone, which can be interspersed with glucose residues [93]. GM and GGM performed an analog function to XG, that of being able to cross-link the cellulose matrix [94, 95]. Mannan was present in the cell wall polysaccharide fraction from the mesocarp in grape berries, be it in a smaller proportion than XG (3 *vs.* 8 mol %) [96]. The novel activity observed for mannan transglycosylase in plant cell walls, which increased upon ripening in tomato [93], supports the involvement of this transglycosylase in the restructuring cell wall process that occurs in fruit ripening. Interestingly, a beta-(1,4)-mannan-endohydrolase (MET) has been detected in grape berry, which probably undertakes transglycosylase activity by the close clustering with the mannan endohydrolases characterized in other fruits [97]. In this study, MET has been detected for the first time. It accumulated from the onset of ripening with a change during maturation from 2.5- up to 3.7-fold, and it could be a new candidate to act in coordination with XET and other cell wall-loosening enzymes in grape berry fruit softening.

9. Defense Proteins (Additional File 9M*)*

The defence potential arsenal in grape berries encloses pathogenesis-related proteins (PR), which act preventing pathogen multiplication or spread [98]. It is known since long that grape berries accumulate this class of proteins upon onset of ripening [99, 101] as part of the normal fruit development program because its expression in

apparently healthy berries increases abruptly at ripening [7, 102]. Among the wide diversity of PR-proteins, members of PR-2, PR-3, PR-4, PR-5, PR-10 and PR-14 families have been detected deregulated in this study being accumulated upon triggering of ripening except for the proteins belonging to PR-10 and PR-14 families.

During green development, only three proteins that belong to PR-10 and PR-14 families were identified. PR-10.7 protein increases in abundance towards fruit set and the two lipid transfer proteins (LTP) from PR-14 family increase towards *véraison*. PR-10.7 is highly homologous to Mald1, the major apple allergen [103]. PR-10 proteins are structurally related to ribonucleases and it is hypothesized that these proteins provide protections against viruses [98].

The other two lipid transfer proteins (LTPs) that belong to PR-14 family, with a moderate increase up to 7mm followed by an abrupt decrease before *véraison*, clearly point to a role for them during the first growth phase. LTPs are basic 9 KDa proteins that can enhance the *in vitro* transfer of phospholipids between membranes and can bind acyl chains. On the basis of these properties, LTPs were thought to participate in membrane biogenesis and regulation of the intracellular fatty acid pools. The presence of a signal peptide indicates that LTPs could enter the secretory pathway and they were found being secreted and located in the cell wall [104].

The rest of PR proteins identified are strongly accumulated at *véraison* and along ripening and include a β-1,3-glucanase (PR-2) (b-Gluc), two class IV chitinase isoforms (PR-3), two thaumatin-like proteins (PR-5) (TL) and a PR-4 protein. Endo-chitinases are known to be abundant in mesocarp and grape juices [105] and are supposed to play a role in berry protection because of its antifungal activity. TL proteins, which are presumed to have an extracellular location [100] in grape, are systematically expressed in many ripe fruit and potentially involved in the resistance

to pathogen or defence in grapes [7, 71]. TL proteins belongs to PR-5 family in which the osmotin members are related to the degree of osmotic stress but the TL proteins' role in osmotic stress adaptation or response to such stresses remains unknown.

Also, two lipases-like SGNH containing GDSL-like motif have been found with a moderate increase at ripening. These proteins were detected in the grapevine cell culture extracellular medium increasing in response to elicitation with cyclodextrin [106]. And this response was interpreted by authors as similar to that produced against a pathogen attack.

BGluc whose profile coincides with previous proteomic and transcriptomic reports [16, 89] is believed to play a role in fruit ripening and softening [107, 108].

Interestingly, a β-glycosidase (IGBG4-L), which was similar to a homologous isoform G4 active in flavonol 3-O- and anthocyanidin 3-O-glucoside in *Medicago truncatula* [109], was moderately up-regulated from ripening initiation in the mesocarp. The *M. truncatula* G4 β-glycosidase was induced in cell suspensions by methyl jasmonate; it has been proposed to play a role in the mobilization of isoflavonoid phytoalexins. The same protein was also found to be strongly up-regulated during *véraison* in grape berry skins, and it has been suggested to play a role in defense [16]. However, its presence also in the ripening grape berry mesocarp, where flavonols glycosides, but not anthocyanins, accumulate, suggests a role beyond the biosynthetic pathway of this class of flavonoids, which is still to be explored.

10. Other Proteins of Interest **(**Additional File 9N*)*

Four proteins were detected strongly deregulated relating with the ripening process. The proteins identified here, Grip22 and Grip32, were described as berry- and ripening-specific and Grip31 and Grip68, although not being grape and ripening-specific, showed significantly higher transcript levels at ripening [110]. Its protein

levels run in parallel with the transcripts profiles determined by Davies and Robinson [110]. The function of these proteins remains unknown although a sequence homology analysis may suggest a putative function. Grip31 is homologous to the allergenic related protein Pt2L4 (42%) and to the AG13 protein (44%). And it has been speculated that these homologous proteins perform a structural role in cell walls due to their high Pro content and hydrophilic nature [111]. Grip68 does not have protein homology, thus unbeing known any ortholog gene. Grip22 has homology with kiwellin protein (76%), a new kiwi fruit allergen, which represents one third of kiwi total protein content [112]. The Grip 32 protein is homologous to a SRC1 protein (46%) and several other proteins named as cold- and water-stress response proteins (45-46%), which may be involved in the adaptation of plants to low-temperature stress. The presence of a putative nuclear targeting signal indicates that the Grip 32 putative protein may be located in the nucleus.

Another interesting protein has been detected with a strong up-regulation of up to 20-fold from the onset to the end of ripening. ABA-induced plasma membrane associated protein (AIP) belongs to the AWPM-19 family protein whose members are 19 kDa membrane proteins. It is known that the levels of the plant protein AWPM-19 increase dramatically when there is an increase level of ABA. The increasing presence of this protein has been related to a greater tolerance of freezing [113]. In grape berries, AIP protein is strongly accumulated after *véraison*, which may also be triggered by ABA levels in the fruit. AIP identified here is a membrane protein with a signal peptide targeting to the secretory pathway. The implication of the homologous protein AWPM-19 in response to ABA treatments indicates that the novel protein identified in the mesocarp of ripe berries, AIP, is relevant to be considered as a candidate to study its role in the ripening process.

Three seed storage proteins (SSP) were detected, two of them were sharply up-regulated at the end of ripening, homologous to a cruciferin and a vicillin precursor, while the legumin-like protein was not significantly deregulated. Ripening is an aspect of fruit development that is initiated after seed maturation has almost been completed, supporting the hypothesis that seeds influence fruit development and ripening. The grape embryo grows after seed development has ceased and reaches full size along the ripening phase [114]. It seems that the mesocarp continues synthesizing storages for the developing embryo. In particular, two storage proteins rise strongly from 110g/l-to140g/l in order to prepare seeds before their dispersion after fruit maturation. According to these findings, thioredoxins type h (TRXh) and the chloroplastic type m (TRXm) increases along the first growth phase and ripening (Additional File 9H). TRXh is involved in multiple processes although the best characterized is its implication in the reserve breakdown that sustains early seedling growth of germinating cereal seeds [115]. Among the known target proteins of TRXh in seeds are storage proteins such as hordeins in barley [116] and glutenins and gliadins in wheat [117], which are insolubilized in disulfide-bound complexes during maturation and drying.

11. Unknown Proteins (Additional File 9O*)*

This group of proteins includes those not sequenced and described to date. They are all annotated as hypothetical proteins or unknown proteins, and no blast hits with a description can be matched. This is an especially interesting group because it could prove remarkable in the search of protein biomarkers; indeed some identified proteins displayed a strong deregulation during development, mostly from 7-to-15mm and 15mm-to-V100, when fruit underwent dramatic changes at both the molecular and physiological levels.

References

1. Conde C, da Silva P, Fontes N, Dias ACP, Tavares RM, Sousa MJ, Agasse A. Delrot S, Gerós H: **Biochemical changes throughout grape berry development and fruit and wine quality.** *Food* 2007, **1**:1-22.

2. Martínez-Esteso MJ, Sellés-Marchart S, Lijavetzky D, Pedreño MA, Bru-Martínez R: **A DIGE-based quantitative proteomic analysis of grape berry flesh development and ripening reveals key events in sugar and organic acid metabolism.** *J Exp Bot* 2011, **62** (8):2521-2569.

3. Deluc LG, Grimplet J, Wheatley MD, Tillet RL, Quilici D, Osborne C, Schlauch KA, Schooley DA, Cushman JC, Cramer GR: **Transcriptomic and metabolite analyses of Cabernet Sauvignon grape berry development.** *BMC Genomics* 2007, **8**:429.

4. Stines AP, Grubb J, Gockowiak H, Henschke PA, Høj PB, van Heeswijck R: **Proline and arginine accumulation in developing berries of *V. vinifera* in Australian vineyards: influence of vine cultivar, berry maturity and tissue type.** *Aust J Grape Wine Res* 2000, **6**:150-158.

5. Ghisi R, Jannini B, Passera C: **Changes in the activities of enzymes involved in nitrogen and sulphur assimilation during leaf and berry development of *Vitis vinifera*.** *Vitis* 1984, **23**:257-267.

6. Sieciechowicz KA, Joy KW, Ireland RJ: **The metabolism of asparagines in plants.** *Phytochemistry* 1988, **27**:663-671.

7. Tattersall DB, van Heeswijck R, PB Hoj: **Identification and characterization of a fruit -specific thaumatin-like protein which accumulates at very low levels in conjunction with the onset of sugar accumulation and berry softening in *V. vinifera*.** *Plant Physiol* 1997, **114**:759-769.

8. Sauter M, Lorbiecke R, Yang B, Pochapsky TC, Rzewuski G: **The immediate-early ethylene response gene OsARD1 encodes an acireductone dioxygenase involved in recycling of the ethylene precursor S-adenosylmethionine.** *Plant J* 2005, **44**:718-729.

9. Chervin C, El-Kereamy A, Roustan JP, Latche A, Lamon J, Bouzayen M: **Ethylene seems required for the berry development and ripening in grape, a non-climacteric fruit.** *Plant Sci* 2004, **167**:1301-1305.

10. Croteau RB, David EM, Ringer KL Wildung MR: **Menthol biosynthesis and molecular genetics.** *Naturwissenschaften* 2005, **92**:562-577.

11. Ringer KL, Davis EM, Croteau R: **Monoterpene metabolism. Cloning, expression, and characterization of (-)-isopiperitenol/(-)-carveol dehydrogenase of peppermint and spearmint.** *Plant Physiol* 2005, **137**(3):863-72.

12. Fenoll J, Manso A, Hellin P, Ruiz L, Flores P: **Changes in the aromatic composition of the Vitis vinifera grape Muscat Hamburg during ripening**. *Food Chem* 2009, **114**:420-428.

13. Zhang DY, Wu J, Ye F, Xue L, Jiang S, Yi J, Zhang W, Wei H, Sung M, Wang W, Li X: **Inhibition of cancer cell proliferation and prostaglandin E2 synthesis by *Scutellaria baicalensis*.** *Cancer Res* 2003, **63**:4037-4043.

14. Baydar NG, Harmankaya N: **Changes in endogenous hormone levels during the ripening of grape cultivars having different berry set mechanisms.** *Turk J Agric For* 2005, **29**:205-210.

15. Symons GM, Davies C, Shavrukov Y, Dry IB, Reid JB, Thomas MR: **Grapes on steroids. Brassinosteroids are involved in grape berry ripening.** *Plant Physiol* 2006, **140**:150-158.

16. Martínez-Esteso MJ, Casado-Vela J, Sellés-Marchart S, Elortza F, Pedreño MA, Bru-Martínez R: **iTRAQ-based profiling of grape berry exocarp proteins during ripening using a parallel mass spectrometric method.** *Mol Biosyst* 2011, **7**(3):749-65.

17. Hale CR, Coombe BG, Hawker JS: **Effects of ethylene and 2-chloroethylphosphonic acid on the ripening of grapes.** *Plant Physiol* 1970, **45**:620-623.

18. Tira-Umphon A, Roustan JP, Chervin C: **The stimulation by ethylene of the UDP glucose-flavonoid 3-O-glucosyltransferase (UFGT) in grape tissues is independent from MybA transcription factors.** *Vitis* 2007, **46**(4):210-211.

19. Grimplet J, Deluc LG, Tillett RL, Wheatley MD, Schlauch KA, Cramer GR, Cushman JC: **Tissue-specific mRNA expression profiling in grape berry tissues.** *BMC Genomics* 2007, **8**:187.

20. Manning K: **Detoxification of cyanide by plants and hormone action.** *Ciba F Symp* 1988, **140**:92-110.

21. Giribaldi M, Perugini I, Sauvage FX, Shubert A: **Analysis of protein changes during grape berry ripening by 2-DE and MALDI-TOF.** *Proteomics* 2007, **7**:3154-3170.

22. Pilati S, Perazzolli M, Malossini A, Cestaro A, Dematte L, Fontana P, Dal Ri A, Viola R, Velasco R, Moser C: **Genome-wide transcriptional analysis of grapevine berry ripening reveals a set of genes similarly modulated during three seasons and the occurrence of an oxidative burst at véraison.** *BMC Genomics* 2007, **8**:428.

23. Kondo S, Tomiyama A, Seto H: **Changes of endogenous jasmonic acid and methyl jasmonate in apples and sweet cherries during fruit development.** *J Am Soc Hort Sci* 2000, **125**:282-287.

24. Coombe BG, Hale CR: **The hormone content of ripening grape berries and the effects of growth substance treatments.** *Plant Physiol* 1973, **51**:629-634.

25. Hale CR, Coombe BG: **Abscisic acid: an effect on the onset of ripening of grapes (*Vitis vinifera L.*).** *Royal Soc N Z Bull* 1974. **12:**831-836.

26. Cawthon DL, Morris JR: **Relationship of seed number and maturity to berry development, fruit maturation, hormonal changes, and uneven ripening of Concord (*Vitis labrusca* L.) grapes.** *J Am Soc Hort Sci* 1982, **107**:1097-1104.

27. Kataoka I, Sugiura A, Utsunomiya N, Tomana T: **Effect of abscisic acid and defoliation on anthocyanin accumulation in Kyoho grapes (*Vitis vinifera* L. x *V. labruscana* BAILEY).** *Vitis* 1982, **21**:325-332.

28. Okamoto G, Kuwamura T, Hirano K: **Effects of water deficit stress on leaf and berry ABA and berry ripening in Chardonnay grapevines (*Vitis vinifera*).** *Vitis* 2004, **43**:15-17.

29. Deytieux-Belleau C, Gagné S, L'Hyvernay A, Donèche B, Geny L: **Possible roles of both abscisic acid and indolacetic acid in controlling grape berry ripening process**. *J Int Sci Vigne Vin* 2007, **41**:141-148.

30. Inaba A, Ishidia M, Sobajima Y: **Changes in endogenous hormone concentrations during berry development in relation to ripening of Delaware grapes**. *J Jpn Soc Hort Sci* 1976, **45**(3):245-252.

31. Scienza A, Miravalle R, Visai C, Fregoni M: **Relationship between seed number, gibberellin and abscisic acid levels and ripening in cabernet sauvignon grape berries.** *Vitis* 1978, **17**:361-368.

32. Davies C, Boss PK, Robinson SP: **Treatment of grape berries, a nonclimacteric fruit with a synthetic auxin, retards ripening and alters the expression of developmentally regulated genes.** *Plant Physiol* 1997, **115**,1155-116.

33. Kondo S, Kawai M: **Relationship between free and conjugated ABA levels in seeded and gibberellin-treated seedless, maturing 'Pione' grape berries.** *J Am Soc Hortic Sci* 1998, **123**:750-754.

34. Matsushima J, Hiratsuka S, Taniguchi N, Wada R, Suzaki N: **Anthocyanin accumulation and sugar content in the skin of grape cultivar 'Olympia' treated with ABA.** *J Jpn Soc Hort Sci* 1989, **58**:551-555.

35. Cakir B, Agasse A, Gaillard C, Sumonneau A, Delrot S, Atanasova R: **A grape ASR protein involved in sugar and abscisic acid signaling.** *Plant Cell* 2003, **15**:2165-2180.

36. Davies C, Böttcher C: **Hormonal control of grape berry ripening.** In *Grapevine molecular physiology and biotechnology*. Edited by Roubelakis-Angelakis KA. The Netherlands: Springer; 2009:229-261.

37. Logan DC, Domergue O, Teyssendier de la Serve T, Rossignol M: **A new family of plasma membrane polypeptides differentially regulated during plant development.** *Biochem Mol Biol* 1997, **43(**5):1051-1062.

38. Tang W, Kim T-W, Oses-Prieto J-A, Sun Y, Deng Z, Zhu S, Wang R, Burlingame AL, Wang Z-Y: **Transduction from the receptor kinase BRI1 in *Arabidopsis*.** *Science* 2008. **321**:557-560.

39. Lücker J, Laszczak M, Smith D, Lund ST: **Generation of a predicted protein database from EST data and application to iTRAQ analyses in grape (*Vitis vinifera* cv. Cabernet Sauvignon) berries at ripening initiation.** *BMC Genomics* 2009, **10**:1-17.

40. Kondo S, Fukuda F: **Changes of jasmonates in grape berries and their possible roles in fruit development.** *Scientia Hort* 2001, **91**:275-288.

41. Wasilewska A, Vla F, Sirichandr C, Redkob Y, Jammesc F, Valona C, Frei N, Leunga J: **Na update on abscisic acid signaling in plants and more....** *Mol plant* 2008, **1**(2):198-217.

42. Xu S: **Abscisic acid activates a Ca^{2+}-calmodulin-stimulated protein kinase involved in antioxidant defense in maize leaves.** *Acta Biochim Biophys Sin* 2010, **42**(9):646-655.

43. Lee S, Lee EJ, Yang EJ, Lee JE, Park AR, Song WH, Park OK: **Proteomic identification of annexins, calcium-dependent membrane binding proteins that mediate osmotic stress and abscisic acid signal transduction in *Arabidopsis*.** *Plant Cell* 2004, **16**:1378-1391.

44. Munnik T, Arisz SA, Vrije T, Musgrave A: **G protein activation stimulates phospholipase D signaling in plants.** *Plant Cell* 1995, **7**:2197-2210.

45. Noctor G, Foyer CH: **Ascorbate and glutathione: keeping active oxygen under control.** *Annu Rev Plant Phys* 1998, **49**:249-279.

46. Jimenez A, Creissen G, Kular B, Firmin J, Robinson S, Verhoeyen M, Mullineaux P: **Changes in oxidative processes and components of the antioxidant system during tomato fruit ripening.** *Planta* 2002, **214**:751-758.

47. Yang EJ, Oh YA, Lee ES, Park AR, Cho SK, Yoo YJ, Park OK: **Oxygen-evolving enhancer protein 2 is phosphorylated by glycine-rich protein 3/wall-associated kinase 1 in Arabidopsis.** *Biochem Bioph Res Co* 2003, **305**:862-868.

48. Carpita N, Sabularse D, Montezinos D, Delmer DP: **Determination of pore size of cell walls of living plant cells.** *Science* 1979, **205**:1144-1147.

49. Close TJ: **Dehydrins: emergence of a biochemical role of a family of plant dehydration proteins.** *Physiol Plant* 1996, **97**:795-803.

50. Parra MDM, Del pozo O, Lona R, Godoy JA, Pintor-Toro JA: **Structure of the dehydrine tas14 gene of tomato and its developmental and environmental regulation in transgenic tobacco.** *Plant Mol Biol* 1996, **32**:453-460.

51. Giordani T, Natali L, D'Ercole A, Pugliesi C, Fambrini M, Vernieri P, Vitagliano C, Cavallini A: **Expression of a dehydrin gene during embryo development and drought stress in ABA-deficient mutans of sunflower (*Helianthus annuus* L.).** *Plant Mol Biol* 1999, **39**:739-748.

52. Ouvrard O, Cellier F, Ferrare K, Tousch D, Lamaze T, Dupuis JM, Casse-Delbard F: **Identification and expression of water stress- and abscisic acid-regulated genes in grought-tolerance sunflower genotype.** *Plant Mol Biol* 1996, **31**:819-829.

53. Liu Y, Li H, Shi Y, Song Y, Wang T, Li Y: **A maize early responsive to dehydration gene, ZmERD4, provides enhanced drought and salt tolerance in *Arabidopsis*.** *Plant Mol Biol* 2009**, 27**(4):542-548.

54. Maqbool A, Zahur M, Husnain T Riazuddin S: **GUSP1 and GUSP2, two drought-responsive genes in *Gossypium arboreum* have homology to universal stress proteins.** *Plant Mol Biol Rep* 2009, **27**:109-114.

55. Zahur M, Maqbool A, Irfan M, Younas M, Barozai K, Rashid B, Riazuddin S, HusnainT: **Isolation and functional analysis of cotton universal stress protein promoter in response to phytohormones and abiotic stresses.** *J Mol Biol* 2009, **43**:578-585.

56. Coombe BG: **Australian temperate fruits review conference. Fruit setting, development and ripening.** *Acta Hort* 1988, **240**:209-216.

57. Negri AS, Prinsi B, Rossoni M, Failla O, Scienza A, Cocucci M, Espen L: **Proteome changes in the skin of the grape cultivar Barbera among different stages of ripening.** *BMC Genomics* 2008, **9**:378.

58. Dry IB, Robinson SP: **Molecular cloning and characterization of grape berry polyphenol oxidase.** *Plant Mol Biol* 1994, **26**:495-502.

59. Kuwabara T: **The 60-kDa precursor to the dithiothreitol-sensitive tetrameric protease of spinach thylakoids: structural similarities between the protease and polyphenol oxidase.** *FEBS Letters* 1995, **371**:195-198.

60. Thipyapong P, Melkonian J, Wolfe DW, Steffens JC: **Suppression of polyphenol oxidases increases stress tolerance in tomato.** *Plant Sci* 2004, **167**:693-703.

61. Fedoroff NV: **RNA-binding proteins in plants: the tip of an iceberg?.** Curr Opin Plant Biol 2002, **5**:452-459.

62. Terrier N, Francois-Xavier S, Ageorges A, Romieu C: **Changes in acidity and in proton transport at the tonoplast of grape berries during development.** *Planta* 2001, **213**:20-28.

63. Waters DLE, Holton TA, Ablett EM, Lee LS, Henry RJ: **cDNA microarray analysis of the developing grape (*Vitis vinifera* cv. Shiraz) berry skin.** *Funct Integr Genomics* 2005, **5**:40-58.

64. Schumaner K, Chory J: **Brassinosteroid signal transduction: still casting the actors.** *Curr Opin Plant Biol* 2000, **3**(1):79-84.

65. Krishna P, Gloor G: **The Hsp90 family of proteins in *Arabidopsis thaliana*.** *Cell Stress Chaperon* 2001, **6**(3):238-246.

66. Lewis VA, Hynes GM, Zheng D, Saibil H, Willison K: **T-complex polypeptide-1 is a subunit of a heteromeric particle in the eukaryotic cytosol.** *Nature* 1992, **358**:249-252.

67. Yaffe MB, Farr GW, Miklos D, Horwich AL, Sternlicht ML, Sternlicht H: **TCP1 complex is a molecular chaperone in tubulin biogenesis.** *Nature* 1992, **358**:245-248.

68. Sternlicht, H., Farr, G. W., Sternlicht, M. L., Driscoll, J. K., Willison, K. and Yaffe, M. B: **The t-complex polypeptide 1 complex is a chaperonin for tubulin and actin in vivo.** *Proc Nat Acad Sci USA* 1993, **90**, 9422-9426.

69. Moser M, Schäfer E, Ehmann B: **Characterization of protein and transcript levels of the chaperonin containing tailless complex protein-1 and tubulin during light-regulated growth of oat seedlings.** *Plant Physiol* 2000, **124**(1):313-320.

70. Wang W, Scali M, Vignani R, Spadafora A, Sensi E, Mazzuca S, Cresti M: **Protein extraction for two-dimensional electrophoresis from olive leaf, a plant tissue containing high levels of interfering compounds.** *Electrophoresis* 2003, **24**:2369-2375.

71. Waters EJ, Shirley NJ, Williams PJ: **Nuisance proteins of wine are grape pathogenesis-related proteins.** *J Agr Food Chem* 1996, **44**:3-5.

72. da Silva FG; Iandolino A, Al-Kayal F, Bohlmann MC, Cushman MA, Lim H, Ergul A, Figueroa R, Kabuloglu EK, Osborne C, Rowe J, Tattersall E, Leslie A, Xu J, Baek JM, Cramer JR, Cushman JC, Cook DR: **Characterization the grape transcriptome. Analysis of expressed sequence tags from multiple Vitis species and development of a compendium of gene expression during berry development.** *Plant Physiol* 2005, **239**:574-597.

73. Song EJ, Yim SH, Kim E, Kim NS, Lee KJ: **Human fas-associated factor 1, interacting with ubiquitinated proteins and valosin-containing protein, is involved in the ubiquitin-proteasome pathway.** *Mol Cell Biol* 2005, **25**(6):2511-2524.

74. Salomon M, Belenghi B, Delledonne M, Menachem E, Levinea A: **The involvement of cysteine proteases and protease inhibitor genes in the regulation of programmed cell death (PCD) in plants.** *Plant Cell* 1999, **11**:431-443.

75. Aharoni A, Keizer LCP, Van Den Broeck HC, Blanco-Portales R, Muñoz-Blanco J, Bois G, Smit P, De Vos RCH, O'Connell AP: **Novel insight into vascular, stress, and auxin-dependent and -independent gene expression programs in strawberry, a non-climacteric fruit.** *Plant Physiol* 2002, **129**(3):1019-1031.

76. Vartapetian AB, Tuzhikov AI, Chichkova NV, Taliansky M, Wolpert TJ: **A plant alternative to animal caspases: subtilisin-like proteases.** *Cell Death Differ* 2011, **18**:1289-1297.

77. Gabriel K, Egan B Lithgow T: **Tom40, the import channel of the mitochondrial outer membrane, plays an active role in sorting imported proteins.** *EMBO J* 2003, **22**: 2380-2386.

78. Lauring B, Sakai H, Kreibich G, Wiedmann M: **Nascent polypeptide-associated complex protein prevents mistargeting of nascent chains to the endoplasmic reticulum.** *P Natl Acad Sci USA* 1995, **92**:5411-5415.

79. Berkowitz O, Jost R, Pollmann S, Masle J: **Characterization of TCTP, the Translationally Controlled Tumor Protein, from Arabidopsis thalian.** *Plant Cell* 2008, **20**:3430-3447.

80. Vernoud V, Horton AC, Yang Z, Nielsen V: **Analysis of the small GTPase gene superfamily of *Arabidopsis*.** *Plant Physiol* 2003, **131**:1191-1208.

81. Huber LA, de Hoop MJ, Dupree P, Zerial M, Simons K, Dotti C: **Protein transport to the dendritic plasma membrane of cultured neurons is regulated by rab8p.** *J Cell Biol* 1993, **123**:47-55.

82. Huber LA, Pimplikar S, Parton RG, Virta H, Zerial M, Simons K: **Rab8, a small GTPase involved in vesicular traffic between the TGN and the basolateral plasma membrane.** *J Cell Biol* 1993, **123**:35-45.

83. Lu C, Zainal Z, Tucker GA, Lycett GW: **Developmental abnormalities and reduced fruit softening in tomato plants expressing an antisense Rab11 GTPase gene.** *Plant Cell* 2001, 13:1819-1833.

84. Kaiser C, Schekman R: **Distinct sets of SEC genes govern transport vesicle formation and fusion early in the secretory pathway.** *Cell* 1990, **61**:723-733.

85. Peterman TK, Ohol YM, McReynolds LJ, Luna EJ: **Patellin1, a novel Sec14-like protein, localizes to the cell plate and binds phosphoinositides.** *Plant Physiol* 2004, **136**:1-15.

86. Harris JM, Kriedmann PE, Possingham JV: **Anatomical aspects of grape berry development.** *Vitis* 1968, **7**:106-119.

87. Yang XH, Xu ZH, Xue HW: **Arabidopsis membrane steroid binding protein 1 is involved in inhibition of cell elongation.** *Plant Cell* 2005, **17**:116-131.

88. Song L, Shi Q-M, Yang X-H, Xu Z-H, Xue H-W: **Membrane steroid-binding protein 1 (MSBP1) negatively regulates brassinosteroid signaling by enhancing the endocytosis of BAK1.** *Cell Res* 2009, **19**:864-876.

89. Schlosser J, Olsson N, Weis M, Reid K, Peng F, Lund S, Bowen P: **Cellular expansion and gene expression in the developing grape (*Vitis vinifera* L.).** *Protoplasma* 2008, **232**:255-265.

90. Nunan KJ, Sims LM, Bacic A, Robinson SP, Fincher GB: **Changes in cell wall composition during ripening of grape berries.** *Plant Physiol* 1998, **118**:783-792.

91. Nunan KJ, Davies C, Robinson SP, Fincher GB: **Expression patterns of cell wall-modifying enzymes during grape berry development.** *Planta* 2001, **214**:257-264.

92. Ishimaru M, Kobayashi S: **Expression of a xyloglucan endo-transglycosylase gene is closely related to grape berry softening.** *Plant Sci* 2002, **162**:621-628.

93. Schröder R, Wegrzyn TF, Bolitho KM, Redgwell RJ: **Mannan transglycosylase: a novel enzyme activity in cell walls of higher plants**. *Planta* 2004, **219**:590-600.

94. Schröder R, Atkinson RG, Langenkämper G, Redgwell RJ: **Biochemical and molecular characterisation of xyloglucan endotransglycosylase from ripe kiwifruit.** *Planta* 1998, **204**:242-251.

95. Whitney SEC, Brigham JE, Darke AH, Reid JSG, Gidley MJ: **Structural aspects of the interaction of mannan-based polysaccharides with bacterial cellulose.** *Carbohyd Res* 1998, **307**:299-309.

96. Nunan KJ, Sims IM, Bacic A, Robinson SP, Fincher GB: **Isolation and characterization of cell walls from the mesocarp of mature grape berries (*Vitis vinifera*).** *Planta* 1997, **203**:93-100.

97. Schröder R,Atkinson RG,Redgwell RJ: **Re-interpreting the role of endo-β-mannanases as mannan endotransglycosylase/hydrolases in the plant cell wall.** *Ann Bot* 2009, **104**(2):197-204.

98. van Loon LC, van Strien EA: **The families of pathogenesis related proteins, their activities, and comparative analysis of PR-1 type proteins.** *Physiol Mol Plant P* 1999, **55**:85-97.

99. Tattersall D, Pocock K, Hayasaka Y, Adams K, van Heeswijck R, Waters, E, Hoj P: **Pathogenesis related proteins, their accumulation in grapes during berry growth and their involvement in white wine heat instability. Current knowledge and future perspectives in relation to winemaking practices.** In *Molecular Biology and Biotechnology of the Grapevine.* Edited by: Roubelakis-Angelakis KA. New York: Kluwer academic publishers; 2001:183-201.

100. Tattersall DB: **Identification and characterization of *Vitis vinifera* pathogenesis-related proteins that accumulate during berry ripening.** *PhD thesis.* Adelaide university; 1999.

101. Robinson SP, Jacobs AK, Dry IB: **A class IV chitinase is highly expressed in grape berries during ripening.** *Plant Physiol* 1997, **114**:771-778.

102. Pocock KF, Hayasaka Y, McCarthy MG, Waters EJ: **Thaumatin-like proteins and chitinases, the haze-forming proteins of wine, accumulate during ripening of grape (*Vitis vinifera*) berries and drought stress does not affect the final levels per berry at maturity.** *J Agric Food Chem* 2000, **48**(5):1637-1643.

103. Gao ZS, van de Weg WE, Schaart JG, van der Meer IM, Kodde L, Laimer M, Breiteneder H, Hoffmann-Sommergruber K, Gilissen LJ: **Linkage map positions and allelic diversity of two Mal d 3 (non-specific lipid transfer protein) genes in the cultivated apple (*Malus domestica*).** *Theor Appl Genet* 2005, **13**:1432-1442.

104. Kader J-C: **Lipid-transfer proteins in plants.** *Annu Rev Plant Phys* 1996, **47**: 627-654.

105. Vincenzi S, Curioni A: **Anomalous electrophoretic behavior of a chitinase isoform from grape berries and wine in glycol chitin containing sodium dodecyl sulfate-polyacrylamide gel electrophoresis gels.** *Electrophoresis* 2005, **26**:60-63.

106. Martínez-Esteso MJ, Selles-Marchart S, Vera-Urbina JC, Pedreño MA, Bru-Martínez R: **Changes of defense proteins in the extracellular proteome of grapevine (*Vitis vinifera* cv. Gamay) cell cultures in response to elicitors.** *J Proteomics* 2009, **73:**331-341.

107. Cosgrove DJ: **Expansive growth of plant cell walls.** *Plant Physiol Bioch* 2000, **38**:109-124.

108. Wang W, Bianchi L, Scali M, Liu L, Bini L, Cresti M: **Proteomic analysis of β-1,3-glucanase in grape berry tissues.** *Acta Physiol Plant* 2009, **31**(3):597-604.

109. Naoumkina M, Farag MA, Sumner LW, Tang Y, Liu C-J, Dixon RA: **Different mechanisms for phytoalexin induction by pathogen and wound signals in Medicago truncatula.** *P Natl Acad Sci USA* 2007, **13**(46):17909-17915.

110. Davies C, Robinson SP: **Differential screening indicates a dramatic change in mRNA profiles during grape berry ripening. Cloning and characterization of cDNAs encoding putative cell wall and stress response proteins.** *Plant Physiol* 2000, **122**:803-812.

111. Guan C, Akkermans ADL, van Kammen A, Bisseling T, Pawlowski K: **ag13 is expressed in *Alnus glutinossa* nodules in infected cells during endosymbiont degradation Kiwellin, a novel protein from kiwi fruit. Purification, biochemical**

characterization and identification as an allergen. *Physiol Plant* 1997, **99**(1):601-607.

112. Tamburrini M, Cerasuolo I, Carratore V, Stanziola AA, Zofra S, Romano L, Camardella L, Ciardiello MA: **Kiwellin, a novel protein from kiwi fruit. Purification, biochemical characterization and identification as an allergen.** *Protein J* 2005, **24**(7-8):423-429.

113. Koike M, Takezawa D, Arakawa K, Yoshida S: **Accumulation of 19-kDa plasma membrane polypeptide during induction of freezing tolerance in wheat suspension cultures cells by abscisic acid.** *Plant Cell Physiol* 1997, **38**:707-716.

114. Matsui H: **A study on the maturation of Delaware grape berry.** PhD thesis. Osaka Prefecture University, 1976.

115. Wong JH, Kim YB, Ren PH, Cai N, Cho MJ, Hedden P, Lemaux PG, Buchanan BB: **Transgenic barley grain overexpressing thioredoxin shows evidence that the starchy endosperm communicates with the embryo and the aleurone.** *Proc Natl Acad Sci USA* 2002, **99**:16325-16330.

116. Yano H, Wong JH, Lee YM, Cho MJ, Buchanan BB: **A strategy for the identification of proteins targeted by thioredoxin.** *Proc Natl Acad Sci USA* 2001, **98**:4794-4799.

117. Wong JH, Balmer Y, Cai N, Tanaka CK, Vensel WH, Hrukman WJ, Buchanan BB: **Unravelling thioredoxin-linked metabolic processes of ceral starchy endosperm using proteomics.** *FEBS Lett* 2003, **547**:151-156.
