# Supplementary material for: iTRAQ-based protein profiling provides insights into the central metabolism changes driving grape berry development and ripening
Source: BMC Plant Biol. 2013 Oct 24;13:167. doi: 10.1186/1471-2229-13-167 (PMC4016569; doi:10.1186/1471-2229-13-167)
Supplement: Additional file 12 — Phylogram of the grapevine GSTs identified in grape berry during development [104]. [file 1471-2229-13-167-S12.pdf]

**Additional Figure 12. Phylogram of the grapevine GSTs identified in grape berry during development.** A multiple sequence alignment was performed with the ClustalW2 [104] interface on the EBI website using the following parameters: full alignment, Gonnet 250 matrix, penalties for gap opening 10, gap extension 0.2, gap separation 4 and end gap exclusions yes. Weighting factors are provided next to each sequence accession number. The GSTs which accumulated in the grape berry exocarp [36] are marked with a horizontal purple arrow, those which accumulated in the elicited grape cell culture, leading to resveratrol secretion [65], are marked with a horizontal brown arrow, and those which were quantified in the present study are marked with a horizontal yellow arrow. The GSTs identified herein are marked with a green vertical arrow if they were identified in the green stages experiment, or in light purple in the ripe stages experiment. The direction of the arrow also indicates if proteins were down- or up-regulated.

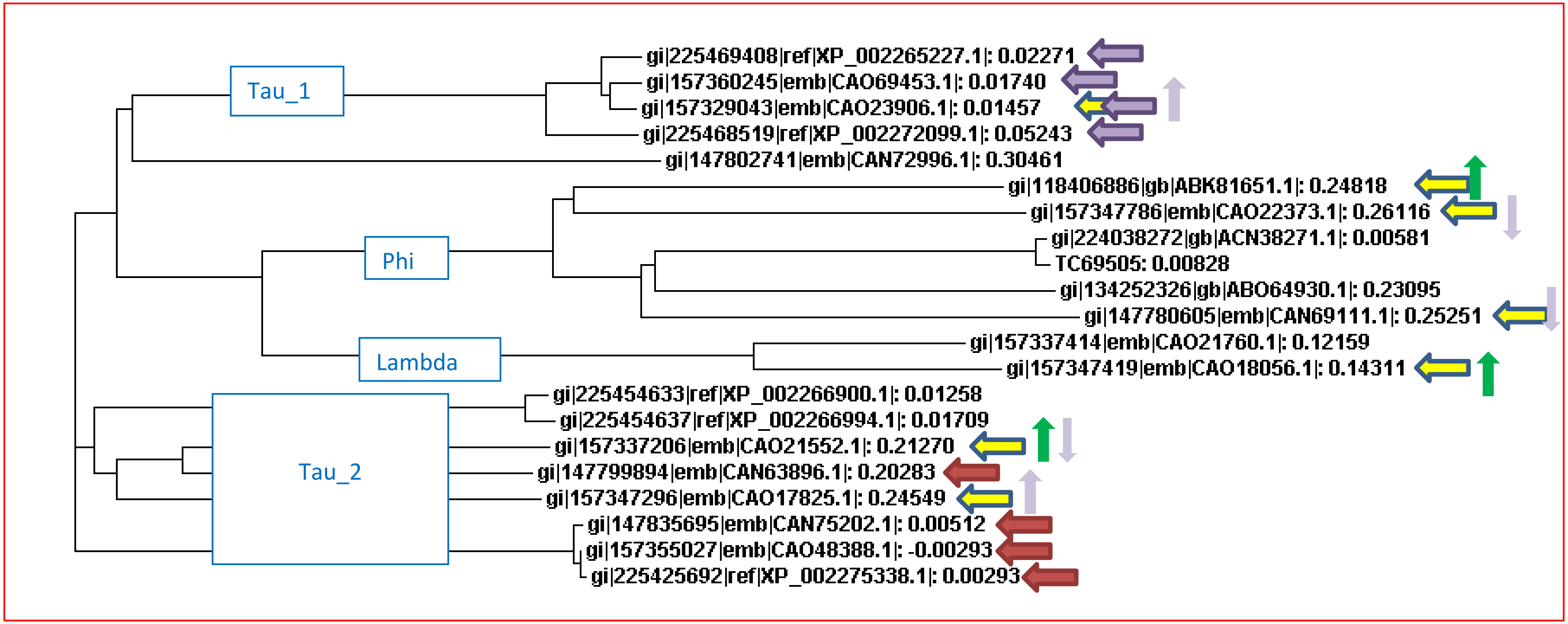
Tau_1
gi|225469408|ref|XP_002265227.1|: 0.02271
gi|157360245|emb|CAO69453.1|: 0.01740
gi|157329043|emb|CAO23906.1|: 0.01457
gi|225468519|ref|XP_002272099.1|: 0.05243
gi|147802741|emb|CAN72996.1|: 0.30461
Phi
gi|118406886|gb|ABK81651.1|: 0.24818
gi|157347786|emb|CAO22373.1|: 0.26116
gi|224038272|gb|ACN38271.1|: 0.00581
TC69505: 0.00828
gi|134252326|gb|ABO64930.1|: 0.23095
gi|147780605|emb|CAN69111.1|: 0.25251
Lambda
gi|157337414|emb|CAO21760.1|: 0.12159
gi|157347419|emb|CAO18056.1|: 0.14311
Tau_2
gi|225454633|ref|XP_002266900.1|: 0.01258
gi|225454637|ref|XP_002266994.1|: 0.01709
gi|157337206|emb|CAO21552.1|: 0.21270
gi|147799894|emb|CAN63896.1|: 0.20283
gi|157347296|emb|CAO17825.1|: 0.24549
gi|147835695|emb|CAN75202.1|: 0.00512
gi|157355027|emb|CAO48388.1|: -0.00293
gi|225425692|ref|XP_002275338.1|: 0.00293
