# Supplementary material for: iTRAQ-based protein profiling provides insights into the central metabolism changes driving grape berry development and ripening
Source: BMC Plant Biol. 2013 Oct 24;13:167. doi: 10.1186/1471-2229-13-167 (PMC4016569; doi:10.1186/1471-2229-13-167)
Supplement: Additional file 13 — Scenario of the photosynthetic machinery of both the light and dark phases during grape berry development. [file 1471-2229-13-167-S13.pdf]

**Additional Figure 13. Scenario of the photosynthetic machinery of both the light and dark phases during grape berry development.** The protein levels of regulated enzymes are shown in coloured squares, indicating the change of expression ($log_2$ ratio) for each developmental stage in relation to the 15mm stage. In sequence order (left to right), stages are displayed from FS, 4 mm, 7 mm, 15 mm, V-100, 110 g/l, and 140 g/l. Different isoforms or subunits of proteins are shown as different rows.

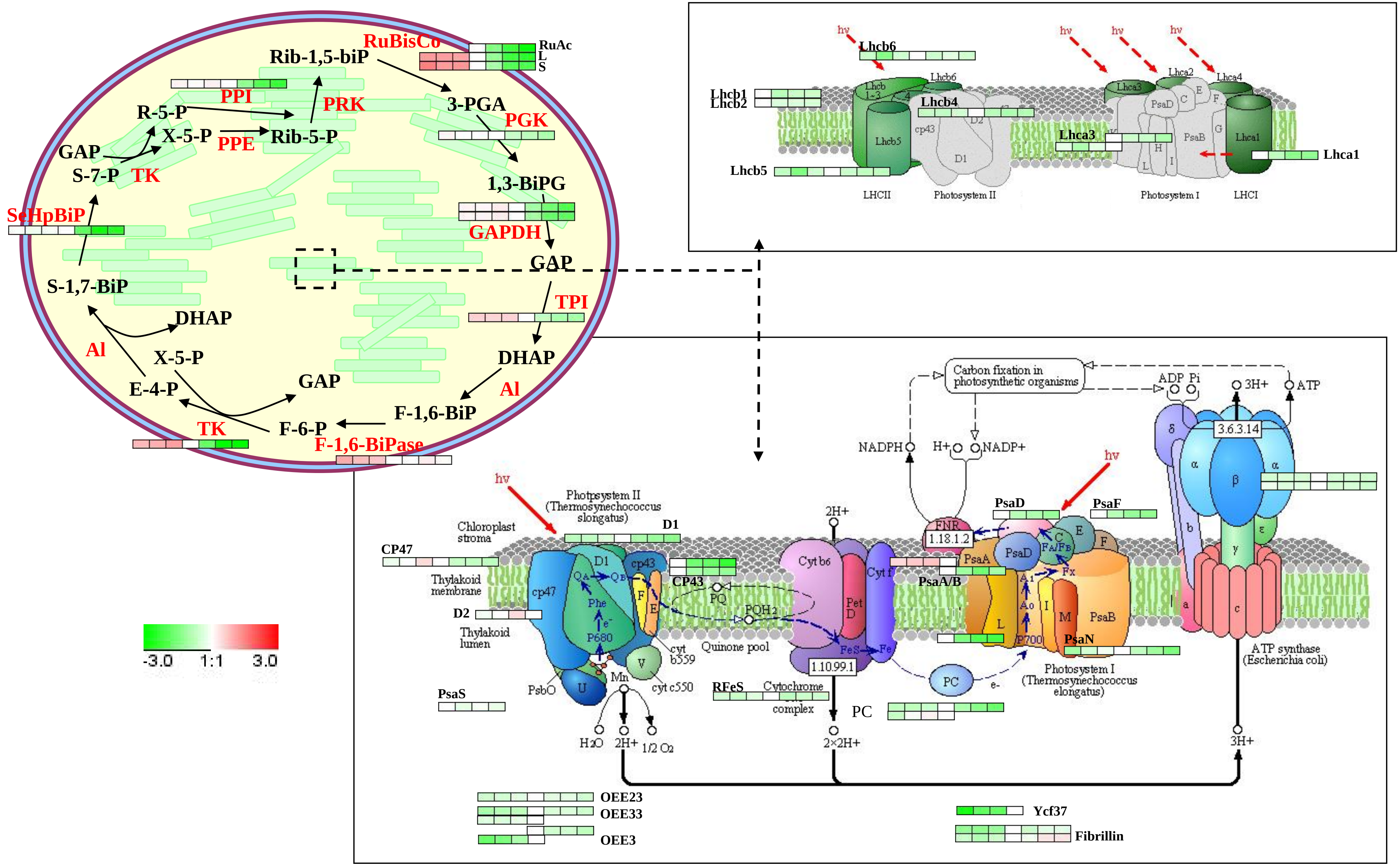

RuBisCo
RuAc
L
S
Rib-1,5-biP
3-PGA
PPI
PRK
PGK
R-5-P
X-5-P
Rib-5-P
PPE
GAP
S-7-P
TK
1,3-BiPG
SeHpBiP
GAPDH
GAP
S-1,7-BiP
TPI
DHAP
Al
X-5-P
DHAP
E-4-P
GAP
Al
F-1,6-BiP
TK
F-6-P
F-1,6-BiPase
-3.0
1:1
3.0
hv
Lhcb6
Lhcb1
Lhcb2
Lhcb4
Lhcb5
Lhca3
Lhca2
Lhca4
Lhca1
LHCII
Photosystem II
Photosystem I
LHCI
Carbon fixation in photosynthetic organisms
ADP Pi
3H+
ATP
NADPH
H+
NADP+
3.6.3.14
Photpsystem II (Thermosynechococcus slongatus)
Chloroplast stroma
D1
CP47
CP43
Thylakoid membrane
D2
Thylakoid lumen
PsaS
PsbO
Mn
cyt b559
cyt c550
Quinone pool
PQ
PQH2
Cyt b6
Pet D
Cyt f
1.10.99.1
RFeS
Cytochrome complex
PC
FNR
1.18.1.2
PsaD
PsaF
PsaA
PsaA/B
PsaB
PsaN
P700
Photosystem I (Thermosynechococcus elongatus)
ATP synthase (Escherichia coli)
H2O
2H+
1/2 O2
2H+
2×2H+
3H+
OEE23
OEE33
OEE3
Ycf37
Fibrillin
