# Supplementary material for: iTRAQ-based protein profiling provides insights into the central metabolism changes driving grape berry development and ripening
Source: BMC Plant Biol. 2013 Oct 24;13:167. doi: 10.1186/1471-2229-13-167 (PMC4016569; doi:10.1186/1471-2229-13-167)
Supplement: Additional file 8 — The GO terms enriched and sequences annotated with such terms in the up- or down-regulated subsets of proteins in each grape berry developmental stage step analyzed. [file 1471-2229-13-167-S8.zip › Additional file 8/110 to E100 down.doc]

| **Cellular compoennt** | **GO:0043232- intracellular non-membrane-bound organelle**  GO: 0043228-  **non-membrane-bound organelle** | gi\|147778228\|emb\|CAN69723.1\| **translation initiation factor (--)**  gi\|157347481\|emb\|CAO18118.1\| **elongation factor 1-expressed (0.62)**  gi\|147833057\|emb\|CAN70546.1\| **eukaryotic translation initiation factor 4 gamma (0.67)**  gi\|91701853\|gb\|ABE47678.1\| **elongation factor 1-alpha (0.67)**  gi\|157352902\|emb\|CAO44792.1\| **60s ribosomal protein l22 (0.48)**  gi\|76573307\|gb\|ABA46758.1\| **40s ribosomal protein s15a (0.65)**  gi\|147815159\|emb\|CAN72332.1**\| 40s ribosomal protein s24 (0.59)**  gi\|125555687\|gb\|EAZ01293.1\| **ribosomal proteincytosolic (0.55)**  gi\|159479900\|ref\|XP_001698024.1\| **60s ribosomal protein l21 (0.54)**  gi\|157336744\|emb\|CAO71429.1\| **60s ribosomal protein l14 (0.52)**  gi\|157335152\|emb\|CAO60982.1\| **beta-tubulin (0.64)**  gi\|157359041\|emb\|CAO66943.1\| **40s ribosomal protein s14 (0.63)**  gi\|76573325\|gb\|ABA46767.1\| **40s ribosomal protein s9(0.46)**  gi\|157348680\|emb\|CAO23572.1\| **40s ribosomal protein s2 (0.44)**  gi\|147772865\|emb\|CAN75906.1\| **60s ribosomal protein l26 (0.65)**  gi\|147858266\|emb\|CAN83920.1\| **40s ribosomal protein s19 (0.60)**  gi\|147826473\|emb\|CAN72805.1\| **40s ribosomal protein s16 (0.47)**  gi\|157354887\|emb\|CAO48233.1\| **ribosomal protein l8 (0.49)**  gi\|56202344\|dbj\|BAD73824.1\| **60s ribosomal protein l10a (0.51)**  gi\|147802483\|emb\|CAN77412.1\| **alpha tubulin 1 (0.63)**  gi\|78191412\|gb\|ABB29927.1\| **60s ribosomal protein l35 (0.55)**  gi\|5706704\|gb\|AAD47346.1\|AF112440_1 **40s ribosomal protein s26 (0.37)**  gi\|157357316\|emb\|CAO63839.1\| **ribosomal protein s4 (0.50)**  gi\|464621\|sp\|P34091.1\|RL6_MESCR **60s ribosomal protein l6 (0.51)**  gi\|82623429\|gb\|ABB87129.1\| **60s ribosomal protein l7a (0.63)**  gi\|89147796\|gb\|ABD62753.1\| **histone h4 (0.55)**  gi\|157338974\|emb\|CAO42325.1\| **high mobility group protein (0.19)** | 3.01635E-8 |
| --- | --- | --- | --- |
|  | **GO:0005843- cytosolic small ribosomal subunit (sensu Eukaryota)** | gi\|76573307\|gb\|ABA46758.1\|, gi\|157359041\|emb\|CAO66943.1\|, gi\|76573325\|gb\|ABA46767.1\|, gi\|157348680\|emb\|CAO23572.1\|, gi\|147826473\|emb\|CAN72805.1\|, gi\|157357316\|emb\|CAO63839.1\| | 3.10541E-4 |
|  | **GO:0033279- ribosomal subunit** | gi\|76573307\|gb\|ABA46758.1\|, gi\|125555687\|gb\|EAZ01293.1\|, gi\|157359041\|emb\|CAO66943.1\|, gi\|76573325\|gb\|ABA46767.1\|, gi\|157348680\|emb\|CAO23572.1\|, gi\|147772865\|emb\|CAN75906.1\|, gi\|147826473\|emb\|CAN72805.1\|, gi\|56202344\|dbj\|BAD73824.1\|, gi\|157357316\|emb\|CAO63839.1\| | 1.96788E-4 |
|  | **GO:0048492- ribulose bisphosphate carboxylase complex**  **GO:0009573- chloroplast ribulose bisphosphate carboxylase complex** | gi\|147807983\|emb\|CAN64291.1\|, gi\|11467200\|ref\|NP_043033.1\|, gi\|21950712\|gb\|AAM78591.1\| | 7.64749E-4 |
|  | **GO:0015935- small ribosomal subunit** | gi\|76573307\|gb\|ABA46758.1\|, gi\|157359041\|emb\|CAO66943.1\|, gi\|76573325\|gb\|ABA46767.1\|, gi\|157348680\|emb\|CAO23572.1\|, gi\|147826473\|emb\|CAN72805.1\|, gi\|157357316\|emb\|CAO63839.1\| | 8.65304E-4 |
|  | **GO:0005830- cytosolic ribosome (sensu Eukaryota)** | gi\|76573307\|gb\|ABA46758.1\|, gi\|125555687\|gb\|EAZ01293.1\|, gi\|157359041\|emb\|CAO66943.1\|, gi\|76573325\|gb\|ABA46767.1\|, gi\|157348680\|emb\|CAO23572.1\|, gi\|147826473\|emb\|CAN72805.1\|, gi\|157357316\|emb\|CAO63839.1\| | 4.98557E-5 |
|  | **GO:0005840- ribosome**  **GO:0030529- ribonucleoprotein complex** | gi\|147778228\|emb\|CAN69723.1\|, gi\|157347481\|emb\|CAO18118.1\|, gi\|147833057\|emb\|CAN70546.1\|, gi\|91701853\|gb\|ABE47678.1\|, gi\|157352902\|emb\|CAO44792.1\|, gi\|76573307\|gb\|ABA46758.1\|, gi\|147815159\|emb\|CAN72332.1\|, gi\|125555687\|gb\|EAZ01293.1\|, gi\|159479900\|ref\|XP_001698024.1\|, gi\|157336744\|emb\|CAO71429.1\|, gi\|157359041\|emb\|CAO66943.1\|, gi\|76573325\|gb\|ABA46767.1\|, gi\|157348680\|emb\|CAO23572.1\|, gi\|147772865\|emb\|CAN75906.1\|, gi\|147858266\|emb\|CAN83920.1\|, gi\|147826473\|emb\|CAN72805.1\|, gi\|157354887\|emb\|CAO48233.1\|, gi\|56202344\|dbj\|BAD73824.1\|, gi\|78191412\|gb\|ABB29927.1\|, gi\|5706704\|gb\|AAD47346.1\|AF112440_1, gi\|157357316\|emb\|CAO63839.1\|, gi\|464621\|sp\|P34091.1\|RL6_MESCR, gi\|82623429\|gb\|ABB87129.1\| | 2.5159E-7  4.09319E-7 |
| **Biological process** | **GO:0006996- organelle organization and biogenesis** | gi\|157352902\|emb\|CAO44792.1\|, gi\|76573307\|gb\|ABA46758.1\|, gi\|147815159\|emb\|CAN72332.1\|, gi\|125555687\|gb\|EAZ01293.1\|, gi\|159479900\|ref\|XP_001698024.1\|, gi\|157336744\|emb\|CAO71429.1\|, gi\|157335152\|emb\|CAO60982.1\|, gi\|157359041\|emb\|CAO66943.1\|, gi\|76573325\|gb\|ABA46767.1\|, gi\|157348680\|emb\|CAO23572.1\|, gi\|147772865\|emb\|CAN75906.1\|, gi\|147858266\|emb\|CAN83920.1\|, gi\|147826473\|emb\|CAN72805.1\|, gi\|157354887\|emb\|CAO48233.1\|, gi\|56202344\|dbj\|BAD73824.1\|, gi\|147802483\|emb\|CAN77412.1\|, gi\|78191412\|gb\|ABB29927.1\|, gi\|5706704\|gb\|AAD47346.1\|AF112440_1, gi\|157357316\|emb\|CAO63839.1\|, gi\|464621\|sp\|P34091.1\|RL6_MESCR, gi\|82623429\|gb\|ABB87129.1\|, gi\|89147796\|gb\|ABD62753.1\|, gi\|157338974\|emb\|CAO42325.1\| | 1.95897E-7 |
|  | **GO:0042254- ribosome biogenesis and assembly** | gi\|157352902\|emb\|CAO44792.1\|, gi\|76573307\|gb\|ABA46758.1\|, gi\|147815159\|emb\|CAN72332.1\|, gi\|125555687\|gb\|EAZ01293.1\|, gi\|159479900\|ref\|XP_001698024.1\|, gi\|157336744\|emb\|CAO71429.1\|, gi\|157359041\|emb\|CAO66943.1\|, gi\|76573325\|gb\|ABA46767.1\|, gi\|157348680\|emb\|CAO23572.1\|, gi\|147772865\|emb\|CAN75906.1\|, gi\|147858266\|emb\|CAN83920.1\|, gi\|147826473\|emb\|CAN72805.1\|, gi\|157354887\|emb\|CAO48233.1\|, gi\|56202344\|dbj\|BAD73824.1\|, gi\|78191412\|gb\|ABB29927.1\|, gi\|5706704\|gb\|AAD47346.1\|AF112440_1, gi\|157357316\|emb\|CAO63839.1\|, gi\|464621\|sp\|P34091.1\|RL6_MESCR, gi\|82623429\|gb\|ABB87129.1\| | 3.40321E-8 |
|  | **GO:0022613- ribonucleoprotein complex biogenesis and assembly** | gi\|147778228\|emb\|CAN69723.1\|, gi\|147833057\|emb\|CAN70546.1\|, gi\|157352902\|emb\|CAO44792.1\|, gi\|76573307\|gb\|ABA46758.1\|, gi\|147815159\|emb\|CAN72332.1\|, gi\|125555687\|gb\|EAZ01293.1\|, gi\|159479900\|ref\|XP_001698024.1\|, gi\|157336744\|emb\|CAO71429.1\|, gi\|157359041\|emb\|CAO66943.1\|, gi\|76573325\|gb\|ABA46767.1\|, gi\|157348680\|emb\|CAO23572.1\|, gi\|147772865\|emb\|CAN75906.1\|, gi\|147858266\|emb\|CAN83920.1\|, gi\|147826473\|emb\|CAN72805.1\|, gi\|157354887\|emb\|CAO48233.1\|, gi\|56202344\|dbj\|BAD73824.1\|, gi\|78191412\|gb\|ABB29927.1\|, gi\|5706704\|gb\|AAD47346.1\|AF112440_1, gi\|157357316\|emb\|CAO63839.1\|, gi\|464621\|sp\|P34091.1\|RL6_MESCR, gi\|82623429\|gb\|ABB87129.1\| | 8.83383E-8 |
|  | **GO:0006412- translation** | gi\|157343454\|emb\|CAO67960.1\| **phenylalanyl-trna synthetase beta-subunit (0.64)**  gi\|157350291\|emb\|CAO40229.1\| **multifunctional aminoacyl-trna ligase-like protein (0.67)**  gi\|147778228\|emb\|CAN69723.1\|, gi\|157347481\|emb\|CAO18118.1\|, gi\|147833057\|emb\|CAN70546.1\|, gi\|91701853\|gb\|ABE47678.1\|, gi\|157352902\|emb\|CAO44792.1\|, gi\|76573307\|gb\|ABA46758.1\|, gi\|147815159\|emb\|CAN72332.1\|, gi\|125555687\|gb\|EAZ01293.1\|, gi\|159479900\|ref\|XP_001698024.1\|, gi\|157336744\|emb\|CAO71429.1\|, gi\|157359041\|emb\|CAO66943.1\|, gi\|76573325\|gb\|ABA46767.1\|, gi\|157348680\|emb\|CAO23572.1\|, gi\|147772865\|emb\|CAN75906.1\|, gi\|147858266\|emb\|CAN83920.1\|, gi\|147826473\|emb\|CAN72805.1\|, gi\|157354887\|emb\|CAO48233.1\|, gi\|56202344\|dbj\|BAD73824.1\|, gi\|78191412\|gb\|ABB29927.1\|, gi\|5706704\|gb\|AAD47346.1\|AF112440_1, gi\|157357316\|emb\|CAO63839.1\|, gi\|464621\|sp\|P34091.1\|RL6_MESCR, gi\|82623429\|gb\|ABB87129.1\| | 2.55005E-7 |
|  | **GO:0043170- macromolecule metabolic process** | gi\|157348674\|emb\|CAO23566.1\| **atsip2 (arabidopsis thaliana seed imbibition 2)hydrolyzing o-glycosyl compounds (0.64)**  gi\|157353049\|emb\|CAO44939.1\| **galactose kinase (0.57)** gi\|157356623\|emb\|CAO62818.1\| **protein kinase-like protein (0.67)**  gi\|17221690\|gb\|AAL36459.1\|AF205603_1 **nadh-cytochrome b5 reductase (0.61)** gi\|159474126\|ref\|XP_001695180.1\| **ubiquitin-activating enzyme e1 (0.56)**  gi\|157341429\|emb\|CAO49336.1\| **mannose-6-phosphate isomerase (0.62)**  gi\|157348694\|emb\|CAO23586.1\| **insulin degrading enzyme (0.67)**  gi\|157342617\|emb\|CAO65329.1\| **casein kinase i (0.59)** gi\|9885272\|gb\|AAG01381.1\|AF194173_1 **alcohol dehydrogenase (0.53)**  gi\|157357936\|emb\|CAO64948.1\| **succinic semialdehyde dehydrogenase (0.68)**  gi\|157345040\|emb\|CAO71768.1\| **disproportionating enzyme (0.67)**  gi\|7271945\|gb\|AAF44702.1\|AF241524_1 **chlorophyll a b-binding protein (0.66)**  gi\|147771556\|emb\|CAN78176.1\| **endo-xyloglucan transferase-like protein (0.50)** gi\|147820236\|emb\|CAN62640.1\| **subtilisin-like protease (0.60)**  gi\|147778228\|emb\|CAN69723.1\|, gi\|157343454\|emb\|CAO67960.1\|, gi\|157329624\|emb\|CAO41063.1\|, gi\|157355254\|emb\|CAO48630.1\|, gi\|157347481\|emb\|CAO18118.1\|, gi\|147833057\|emb\|CAN70546.1\|, gi\|157340835\|emb\|CAO47640.1\|, gi\|91701853\|gb\|ABE47678.1\|, gi\|157332908\|emb\|CAO15482.1\|, gi\|157340619\|emb\|CAO47424.1\|, gi\|157350291\|emb\|CAO40229.1\|, gi\|157352902\|emb\|CAO44792.1\|, gi\|76573307\|gb\|ABA46758.1\|, gi\|147815159\|emb\|CAN72332.1\|, gi\|147770696\|emb\|CAN77927.1\|, gi\|125555687\|gb\|EAZ01293.1\|, gi\|159479900\|ref\|XP_001698024.1\|, gi\|157336744\|emb\|CAO71429.1\|, gi\|157335152\|emb\|CAO60982.1\|, gi\|157359041\|emb\|CAO66943.1\|, gi\|76573325\|gb\|ABA46767.1\|, gi\|157348680\|emb\|CAO23572.1\|, gi\|147772865\|emb\|CAN75906.1\|, gi\|147858266\|emb\|CAN83920.1\|, gi\|147826473\|emb\|CAN72805.1\|, gi\|157342473\|emb\|CAO65185.1\|, gi\|157354887\|emb\|CAO48233.1\|, gi\|56202344\|dbj\|BAD73824.1\|, gi\|147807983\|emb\|CAN64291.1\|, gi\|147802483\|emb\|CAN77412.1\|, gi\|78191412\|gb\|ABB29927.1\|, gi\|5706704\|gb\|AAD47346.1\|AF112440_1, gi\|157357316\|emb\|CAO63839.1\|, gi\|157358587\|emb\|CAO66263.1\|, gi\|77540210\|gb\|ABA86963.1\|, gi\|11467200\|ref\|NP_043033.1\|, gi\|464621\|sp\|P34091.1\|RL6_MESCR, gi\|5881134\|gb\|AAD55057.1\|, gi\|119026097\|ref\|YP_909942.1\|, gi\|82623429\|gb\|ABB87129.1\|, gi\|89147796\|gb\|ABD62753.1\|, gi\|157350105\|emb\|CAO40043.1\|, gi\|147835837\|emb\|CAN72939.1\|, gi\|157333396\|emb\|CAO17011.1\|, gi\|157338974\|emb\|CAO42325.1\|, | 0.00137113 |
|  | **GO:0009059- macromolecule biosynthetic process** | gi\|157329624\|emb\|CAO41063.1\| **phosphoenolpyruvate carboxykinase (0.65)**  gi\|147770696\|emb\|CAN77927.1\| **phosphoenolpyruvate carboxylase (0.63)**  gi\|119026097\|ref\|YP_909942.1\| **glyceraldehyde-3-phosphate dehydrogenase (0.63)**  gi\|147807983\|emb\|CAN64291.1\|, gi\|157358587\|emb\|CAO66263.1\|, gi\|77540210\|gb\|ABA86963.1\|, gi\|11467200\|ref\|NP_043033.1\|, gi\|5881134\|gb\|AAD55057.1\|, gi\|147835837\|emb\|CAN72939.1\|, gi\|157333396\|emb\|CAO17011.1\|  gi\|147778228\|emb\|CAN69723.1\|, gi\|157343454\|emb\|CAO67960.1\|, gi\|157347481\|emb\|CAO18118.1\|, gi\|147833057\|emb\|CAN70546.1\|, gi\|91701853\|gb\|ABE47678.1\|, gi\|157332908\|emb\|CAO15482.1\|, gi\|157350291\|emb\|CAO40229.1\|, gi\|157352902\|emb\|CAO44792.1\|, gi\|76573307\|gb\|ABA46758.1\|, gi\|147815159\|emb\|CAN72332.1\|, gi\|125555687\|gb\|EAZ01293.1\|, gi\|159479900\|ref\|XP_001698024.1\|, gi\|157336744\|emb\|CAO71429.1\|, gi\|157359041\|emb\|CAO66943.1\|, gi\|76573325\|gb\|ABA46767.1\|, gi\|157348680\|emb\|CAO23572.1\|, gi\|147772865\|emb\|CAN75906.1\|, gi\|147858266\|emb\|CAN83920.1\|, gi\|147826473\|emb\|CAN72805.1\|, gi\|157354887\|emb\|CAO48233.1\|, gi\|56202344\|dbj\|BAD73824.1\|, gi\|78191412\|gb\|ABB29927.1\|, gi\|5706704\|gb\|AAD47346.1\|AF112440_1, gi\|157357316\|emb\|CAO63839.1\|, gi\|464621\|sp\|P34091.1\|RL6_MESCR, gi\|82623429\|gb\|ABB87129.1\|, | 2.95193E-6 |
|  | **GO:0019253- reductive pentose-phosphate cycle**  **GO:0019685- photosynthesis, dark reaction** | gi\|147807983\|emb\|CAN64291.1\| **ribulose bisphosphatesmall subunit (0.51)**  gi\|157358587\|emb\|CAO66263.1\| **glyceraldehyde-3-phosphate dehydrogenase (0.67)**  gi\|77540210\|gb\|ABA86963.1\| **at3g26650 mlj15_5 (0.60)**  gi\|11467200\|ref\|NP_043033.1\| **ribulose--bisphosphate carboxylase oxygenase large subunit (0.48)**  gi\|5881134\|gb\|AAD55057.1\| **phosphoribulokinase precursor (0.58)**  gi\|147835837\|emb\|CAN72939.1\| **transketolase 1 (0.26)**  gi\|157333396\|emb\|CAO17011.1\| **chloroplast sedoheptulose—bisphosphatase (0.51)** | 1.12078E-5 |
|  | **GO:0015977- carbon utilization by fixation of carbon dioxide** | gi\|21950712\|gb\|AAM78591.1\| **rubisco activase (0.56)**  gi\|147770696\|emb\|CAN77927.1\|, gi\|147807983\|emb\|CAN64291.1\|, gi\|157358587\|emb\|CAO66263.1\|, gi\|77540210\|gb\|ABA86963.1\|, gi\|11467200\|ref\|NP_043033.1\|, gi\|5881134\|gb\|AAD55057.1\|, gi\|147835837\|emb\|CAN72939.1\|, gi\|157333396\|emb\|CAO17011.1\| | 3.85682E-4 |
|  | **GO:0009058- biosynthetic process** | gi\|157332908\|emb\|CAO15482.1\| **phosphoenolpyruvate carboxykinase (0.67)**  gi\|157357156\|emb\|CAO63650.1\|**hydroxymethylglutaryl coenzyme a synthase (0.62)**  gi\|157348803\|emb\|CAO23804.1\| **nadh dehydrogenase (0.61)**  gi\|157336586\|emb\|CAO71271.1\| **at5g28840 f7p1_20 (0.61)**  gi\|157342473\|emb\|CAO65185.1\| **sucrose synthase (0.56)** gi\|157354887\|emb\|CAO48233.1\| **ribosomal protein l8 (0.49)**  gi\|11467200\|ref\|NP_043033.1\| **ribulose--bisphosphate carboxylase oxygenase large subunit (0.48)** gi\|1705761\|sp\|P51117.1\|CFI1_VITVI **chalcone isomerase (0.65)**  gi\|157348402\|emb\|CAO23189.1\| **phosphoglycerate dehydrogenase (0.32)**  gi\|147778228\|emb\|CAN69723.1\|, gi\|157343454\|emb\|CAO67960.1\|, gi\|157329624\|emb\|CAO41063.1\|, gi\|157347481\|emb\|CAO18118.1\|, gi\|147833057\|emb\|CAN70546.1\|, gi\|91701853\|gb\|ABE47678.1\|, gi\|157350291\|emb\|CAO40229.1\|, gi\|157352902\|emb\|CAO44792.1\|, gi\|76573307\|gb\|ABA46758.1\|, gi\|147815159\|emb\|CAN72332.1\|, gi\|147770696\|emb\|CAN77927.1\|, gi\|125555687\|gb\|EAZ01293.1\|, gi\|159479900\|ref\|XP_001698024.1\|, gi\|157336744\|emb\|CAO71429.1\|, gi\|157359041\|emb\|CAO66943.1\|, gi\|76573325\|gb\|ABA46767.1\|, gi\|157348680\|emb\|CAO23572.1\|, gi\|147772865\|emb\|CAN75906.1\|, gi\|147858266\|emb\|CAN83920.1\|, gi\|147826473\|emb\|CAN72805.1\|, gi\|56202344\|dbj\|BAD73824.1\|, gi\|147807983\|emb\|CAN64291.1\|, gi\|78191412\|gb\|ABB29927.1\|, gi\|5706704\|gb\|AAD47346.1\|AF112440_1, gi\|157357316\|emb\|CAO63839.1\|, gi\|157358587\|emb\|CAO66263.1\|, gi\|77540210\|gb\|ABA86963.1\|, gi\|464621\|sp\|P34091.1\|RL6_MESCR, gi\|5881134\|gb\|AAD55057.1\|, gi\|119026097\|ref\|YP_909942.1\|, gi\|82623429\|gb\|ABB87129.1\|, gi\|147835837\|emb\|CAN72939.1\|, gi\|157333396\|emb\|CAO17011.1\|, | 3.70561E-4 |
|  | **GO:0016043- cellular component organization and biogenesis** | gi\|157355254\|emb\|CAO48630.1\| **importin beta (0.59)**  gi\|157340619\|emb\|CAO47424.1\| **transportin 1 (0.61)**  gi\|147861641\|emb\|CAN79299.1\| **dwf1 (diminuto 1) catalytic (0.63)**  gi\|157343968\|emb\|CAO68901.1\| **component of the exocyst complex (0.54)**  gi\|157350105\|emb\|CAO40043.1\| **pectinesterase family protein (0.56)**  gi\|147778228\|emb\|CAN69723.1\|, gi\|147833057\|emb\|CAN70546.1\|, gi\|157340835\|emb\|CAO47640.1\|, gi\|157352902\|emb\|CAO44792.1\|, gi\|76573307\|gb\|ABA46758.1\|, gi\|147815159\|emb\|CAN72332.1\|, gi\|125555687\|gb\|EAZ01293.1\|, gi\|159479900\|ref\|XP_001698024.1\|, gi\|157336744\|emb\|CAO71429.1\|, gi\|157335152\|emb\|CAO60982.1\|, gi\|157359041\|emb\|CAO66943.1\|, gi\|76573325\|gb\|ABA46767.1\|, gi\|157348680\|emb\|CAO23572.1\|, gi\|147772865\|emb\|CAN75906.1\|, gi\|147858266\|emb\|CAN83920.1\|, gi\|147826473\|emb\|CAN72805.1\|, gi\|157354887\|emb\|CAO48233.1\|, gi\|56202344\|dbj\|BAD73824.1\|, gi\|147802483\|emb\|CAN77412.1\|, gi\|78191412\|gb\|ABB29927.1\|, gi\|5706704\|gb\|AAD47346.1\|AF112440_1, gi\|157357316\|emb\|CAO63839.1\|, gi\|464621\|sp\|P34091.1\|RL6_MESCR, gi\|82623429\|gb\|ABB87129.1\|, gi\|89147796\|gb\|ABD62753.1\|, gi\|157338974\|emb\|CAO42325.1\| | 2.30802E-5 |
| **Molecular function** | **GO:0003735- structural constituent of ribosome** | gi\|157352902\|emb\|CAO44792.1\|, gi\|76573307\|gb\|ABA46758.1\|, gi\|147815159\|emb\|CAN72332.1\|, gi\|125555687\|gb\|EAZ01293.1\|, gi\|159479900\|ref\|XP_001698024.1\|, gi\|157336744\|emb\|CAO71429.1\|, gi\|157359041\|emb\|CAO66943.1\|, gi\|76573325\|gb\|ABA46767.1\|, gi\|157348680\|emb\|CAO23572.1\|, gi\|147772865\|emb\|CAN75906.1\|, gi\|147858266\|emb\|CAN83920.1\|, gi\|147826473\|emb\|CAN72805.1\|, gi\|157354887\|emb\|CAO48233.1\|, gi\|56202344\|dbj\|BAD73824.1\|, gi\|78191412\|gb\|ABB29927.1\|, gi\|5706704\|gb\|AAD47346.1\|AF112440_1, gi\|157357316\|emb\|CAO63839.1\|, gi\|464621\|sp\|P34091.1\|RL6_MESCR, gi\|82623429\|gb\|ABB87129.1\| | 3.40321E-8 |
|  | **GO:0005198- structural molecule activity** | gi\|157340835\|emb\|CAO47640.1\| **coatomer protein gamma 2-subunit (0.67)**  gi\|157352902\|emb\|CAO44792.1\|, gi\|76573307\|gb\|ABA46758.1\|, gi\|147815159\|emb\|CAN72332.1\|, gi\|125555687\|gb\|EAZ01293.1\|, gi\|159479900\|ref\|XP_001698024.1\|, gi\|157336744\|emb\|CAO71429.1\|, gi\|157335152\|emb\|CAO60982.1\|, gi\|157359041\|emb\|CAO66943.1\|, gi\|76573325\|gb\|ABA46767.1\|, gi\|157348680\|emb\|CAO23572.1\|, gi\|147772865\|emb\|CAN75906.1\|, gi\|147858266\|emb\|CAN83920.1\|, gi\|147826473\|emb\|CAN72805.1\|, gi\|157354887\|emb\|CAO48233.1\|, gi\|56202344\|dbj\|BAD73824.1\|, gi\|147802483\|emb\|CAN77412.1\|, gi\|78191412\|gb\|ABB29927.1\|, gi\|5706704\|gb\|AAD47346.1\|AF112440_1, gi\|157357316\|emb\|CAO63839.1\|, gi\|464621\|sp\|P34091.1\|RL6_MESCR, gi\|82623429\|gb\|ABB87129.1\| | 3.81573E-8 |
|  | **GO:0016984- ribulose-bisphosphate carboxylase activity** | gi\|147807983\|emb\|CAN64291.1\|, gi\|11467200\|ref\|NP_043033.1\|, gi\|21950712\|gb\|AAM78591.1\| | 7.64749E-4 |
