# Supplementary material for: iTRAQ-based protein profiling provides insights into the central metabolism changes driving grape berry development and ripening
Source: BMC Plant Biol. 2013 Oct 24;13:167. doi: 10.1186/1471-2229-13-167 (PMC4016569; doi:10.1186/1471-2229-13-167)
Supplement: Additional file 8 — The GO terms enriched and sequences annotated with such terms in the up- or down-regulated subsets of proteins in each grape berry developmental stage step analyzed. [file 1471-2229-13-167-S8.zip › Additional file 8/110 to E100 up.doc]

| **Cellular compoent** | **GO:0016272- prefoldin complex** | gi\|47496854\|dbj\|BAD19818.1\| **prefoldin 1 (1.89)**  gi\|157336490\|emb\|CAO71175.1\| **prefoldin subunit 2 (1.52)**  gi\|157360839\|emb\|CAO70650.1\| **prefoldin subunit 4 (1.50)**  gi\|126309694\|ref\|XP_001369480.1\| **prefoldin subunit 6 (1.50)** | 1.0686E-5 |
| --- | --- | --- | --- |
| **Biological process** | **GO: 0006030- chitin metabolic process**  **GO:0006043- glucosamine catabolic process**  **GO:0006032- chitin catabolic process**  **GO:0006044- N-acetylglucosamine metabolic process**  **GO:0006041- glucosamine metabolic process**  **GO:0030247- polysaccharide binding**  **GO:0006046- N-acetylglucosamine catabolic process**  **GO:0016998- cell wall catabolic process**  **GO:0000272- polysaccharide catabolic process**  **GO:0010382- cell wall metabolic process**  **GO:0046348-**  **amino sugar catabolic process**  **GO:0044247-**  **cellular polysaccharide catabolic process** | gi\|116329\|sp\|P29022.1\|CHIA_MAIZE **chitinase (1.63)**  gi\|3511147\|gb\|AAC33732.1\| **pr-4 type protein (1.97)**  gi\|157353734\|emb\|CAO46266.1\| **class iv chitinase (1.65)** | 7.34282E-4 |
|  | **GO:0005976- polysaccharide metabolic process** | gi\|116329\|sp\|P29022.1\|CHIA_MAIZE, gi\|3511147\|gb\|AAC33732.1\|, gi\|157353734\|emb\|CAO46266.1\|,  gi\|157348466\|emb\|CAO23358.1\| **beta 1-3 glucanase (1.68)** | 7.34282E-4 |
| **Molecular function** | **GO:0004197- cysteine-type endopeptidase activity**  **GO:0008234- cysteine-type peptidase activity** | gi\|218183\|dbj\|BAA14403.1\| **cysteine protease component of protease-inhibitor complex (1.98)**  gi\|147790682\|emb\|CAN61026.1\| **cysteine protease component of protease-inhibitor complex (1.75)**  gi\|157341042\|emb\|CAO47847.1\| **cysteine proteinase (1.95)**  gi\|157350311\|emb\|CAO40249.1\| **cathepsin b-like cysteine protease (1.56)** | 1.46543E-4  3.26966E-4 |
|  | **GO:0008061- chitin binding**  **GO:0004568- chitinase activity** | gi\|116329\|sp\|P29022.1\|CHIA_MAIZE, gi\|3511147\|gb\|AAC33732.1\|, gi\|157353734\|emb\|CAO46266.1\| | 7.34282E-4 |
|  | **GO:0004623- phospholipase A2 activity** | gi\|218183\|dbj\|BAA14403.1\|,  gi\|147790682\|emb\|CAN61026.1\|,  gi\|157341042\|emb\|CAO47847.1\| | 7.34282E-4 |
|  | **GO:0001871- pattern binding** | gi\|116329\|sp\|P29022.1\|CHIA_MAIZE, gi\|3511147\|gb\|AAC33732.1\|, gi\|157353734\|emb\|CAO46266.1\| | 7.34282E-4 |
