# Supplementary material for: iTRAQ-based protein profiling provides insights into the central metabolism changes driving grape berry development and ripening
Source: BMC Plant Biol. 2013 Oct 24;13:167. doi: 10.1186/1471-2229-13-167 (PMC4016569; doi:10.1186/1471-2229-13-167)
Supplement: Additional file 8 — The GO terms enriched and sequences annotated with such terms in the up- or down-regulated subsets of proteins in each grape berry developmental stage step analyzed. [file 1471-2229-13-167-S8.zip › Additional file 8/140 to 110 up.doc]

| **Molecular function** | **GO:0045735- nutrient reservoir activity** | 3.92405E-5 | gi\|157350579\|emb\|CAO40628.1\| **12s cruciferin seed storage protein (4.95)**  gi\|157347968\|emb\|CAO22555.1\| **vicilin precursor (132.31)** gi\|157350582\|emb\|CAO40631.1\| **12s cruciferin seed storage protein (2.71)** |
| --- | --- | --- | --- |

**140 to 110 - up**
