# Supplementary material for: iTRAQ-based protein profiling provides insights into the central metabolism changes driving grape berry development and ripening
Source: BMC Plant Biol. 2013 Oct 24;13:167. doi: 10.1186/1471-2229-13-167 (PMC4016569; doi:10.1186/1471-2229-13-167)
Supplement: Additional file 8 — The GO terms enriched and sequences annotated with such terms in the up- or down-regulated subsets of proteins in each grape berry developmental stage step analyzed. [file 1471-2229-13-167-S8.zip › Additional file 8/15mm E100 down.doc]

| **Cellular compoent** | **GO:0044436- thylakoid part** | gi\|157329265\|emb\|CAO38811.1\|, gi\|81176302\|ref\|YP_398379.1\|, gi\|147804704\|emb\|CAN71620.1\|, gi\|157347388\|emb\|CAO17917.1\|, gi\|147770824\|emb\|CAN76428.1\|, gi\|157329311\|emb\|CAO39792.1\|, \|, gi\|147826727\|emb\|CAN66108.1\|,gi\|7271945\|gb\|AAF44702.1\|AF241524_1, gi\|157353870\|emb\|CAO46405.1\|,  gi\|157359015\|emb\|CAO66917.1\|, gi\|157346115\|emb\|CAO15812.1\|, gi\|157354460\|emb\|CAO47086.1\|, gi\|147800882\|emb\|CAN62373.1\|, gi\|115470052\|ref\|NP_001058625.1\|, gi\|157350250\|emb\|CAO40188.1\|, gi\|77744799\|gb\|ABB02355.1\|, gi\|115531915\|ref\|YP_784071.1\|, gi\|84620802\|gb\|ABC59515.1\|, gi\|5881134\|gb\|AAD55057.1\|, gi\|157344623\|emb\|CAO70006.1\|, gi\|47169444\|pdb\|1VCR\|A, gi\|139387445\|ref\|YP_001122799.1\|, gi\|74027097\|gb\|AAZ94647.1\|, gi\|147835837\|emb\|CAN72939.1\|  gi\|115604959\|ref\|YP_784412.1 **photosystem ii cp47 chlorophyll apoprotein (0.63)**  gi\|157357659\|emb\|CAO64639.1\| **h+-transporting atp synthase-like protein (0.63)**  gi\|157342483\|emb\|CAO65195.1\| **peroxiredoxin q (0.48)** | 2.79152E-11 |
| --- | --- | --- | --- |
|  | **GO:0009579- thylakoid** | gi\|157329265\|emb\|CAO38811.1\|, gi\|81176302\|ref\|YP_398379.1\|, gi\|147804704\|emb\|CAN71620.1\|, gi\|157347388\|emb\|CAO17917.1\|, gi\|147770824\|emb\|CAN76428.1\|, gi\|157329311\|emb\|CAO39792.1\|, gi\|115604959\|ref\|YP_784412.1\|, gi\|157357659\|emb\|CAO64639.1\|, gi\|7271945\|gb\|AAF44702.1\|AF241524_1, gi\|147826727\|emb\|CAN66108.1\|, gi\|157353870\|emb\|CAO46405.1\|, gi\|157359015\|emb\|CAO66917.1\|, gi\|157346115\|emb\|CAO15812.1\|, gi\|157354460\|emb\|CAO47086.1\|, gi\|147800882\|emb\|CAN62373.1\|, gi\|115470052\|ref\|NP_001058625.1\|, gi\|157350250\|emb\|CAO40188.1\|, gi\|77744799\|gb\|ABB02355.1\|, gi\|115531915\|ref\|YP_784071.1\|, \|, gi\|84620802\|gb\|ABC59515.1\|, gi\|5881134\|gb\|AAD55057.1\|, gi\|157344623\|emb\|CAO70006.1\|, gi\|47169444\|pdb\|1VCR\|A, gi\|139387445\|ref\|YP_001122799.1\|, gi\|74027097\|gb\|AAZ94647.1\|, gi\|147835837\|emb\|CAN72939.1\|  gi\|157336150\|emb\|CAO61980.1\| **chlorophyll a b binding protein (0.51)** | 9.34499E-11 |
|  | **GO:0009521- photosystem** | gi\|157329265\|emb\|CAO38811.1\|, gi\|81176302\|ref\|YP_398379.1\|, gi\|147804704\|emb\|CAN71620.1\|, gi\|157347388\|emb\|CAO17917.1\|, gi\|147770824\|emb\|CAN76428.1\|, gi\|115604959\|ref\|YP_784412.1\|, gi\|7271945\|gb\|AAF44702.1\|AF241524_1, gi\|147826727\|emb\|CAN66108.1\|, gi\|157353870\|emb\|CAO46405.1\|, gi\|157359015\|emb\|CAO66917.1\|, gi\|157354460\|emb\|CAO47086.1\|, gi\|147800882\|emb\|CAN62373.1\|, gi\|157350250\|emb\|CAO40188.1\|, gi\|77744799\|gb\|ABB02355.1\|, gi\|115531915\|ref\|YP_784071.1\|, gi\|84620802\|gb\|ABC59515.1\|, gi\|157344623\|emb\|CAO70006.1\|, gi\|47169444\|pdb\|1VCR\|A, gi\|139387445\|ref\|YP_001122799.1\|, gi\|74027097\|gb\|AAZ94647.1\| | 8.44618E-11 |
|  | **GO:0044422- organelle part**  **GO:0044446- intracellular organelle part** | gi\|157357936\|emb\|CAO64948.1\| **succinic semialdehyde dehydrogenase (0.66)**  gi\|157339714\|emb\|CAO44255.1 **synaptobrevin-like protein (0.65)**  gi\|147863982\|emb\|CAN81107.1\| **adenylate translocator (0.62)**  gi\|147781068\|emb\|CAN68131.1\| **ctr1-like protein kinase (0.43)**  gi\|11467200\|ref\|NP_043033.1\| **ribulose--bisphosphate carboxylase oxygenase large subunit (0.43)**  gi\|5881134\|gb\|AAD55057.1\| **phosphoribulokinase precursor (0.41)**  gi\|157338782\|emb\|CAO42133.1\| **at1g68560 f24j5_10 (0.38)**  gi\|157344623\|emb\|CAO70006.1\| **chlorophyll a b-binding protein (0.37)**  gi\|21950712\|gb\|AAM78591.1\| **rubisco activase (0.36)**  gi\|47169444\|pdb\|1VCR\|A **chlorophyll a b binding protein (0.35)**  gi\|139387445\|ref\|YP_001122799.1\| **photosystem i p700 apoprotein a2 (0.34)**  gi\|89147796\|gb\|ABD62753.1\| **histone h4 (0.32)**  gi\|74027097\|gb\|AAZ94647.1\| **photosystem ii cp43 chlorophyll apoprotein (0.31)**  gi\|147835837\|emb\|CAN72939.1\| **transketolase 1 (0.28)**  gi\|563329\|emb\|CAA57778.1\| **histone h2b (0.22)**  gi\|157338974\|emb\|CAO42325.1\| **high mobility group protein (0.17)**  gi\|147860809\|emb\|CAN81065.1\| **heat shock protein 70 (0.59)**  gi\|8699606\|gb\|AAF78758.1\|AF271661_1 **delta tonoplast intrinsic protein (0.58)**  gi\|3115108\|emb\|CAA11757.1\| **uncoupling protein (0.58)**  gi\|57013892\|ref\|YP_173366.1\| **apocytochrome b (0.53)**  gi\|157337397\|emb\|CAO21743.1\| **eca4_arathcalcium-transporting atpaseendoplasmic reticulum-type (0.52)**  gi\|125545056\|gb\|EAY91195.1\| **plastid division protein (0.48)**  gi\|157357316\|emb\|CAO63839.1\| **ribosomal protein s4 (0.48)**  gi\|77540210\|gb\|ABA86963.1\| **at3g26650 mlj15_5 (0.47)**  gi\|84620802\|gb\|ABC59515.1\| **chloroplast photosystem ii 22 kda component (0.46)**  gi\|81076111\|gb\|ABB55387.1\| **adenine nucleotide translocator (0.46)**  gi\|157329265\|emb\|CAO38811.1\|, gi\|81176302\|ref\|YP_398379.1\|, gi\| gi\|157339977\|emb\|CAO45654.1\|, 125555687\|gb\|EAZ01293.1\|, gi\|157359909\|emb\|CAO68645.1\|, gi\|147804704\|emb\|CAN71620.1\|, gi\|157347388\|emb\|CAO17917.1\|, gi\|147789484\|emb\|CAN71757.1\|, \|, gi\|147770824\|emb\|CAN76428.1\|, gi\|157329311\|emb\|CAO39792.1\|, gi\|115604959\|ref\|YP_784412.1\|, gi\|157357659\|emb\|CAO64639.1\|, gi\|157335152\|emb\|CAO60982.1\|, gi\|157359041\|emb\|CAO66943.1\|, gi\|76573325\|gb\|ABA46767.1\|, gi\|157336613\|emb\|CAO71298.1\|, gi\|56199526\|gb\|AAV84252.1\|, gi\|7271945\|gb\|AAF44702.1\|AF241524_1, gi\|157348680\|emb\|CAO23572.1\|, gi\|147826727\|emb\|CAN66108.1\|, gi\|157353870\|emb\|CAO46405.1\|, gi\|147772865\|emb\|CAN75906.1\|, gi\|147826473\|emb\|CAN72805.1\|, gi\|157345457\|emb\|CAO14755.1\|, gi\|157359015\|emb\|CAO66917.1\|, gi\|157346115\|emb\|CAO15812.1\|, gi\|66773102\|ref\|NP_001019593.1\|, gi\|157347411\|emb\|CAO18048.1\|, gi\|157354460\|emb\|CAO47086.1\|, gi\|147800882\|emb\|CAN62373.1\|, gi\|115470052\|ref\|NP_001058625.1\|, gi\|56202344\|dbj\|BAD73824.1\|, gi\|157350250\|emb\|CAO40188.1\|, gi\|147807983\|emb\|CAN64291.1\|, gi\|147802483\|emb\|CAN77412.1\|, gi\|77744799\|gb\|ABB02355.1\|, gi\|115531915\|ref\|YP_784071.1\|, gi\|157342483\|emb\|CAO65195.1\|, gi\|157360345\|emb\|CAO69559.1\|, gi\|157352918\|emb\|CAO44808.1\|, | 6.25451E-8 |
|  | **GO:0033279- ribosomal subunit** | gi\|157339977\|emb\|CAO45654.1\|, gi\|125555687\|gb\|EAZ01293.1\|, gi\|157359909\|emb\|CAO68645.1\|, gi\|147789484\|emb\|CAN71757.1\|, gi\|157359041\|emb\|CAO66943.1\|, gi\|76573325\|gb\|ABA46767.1\|, gi\|157336613\|emb\|CAO71298.1\|, gi\|56199526\|gb\|AAV84252.1\|, gi\|157348680\|emb\|CAO23572.1\|, gi\|147772865\|emb\|CAN75906.1\|, gi\|147826473\|emb\|CAN72805.1\|, gi\|157345457\|emb\|CAO14755.1\|, gi\|157347411\|emb\|CAO18048.1\|, gi\|56202344\|dbj\|BAD73824.1\|, gi\|157357316\|emb\|CAO63839.1\|, gi\|157360345\|emb\|CAO69559.1\|, gi\|157352918\|emb\|CAO44808.1\| | 1.54165E-7 |
|  | **GO:0009522- photosystem I** | gi\|81176302\|ref\|YP_398379.1\|, gi\|157347388\|emb\|CAO17917.1\|, gi\|7271945\|gb\|AAF44702.1\|AF241524_1, gi\|157353870\|emb\|CAO46405.1\|, gi\|157359015\|emb\|CAO66917.1\|, gi\|157354460\|emb\|CAO47086.1\|, gi\|147800882\|emb\|CAN62373.1\|, gi\|157350250\|emb\|CAO40188.1\|, gi\|157344623\|emb\|CAO70006.1\|, gi\|47169444\|pdb\|1VCR\|A, gi\|139387445\|ref\|YP_001122799.1\| | 8.0611E-7 |
|  | **GO:0009523**- **photosystem II** | gi\|157329265\|emb\|CAO38811.1\|, gi\|147804704\|emb\|CAN71620.1\|, gi\|157347388\|emb\|CAO17917.1\|, gi\|147770824\|emb\|CAN76428.1\|, gi\|7271945\|gb\|AAF44702.1\|AF241524_1, gi\|147826727\|emb\|CAN66108.1\|, gi\|157359015\|emb\|CAO66917.1\|, gi\|157350250\|emb\|CAO40188.1\|, gi\|77744799\|gb\|ABB02355.1\|, gi\|115531915\|ref\|YP_784071.1\|, gi\|84620802\|gb\|ABC59515.1\|, gi\|157344623\|emb\|CAO70006.1\|, gi\|47169444\|pdb\|1VCR\|A, gi\|74027097\|gb\|AAZ94647.1\| | 4.54464E-7 |
|  | **GO:0005830- cytosolic ribosome (sensu Eukaryota)** | gi\|125555687\|gb\|EAZ01293.1\|, gi\|157359909\|emb\|CAO68645.1\|, gi\|147789484\|emb\|CAN71757.1\|, gi\|157359041\|emb\|CAO66943.1\|, gi\|76573325\|gb\|ABA46767.1\|, gi\|157336613\|emb\|CAO71298.1\|, gi\|56199526\|gb\|AAV84252.1\|, gi\|157348680\|emb\|CAO23572.1\|, gi\|147826473\|emb\|CAN72805.1\|, gi\|157357316\|emb\|CAO63839.1\| | 1.285E-5 |
|  | **GO:0005843- cytosolic small ribosomal subunit (sensu Eukaryota)** | gi\|157359909\|emb\|CAO68645.1\|, gi\|147789484\|emb\|CAN71757.1\|, gi\|157359041\|emb\|CAO66943.1\|, gi\|76573325\|gb\|ABA46767.1\|, gi\|157336613\|emb\|CAO71298.1\|, gi\|56199526\|gb\|AAV84252.1\|, gi\|157348680\|emb\|CAO23572.1\|, gi\|147826473\|emb\|CAN72805.1\|, gi\|157357316\|emb\|CAO63839.1\| | 5.18482E-5 |
|  | **GO:0044435- plastid part** | gi\|81176302\|ref\|YP_398379.1\|, gi\|147770824\|emb\|CAN76428.1\|, gi\|157329311\|emb\|CAO39792.1\|, gi\|157357659\|emb\|CAO64639.1\|, gi\|147860809\|emb\|CAN81065.1\|, gi\|157345457\|emb\|CAO14755.1\|, gi\|157346115\|emb\|CAO15812.1\|, gi\|157354460\|emb\|CAO47086.1\|, gi\|115470052\|ref\|NP_001058625.1\|, gi\|147807983\|emb\|CAN64291.1\|, gi\|125545056\|gb\|EAY91195.1\|, gi\|157342483\|emb\|CAO65195.1\|, gi\|77540210\|gb\|ABA86963.1\|, gi\|11467200\|ref\|NP_043033.1\|, gi\|5881134\|gb\|AAD55057.1\|, gi\|21950712\|gb\|AAM78591.1\|, gi\|147835837\|emb\|CAN72939.1\| | 5.89019E-5 |
|  | **GO:0016021- integral to membrane**  **GO:0031224- intrinsic to membrane** | gi\|147861641\|emb\|CAN79299.1\| **dwf1 (diminuto 1) catalytic (0.68)**  gi\|157347500\|emb\|CAO18137.1\| **syntaxin 71-like protein (0.59)** gi\|8886718\|gb\|AAF80556.1\|AF188843_1 **aquaporin (0.58)** gi\|157339714\|emb\|CAO44255.1\| **synaptobrevin-like protein (0.65)** gi\|8071628\|gb\|AAF71820.1\|AF141900_1 **plasma membrane intrinsic protein (0.40)**  gi\|416664\|sp\|Q03194.1\|PMA4_NICPL **plasma membrane h+-atpase (0.36)**  gi\|7378771\|emb\|CAB85495.1\| **plasma membrane h+ atpase (0.35)**  gi\|147804704\|emb\|CAN71620.1\|, gi\|157347388\|emb\|CAO17917.1\|, gi\|147863982\|emb\|CAN81107.1\|, gi\|7271945\|gb\|AAF44702.1\|AF241524_1, gi\|147826727\|emb\|CAN66108.1\|, gi\|8699606\|gb\|AAF78758.1\|AF271661_1, gi\|3115108\|emb\|CAA11757.1\|, gi\|157359015\|emb\|CAO66917.1\|, gi\|115470052\|ref\|NP_001058625.1\|, gi\|57013892\|ref\|YP_173366.1\|, gi\|157337397\|emb\|CAO21743.1\|, gi\|157350250\|emb\|CAO40188.1\|, gi\|77744799\|gb\|ABB02355.1\|, gi\|115531915\|ref\|YP_784071.1\|, gi\|84620802\|gb\|ABC59515.1\|, gi\|81076111\|gb\|ABB55387.1\|, gi\|157344623\|emb\|CAO70006.1\|, gi\|47169444\|pdb\|1VCR\|A, gi\|139387445\|ref\|YP_001122799.1\|, gi\|74027097\|gb\|AAZ94647.1\| | 7.28992E-5  9.88631E-5 |
|  | **GO:0043232- intracellular non-membrane-bound organelle**  **GO:0043228- non-membrane-bound organelle** | gi\|157339977\|emb\|CAO45654.1\|, gi\|125555687\|gb\|EAZ01293.1\|, gi\|157359909\|emb\|CAO68645.1\|, gi\|159479900\|ref\|XP_001698024.1\|, gi\|147835114\|emb\|CAN67946.1\|, gi\|157336744\|emb\|CAO71429.1\|, gi\|147789484\|emb\|CAN71757.1\|, gi\|61807078\|gb\|AAX55706.1\|, gi\|157335152\|emb\|CAO60982.1\|, gi\|157359041\|emb\|CAO66943.1\|, gi\|76573325\|gb\|ABA46767.1\|, gi\|157336613\|emb\|CAO71298.1\|, gi\|56199526\|gb\|AAV84252.1\|, gi\|157348680\|emb\|CAO23572.1\|, gi\|59800134\|gb\|AAX07420.1\|, gi\|147772865\|emb\|CAN75906.1\|, gi\|147858266\|emb\|CAN83920.1\|, gi\|147826473\|emb\|CAN72805.1\|, gi\|157345457\|emb\|CAO14755.1\|, gi\|157357599\|emb\|CAO64572.1\|, gi\|66773102\|ref\|NP_001019593.1\|, gi\|157347411\|emb\|CAO18048.1\|, gi\|157354887\|emb\|CAO48233.1\|, gi\|56202344\|dbj\|BAD73824.1\|, gi\|147802483\|emb\|CAN77412.1\|, gi\|78191412\|gb\|ABB29927.1\|, gi\|5706704\|gb\|AAD47346.1\|AF112440_1, gi\|157357316\|emb\|CAO63839.1\|, gi\|157360345\|emb\|CAO69559.1\|, gi\|157352918\|emb\|CAO44808.1\|, gi\|464621\|sp\|P34091.1\|RL6_MESCR, gi\|82623429\|gb\|ABB87129.1\|, gi\|89147796\|gb\|ABD62753.1\|, gi\|563329\|emb\|CAA57778.1\|, gi\|157338974\|emb\|CAO42325.1\| | 1.31345E-4 |
|  | **GO:0015934- large ribosomal subunit** | gi\|157339977\|emb\|CAO45654.1\|, gi\|125555687\|gb\|EAZ01293.1\|, gi\|147772865\|emb\|CAN75906.1\|, gi\|157345457\|emb\|CAO14755.1\|, gi\|157347411\|emb\|CAO18048.1\|, gi\|56202344\|dbj\|BAD73824.1\|, gi\|157360345\|emb\|CAO69559.1\|, gi\|157352918\|emb\|CAO44808.1\| | 2.03423E-4 |
|  | **GO:0044434- chloroplast part** | gi\|81176302\|ref\|YP_398379.1\|, gi\|147770824\|emb\|CAN76428.1\|, gi\|157329311\|emb\|CAO39792.1\|, gi\|157357659\|emb\|CAO64639.1\|, gi\|157346115\|emb\|CAO15812.1\|, gi\|157354460\|emb\|CAO47086.1\|, gi\|115470052\|ref\|NP_001058625.1\|, gi\|147807983\|emb\|CAN64291.1\|, gi\|125545056\|gb\|EAY91195.1\|, gi\|157342483\|emb\|CAO65195.1\|, gi\|77540210\|gb\|ABA86963.1\|, gi\|11467200\|ref\|NP_043033.1\|, gi\|5881134\|gb\|AAD55057.1\|, gi\|21950712\|gb\|AAM78591.1\|, gi\|147835837\|emb\|CAN72939.1\| | 2.65705E-4 |
|  | **GO:0009532- plastid stroma** | gi\|147770824\|emb\|CAN76428.1\|, gi\|147860809\|emb\|CAN81065.1\|, gi\|157345457\|emb\|CAO14755.1\|, gi\|147807983\|emb\|CAN64291.1\|, gi\|125545056\|gb\|EAY91195.1\|, gi\|157342483\|emb\|CAO65195.1\|, gi\|77540210\|gb\|ABA86963.1\|, gi\|11467200\|ref\|NP_043033.1\|, gi\|21950712\|gb\|AAM78591.1\| | 3.23896E-4 |
|  | **GO:0015935- small ribosomal subunit** | gi\|157359909\|emb\|CAO68645.1\|, gi\|147789484\|emb\|CAN71757.1\|, gi\|157359041\|emb\|CAO66943.1\|, gi\|76573325\|gb\|ABA46767.1\|, gi\|157336613\|emb\|CAO71298.1\|, gi\|56199526\|gb\|AAV84252.1\|, gi\|157348680\|emb\|CAO23572.1\|, gi\|147826473\|emb\|CAN72805.1\|, gi\|157357316\|emb\|CAO63839.1\| | 3.23896E-4 |
|  | **GO:0031984- organelle subcompartment**  **GO:0031976- plastid thylakoid**  **GO:0009534- chloroplast thylakoid** | gi\|81176302\|ref\|YP_398379.1\|, gi\|147770824\|emb\|CAN76428.1\|, gi\|157329311\|emb\|CAO39792.1\|, gi\|157357659\|emb\|CAO64639.1\|, gi\|157346115\|emb\|CAO15812.1\|, gi\|157354460\|emb\|CAO47086.1\|, gi\|115470052\|ref\|NP_001058625.1\|, gi\|157342483\|emb\|CAO65195.1\|, gi\|5881134\|gb\|AAD55057.1\|, gi\|147835837\|emb\|CAN72939.1\| | 0.00133462 |
|  | **GO:0042651- thylakoid membrane** | gi\|81176302\|ref\|YP_398379.1\|, gi\|157329311\|emb\|CAO39792.1\|, gi\|157357659\|emb\|CAO64639.1\|, gi\|157353870\|emb\|CAO46405.1\|, gi\|157346115\|emb\|CAO15812.1\|, gi\|157354460\|emb\|CAO47086.1\|, gi\|115470052\|ref\|NP_001058625.1\|, gi\|157342483\|emb\|CAO65195.1\|, gi\|5881134\|gb\|AAD55057.1\|, gi\|147835837\|emb\|CAN72939.1\| | 0.00133462 |
|  | **GO:0005840- ribosome**  **GO:0030529- ribonucleoprotein complex** | gi\|157339977\|emb\|CAO45654.1\|, gi\|125555687\|gb\|EAZ01293.1\|, gi\|157359909\|emb\|CAO68645.1\|, gi\|159479900\|ref\|XP_001698024.1\|, gi\|147835114\|emb\|CAN67946.1\|, gi\|157336744\|emb\|CAO71429.1\|, gi\|147789484\|emb\|CAN71757.1\|, gi\|61807078\|gb\|AAX55706.1\|, gi\|157359041\|emb\|CAO66943.1\|, gi\|76573325\|gb\|ABA46767.1\|, gi\|157336613\|emb\|CAO71298.1\|, gi\|56199526\|gb\|AAV84252.1\|, gi\|157348680\|emb\|CAO23572.1\|, gi\|147772865\|emb\|CAN75906.1\|, gi\|147858266\|emb\|CAN83920.1\|, gi\|147826473\|emb\|CAN72805.1\|, gi\|157345457\|emb\|CAO14755.1\|, gi\|157357599\|emb\|CAO64572.1\|, gi\|157347411\|emb\|CAO18048.1\|, gi\|157354887\|emb\|CAO48233.1\|, gi\|56202344\|dbj\|BAD73824.1\|, gi\|78191412\|gb\|ABB29927.1\|, gi\|5706704\|gb\|AAD47346.1\|AF112440_1, gi\|157357316\|emb\|CAO63839.1\|, gi\|157360345\|emb\|CAO69559.1\|, gi\|157352918\|emb\|CAO44808.1\|, gi\|464621\|sp\|P34091.1\|RL6_MESCR, gi\|82623429\|gb\|ABB87129.1\| | 0.00134414  0.00204537 |
|  | **GO:0030076- light-harvesting complex**  **Cellular component** | gi\|157346115\|emb\|CAO15812.1\|, gi\|77744799\|gb\|ABB02355.1\|, gi\|115531915\|ref\|YP_784071.1\|, gi\|74027097\|gb\|AAZ94647.1\| | 0.00151875 |
|  | **GO:0009570- chloroplast stroma** | gi\|147770824\|emb\|CAN76428.1\|, gi\|147807983\|emb\|CAN64291.1\|, gi\|125545056\|gb\|EAY91195.1\|, gi\|157342483\|emb\|CAO65195.1\|, gi\|77540210\|gb\|ABA86963.1\|, gi\|11467200\|ref\|NP_043033.1\|, gi\|21950712\|gb\|AAM78591.1\| | 0.00176787 |
|  | **GO:0030312- external encapsulating structure**  **GO:0005618- cell wall** | gi\|157328673\|emb\|CAO22686.1\| **14-3-3 protein (0.64)**  gi\|157338489\|emb\|CAO41840.1\| **reversibly glycosylated polypeptide (0.58)**  gi\|18202459\|sp\|P94014.1\|GL21_ARATH **germin-like protein (0.52)** gi\|157337343\|emb\|CAO21689.1\| **thaumatin-like cytokinin-binding protein (0.45)**  gi\|157328892\|emb\|CAO23301.1\| **atagal2 (arabidopsis thaliana alpha-galactosidase 2) alpha-galactosidase (0.39)**  gi\|157350105\|emb\|CAO40043.1\| **pectinesterase family protein (0.32)**  gi\|160419153\|sp\|P85076.1\|PME_ACTDE **pectinesterase family protein (0.28)**  gi\|147771556\|emb\|CAN78176.1\| **endo-xyloglucan transferase-like protein (0.19)**  gi\|57013892\|ref\|YP_173366.1\|,  gi\|157360727\|emb\|CAO70326.1\|, | 0.00221137 |
|  | **GO:0009535- chloroplast thylakoid membrane**  **GO:0055035- plastid thylakoid membrane** | gi\|81176302\|ref\|YP_398379.1\|, gi\|157329311\|emb\|CAO39792.1\|, gi\|157357659\|emb\|CAO64639.1\|, gi\|157346115\|emb\|CAO15812.1\|, gi\|157354460\|emb\|CAO47086.1\|, gi\|115470052\|ref\|NP_001058625.1\|, gi\|157342483\|emb\|CAO65195.1\|, gi\|5881134\|gb\|AAD55057.1\|, gi\|147835837\|emb\|CAN72939.1\| | 0.00372159 |
|  | **GO:0009507- chloroplast** | gi\|41053142\|dbj\|BAD08085.1 **ornithine carbamoyltransferase (0.68)**  gi\|147767264\|emb\|CAN69003.1\| **ketol-acid reductoisomerase (0.64)**  gi\|157346136\|emb\|CAO15833.1\| **2-cys peroxiredoxin-like protein (0.68)**  gi\|89242726\|gb\|ABD64688.1\| **allene oxide cyclase (0.63)**  gi\|157352161\|emb\|CAO43189.1\| **dc2 protein (0.63)**  gi\|157352888\|emb\|CAO44758.1\| **dehydroascorbate reductase (0.62)**  gi\|157345040\|emb\|CAO71768.1\| **disproportionating enzyme (0.61)**  gi\|157343766\|emb\|CAO68272.1\| **argininosuccinate synthase (0.58)**  gi\|157329774\|emb\|CAO41806.1**\| inorganic pyrophosphatase (0.57)**  gi\|147798865\|emb\|CAN68050.1\| **swib complex baf60b domain-containing protein (0.52)**  gi\|157342766\|emb\|CAO65478.1\| **glyoxalase I (0.52)**  gi\|157335857\|emb\|CAO61687.1\| **3-deoxy-d-arabino-heptulosonate 7-phosphate synthase (0.49)**  gi\|6635349\|gb\|AAF19792.1\|AF162209_1 **mpbq msbq methyltransferase 2 (0.48)**  gi\|157338214\|emb\|CAO39258.1\| **m-type thioredoxin (0.46)**  gi\|157337766\|emb\|CAO22112.1\| **cpn60b (chaperonin 60 beta) atp binding** **protein binding unfolded protein binding (0.46)**  gi\|40643247\|emb\|CAC83766.1\| **allene oxide cyclase (0.40)**  gi\|157332311\|emb\|CAO70107.1\| **3-deoxy-d-arabino-heptulosonate 7-phosphate synthase (0.36)**  gi\|157329265\|emb\|CAO38811.1\|, \|, gi\|81176302\|ref\|YP_398379.1\|, gi\|147804704\|emb\|CAN71620.1\|, gi\|157346674\|emb\|CAO16743.1\|, gi\|157347388\|emb\|CAO17917.1\|, gi\|147770824\|emb\|CAN76428.1\|, gi\|157329311\|emb\|CAO39792.1\|, gi\|115604959\|ref\|YP_784412.1\|, gi\|157357659\|emb\|CAO64639.1\|, gi\|147802302\|emb\|CAN70407.1\|, gi\|157354569\|emb\|CAO47880.1\|, gi\|157340522\|emb\|CAO47327.1\|, gi\|7271945\|gb\|AAF44702.1\|AF241524_1, gi\|147826727\|emb\|CAN66108.1\|, gi\|157353870\|emb\|CAO46405.1\|, gi\|147860809\|emb\|CAN81065.1\|, gi\|157345457\|emb\|CAO14755.1\|, gi\|157359015\|emb\|CAO66917.1\|, gi\|157346115\|emb\|CAO15812.1\|, gi\|157354460\|emb\|CAO47086.1\|, gi\|147800882\|emb\|CAN62373.1\|, gi\|115470052\|ref\|NP_001058625.1\|, gi\|157336150\|emb\|CAO61980.1\|, gi\|157350250\|emb\|CAO40188.1\|, gi\|147807983\|emb\|CAN64291.1\|, gi\|77744799\|gb\|ABB02355.1\|, gi\|157346726\|emb\|CAO16795.1\|, gi\|115531915\|ref\|YP_784071.1\|, gi\|125545056\|gb\|EAY91195.1\|, gi\|157342483\|emb\|CAO65195.1\|, gi\|157358587\|emb\|CAO66263.1\|, gi\|77540210\|gb\|ABA86963.1\|, gi\|1785613\|gb\|AAB41022.1\|, gi\|84620802\|gb\|ABC59515.1\|, gi\|11467200\|ref\|NP_043033.1\|, gi\|5881134\|gb\|AAD55057.1\|, gi\|157344623\|emb\|CAO70006.1\|, gi\|21950712\|gb\|AAM78591.1\|, gi\|47169444\|pdb\|1VCR\|A, gi\|139387445\|ref\|YP_001122799.1\|, gi\|74027097\|gb\|AAZ94647.1\|, gi\|147835837\|emb\|CAN72939.1\|, gi\|157333396\|emb\|CAO17011.1\| | 0.0 |
|  | **GO:0043229- intracellular organelle**  **GO:0043226- organelle** | gi\|147802302\|emb\|CAN70407.1\| **malate dehydrogenase (0.68)**  gi\|41053142\|dbj\|BAD08085.1\| **ornithine carbamoyltransferase (0.68)**  gi\|157338304\|emb\|CAO39348.1\| **reticulon 4 interacting protein 1 (0.66)**  gi\|33326375\|gb\|AAQ08597.1\| **heat shock protein 82 (0.66)**  gi\|147818169\|emb\|CAN78076.1\| **dienelactone hydrolase family protein (0.61)**  gi\|157341055\|emb\|CAO47860.1\| **phosphoglycerate dehydrogenase (0.60)**  gi\|147801554\|emb\|CAN77010.1\| **10 kda chaperonin (0.60)**  gi\|157328606\|emb\|CAO22302.1\| **subtilisin-like protease (0.59)**  gi\|157359960\|emb\|CAO68704.1\| **transaldolase (0.59)**  gi\|157412354\|ref\|YP_001483220.1\| **heat shock protein (0.58)**  gi\|157345088\|emb\|CAO71816.1\| **aspartate aminotransferase (0.46)**  gi\|157345964\|emb\|CAO15661.1\| **catalytic coenzyme binding (0.45)**  gi\|19070130\|gb\|AAL83720.1\|AF236127_1 **catalase (0.43)**  gi\|1063400\|emb\|CAA63482.1\| **glycolate oxidase (0.43)**  gi\|147856880\|emb\|CAN79174.1\| **sulfite reductase (0.42)**  gi\|157342803\|emb\|CAO65515.1\| **signal peptidase I (0.42)**  gi\|157341593\|emb\|CAO62148.1\| **protein transport (0.42)**  gi\|157336170\|emb\|CAO62000.1\| **sks5 (sku5 similar 5) copper ion binding oxidoreductase (0.41)**  gi\|147771308\|emb\|CAN69536.1\| **nucleoid dna-binding-like protein (0.41)**  gi\|157343385\|emb\|CAO67436.1\| **glycosyl hydrolase family 3 n terminal domain containingexpressed (0.41)**  gi\|157339977\|emb\|CAO45654.1\|, gi\|157329265\|emb\|CAO38811.1\|, gi\|81176302\|ref\|YP_398379.1\|, gi\|157346136\|emb\|CAO15833.1\|, gi\|125555687\|gb\|EAZ01293.1\|, gi\|157359909\|emb\|CAO68645.1\|, gi\|147804704\|emb\|CAN71620.1\|, gi\|157346674\|emb\|CAO16743.1\|, gi\|159479900\|ref\|XP_001698024.1\|, gi\|157357936\|emb\|CAO64948.1\|, gi\|157347388\|emb\|CAO17917.1\|, gi\|147835114\|emb\|CAN67946.1\|, gi\|157336744\|emb\|CAO71429.1\|, gi\|147789484\|emb\|CAN71757.1\|, gi\|157339714\|emb\|CAO44255.1\|, gi\|147770824\|emb\|CAN76428.1\|, gi\|157329311\|emb\|CAO39792.1\|, gi\|147767264\|emb\|CAN69003.1\|, gi\|61807078\|gb\|AAX55706.1\|, gi\|115604959\|ref\|YP_784412.1\|, gi\|157357659\|emb\|CAO64639.1\|, gi\|157335152\|emb\|CAO60982.1\|, gi\|89242726\|gb\|ABD64688.1\|, gi\|157354569\|emb\|CAO47880.1\|, gi\|157352161\|emb\|CAO43189.1\|, gi\|147863982\|emb\|CAN81107.1\|, gi\|157352888\|emb\|CAO44758.1\|, gi\|157359041\|emb\|CAO66943.1\|, gi\|157340522\|emb\|CAO47327.1\|, gi\|157345040\|emb\|CAO71768.1\|, gi\|157355561\|emb\|CAO48961.1\|, gi\|76573325\|gb\|ABA46767.1\|, gi\|157336613\|emb\|CAO71298.1\|, gi\|56199526\|gb\|AAV84252.1\|, gi\|7271945\|gb\|AAF44702.1\|AF241524_1, gi\|157348680\|emb\|CAO23572.1\|, gi\|59800134\|gb\|AAX07420.1\|, gi\|147826727\|emb\|CAN66108.1\|, gi\|157353870\|emb\|CAO46405.1\|, gi\|147860809\|emb\|CAN81065.1\|, gi\|147772865\|emb\|CAN75906.1\|, gi\|147858266\|emb\|CAN83920.1\|, gi\|157343766\|emb\|CAO68272.1\|, gi\|147826473\|emb\|CAN72805.1\|, gi\|157338489\|emb\|CAO41840.1\|, gi\|8699606\|gb\|AAF78758.1\|AF271661_1, gi\|3115108\|emb\|CAA11757.1\|, gi\|157329774\|emb\|CAO41806.1\|, gi\|157345457\|emb\|CAO14755.1\|, gi\|157359015\|emb\|CAO66917.1\|, gi\|157357599\|emb\|CAO64572.1\|, gi\|157346115\|emb\|CAO15812.1\|, gi\|66773102\|ref\|NP_001019593.1\|, gi\|157347411\|emb\|CAO18048.1\|, gi\|157354460\|emb\|CAO47086.1\|, gi\|147800882\|emb\|CAN62373.1\|, gi\|115470052\|ref\|NP_001058625.1\|, gi\|57013892\|ref\|YP_173366.1\|, gi\|157336150\|emb\|CAO61980.1\|, gi\|157337397\|emb\|CAO21743.1\|, gi\|147798865\|emb\|CAN68050.1\|, gi\|157354887\|emb\|CAO48233.1\|, gi\|56202344\|dbj\|BAD73824.1\|, gi\|157342766\|emb\|CAO65478.1\|, gi\|157350250\|emb\|CAO40188.1\|, gi\|157360727\|emb\|CAO70326.1\|, gi\|147807983\|emb\|CAN64291.1\|, gi\|147802483\|emb\|CAN77412.1\|, gi\|56412221\|gb\|AAV88605.1\|, gi\|77744799\|gb\|ABB02355.1\|, gi\|157335857\|emb\|CAO61687.1\|, gi\|157346726\|emb\|CAO16795.1\|, gi\|78191412\|gb\|ABB29927.1\|, gi\|115531915\|ref\|YP_784071.1\|, gi\|125545056\|gb\|EAY91195.1\|, gi\|6635349\|gb\|AAF19792.1\|AF162209_1, gi\|157342483\|emb\|CAO65195.1\|, gi\|5706704\|gb\|AAD47346.1\|AF112440_1, gi\|157357316\|emb\|CAO63839.1\|, gi\|157358587\|emb\|CAO66263.1\|, gi\|77540210\|gb\|ABA86963.1\|, gi\|1785613\|gb\|AAB41022.1\|, gi\|157338214\|emb\|CAO39258.1\|, gi\|157360345\|emb\|CAO69559.1\|, gi\|157337766\|emb\|CAO22112.1\|, gi\|84620802\|gb\|ABC59515.1\|, gi\|81076111\|gb\|ABB55387.1\|, gi\|157352918\|emb\|CAO44808.1\|, gi\|157337343\|emb\|CAO21689.1\|, gi\|147781068\|emb\|CAN68131.1\|, gi\|11467200\|ref\|NP_043033.1\|, gi\|464621\|sp\|P34091.1\|RL6_MESCR, gi\|5881134\|gb\|AAD55057.1\|, gi\|40643247\|emb\|CAC83766.1\|, gi\|462187\|sp\|P34899.1\|GLYM_PEA, gi\|157335911\|emb\|CAO61741.1\|, gi\|63029722\|gb\|AAY27752.1\|, gi\|157338782\|emb\|CAO42133.1\|, gi\|157344623\|emb\|CAO70006.1\|, gi\|21950712\|gb\|AAM78591.1\|, gi\|157332311\|emb\|CAO70107.1\|, gi\|47169444\|pdb\|1VCR\|A, gi\|157355918\|emb\|CAO49775.1\|, gi\|139387445\|ref\|YP_001122799.1\|, gi\|82623429\|gb\|ABB87129.1\|, gi\|89147796\|gb\|ABD62753.1\|, gi\|74027097\|gb\|AAZ94647.1\|, gi\|147835837\|emb\|CAN72939.1\|, gi\|157333396\|emb\|CAO17011.1\|, gi\|563329\|emb\|CAA57778.1\|, gi\|157338974\|emb\|CAO42325.1\|, gi\|157348346\|emb\|CAO23133.1\|, gi\|147820236\|emb\|CAN62640.1\|, gi\|157357159\|emb\|CAO63653.1\|, gi\|157348402\|emb\|CAO23189.1\| | 0.00307699 |
|  | **GO:0015979- photosynthesis** | gi\|157329265\|emb\|CAO38811.1\| **photosystem ii oxygen-evolving complex protein 3-like (0.68**)  gi\|81176302\|ref\|YP_398379.1\| **photosystem i subunit vii (0.68**) gi\|147804704\|emb\|CAN71620.1\| **lhcb6 protein (0.68)** gi\|157347388\|emb\|CAO17917.1\| **type iii chlorophyll a b-binding protein (0.66)**  gi\|147770824\|emb\|CAN76428.1\| **chlorophyll a b-binding apoprotein cp26 precursor (0.65)**  gi\|157329311\|emb\|CAO39792.1\| **fibrillin (0.65)** gi\|115604959\|ref\|YP_784412.1\| **photosystem ii cp47 chlorophyll apoprotein (0.63)**  gi\|157354569\|emb\|CAO47880.1\| **phosphoglycerate kinase (0.63)**  gi\|157340522\|emb\|CAO47327.1\| **triosephosphate isomerase (0.62)**  gi\|7271945\|gb\|AAF44702.1\|AF241524_1 **chlorophyll a b-binding protein (0.60)**  gi\|147826727\|emb\|CAN66108.1\| **rieske iron-sulphur protein precursor (0.59)**  gi\|157353870\|emb\|CAO46405.1\| **psan (photosystem i reaction center subunit psi-n) calmodulin binding (0.59)**  gi\|157359015\|emb\|CAO66917.1\| **chlorophyll a b binding protein (0.56)**  gi\|157346115\|emb\|CAO15812.1\| **chlorophyll a b-binding protein cp29 (0.56)**  gi\|157354460\|emb\|CAO47086.1\| **photosystem i subunit iii precursor (0.56)**  gi\|147800882\|emb\|CAN62373.1\| **psi reaction center subunit ii (0.56)**  gi\|115470052\|ref\|NP_001058625.1\| **cell division protein (0.54)**  gi\|157350250\|emb\|CAO40188.1\| **chlorophyll a b binding protein (0.51)**  gi\|147807983\|emb\|CAN64291.1\| **ribulose bisphosphatesmall subunit (0.50)**  gi\|77744799\|gb\|ABB02355.1\| **photosystem ii protein d1 (0.49)**  gi\|115531915\|ref\|YP_784071.1\| **photosystem ii cp43 chlorophyll apoprotein (0.48)**  gi\|157358587\|emb\|CAO66263.1\| **glyceraldehyde-3-phosphate dehydrogenase (0.47)**  gi\|77540210\|gb\|ABA86963.1\| **at3g26650 mlj15_5 (0.47)**  gi\|84620802\|gb\|ABC59515.1\| **chloroplast photosystem ii 22 kda component (0.46)**  gi\|11467200\|ref\|NP_043033.1\| **ribulose--bisphosphate carboxylase oxygenase large subunit (0.43)**  gi\|5881134\|gb\|AAD55057.1\| **phosphoribulokinase precursor (0.41)**  gi\|157344623\|emb\|CAO70006.1\| **chlorophyll a b-binding protein (0.37)**  gi\|47169444\|pdb\|1VCR\|A **chlorophyll a b binding protein (0.35)**  gi\|139387445\|ref\|YP_001122799.1\| **photosystem i p700 apoprotein a2 (0.34)**  gi\|74027097\|gb\|AAZ94647.1\| **photosystem ii cp43 chlorophyll apoprotein (0.31)**  gi\|147835837\|emb\|CAN72939.1\| **transketolase 1 (0.28)**  gi\|157333396\|emb\|CAO17011.1\| **chloroplast sedoheptulose—bisphosphatase (0.26)** | 4.95725E-13 |
|  | **GO:0019684- photosynthesis, light reaction** | gi\|157347388\|emb\|CAO17917.1\|, gi\|147770824\|emb\|CAN76428.1\|, gi\|157329311\|emb\|CAO39792.1\|, gi\|115604959\|ref\|YP_784412.1\|, gi\|7271945\|gb\|AAF44702.1\|AF241524_1, gi\|147826727\|emb\|CAN66108.1\|, gi\|157353870\|emb\|CAO46405.1\|, gi\|157359015\|emb\|CAO66917.1\|, gi\|157346115\|emb\|CAO15812.1\|, gi\|115470052\|ref\|NP_001058625.1\|, gi\|157350250\|emb\|CAO40188.1\|, gi\|77744799\|gb\|ABB02355.1\|, gi\|115531915\|ref\|YP_784071.1\|, gi\|84620802\|gb\|ABC59515.1\|, gi\|157344623\|emb\|CAO70006.1\|, gi\|47169444\|pdb\|1VCR\|A, gi\|74027097\|gb\|AAZ94647.1\| | 1.5376E-8 |
|  | **GO:0006996- organelle organization and biogenesis** | gi\|157346726\|emb\|CAO16795.1\| **rubisco subunit binding-protein alpha subunit (0.49)**  gi\|157339977\|emb\|CAO45654.1\|, gi\|125555687\|gb\|EAZ01293.1\|, gi\|157359909\|emb\|CAO68645.1\|, gi\|159479900\|ref\|XP_001698024.1\|, gi\|147835114\|emb\|CAN67946.1\|, gi\|157336744\|emb\|CAO71429.1\|, gi\|147789484\|emb\|CAN71757.1\|, gi\|61807078\|gb\|AAX55706.1\|, gi\|157335152\|emb\|CAO60982.1\|, gi\|157359041\|emb\|CAO66943.1\|, gi\|76573325\|gb\|ABA46767.1\|, gi\|157336613\|emb\|CAO71298.1\|, gi\|56199526\|gb\|AAV84252.1\|, gi\|157348680\|emb\|CAO23572.1\|, gi\|147772865\|emb\|CAN75906.1\|, gi\|147858266\|emb\|CAN83920.1\|, gi\|147826473\|emb\|CAN72805.1\|, gi\|157345457\|emb\|CAO14755.1\|, gi\|157357599\|emb\|CAO64572.1\|, gi\|66773102\|ref\|NP_001019593.1\|, gi\|157347411\|emb\|CAO18048.1\|, gi\|157354887\|emb\|CAO48233.1\|, gi\|56202344\|dbj\|BAD73824.1\|, gi\|147802483\|emb\|CAN77412.1\|, gi\|78191412\|gb\|ABB29927.1\|, gi\|125545056\|gb\|EAY91195.1\|, gi\|5706704\|gb\|AAD47346.1\|AF112440_1, gi\|157357316\|emb\|CAO63839.1\|, gi\|157360345\|emb\|CAO69559.1\|, gi\|157352918\|emb\|CAO44808.1\|, gi\|464621\|sp\|P34091.1\|RL6_MESCR, gi\|82623429\|gb\|ABB87129.1\|, gi\|89147796\|gb\|ABD62753.1\|, gi\|563329\|emb\|CAA57778.1\|, gi\|157338974\|emb\|CAO42325.1\| | 1.78164E-7 |
|  | **GO:0009765- photosynthesis, light harvesting** | gi\|147804704\|emb\|CAN71620.1\|, gi\|157347388\|emb\|CAO17917.1\|, gi\|147770824\|emb\|CAN76428.1\|, gi\|7271945\|gb\|AAF44702.1\|AF241524_1, gi\|157359015\|emb\|CAO66917.1\|, gi\|157346115\|emb\|CAO15812.1\|, gi\|115470052\|ref\|NP_001058625.1\|, gi\|157350250\|emb\|CAO40188.1\|, gi\|84620802\|gb\|ABC59515.1\|, gi\|157344623\|emb\|CAO70006.1\|, gi\|47169444\|pdb\|1VCR\|A | 3.10826E-6 |
|  | **GO:0018298- protein-chromophore linkage** | gi\|147804704\|emb\|CAN71620.1\|, gi\|157347388\|emb\|CAO17917.1\|, gi\|7271945\|gb\|AAF44702.1\|AF241524_1, gi\|157359015\|emb\|CAO66917.1\|, gi\|157350250\|emb\|CAO40188.1\|, gi\|115531915\|ref\|YP_784071.1\|, gi\|157344623\|emb\|CAO70006.1\|, gi\|47169444\|pdb\|1VCR\|A, gi\|139387445\|ref\|YP_001122799.1\|, gi\|74027097\|gb\|AAZ94647.1\| | 3.58546E-6 |
|  | **GO:0042254- ribosome biogenesis and assembly**  **GO:0003735- structural constituent of ribosome** | gi\|157339977\|emb\|CAO45654.1\|, gi\|125555687\|gb\|EAZ01293.1\|, gi\|157359909\|emb\|CAO68645.1\|, gi\|159479900\|ref\|XP_001698024.1\|, gi\|147835114\|emb\|CAN67946.1\|, gi\|157336744\|emb\|CAO71429.1\|, gi\|147789484\|emb\|CAN71757.1\|, gi\|61807078\|gb\|AAX55706.1\|, gi\|157359041\|emb\|CAO66943.1\|, gi\|76573325\|gb\|ABA46767.1\|, gi\|157336613\|emb\|CAO71298.1\|, gi\|56199526\|gb\|AAV84252.1\|, gi\|157348680\|emb\|CAO23572.1\|, gi\|147772865\|emb\|CAN75906.1\|, gi\|147858266\|emb\|CAN83920.1\|, gi\|147826473\|emb\|CAN72805.1\|, gi\|157345457\|emb\|CAO14755.1\|, gi\|157357599\|emb\|CAO64572.1\|, gi\|157347411\|emb\|CAO18048.1\|, gi\|157354887\|emb\|CAO48233.1\|, gi\|56202344\|dbj\|BAD73824.1\|, gi\|78191412\|gb\|ABB29927.1\|, gi\|5706704\|gb\|AAD47346.1\|AF112440_1, gi\|157357316\|emb\|CAO63839.1\|, gi\|157360345\|emb\|CAO69559.1\|, gi\|157352918\|emb\|CAO44808.1\|, gi\|464621\|sp\|P34091.1\|RL6_MESCR, gi\|82623429\|gb\|ABB87129.1\| | 3.68313E-8 |
| **Biological process** | **GO:0019685- photosynthesis, dark reaction**  **GO:0019253- reductive pentose-phosphate cycle** | gi\|157358587\|emb\|CAO66263.1\| **glyceraldehyde-3-phosphate dehydrogenase (0.47)**  gi\|77540210\|gb\|ABA86963.1\| **at3g26650 mlj15_5 (0.47)**  gi\|157333396\|emb\|CAO17011.1\| **chloroplast sedoheptulose—bisphosphatase (0.26)**  gi\|157354569\|emb\|CAO47880.1\|, gi\|157340522\|emb\|CAO47327.1\|, gi\|147807983\|emb\|CAN64291.1\|, gi\|11467200\|ref\|NP_043033.1\|, gi\|5881134\|gb\|AAD55057.1\|, gi\|147835837\|emb\|CAN72939.1\|, | 1.56512E-5 |
|  | **GO:0022613- ribonucleoprotein complex biogenesis and assembly** | gi\|157339977\|emb\|CAO45654.1\|, gi\|125555687\|gb\|EAZ01293.1\|, gi\|157359909\|emb\|CAO68645.1\|, gi\|159479900\|ref\|XP_001698024.1\|, gi\|147835114\|emb\|CAN67946.1\|, gi\|157336744\|emb\|CAO71429.1\|, gi\|147789484\|emb\|CAN71757.1\|, gi\|61807078\|gb\|AAX55706.1\|, gi\|157359041\|emb\|CAO66943.1\|, gi\|76573325\|gb\|ABA46767.1\|, gi\|157336613\|emb\|CAO71298.1\|, gi\|56199526\|gb\|AAV84252.1\|, gi\|157348680\|emb\|CAO23572.1\|, gi\|147772865\|emb\|CAN75906.1\|, gi\|147858266\|emb\|CAN83920.1\|, gi\|147826473\|emb\|CAN72805.1\|, gi\|157345457\|emb\|CAO14755.1\|, gi\|157357599\|emb\|CAO64572.1\|, gi\|157347411\|emb\|CAO18048.1\|, gi\|157354887\|emb\|CAO48233.1\|, gi\|56202344\|dbj\|BAD73824.1\|, gi\|78191412\|gb\|ABB29927.1\|, gi\|5706704\|gb\|AAD47346.1\|AF112440_1, gi\|157357316\|emb\|CAO63839.1\|, gi\|157360345\|emb\|CAO69559.1\|, gi\|157352918\|emb\|CAO44808.1\|, gi\|464621\|sp\|P34091.1\|RL6_MESCR, gi\|82623429\|gb\|ABB87129.1\| | 1.6656E-5 |
|  | **GO:0009767- photosynthetic electron transport** | gi\|157329311\|emb\|CAO39792.1\|, gi\|115604959\|ref\|YP_784412.1\|, gi\|147826727\|emb\|CAN66108.1\|, gi\|157353870\|emb\|CAO46405.1\|, gi\|115531915\|ref\|YP_784071.1\|, gi\|74027097\|gb\|AAZ94647.1\| | 0.00281885 |
|  | **GO:0016043- cellular component organization and biogenesis** | gi\|157343968\|emb\|CAO68901.1\| **component of the exocyst complex (0.66)**  gi\|157339977\|emb\|CAO45654.1\|, gi\|147861641\|emb\|CAN79299.1\|, gi\|125555687\|gb\|EAZ01293.1\|, gi\|157359909\|emb\|CAO68645.1\|, gi\|159479900\|ref\|XP_001698024.1\|, gi\|147835114\|emb\|CAN67946.1\|, gi\|157336744\|emb\|CAO71429.1\|, gi\|147789484\|emb\|CAN71757.1\|, gi\|157339714\|emb\|CAO44255.1\|, gi\|61807078\|gb\|AAX55706.1\|, gi\|157335152\|emb\|CAO60982.1\|, gi\|157359041\|emb\|CAO66943.1\|, gi\|76573325\|gb\|ABA46767.1\|, gi\|157336613\|emb\|CAO71298.1\|, gi\|56199526\|gb\|AAV84252.1\|, gi\|157348680\|emb\|CAO23572.1\|, gi\|147772865\|emb\|CAN75906.1\|, gi\|157347500\|emb\|CAO18137.1\|, gi\|147858266\|emb\|CAN83920.1\|, gi\|147826473\|emb\|CAN72805.1\|, gi\|157338489\|emb\|CAO41840.1\|, gi\|3115108\|emb\|CAA11757.1\|, gi\|157345457\|emb\|CAO14755.1\|, gi\|157357599\|emb\|CAO64572.1\|, gi\|66773102\|ref\|NP_001019593.1\|, gi\|157347411\|emb\|CAO18048.1\|, gi\|57013892\|ref\|YP_173366.1\|, gi\|157354887\|emb\|CAO48233.1\|, gi\|56202344\|dbj\|BAD73824.1\|, gi\|157360727\|emb\|CAO70326.1\|, gi\|147802483\|emb\|CAN77412.1\|, gi\|157346726\|emb\|CAO16795.1\|, gi\|78191412\|gb\|ABB29927.1\|, gi\|125545056\|gb\|EAY91195.1\|, gi\|5706704\|gb\|AAD47346.1\|AF112440_1, gi\|157357316\|emb\|CAO63839.1\|, gi\|157360345\|emb\|CAO69559.1\|, gi\|157352918\|emb\|CAO44808.1\|, gi\|464621\|sp\|P34091.1\|RL6_MESCR, gi\|82623429\|gb\|ABB87129.1\|, gi\|89147796\|gb\|ABD62753.1\|, gi\|157350105\|emb\|CAO40043.1\|, gi\|160419153\|sp\|P85076.1\|PME_ACTDE, gi\|563329\|emb\|CAA57778.1\|, gi\|157338974\|emb\|CAO42325.1\| | 0.00539337 |
| **Molecular function** | **GO:0005507- copper ion binding** | gi\|157346674\|emb\|CAO16743.1\| **cu zn superoxide dismutase precursor (0.66)**  gi\|157357665\|emb\|CAO64645.1\| **diphenol oxidase (0.56)**  gi\|157360727\|emb\|CAO70326.1\| **pollen-specific protein (0.50)**  gi\|56412221\|gb\|AAV88605.1\| **ole e 5 olive pollen allergen (0.49)**  gi\|1785613\|gb\|AAB41022.1\| **polyphenol oxidase (0.47)**  gi\|157336170\|emb\|CAO62000.1\| **sks5 (sku5 similar 5) copper ion binding oxidoreductase (0.41)**  gi\|157336150\|emb\|CAO61980.1\|, | 0.00176787 |
|  | **GO:0016168- chlorophyll binding** | gi\|115604959\|ref\|YP_784412.1\|, gi\|115531915\|ref\|YP_784071.1\|, gi\|84620802\|gb\|ABC59515.1\|, gi\|74027097\|gb\|AAZ94647.1\| | 0.00151875 |
|  | **GO:0005198- structural molecule activity** | gi\|157339977\|emb\|CAO45654.1\| **ribosomal protein l9 (0.68)** gi\|125555687\|gb\|EAZ01293.1\| **ribosomal proteincytosolic (0.67)**  gi\|157359909\|emb\|CAO68645.1\| **ribosomal protein s6 (0.67)**  gi\|159479900\|ref\|XP_001698024.1\| **60s ribosomal protein l21 (0.66)**  gi\|147835114\|emb\|CAN67946.1\| **40s ribosomal protein s17 (0.65)**  gi\|157336744\|emb\|CAO71429.1\| **60s ribosomal protein l14 (0.65)**  gi\|147789484\|emb\|CAN71757.1\| **40s ribosomal protein s7 (0.65)**  gi\|157329311\|emb\|CAO39792.1\| **fibrillin (0.64)**  gi\|61807078\|gb\|AAX55706.1\| **ribosomal protein s3a (0.64)**  gi\|157335152\|emb\|CAO60982.1\| **beta-tubulin (0.63)**  gi\|157359041\|emb\|CAO66943.1\| **40s ribosomal protein s14 (0.62)**  gi\|76573325\|gb\|ABA46767.1\| **40s ribosomal protein s9 (0.61)**  gi\|157336613\|emb\|CAO71298.1\| **40s ribosomal protein s15 (0.61)**  gi\|56199526\|gb\|AAV84252.1\| **ribosomal protein s8 (0.60)**  gi\|157348680\|emb\|CAO23572.1\| **40s ribosomal protein s2 (0.60)**  gi\|59800134\|gb\|AAX07420.1\| **actin (0.59)**  gi\|147772865\|emb\|CAN75906.1\| **60s ribosomal protein l26 (0.59)**  gi\|147858266\|emb\|CAN83920.1\| **40s ribosomal protein s19 (0.59)**  gi\|147826473\|emb\|CAN72805.1\| **40s ribosomal protein s16 (0.58)**  gi\|157345457\|emb\|CAO14755.1\| **ribosomal protein l12 (0.56)**  gi\|157357599\|emb\|CAO64572.1\| **60s ribosomal proteinbbc1 protein (0.56)**  gi\|66773102\|ref\|NP_001019593.1\| **beta 2b (0.56)**  gi\|157347411\|emb\|CAO18048.1\| **60s ribosomal (0.56)**  gi\|157354887\|emb\|CAO48233.1\| **ribosomal protein l8 (0.52)**  gi\|56202344\|dbj\|BAD73824.1\| **60s ribosomal protein l10a (0.52)**  gi\|147802483\|emb\|CAN77412.1\| **alpha tubulin 1 (0.50)**  gi\|78191412\|gb\|ABB29927.1\| **60s ribosomal protein l35 (0.49)**  gi\|9630742\|ref\|NP_047285.1\| **coat protein (0.48)**  gi\|5706704\|gb\|AAD47346.1\|AF112440_1 **40s ribosomal protein s26 (0.48)**  gi\|157360345\|emb\|CAO69559.1\| **60s ribosomal protein l7 (0.46)**  gi\|157352918\|emb\|CAO44808.1\| **cytoplasmic ribosomal protein l18 (0.46)**  gi\|464621\|sp\|P34091.1\|RL6_MESCR **60s ribosomal protein l6 (0.42)**  gi\|82623429\|gb\|ABB87129.1\| **60s ribosomal protein l7a (0.34)** | 1.75506E-8 |
