# Supplementary material for: iTRAQ-based protein profiling provides insights into the central metabolism changes driving grape berry development and ripening
Source: BMC Plant Biol. 2013 Oct 24;13:167. doi: 10.1186/1471-2229-13-167 (PMC4016569; doi:10.1186/1471-2229-13-167)
Supplement: Additional file 8 — The GO terms enriched and sequences annotated with such terms in the up- or down-regulated subsets of proteins in each grape berry developmental stage step analyzed. [file 1471-2229-13-167-S8.zip › Additional file 8/15mm to E100- up.doc]

| **Cellular component** | **GO:0016023- cytoplasmic membrane-bound vesicle**  **GO:0031410- cytoplasmic vesicle**  **GO:0031982- Vesicle**  **GO:0031988- membrane-bound vesicle** | gi\|157337528\|emb\|CAO21874.1\|, **gri22_vitviripening-related protein** **grip22 precursor (5.49)**  gi\|157344858\|emb\|CAO70613.1\|, **beta expansin 1 precursor (4.26)**  gi\|157336811\|emb\|CAO71496.1\|, **plasma membrane associated protein (3.83)**  gi\|157360883\|emb\|CAO70700.1\|, **aspartic proteinase nepenthesin I (2.81)**  gi\|157345245\|emb\|CAO71973.1\|, **subtilisin-like protease (2.07)**  gi\|157352288\|emb\|CAO43316.1\|, **xylose isomerase (1.81)**  gi\|30689144\|ref\|NP_194903.2\|, **protein phosphatase 2c (1.81)**  gi\|157340449\|emb\|CAO47254.1\|, **ubiquitin fusion degradation ufd1 family protein (1.80)**  gi\|82749763\|gb\|ABB89766.1\|, **cysteine proteinase inhibitor (1.78)**  gi\|157341994\|emb\|CAO63450.1\|, **haloacid dehalogenase-like hydrolase familyexpressed (1.66)**  gi\|147858661\|emb\|CAN80823.1\|, **acireductone dioxygenase (1.59)**  gi\|157350711\|emb\|CAO40802.1\|, **at5g25460 f18g18_200 (1.52)**  gi\|147818959\|emb\|CAN67131.1\|, **---NA--- (1.52)**  gi\|17221690\|gb\|AAL36459.1\|AF205603_1, gi\|157349778\|emb\|CAO39634.1\|, gi\|17402583\|dbj\|BAB78506.1\|, gi\|157340696\|emb\|CAO47501.1\|, gi\|157330183\|emb\|CAO44690.1\|,  gi\|157353734\|emb\|CAO46266.1\|, gi\|157335296\|emb\|CAO61126.1\|, | 0.00191252 |
| --- | --- | --- | --- |
|  | **GO:0005576- extracelullar region**  **15 mm to E100- up** | gi\|71668369\|gb\|AAZ38818.1\| **alpha-expansin 4 (10)**  gi\|17402583\|dbj\|BAB78506.1\| **xyloglucan endo-transglycosylase (9.37)**  gi\|157344858\|emb\|CAO70613.1\|, gi\|157348466\|emb\|CAO23358.1\|, gi\|157348620\|emb\|CAO23512.1\| **polygalacturonase-inhibiting protein (1.80)**  gi\|157343535\|emb\|CAO68041.1\| **phosphatase 2a regulatory a subunit (1.66)**  gi\|61369127\|gb\|AAX43289.1\| **s100 calcium binding protein a7 (1.59)** | 0.0028254 |
| **Biological process** | **GO:0010382- cell wall metabolic process** | gi\|116329\|sp\|P29022.1\|CHIA_MAIZE **chitinase (4.39)**  gi\|3511147\|gb\|AAC33732.1\| **pr-4 type protein (3.74)**  gi\|2306813\|gb\|AAB65777.1\| **class iv chitinase (3.69)**  gi\|157353734\|emb\|CAO46266.1\| **class iv chitinase (2.63)**  gi\|157335296\|emb\|CAO61126.1\| **(1-4)-beta-mannan endohydrolase (2.51)** | 2.97566E-5 |
|  | **GO:0016998- cell wall catabolic process**  **GO:0006032- chitin catabolic process**  **GO:0046348-**  **amino sugar catabolic process**  **GO: 0006030- chitin metabolic process**  **GO:0006041- glucosamine metabolic process**  **GO:0006046- N-acetylglucosamine catabolic process**  **GO:0006043- glucosamine catabolic process**  **GO:0044247- cellular polysaccharide catabolic process**  **GO:0000272- polysaccharide catabolic process**  **GO:0006044- N-acetylglucosamine metabolic process** | gi\|116329\|sp\|P29022.1\|CHIA_MAIZE, gi\|3511147\|gb\|AAC33732.1\|, gi\|2306813\|gb\|AAB65777.1\|, gi\|157353734\|emb\|CAO46266.1\| | 2.42649E-4 |
|  | **GO:0005976- polysaccharide metabolic process** | gi\|17402583\|dbj\|BAB78506.1\| **xyloglucan endo-transglycosylase (9,37)**  gi\|116329\|sp\|P29022.1\|CHIA_MAIZE,  gi\|7406663\|emb\|CAB85625.1\| **invertase pectin methylesterase inhibitor family protein (3.86)**  gi\|157360112\|emb\|CAO69295.1\| **sucrose-phosphate synthase (3.85)**  gi\|3511147\|gb\|AAC33732.1\| **pr-4 type protein (3.74)**  gi\|2306813\|gb\|AAB65777.1\|,  gi\|157340696\|emb\|CAO47501.1\| **sks5 (sku5 similar 5) copper ion binding oxidoreductase (2.98)**  gi\|157353734\|emb\|CAO46266.1\|,  gi\|157335296\|emb\|CAO61126.1\|,  gi\|157348466\|emb\|CAO23358.1\| **beta 1-3 glucanase (2.39)**  gi\|9885274\|gb\|AAG01382.1\|AF194174_1 **alcohol dehydrogenase(2.24)**  gi\|22347633\|gb\|AAM95944.1\| **sucrose synthase (2.19)**  gi\|147800323\|emb\|CAN68704.1\| **sucrose synthase (2.14)**  gi\|157353908\|emb\|CAO46443.1\| **pectin methylesterase-like protein (2.04)**  gi\|147766093\|emb\|CAN65700.1\| **dtdp-glucose 4-6-dehydratases-like protein (1.79)**  gi\|55977779\|sp\|P22221.2\|PPDK_FLATR **phosphatechloroplast precursor (orthophosphate dikinase) (1.74)** | 2.42649E-4 |
|  | **GO:0005982- starch metabolic process** | gi\|17402583\|dbj\|BAB78506.1\| **xyloglucan endo-transglycosylase (9.37)**  gi\|7406663\|emb\|CAB85625.1\|,  gi\|157360112\|emb\|CAO69295.1\|,  gi\|157340696\|emb\|CAO47501.1\|,  gi\|157348466\|emb\|CAO23358.1\|, gi\|9885274\|gb\|AAG01382.1\|AF194174_1, gi\|22347633\|gb\|AAM95944.1\|,  gi\|147800323\|emb\|CAN68704.1\|,  gi\|157353908\|emb\|CAO46443.1\|,  gi\|147766093\|emb\|CAN65700.1\|, gi\|55977779\|sp\|P22221.2\|PPDK_FLATR | 3.93896E-4 |
|  | **GO:0006040- amino sugar metabolic process** | gi\|116329\|sp\|P29022.1\|CHIA_MAIZE, gi\|3511147\|gb\|AAC33732.1\|, gi\|2306813\|gb\|AAB65777.1\|, gi\|157353734\|emb\|CAO46266.1\|, gi\|17221690\|gb\|AAL36459.1\|AF205603_1 **nadh-cytochrome b5 reductase (1.50)** | 5.0465E-4 |
|  | **GO:0005984- disaccharide metabolic process**  **GO:0005985- sucrose metabolic process** | gi\|17402583\|dbj\|BAB78506.1\|, gi\|7406663\|emb\|CAB85625.1\|, gi\|157360112\|emb\|CAO69295.1\|, gi\|157340696\|emb\|CAO47501.1\|, gi\|157348466\|emb\|CAO23358.1\|, gi\|9885274\|gb\|AAG01382.1\|AF194174_1, gi\|22347633\|gb\|AAM95944.1\|, gi\|147800323\|emb\|CAN68704.1\|, gi\|157353908\|emb\|CAO46443.1\|, gi\|147766093\|emb\|CAN65700.1\| | 9.20361E-4 |
|  | **GO:0006073- glucan metabolic process** | gi\|17402583\|dbj\|BAB78506.1\|, gi\|7406663\|emb\|CAB85625.1\|, gi\|157360112\|emb\|CAO69295.1\|, gi\|157340696\|emb\|CAO47501.1\|, gi\|157348466\|emb\|CAO23358.1\|, gi\|9885274\|gb\|AAG01382.1\|AF194174_1, gi\|22347633\|gb\|AAM95944.1\|, gi\|147800323\|emb\|CAN68704.1\|, gi\|157353908\|emb\|CAO46443.1\|, gi\|147766093\|emb\|CAN65700.1\|, gi\|55977779\|sp\|P22221.2\|PPDK_FLATR | 0.00140388 |
| **Molecular function** | **GO:0008061- chitin binding**  **GO:0030247- polysaccharide binding**  **GO:0004568- chitinase activity**  **GO:0001871- pattern binding** | gi\|116329\|sp\|P29022.1\|CHIA_MAIZE, gi\|3511147\|gb\|AAC33732.1\|, gi\|2306813\|gb\|AAB65777.1\|, gi\|157353734\|emb\|CAO46266.1\| | 2.42649E-4 |
|  | **GO:0016829- lyase activity** | gi\|157343720\|emb\|CAO68226.1\| **ketose-bisphosphate aldolase class-ii family protein (3.88)**  gi\|20260174\|gb\|AAM12985.1\| **enolase (2-phospho-d-glycerate hydroylase) (3.21)**  gi\|146165484\|ref\|XP_001470771.1\| **2-hydroxyacyl-lyase 1 (2.72)** gi\|157349778\|emb\|CAO39634.1\| **beta-cyanoalanine synthase (2.19)**  gi\|157355881\|emb\|CAO49738.1\| **enolase (1.85)**  gi\|147766093\|emb\|CAN65700.1\|  gi\|157339869\|emb\|CAO45546.1\| **alanine aminotransferase (1.79)**  gi\|6996529\|emb\|CAB75428.1\| **enolase (1.71)** gi\|157328520\|emb\|CAO21229.1\| **glyoxalase i (1.7)** gi\|157336495\|emb\|CAO71180.1\| **glyoxalase bleomycin resistance protein dioxygenase (1.69)**  gi\|157357369\|emb\|CAO63892.1\| **pyruvate decarboxylase (1.69)**  gi\|157336882\|emb\|CAO71567.1\| **pectate lyase (1.68)**  gi\|126730565\|ref\|ZP_01746375.1\| **p-hydroxycinnamoylhydratase lyase (1.64)**  gi\|157354891\|emb\|CAO48237.1\| **root cap-specific protein (1.62)**  gi\|82941449\|dbj\|BAE48790.1\| **fructose-bisphosphate aldolase (1.58)**  gi\|157360021\|emb\|CAO68769.1\| **oxalyl-decarboxylase (1.57)**  gi\|157354769\|emb\|CAO48087.1\| **pyruvate decarboxylase (1.54)** | 3.85104E-4 |
|  | **GO:0004553- hydrolase activity, hydrolyzing O-glycosyl compounds**  **GO:0016798- hydrolase activity, acting on glycosyl bonds** | gi\|17402583\|dbj\|BAB78506.1\|, gi\|157348674\|emb\|CAO23566.1\|, gi\|116329\|sp\|P29022.1\|CHIA_MAIZE, gi\|3511147\|gb\|AAC33732.1\|, gi\|2306813\|gb\|AAB65777.1\|, gi\|157353734\|emb\|CAO46266.1\|, gi\|157335296\|emb\|CAO61126.1\|,  gi\|157348466\|emb\|CAO23358.1\|,  gi\|157330183\|emb\|CAO44690.1\| **latex cyanogenic beta glucosidase (1.53)** | 7.23969E-4  0.0015219 |
