# Supplementary material for: iTRAQ-based protein profiling provides insights into the central metabolism changes driving grape berry development and ripening
Source: BMC Plant Biol. 2013 Oct 24;13:167. doi: 10.1186/1471-2229-13-167 (PMC4016569; doi:10.1186/1471-2229-13-167)
Supplement: Additional file 8 — The GO terms enriched and sequences annotated with such terms in the up- or down-regulated subsets of proteins in each grape berry developmental stage step analyzed. [file 1471-2229-13-167-S8.zip › Additional file 8/4 mm to 7 up.doc]

| **Biological process** | **GO:0009765-photosynthesis, light harvesting**  **4mm to 7- up** | 0.0159709 | gi\|37780998\|gb\|AAP35043.1\|  **type iii chlorophyll a b-binding protein (1.54)**  gi\|147770824\|emb\|CAN76428.1\|  **chlorophyll a b-binding apoprotein cp26 precursor (1.88)**  gi\|147804704\|emb\|CAN71620.1\|  **lhcb6 protein (1.79)** |
| --- | --- | --- | --- |
