# Supplementary material for: iTRAQ-based protein profiling provides insights into the central metabolism changes driving grape berry development and ripening
Source: BMC Plant Biol. 2013 Oct 24;13:167. doi: 10.1186/1471-2229-13-167 (PMC4016569; doi:10.1186/1471-2229-13-167)
Supplement: Additional file 8 — The GO terms enriched and sequences annotated with such terms in the up- or down-regulated subsets of proteins in each grape berry developmental stage step analyzed. [file 1471-2229-13-167-S8.zip › Additional file 8/7mm to 15mm down.doc]

| **Cellular components**  **7 to 15 mm- down** | **GO:0043232- intracellular non-membrane-bound organelle**  **GO:0043228- non-membrane-bound organelle** | 1.04346E-4 | gi\|147815159\|emb\|CAN72332.1\|, gi\|147781362\|emb\|CAN67222.1\|, gi\|147802483\|emb\|CAN77412.1\|, gi\|78191412\|gb\|ABB29927.1\|, gi\|33590374\|gb\|AAQ22726.1\|, gi\|147819028\|emb\|CAN64894.1\|, gi\|156322163\|ref\|XP_001618301.1\|, gi\|125572544\|gb\|EAZ14059.1\|, gi\|147841321\|emb\|CAN60181.1\|, gi\|147835694\|emb\|CAN75201.1\|, gi\|62286683\|sp\|Q9M531.1\|H2A_EUPES, gi\|157359909\|emb\|CAO68645.1\|, gi\|61807078\|gb\|AAX55706.1\|, gi\|157357599\|emb\|CAO64572.1\|, gi\|798818\|emb\|CAA60445.1\|, gi\|157338639\|emb\|CAO41990.1\|, gi\|147801437\|emb\|CAN63603.1\|, gi\|157337755\|emb\|CAO22101.1\|, gi\|6984222\|gb\|AAF34799.1\|AF227979_1, gi\|157357316\|emb\|CAO63839.1\|, gi\|157347411\|emb\|CAO18048.1\|, gi\|18203445\|sp\|Q9SPB3.1\|RL10_VITRI, gi\|147820740\|emb\|CAN69640.1\|, gi\|157336744\|emb\|CAO71429.1\|, gi\|147789111\|emb\|CAN73497.1\|, gi\|125602544\|gb\|EAZ41869.1 gi\|125597523\|gb\|EAZ37303.1\|, gi\|125580801\|gb\|EAZ21732.1 gi\|147789820\|emb\|CAN67239.1\|, gi\|157343196\|emb\|CAO66685.1\|  gi\|157348808\|emb\|CAO23809.1\| **elongation factor 2 (0.49)**  gi\|125527658\|gb\|EAY75772.1\| **elongation factor 2 (0.53)**  gi\|74486736\|gb\|ABA12221.1\| **elongation factor 1-expressed (0.65**)  gi\|12580861\|emb\|CAC27139.1\| **ef-1 alpha (0.62)**  gi\|157358557\|emb\|CAO66233.1\| **small nuclear (0.65)** |
| --- | --- | --- | --- |
|  | **GO:0005840- ribosome** | 0.00306652 | gi\|147815159\|emb\|CAN72332.1\|, gi\|78191412\|gb\|ABB29927.1\|, gi\|33590374\|gb\|AAQ22726.1\|, gi\|125572544\|gb\|EAZ14059.1\|, gi\|147835694\|emb\|CAN75201.1\|, gi\|157359909\|emb\|CAO68645.1\|, gi\|61807078\|gb\|AAX55706.1\|, gi\|157357599\|emb\|CAO64572.1\|, gi\|798818\|emb\|CAA60445.1\|, gi\|157338639\|emb\|CAO41990.1\|, gi\|147801437\|emb\|CAN63603.1\|, gi\|157337755\|emb\|CAO22101.1\|, gi\|6984222\|gb\|AAF34799.1\|AF227979_1, gi\|157347411\|emb\|CAO18048.1\|, gi\|18203445\|sp\|Q9SPB3.1\|RL10_VITRI, gi\|147820740\|emb\|CAN69640.1\|, gi\|157336744\|emb\|CAO71429.1\|, gi\|147789111\|emb\|CAN73497.1\|, gi\|157348808\|emb\|CAO23809.1\|, gi\|125527658\|gb\|EAY75772.1\|, gi\|125602544\|gb\|EAZ41869.1\|, gi\|74486736\|gb\|ABA12221.1\|, gi\|125597523\|gb\|EAZ37303.1\|, gi\|157357316\|emb\|CAO63839.1\|, gi\|125580801\|gb\|EAZ21732.1\|, gi\|12580861\|emb\|CAC27139.1\|, gi\|147789820\|emb\|CAN67239.1\|, gi\|157343196\|emb\|CAO66685.1\| |
|  | **GO:00030529- ribonucleoprotein complex** | 8.7068E-4 | gi\|157356562\|emb\|CAO62749.1\| **hla-b associated transcript 1 (0.62)**  gi\|147815159\|emb\|CAN72332.1\|, gi\|78191412\|gb\|ABB29927.1\|, gi\|33590374\|gb\|AAQ22726.1\|, gi\|125572544\|gb\|EAZ14059.1\|, gi\|147835694\|emb\|CAN75201.1\|, gi\|157359909\|emb\|CAO68645.1\|, gi\|61807078\|gb\|AAX55706.1\|, gi\|157357599\|emb\|CAO64572.1\|, gi\|798818\|emb\|CAA60445.1\|, gi\|157338639\|emb\|CAO41990.1\|, gi\|147801437\|emb\|CAN63603.1\|, gi\|157337755\|emb\|CAO22101.1\|, gi\|6984222\|gb\|AAF34799.1\|AF227979_1, gi\|157347411\|emb\|CAO18048.1\|, gi\|18203445\|sp\|Q9SPB3.1\|RL10_VITRI, gi\|147820740\|emb\|CAN69640.1\|, gi\|157336744\|emb\|CAO71429.1\|, gi\|147789111\|emb\|CAN73497.1\|, gi\|157348808\|emb\|CAO23809.1\|, gi\|125527658\|gb\|EAY75772.1\|, gi\|125602544\|gb\|EAZ41869.1\|, gi\|74486736\|gb\|ABA12221.1\|, gi\|125597523\|gb\|EAZ37303.1\|, gi\|157357316\|emb\|CAO63839.1\|, gi\|125580801\|gb\|EAZ21732.1\|, gi\|12580861\|emb\|CAC27139.1\|, gi\|157358557\|emb\|CAO66233.1\|, gi\|147789820\|emb\|CAN67239.1\|, gi\|157343196\|emb\|CAO66685.1\| |
| **BIological process** | **GO:0006996-**  **organelle organization and biogenesis** | 4.93807E-5 | gi\|147815159\|emb\|CAN72332.1\| **40s ribosomal protein s24 (0.54)**  gi\|147781362\|emb\|CAN67222.1\| **histone h2b (0.53)**  gi\|157347894\|emb\|CAO22481.1\| **ran gtpase activating protein 1 protein (0.44)**  gi\|147802483\|emb\|CAN77412.1\| **alpha tubulin 1 (0.50)**  gi\|78191412\|gb\|ABB29927.1\| **60s ribosomal protein l35 (0.45)**  gi\|33590374\|gb\|AAQ22726.1\| **ribosomal protein s25 (0.66)**  gi\|147819028\|emb\|CAN64894.1\| **beta-6 tubulin (0.45)**  gi\|156322163\|ref\|XP_001618301.1\| **protein (0.47)**  gi\|125572544\|gb\|EAZ14059.1\| **40s ribosomal protein s23 (0.57)**  gi\|147841321\|emb\|CAN60181.1\| **histone h2a (0.47)**  gi\|147835694\|emb\|CAN75201.1\| **60s ribosomal protein l34 (0.48)** gi\|62286683\|sp\|Q9M531.1\|H2A_EUPES **histone h2a (0.44)** gi\|157359909\|emb\|CAO68645.1\| **ribosomal protein s6 (0.56)**  gi\|61807078\|gb\|AAX55706.1\| **ribosomal protein s3a (0.63)**  gi\|157357599\|emb\|CAO64572.1\| **60s ribosomal proteinbbc1 protein (0.55)**  gi\|798818\|emb\|CAA60445.1\| **60s ribosomal protein l2 (0.65)**  gi\|157338639\|emb\|CAO41990.1\| **60s ribosomal protein l27 (0.60)**  gi\|147801437\|emb\|CAN63603.1\| **60s ribosomal protein l13a (0.65)**  gi\|157337755\|emb\|CAO22101.1\| **60s ribosomal (0.53)**  gi\|147853720\|emb\|CAN80223.1\| **plasma membrane h+-atpase(0.67)**  gi\|6984222\|gb\|AAF34799.1\|AF227979_1 **40s ribosomal protein s16 (0.68)**  gi\|157347411\|emb\|CAO18048.1\| **60s ribosomal (0.54)** gi\|18203445\|sp\|Q9SPB3.1\|RL10_VITRI **60s ribosomal protein l10 (0.38)**  gi\|147820740\|emb\|CAN69640.1\| **ribosomal protein s13 (0.61)**  gi\|157336744\|emb\|CAO71429.1\| **60s ribosomal protein l14 (0.50)**  gi\|147789111\|emb\|CAN73497.1\| **60s ribosomal protein l21 (0.60)**  gi\|125602544\|gb\|EAZ41869.1\| **pectinesterase family protein (0.34)**  gi\|125597523\|gb\|EAZ37303.1\| **ribosomal proteincytosolic (0.59)**  gi\|157357316\|emb\|CAO63839.1\| **ribosomal protein s4 (0.53)**  gi\|157342079\|emb\|CAO64192.1\| **s-adenosyl-l-homocysteine hydrolase(0.47)**  gi\|147789820\|emb\|CAN67239.1\| **emp nonaspanin domain family protein (0.57)**  gi\|157343196\|emb\|CAO66685.1\| **40s ribosomal protein s12 (0.67)** |
|  | **GO:0016043-**  **cellular component organization and biogenesis** | 8.22181E-5 | gi\|147815159\|emb\|CAN72332.1\|, gi\|147781362\|emb\|CAN67222.1\|, gi\|157347894\|emb\|CAO22481.1\|, gi\|147802483\|emb\|CAN77412.1\|, gi\|78191412\|gb\|ABB29927.1\|, gi\|33590374\|gb\|AAQ22726.1\|, gi\|147819028\|emb\|CAN64894.1\|, gi\|156322163\|ref\|XP_001618301.1\|, gi\|125572544\|gb\|EAZ14059.1\|, gi\|147841321\|emb\|CAN60181.1\|, gi\|147835694\|emb\|CAN75201.1\|, gi\|62286683\|sp\|Q9M531.1\|H2A_EUPES, gi\|157359909\|emb\|CAO68645.1\|, gi\|61807078\|gb\|AAX55706.1\|, gi\|157357599\|emb\|CAO64572.1\|, gi\|798818\|emb\|CAA60445.1\|, gi\|157338639\|emb\|CAO41990.1\|, gi\|147801437\|emb\|CAN63603.1\|,  gi\|157337755\|emb\|CAO22101.1\|, gi\|147853720\|emb\|CAN80223.1\|,  gi\|6984222\|gb\|AAF34799.1\|AF227979_1,gi\|157347411\|emb\|CAO18048.1\|, gi\|18203445\|sp\|Q9SPB3.1\|RL10_VITRI, gi\|147820740\|emb\|CAN69640.1\|,  gi\|157337755\|emb\|CAO22101.1\|, gi\|147853720\|emb\|CAN80223.1\|,  gi\|6984222\|gb\|AAF34799.1\|AF227979_1,gi\|147820740\|emb\|CAN69640.1\|,  gi\|157347411\|emb\|CAO18048.1\|, gi\|18203445\|sp\|Q9SPB3.1\|RL10_VITRI, gi\|147789111\|emb\|CAN73497.1\|, gi\|125597523\|gb\|EAZ37303.1\|  gi\|157357316\|emb\|CAO63839.1\|, gi\|157342079\|emb\|CAO64192.1\|,  gi\|147789820\|emb\|CAN67239.1\|, gi\|157343196\|emb\|CAO66685.1\|,  gi\|157340835\|emb\|CAO47640.1\| **coatomer protein gamma 2-subunit (0.57)**  gi\|147861641\|emb\|CAN79299.1\| **dwf1 (diminuto 1) catalytic (0.68)**  gi\|157337082\|emb\|CAO21428.1\| **clathrin heavy chain (0.58)**  gi\|79324082\|ref\|NP_001031469.1\| **vesicle-associated membranesynaptobrevin 7b (0.63)**  gi\|157336744\|emb\|CAO71429.1\| **60s ribosomal protein l14 (0.50)**  gi\|157336145\|emb\|CAO61975.1\| **24-sterol c-methyltransferase (0.67)**  gi\|125602544\|gb\|EAZ41869.1\| **pectinesterase family protein (0.34)**  gi\|125580801\|gb\|EAZ21732.1\| **26s proteasome regulatory subunit s2 (0.13**)  gi\|157356287\|emb\|CAO62439.1\| **reversibly glycosylated polypeptide (0.62)**  gi\|157358447\|emb\|CAO66107.1\| **acla-1 (atp-citrate lyase a-1) (0.43)**  gi\|157359612\|emb\|CAO67829.1\| **membrane import protein (0.61)** |
|  | **GO:0009698-**  **phenylpropanoid metabolic process** | 1.61722E-4 | gi\|147853720\|emb\|CAN80223.1\| **plasma membrane h+-atpase (0.67)**  gi\|147801854\|emb\|CAN74850.1\| **phenylalanine ammonia-lyase (0.57)**  gi\|147775009\|emb\|CAN77208.1\| **trans-cinnamate 4-hydroxylase (0.46)**  gi\|22266677\|dbj\|BAC07545.1\| **anthocyanidin synthase (0.51)**  gi\|122893272\|gb\|ABM67589.1\| **flavanone 3-hydroxylase (0.61)**  gi\|147799448\|emb\|CAN61338.1\| **dihydroflavonol 4-reductase(0.51)**  gi\|18376653\|dbj\|BAB84111.1\| **chalcone synthase (0.65)**  gi\|18376655\|dbj\|BAB84112.1\| **chalcone synthase (0.29)** |
|  | **GO:0019438-**  **aromatic compound biosynthetic process**  **GO:0042398- amino acid derivative biosynthetic process** | 1.6455E-4  3.0607E-4 | gi\|147853720\|emb\|CAN80223.1\|, gi\|147775009\|emb\|CAN77208.1\|, gi\|22266677\|dbj\|BAC07545.1\|, gi\|122893272\|gb\|ABM67589.1\|, gi\|147799448\|emb\|CAN61338.1\|, gi\|18376653\|dbj\|BAB84111.1\|, gi\|18376655\|dbj\|BAB84112.1\|  gi\|2398679\|emb\|CAA75092.1\| **3-deoxy-d-arabino-heptulosonate 7-phosphate synthase (0.53)**  gi\|157349388\|emb\|CAO24534.1\| **5-enol-pyruvylshikimate-phosphate synthase (0.40)**  gi\|157344022\|emb\|CAO68955.1\| **chorismate synthase (0.49)**  gi\|157343467\|emb\|CAO67973.1\| **enolase (0.54)**  gi\|147837950\|emb\|CAN60561.1\| **dehydroquinate dehydratase shikimate:nadp oxidoreductase (0.35)**  gi\|157329644\|emb\|CAO41079.1\| **3-deoxy-d-arabino-heptulosonate 7-phosphate synthase (0.67)** |
|  | **GO:0009699-**  **phenylpropanoid biosynthetic process** | 6.05467E-4 | gi\|147853720\|emb\|CAN80223.1\|, gi\|147775009\|emb\|CAN77208.1\|, gi\|22266677\|dbj\|BAC07545.1\|, gi\|122893272\|gb\|ABM67589.1\|, gi\|147799448\|emb\|CAN61338.1\|, gi\|18376653\|dbj\|BAB84111.1\|, gi\|18376655\|dbj\|BAB84112.1\| |
|  | **GO:0009813-**  **flavonoid biosynthetic process**  **GO:0009812-**  **flavonoid metabolic process** | 6.72451E-4 | gi\|147853720\|emb\|CAN80223.1\|, gi\|22266677\|dbj\|BAC07545.1\|, gi\|122893272\|gb\|ABM67589.1\|, gi\|147799448\|emb\|CAN61338.1\|, gi\|18376653\|dbj\|BAB84111.1\|, gi\|18376655\|dbj\|BAB84112.1\| |
|  | **GO:0009058**  **biosynthetic process** | 3.65697E-6 | gi\|147815159\|emb\|CAN72332.1\|, gi\|78191412\|gb\|ABB29927.1\|, gi\|33590374\|gb\|AAQ22726.1\|, gi\|125572544\|gb\|EAZ14059.1\|, gi\|147835694\|emb\|CAN75201.1\|, gi\|157359909\|emb\|CAO68645.1\|, gi\|61807078\|gb\|AAX55706.1\|, gi\|157357599\|emb\|CAO64572.1\|, gi\|798818\|emb\|CAA60445.1\|, gi\|157338639\|emb\|CAO41990.1\|, gi\|157355637\|emb\|CAO49454.1\|, gi\|147801437\|emb\|CAN63603.1\|, gi\|157337755\|emb\|CAO22101.1\|, gi\|157348402\|emb\|CAO23189.1\|, gi\|147853720\|emb\|CAN80223.1\|, gi\|6984222\|gb\|AAF34799.1\|AF227979_1, gi\|157347411\|emb\|CAO18048.1\|, gi\|157342473\|emb\|CAO65185.1\|, gi\|18203445\|sp\|Q9SPB3.1\|RL10_VITRI, gi\|147820740\|emb\|CAN69640.1\|, gi\|157343766\|emb\|CAO68272.1\|, gi\|147835837\|emb\|CAN72939.1\|, gi\|157336744\|emb\|CAO71429.1\|, gi\|147789111\|emb\|CAN73497.1\|, gi\|157348808\|emb\|CAO23809.1\|, gi\|28076706\|gb\|AAO31551.1\|, gi\|157336145\|emb\|CAO61975.1\|, gi\|125527658\|gb\|EAY75772.1\|, gi\|157338441\|emb\|CAO39485.1\|, gi\|125602544\|gb\|EAZ41869.1\|, gi\|91984000\|ref\|YP_567084.1\|, gi\|74486736\|gb\|ABA12221.1\|, gi\|157349638\|emb\|CAO38776.1\|, gi\|157352913\|emb\|CAO44803.1\|, gi\|125597523\|gb\|EAZ37303.1\|, gi\|157357316\|emb\|CAO63839.1\|, gi\|157350039\|emb\|CAO39977.1\|, gi\|157344022\|emb\|CAO68955.1\|, gi\|157338864\|emb\|CAO42215.1\|, gi\|125580801\|gb\|EAZ21732.1\|, gi\|157350588\|emb\|CAO40637.1\|, gi\|157358622\|emb\|CAO66298.1\|, gi\|157358623\|emb\|CAO66299.1\|, gi\|12580861\|emb\|CAC27139.1\|, gi\|157356287\|emb\|CAO62439.1\|, gi\|157338353\|emb\|CAO39397.1\|, gi\|157329588\|emb\|CAO40867.1\|, gi\|157358447\|emb\|CAO66107.1\|, gi\|147807983\|emb\|CAN64291.1\|, gi\|147864944\|emb\|CAN83625.1\|, gi\|157343467\|emb\|CAO67973.1\|, gi\|147789820\|emb\|CAN67239.1\|, gi\|2398679\|emb\|CAA75092.1\|, gi\|157343196\|emb\|CAO66685.1\|, gi\|147801854\|emb\|CAN74850.1\|, gi\|157349388\|emb\|CAO24534.1\|, gi\|147775009\|emb\|CAN77208.1\|, gi\|157337620\|emb\|CAO21966.1\|, gi\|22266677\|dbj\|BAC07545.1\|, gi\|157345093\|emb\|CAO71821.1\|, gi\|122893272\|gb\|ABM67589.1\|, gi\|147837950\|emb\|CAN60561.1\|, gi\|147799448\|emb\|CAN61338.1\|, gi\|18376653\|dbj\|BAB84111.1\|, gi\|157329644\|emb\|CAO41079.1\|, gi\|18376655\|dbj\|BAB84112.1\| |
|  | **GO:0006725**  **aromatic compound metabolic process** | 0.00157201 | gi\|147853720\|emb\|CAN80223.1\|, gi\|157352913\|emb\|CAO44803.1\|, gi\|157344022\|emb\|CAO68955.1\|, gi\|157343467\|emb\|CAO67973.1\|, gi\|2398679\|emb\|CAA75092.1\|, gi\|147801854\|emb\|CAN74850.1\|, gi\|157349388\|emb\|CAO24534.1\|, gi\|147775009\|emb\|CAN77208.1\|, gi\|22266677\|dbj\|BAC07545.1\|, gi\|122893272\|gb\|ABM67589.1\|, gi\|147837950\|emb\|CAN60561.1\|, gi\|147799448\|emb\|CAN61338.1\|, gi\|18376653\|dbj\|BAB84111.1\|, gi\|157329644\|emb\|CAO41079.1\|, gi\|18376655\|dbj\|BAB84112.1\|  gi\|147860393\|emb\|CAN82572.1\| **methylenetetrahydrofolate reductase (0.42)**  gi\|157350039\|emb\|CAO39977.1\| **methylenetetrahydrofolate dehydrogenase--methenyltetrahydrofolate cyclohydrolase (0.68)** gi\|147843670\|emb\|CAN84153.1\| **d-isomer specific 2-hydroxyacid dehydrogenase (0.55)**  gi\|147864944\|emb\|CAN83625.1\| **nucleoside diphosphate kinase 3 (0.68)** |
|  | **GO:44238**  **primary metabolic process** | 1.06026E-4 | gi\|157344999\|emb\|CAO71727.1\| **at5g64760 mvp7_9 (0.67)**  gi\|3914449\|sp\|O64982.1\|PRS7_PRUPE **26s proteasome atpase subunit (0.62)**  gi\|147815159\|emb\|CAN72332.1\|, gi\|147781362\|emb\|CAN67222.1\|, gi\|147802483\|emb\|CAN77412.1\|, gi\|78191412\|gb\|ABB29927.1\|, gi\|33590374\|gb\|AAQ22726.1\|, gi\|147819028\|emb\|CAN64894.1\|, gi\|156322163\|ref\|XP_001618301.1\|, gi\|125572544\|gb\|EAZ14059.1\|, gi\|147841321\|emb\|CAN60181.1\|, gi\|147835694\|emb\|CAN75201.1\|, gi\|62286683\|sp\|Q9M531.1\|H2A_EUPES, gi\|157359909\|emb\|CAO68645.1\|, gi\|61807078\|gb\|AAX55706.1\|, gi\|157357599\|emb\|CAO64572.1\|, gi\|798818\|emb\|CAA60445.1\|, gi\|157338639\|emb\|CAO41990.1\|, gi\|157340835\|emb\|CAO47640.1\|, gi\|157337082\|emb\|CAO21428.1\|, gi\|157346978\|emb\|CAO17360.1\|, gi\|147769690\|emb\|CAN65529.1\|, gi\|147801437\|emb\|CAN63603.1\|, gi\|157337755\|emb\|CAO22101.1\|, gi\|157348402\|emb\|CAO23189.1\|, gi\|147853720\|emb\|CAN80223.1\|, gi\|114804265\|ref\|YP_762261.1\|, gi\|6984222\|gb\|AAF34799.1\|AF227979_1, gi\|157347411\|emb\|CAO18048.1\|, gi\|157342473\|emb\|CAO65185.1\|, gi\|18203445\|sp\|Q9SPB3.1\|RL10_VITRI, gi\|157345761\|emb\|CAO15059.1\|, gi\|157329159\|emb\|CAO24586.1\|, gi\|147820740\|emb\|CAN69640.1\|, gi\|157343766\|emb\|CAO68272.1\|, gi\|125539802\|gb\|EAY86197.1\|, gi\|147835837\|emb\|CAN72939.1\|, gi\|157336744\|emb\|CAO71429.1\|, gi\|147789111\|emb\|CAN73497.1\|, gi\|157348808\|emb\|CAO23809.1\|, gi\|28076706\|gb\|AAO31551.1\|, gi\|157336145\|emb\|CAO61975.1\|, gi\|147856673\|emb\|CAN81359.1\|, gi\|125527658\|gb\|EAY75772.1\|, gi\|157338441\|emb\|CAO39485.1\|, gi\|125602544\|gb\|EAZ41869.1\|, gi\|15236375\|ref\|NP_193129.1\|, gi\|147860393\|emb\|CAN82572.1\|, gi\|91984000\|ref\|YP_567084.1\|, gi\|74486736\|gb\|ABA12221.1\|, gi\|157349638\|emb\|CAO38776.1\|, gi\|157356562\|emb\|CAO62749.1\|, , gi\|157352913\|emb\|CAO44803.1\|, gi\|125597523\|gb\|EAZ37303.1\|, gi\|157357316\|emb\|CAO63839.1\|, gi\|157352447\|emb\|CAO43527.1\|, gi\|157342079\|emb\|CAO64192.1\|, gi\|157344022\|emb\|CAO68955.1\|, gi\|147843670\|emb\|CAN84153.1\|, gi\|157338864\|emb\|CAO42215.1\|, gi\|147820522\|emb\|CAN67659.1\|, gi\|125580801\|gb\|EAZ21732.1\|, gi\|157350588\|emb\|CAO40637.1\|, gi\|157358622\|emb\|CAO66298.1\|, gi\|157358623\|emb\|CAO66299.1\|, gi\|12580861\|emb\|CAC27139.1\|, gi\|147826481\|emb\|CAN72808.1\|, gi\|157356287\|emb\|CAO62439.1\|, gi\|157338353\|emb\|CAO39397.1\|, gi\|157329588\|emb\|CAO40867.1\|, gi\|157358447\|emb\|CAO66107.1\|, gi\|147807983\|emb\|CAN64291.1\|, gi\|147839059\|emb\|CAN67965.1\|, gi\|157350251\|emb\|CAO40189.1\|, gi\|147864944\|emb\|CAN83625.1\|, gi\|157343197\|emb\|CAO66686.1\|, gi\|157343467\|emb\|CAO67973.1\|, gi\|157340532\|emb\|CAO47337.1\|, gi\|157358557\|emb\|CAO66233.1\|, gi\|147789820\|emb\|CAN67239.1\|, gi\|2398679\|emb\|CAA75092.1\|, gi\|157343196\|emb\|CAO66685.1\|, gi\|147801854\|emb\|CAN74850.1\|, gi\|157349388\|emb\|CAO24534.1\|, gi\|147775009\|emb\|CAN77208.1\|, gi\|157337620\|emb\|CAO21966.1\|, gi\|22266677\|dbj\|BAC07545.1\|, gi\|157345093\|emb\|CAO71821.1\|, gi\|122893272\|gb\|ABM67589.1\|, gi\|147837950\|emb\|CAN60561.1\|, gi\|147799448\|emb\|CAN61338.1\|, gi\|18376653\|dbj\|BAB84111.1\|, gi\|157329644\|emb\|CAO41079.1\|, gi\|18376655\|dbj\|BAB84112.1\| |
|  | **GO:0044249**  **cellular biosynthetic process** | 3.66007E-4 | gi\|157345093\|emb\|CAO71821.1\| **myo-inositol-1-phosphate synthase (0.54)**  gi\|157337620\|emb\|CAO21966.1\| **lipoxygenase (0.29)**  gi\|147815159\|emb\|CAN72332.1\|, gi\|78191412\|gb\|ABB29927.1\|, gi\|33590374\|gb\|AAQ22726.1\|, gi\|125572544\|gb\|EAZ14059.1\|, gi\|147835694\|emb\|CAN75201.1\|, gi\|157359909\|emb\|CAO68645.1\|, gi\|61807078\|gb\|AAX55706.1\|, gi\|157357599\|emb\|CAO64572.1\|, gi\|798818\|emb\|CAA60445.1\|, gi\|157338639\|emb\|CAO41990.1\|, gi\|147801437\|emb\|CAN63603.1\|, gi\|157337755\|emb\|CAO22101.1\|, gi\|157348402\|emb\|CAO23189.1\|, gi\|147853720\|emb\|CAN80223.1\|, gi\|6984222\|gb\|AAF34799.1\|AF227979_1, gi\|157347411\|emb\|CAO18048.1\|, gi\|18203445\|sp\|Q9SPB3.1\|RL10_VITRI, gi\|147820740\|emb\|CAN69640.1\|, gi\|157343766\|emb\|CAO68272.1\|, gi\|157336744\|emb\|CAO71429.1\|, gi\|147789111\|emb\|CAN73497.1\|, gi\|157348808\|emb\|CAO23809.1\|, gi\|125527658\|gb\|EAY75772.1\|, gi\|157338441\|emb\|CAO39485.1\|, gi\|125602544\|gb\|EAZ41869.1\|, gi\|74486736\|gb\|ABA12221.1\|, gi\|157349638\|emb\|CAO38776.1\|, gi\|157352913\|emb\|CAO44803.1\|, gi\|125597523\|gb\|EAZ37303.1\|, gi\|157357316\|emb\|CAO63839.1\|, gi\|157350039\|emb\|CAO39977.1\|, gi\|157344022\|emb\|CAO68955.1\|, gi\|157338864\|emb\|CAO42215.1\|, gi\|125580801\|gb\|EAZ21732.1\|, gi\|157358622\|emb\|CAO66298.1\|, gi\|157358623\|emb\|CAO66299.1\|, gi\|12580861\|emb\|CAC27139.1\|, gi\|157338353\|emb\|CAO39397.1\|, gi\|157329588\|emb\|CAO40867.1\|, gi\|157358447\|emb\|CAO66107.1\|, gi\|147864944\|emb\|CAN83625.1\|, gi\|157343467\|emb\|CAO67973.1\|, gi\|147789820\|emb\|CAN67239.1\|, gi\|2398679\|emb\|CAA75092.1\|, gi\|157343196\|emb\|CAO66685.1\|, gi\|147801854\|emb\|CAN74850.1\|, gi\|157349388\|emb\|CAO24534.1\|, gi\|147775009\|emb\|CAN77208.1\|, gi\|22266677\|dbj\|BAC07545.1\|, gi\|122893272\|gb\|ABM67589.1\|, gi\|147837950\|emb\|CAN60561.1\|, gi\|147799448\|emb\|CAN61338.1\|, gi\|18376653\|dbj\|BAB84111.1\|, gi\|157329644\|emb\|CAO41079.1\|, gi\|18376655\|dbj\|BAB84112.1\| |
|  | **GO:0009059**  **macromolecule biosynthetic process** | 8.9765E-4 | gi\|157355637\|emb\|CAO49454.1\| **at5g23940 mro11_2 (0.45)**  gi\|157338441\|emb\|CAO39485.1\| **rhamnose biosynthetic enzymeexpressed (0.50)**  gi\|157350588\|emb\|CAO40637.1\| **cytosolic malate dehydrogenase (0.68)**  gi\|157358622\|emb\|CAO66298.1\| **pyrophosphate-dependent phosphofructokinase alpha subunit (0.51)**  gi\|157358623\|emb\|CAO66299.1\| **pyrophosphate-dependent phosphofructokinase alpha subunit (0.56)**  gi\|157329588\|emb\|CAO40867.1\| **pyrophosphate-dependent phosphofructokinase beta subunit (0.53)**  gi\|157345093\|emb\|CAO71821.1 **myo-inositol-1-phosphate synthase (0.54)**  gi\|147815159\|emb\|CAN72332.1\|,  gi\|78191412\|gb\|ABB29927.1\|, gi\|33590374\|gb\|AAQ22726.1\|, gi\|125572544\|gb\|EAZ14059.1\|, gi\|147835694\|emb\|CAN75201.1\|, gi\|157359909\|emb\|CAO68645.1\|, gi\|61807078\|gb\|AAX55706.1\|, gi\|157357599\|emb\|CAO64572.1\|, gi\|798818\|emb\|CAA60445.1\|, gi\|157338639\|emb\|CAO41990.1\|, gi\|147801437\|emb\|CAN63603.1\|, gi\|157337755\|emb\|CAO22101.1\|, gi\|6984222\|gb\|AAF34799.1\|AF227979_1, gi\|157347411\|emb\|CAO18048.1\|, gi\|18203445\|sp\|Q9SPB3.1\|RL10_VITRI, gi\|147820740\|emb\|CAN69640.1\|, gi\|147835837\|emb\|CAN72939.1\|, gi\|157336744\|emb\|CAO71429.1\|, gi\|147789111\|emb\|CAN73497.1\|, gi\|157348808\|emb\|CAO23809.1\|, gi\|28076706\|gb\|AAO31551.1\|, gi\|125527658\|gb\|EAY75772.1\|, gi\|125602544\|gb\|EAZ41869.1\|, gi\|91984000\|ref\|YP_567084.1\|, gi\|74486736\|gb\|ABA12221.1\|, gi\|157349638\|emb\|CAO38776.1\|, gi\|125597523\|gb\|EAZ37303.1\|, gi\|157357316\|emb\|CAO63839.1\|, gi\|157338864\|emb\|CAO42215.1\|, gi\|125580801\|gb\|EAZ21732.1\|, gi\|12580861\|emb\|CAC27139.1\|, gi\|157356287\|emb\|CAO62439.1\|, gi\|157338353\|emb\|CAO39397.1\|, gi\|157358447\|emb\|CAO66107.1\|, gi\|157343467\|emb\|CAO67973.1\|, gi\|147789820\|emb\|CAN67239.1\|, gi\|157343196\|emb\|CAO66685.1\|, \|, gi\|18376653\|dbj\|BAB84111.1\|, gi\|18376655\|dbj\|BAB84112.1\| |
|  | **GO:0006519**  **amino acid and derivative metabolic process** | 0.00202711 | gi\|157346978\|emb\|CAO17360.1\| **s-adenosylmethionine synthetase (0.49)**  gi\|157348402\|emb\|CAO23189.1\| **phosphoglycerate dehydrogenase (0.57)**  gi\|157343766\|emb\|CAO68272.1\| **argininosuccinate synthase (0.52)**  gi\|15236375\|ref\|NP_193129.1\| **serine hydroxymethyltransferase (0.57)**  gi\|157349638\|emb\|CAO38776.1\| **pyruvate dehydrogenase e1 beta subunit (0.53)**  gi\|157352447\|emb\|CAO43527.1\| **serine hydroxymethyltransferase (0.65)**  gi\|157350251\|emb\|CAO40189.1\| **s-adenosylmethionine synthetase (0.38)**  gi\|147853720\|emb\|CAN80223.1\|, gi\|125539802\|gb\|EAY86197.1\|, gi\|147860393\|emb\|CAN82572.1\|, gi\|157352913\|emb\|CAO44803.1\|, gi\|157342079\|emb\|CAO64192.1\|, gi\|157344022\|emb\|CAO68955.1\|, gi\|147843670\|emb\|CAN84153.1\|, gi\|157343467\|emb\|CAO67973.1\|, gi\|2398679\|emb\|CAA75092.1\|, gi\|147801854\|emb\|CAN74850.1\|, gi\|157349388\|emb\|CAO24534.1\|, gi\|147775009\|emb\|CAN77208.1\|, gi\|22266677\|dbj\|BAC07545.1\|, gi\|122893272\|gb\|ABM67589.1\|, gi\|147837950\|emb\|CAN60561.1\|, gi\|147799448\|emb\|CAN61338.1\|, gi\|18376653\|dbj\|BAB84111.1\|, gi\|157329644\|emb\|CAO41079.1\|, gi\|18376655\|dbj\|BAB84112.1\| |
|  | **GO:0009987-**  **cellular process** | 0.00325255 | gi\|147815159\|emb\|CAN72332.1\|, gi\|147781362\|emb\|CAN67222.1\|, gi\|157347894\|emb\|CAO22481.1\|, gi\|147802483\|emb\|CAN77412.1\|, gi\|78191412\|gb\|ABB29927.1\|, gi\|33590374\|gb\|AAQ22726.1\|, gi\|147819028\|emb\|CAN64894.1\|, gi\|156322163\|ref\|XP_001618301.1\|, gi\|125572544\|gb\|EAZ14059.1\|, gi\|147841321\|emb\|CAN60181.1\|, gi\|147835694\|emb\|CAN75201.1\|, gi\|62286683\|sp\|Q9M531.1\|H2A_EUPES, gi\|157359909\|emb\|CAO68645.1\|, gi\|61807078\|gb\|AAX55706.1\|, gi\|157357599\|emb\|CAO64572.1\|, gi\|798818\|emb\|CAA60445.1\|, gi\|157338639\|emb\|CAO41990.1\|, gi\|157340835\|emb\|CAO47640.1\|, gi\|147861641\|emb\|CAN79299.1\|, gi\|157337082\|emb\|CAO21428.1\|, gi\|157346978\|emb\|CAO17360.1\|, gi\|147801437\|emb\|CAN63603.1\|, gi\|157337755\|emb\|CAO22101.1\|, gi\|157348402\|emb\|CAO23189.1\|, gi\|147853720\|emb\|CAN80223.1\|, gi\|114804265\|ref\|YP_762261.1\|, gi\|6984222\|gb\|AAF34799.1\|AF227979_1, gi\|157347411\|emb\|CAO18048.1\|, gi\|157342473\|emb\|CAO65185.1\|, gi\|18203445\|sp\|Q9SPB3.1\|RL10_VITRI, gi\|157345761\|emb\|CAO15059.1\|, gi\|157329159\|emb\|CAO24586.1\|, gi\|147820740\|emb\|CAN69640.1\|, gi\|157343766\|emb\|CAO68272.1\|, gi\|157347550\|emb\|CAO18187.1\|, gi\|125539802\|gb\|EAY86197.1\|, gi\|147835837\|emb\|CAN72939.1\|, gi\|79324082\|ref\|NP_001031469.1\|, gi\|157336744\|emb\|CAO71429.1\|, gi\|147789111\|emb\|CAN73497.1\|, gi\|157348808\|emb\|CAO23809.1\|, gi\|28076706\|gb\|AAO31551.1\|, gi\|157336145\|emb\|CAO61975.1\|, gi\|147856673\|emb\|CAN81359.1\|, gi\|125527658\|gb\|EAY75772.1\|, gi\|157338441\|emb\|CAO39485.1\|, gi\|125602544\|gb\|EAZ41869.1\|, gi\|15236375\|ref\|NP_193129.1\|, gi\|147860393\|emb\|CAN82572.1\|, gi\|91984000\|ref\|YP_567084.1\|, gi\|157344999\|emb\|CAO71727.1\|, gi\|74486736\|gb\|ABA12221.1\|, gi\|157349638\|emb\|CAO38776.1\|, gi\|157356562\|emb\|CAO62749.1\|, gi\|3914449\|sp\|O64982.1\|PRS7_PRUPE, gi\|157352913\|emb\|CAO44803.1\|, gi\|125597523\|gb\|EAZ37303.1\|, gi\|157357316\|emb\|CAO63839.1\|, gi\|157350039\|emb\|CAO39977.1\|, gi\|157352447\|emb\|CAO43527.1\|, gi\|157342079\|emb\|CAO64192.1\|, gi\|157344022\|emb\|CAO68955.1\|, gi\|147843670\|emb\|CAN84153.1\|, gi\|157338864\|emb\|CAO42215.1\|, gi\|147820522\|emb\|CAN67659.1\|, gi\|125580801\|gb\|EAZ21732.1\|, gi\|157350588\|emb\|CAO40637.1\|, gi\|157358622\|emb\|CAO66298.1\|, gi\|157358623\|emb\|CAO66299.1\|, gi\|12580861\|emb\|CAC27139.1\|, gi\|147826481\|emb\|CAN72808.1\|, gi\|157356287\|emb\|CAO62439.1\|, gi\|157338353\|emb\|CAO39397.1\|, gi\|157329588\|emb\|CAO40867.1\|, gi\|157358447\|emb\|CAO66107.1\|, gi\|147807983\|emb\|CAN64291.1\|, gi\|147839059\|emb\|CAN67965.1\|, gi\|157350251\|emb\|CAO40189.1\|, gi\|147864944\|emb\|CAN83625.1\|, gi\|157343197\|emb\|CAO66686.1\|, gi\|157343467\|emb\|CAO67973.1\|, gi\|83943404\|ref\|ZP_00955863.1\|, gi\|157340532\|emb\|CAO47337.1\|, gi\|157358557\|emb\|CAO66233.1\|, gi\|147789820\|emb\|CAN67239.1\|, gi\|2398679\|emb\|CAA75092.1\|, gi\|157343196\|emb\|CAO66685.1\|, gi\|59857604\|dbj\|BAD89742.1\|, gi\|147801854\|emb\|CAN74850.1\|, gi\|157349388\|emb\|CAO24534.1\|, gi\|147775009\|emb\|CAN77208.1\|, gi\|157337620\|emb\|CAO21966.1\|, gi\|22266677\|dbj\|BAC07545.1\|, gi\|157345093\|emb\|CAO71821.1\|, gi\|122893272\|gb\|ABM67589.1\|, gi\|147837950\|emb\|CAN60561.1\|, gi\|147799448\|emb\|CAN61338.1\|, gi\|18376653\|dbj\|BAB84111.1\|, gi\|157329644\|emb\|CAO41079.1\|, gi\|18376655\|dbj\|BAB84112.1\|, gi\|157359612\|emb\|CAO67829.1\| |
|  | **GO: 0042254**  **ribosome biogenesis and assembly** | 0.0013625 | gi\|147815159\|emb\|CAN72332.1\|, gi\|78191412\|gb\|ABB29927.1\|, gi\|33590374\|gb\|AAQ22726.1\|, gi\|125572544\|gb\|EAZ14059.1\|, gi\|147835694\|emb\|CAN75201.1\|, gi\|157359909\|emb\|CAO68645.1\|, gi\|61807078\|gb\|AAX55706.1\|, gi\|157357599\|emb\|CAO64572.1\|, gi\|798818\|emb\|CAA60445.1\|, gi\|157338639\|emb\|CAO41990.1\|, gi\|147801437\|emb\|CAN63603.1\|, gi\|157337755\|emb\|CAO22101.1\|, gi\|6984222\|gb\|AAF34799.1\|AF227979_1, gi\|157347411\|emb\|CAO18048.1\|, gi\|18203445\|sp\|Q9SPB3.1\|RL10_VITRI, gi\|147820740\|emb\|CAN69640.1\|, gi\|157336744\|emb\|CAO71429.1\|, gi\|147789111\|emb\|CAN73497.1\|, gi\|125602544\|gb\|EAZ41869.1\|, gi\|125597523\|gb\|EAZ37303.1\|, gi\|157357316\|emb\|CAO63839.1\|, gi\|147789820\|emb\|CAN67239.1\|, gi\|157343196\|emb\|CAO66685.1\| |
|  | **GO:0022613**  **ribonucleoprotein complex biogenesis and assembly** | 0.003453 | gi\|147815159\|emb\|CAN72332.1\|, gi\|78191412\|gb\|ABB29927.1\|, gi\|33590374\|gb\|AAQ22726.1\|, gi\|125572544\|gb\|EAZ14059.1\|, gi\|147835694\|emb\|CAN75201.1\|, gi\|157359909\|emb\|CAO68645.1\|, gi\|61807078\|gb\|AAX55706.1\|, gi\|157357599\|emb\|CAO64572.1\|, gi\|798818\|emb\|CAA60445.1\|, gi\|157338639\|emb\|CAO41990.1\|, gi\|147801437\|emb\|CAN63603.1\|, gi\|157337755\|emb\|CAO22101.1\|, gi\|6984222\|gb\|AAF34799.1\|AF227979_1, gi\|157347411\|emb\|CAO18048.1\|, gi\|18203445\|sp\|Q9SPB3.1\|RL10_VITRI, gi\|147820740\|emb\|CAN69640.1\|, gi\|157336744\|emb\|CAO71429.1\|, gi\|147789111\|emb\|CAN73497.1\|, gi\|125602544\|gb\|EAZ41869.1\|, gi\|125597523\|gb\|EAZ37303.1\|, gi\|157357316\|emb\|CAO63839.1\|, gi\|125580801\|gb\|EAZ21732.1\|, gi\|147789820\|emb\|CAN67239.1\|, gi\|157343196\|emb\|CAO66685.1\| |
|  | **GO:0006558- L-phenylalanine metabolic process** | 0.0030516 | gi\|157344022\|emb\|CAO68955.1\|, gi\|147843670\|emb\|CAN84153.1\|, gi\|157343467\|emb\|CAO67973.1\|, gi\|2398679\|emb\|CAA75092.1\|, gi\|147801854\|emb\|CAN74850.1\|, gi\|157349388\|emb\|CAO24534.1\|, gi\|147775009\|emb\|CAN77208.1\|, gi\|147837950\|emb\|CAN60561.1\|, gi\|157329644\|emb\|CAO41079.1\| |
|  | **GO:0006575**  **amino acid derivative metabolic process** | 9.50313E-4 | gi\|147853720\|emb\|CAN80223.1\|, gi\|157344022\|emb\|CAO68955.1\|, gi\|157343467\|emb\|CAO67973.1\|, gi\|2398679\|emb\|CAA75092.1\|, gi\|147801854\|emb\|CAN74850.1\|, gi\|157349388\|emb\|CAO24534.1\|, gi\|147775009\|emb\|CAN77208.1\|, gi\|22266677\|dbj\|BAC07545.1\|, gi\|122893272\|gb\|ABM67589.1\|, gi\|147837950\|emb\|CAN60561.1\|, gi\|147799448\|emb\|CAN61338.1\|, gi\|18376653\|dbj\|BAB84111.1\|, gi\|157329644\|emb\|CAO41079.1\|, gi\|18376655\|dbj\|BAB84112.1\|  gi\|157352913\|emb\|CAO44803.1\| **aim1 protein (0.52)** |
| **Molecular function** | **GO:0005198-**  **structural molecule activity** | 1.79658E-4 | gi\|147815159\|emb\|CAN72332.1\|, gi\|147802483\|emb\|CAN77412.1\|, gi\|78191412\|gb\|ABB29927.1\|, gi\|33590374\|gb\|AAQ22726.1\|, gi\|147819028\|emb\|CAN64894.1\|, gi\|125572544\|gb\|EAZ14059.1\|, gi\|147835694\|emb\|CAN75201.1\|, gi\|157359909\|emb\|CAO68645.1\|, gi\|61807078\|gb\|AAX55706.1\|, gi\|157357599\|emb\|CAO64572.1\|, gi\|798818\|emb\|CAA60445.1\|, gi\|157338639\|emb\|CAO41990.1\|, gi\|157340835\|emb\|CAO47640.1\|, gi\|157337082\|emb\|CAO21428.1\|, gi\|147801437\|emb\|CAN63603.1\|, gi\|157337755\|emb\|CAO22101.1\|, gi\|6984222\|gb\|AAF34799.1\|AF227979_1, gi\|157347411\|emb\|CAO18048.1\|, gi\|18203445\|sp\|Q9SPB3.1\|RL10_VITRI, gi\|147820740\|emb\|CAN69640.1\|, gi\|157336744\|emb\|CAO71429.1\|, gi\|147789111\|emb\|CAN73497.1\|, gi\|125602544\|gb\|EAZ41869.1\|, gi\|125597523\|gb\|EAZ37303.1\|, gi\|157357316\|emb\|CAO63839.1\|, gi\|147789820\|emb\|CAN67239.1\|, gi\|157343196\|emb\|CAO66685.1\| |
|  | **GO:0004497-**  **monooxygenase activity** | 0.00247162 | gi\|28076706\|gb\|AAO31551.1\| **ribulose-bisphosphate carboxylase oxygenase large subunit (0.53)**  gi\|91984000\|ref\|YP_567084.1\| **ribulose-biphosphate carboxylase (0.48)** gi\|147807983\|emb\|CAN64291.1\| **ribulose bisphosphatesmall subunit (0.42)**  gi\|147775009\|emb\|CAN77208.1\| **trans-cinnamate 4-hydroxylase (0.46)** |
|  | **GO:0016829**  **lyase activity** | 0.00330388 | gi\|125539802\|gb\|EAY86197.1\| **serine decarboxylase (0.59)**  gi\|157338864\|emb\|CAO42215.1\| **alfc3_arath probable fructose-bisphosphate aldolasechloroplast precursor (0.63)**  gi\|157338353\|emb\|CAO39397.1\| **phosphoenolpyruvate carboxylase (0.66)**  gi\|28076706\|gb\|AAO31551.1\|, gi\|91984000\|ref\|YP_567084.1\|, gi\|157352913\|emb\|CAO44803.1\|, gi\|157344022\|emb\|CAO68955.1\|, gi\|157358447\|emb\|CAO66107.1\|, gi\|147807983\|emb\|CAN64291.1\|, gi\|157343467\|emb\|CAO67973.1\|, gi\|2398679\|emb\|CAA75092.1\|, gi\|147801854\|emb\|CAN74850.1\|, gi\|147837950\|emb\|CAN60561.1\|, gi\|157329644\|emb\|CAO41079.1\| |
|  | **GO:0003735- structural constituent of ribosome** | 0.0013625 | gi\|147815159\|emb\|CAN72332.1\|, gi\|78191412\|gb\|ABB29927.1\|, gi\|33590374\|gb\|AAQ22726.1\|, gi\|125572544\|gb\|EAZ14059.1\|, gi\|147835694\|emb\|CAN75201.1\|, gi\|157359909\|emb\|CAO68645.1\|, gi\|61807078\|gb\|AAX55706.1\|, gi\|157357599\|emb\|CAO64572.1\|, gi\|798818\|emb\|CAA60445.1\|, gi\|157338639\|emb\|CAO41990.1\|, gi\|147801437\|emb\|CAN63603.1\|, gi\|157337755\|emb\|CAO22101.1\|, gi\|6984222\|gb\|AAF34799.1\|AF227979_1, gi\|157347411\|emb\|CAO18048.1\|, gi\|18203445\|sp\|Q9SPB3.1\|RL10_VITRI, gi\|147820740\|emb\|CAN69640.1\|, gi\|157336744\|emb\|CAO71429.1\|, gi\|147789111\|emb\|CAN73497.1\|, gi\|125602544\|gb\|EAZ41869.1\|, gi\|125597523\|gb\|EAZ37303.1\|, gi\|157357316\|emb\|CAO63839.1\|, gi\|147789820\|emb\|CAN67239.1\|, gi\|157343196\|emb\|CAO66685.1\| |
