# Supplementary material for: iTRAQ-based protein profiling provides insights into the central metabolism changes driving grape berry development and ripening
Source: BMC Plant Biol. 2013 Oct 24;13:167. doi: 10.1186/1471-2229-13-167 (PMC4016569; doi:10.1186/1471-2229-13-167)
Supplement: Additional file 8 — The GO terms enriched and sequences annotated with such terms in the up- or down-regulated subsets of proteins in each grape berry developmental stage step analyzed. [file 1471-2229-13-167-S8.zip › Additional file 8/set to 4mm down.doc]

| **Biological process**  **Set to 4mm -down** | **GO:0009813- flavonoid biosynthetic process** | gi\|22266677\|dbj\|BAC07545.1\|- **anthocyanidin synthase (0.665)**  gi\|122893272\|gb\|ABM67589.1\|- **flavanone 3-hydroxylase (0.656)**  gi\|147799448\|emb\|CAN61338.1\|- **dihydroflavonol 4-reductase (0.604)**  gi\|18376653\|dbj\|BAB84111.1\|- **chalcone synthase (0.598)**  gi\|18376655\|dbj\|BAB84112.1\|- **chalcone synthase (0.512)** | 1.36881E-6 |
| --- | --- | --- | --- |
|  | **GO:0009812- flavonoid metabolic process** | gi\|22266677\|dbj\|BAC07545.1\| **anthocyanidin synthase (0.665)**gi\|122893272\|gb\|ABM67589.1\|  **flavanone 3-hydroxylase (0.656)** gi\|147799448\|emb\|CAN61338.1\|  **dihydroflavonol 4-reductase (0.604)**  gi\|18376653\|dbj\|BAB84111.1\|  **chalcone synthase (0.598)**  gi\|18376655\|dbj\|BAB84112.1\| **chalcone synthase (0.512)** | 1.36881E-6 |
|  | **GO:0009699- phenylpropanoid biosynthetic process** | gi\|147775009\|emb\|CAN77208.1\| - **trans-cinnamate 4-hydroxylase (0.6819**  gi\|22266677\|dbj\|BAC07545.1\|  **anthocyanidin synthase (0.665)**  gi\|122893272\|gb\|ABM67589.1\|  **flavanone 3-hydroxylase (0.656)** gi\|147799448\|emb\|CAN61338.1\|  **dihydroflavonol 4-reductase (0.604)**  gi\|18376653\|dbj\|BAB84111.1\|  **chalcone synthase (0.598)**  gi\|18376655\|dbj\|BAB84112.1\|  **chalcone synthase (0.512)** | 1.67726E-7 |
|  | **GO:0009698- phenylpropanoid metabolic process** | gi\|147801854\|emb\|CAN74850.1\|  **phenylalanine ammonia-lyase (0.686)**  gi\|147775009\|emb\|CAN77208.1\|  **trans-cinnamate 4-hydroxylase (0.681)**  gi\|22266677\|dbj\|BAC07545.1\|  **anthocyanidin synthase (0.665)** gi\|122893272\|gb\|ABM67589.1\|  **flavanone 3-hydroxylase (0.656)** gi\|147799448\|emb\|CAN61338.1\|  **dihydroflavonol 4-reductase (0.603)**  gi\|18376653\|dbj\|BAB84111.1\|  **chalcone synthase (0.598)** gi\|18376655\|dbj\|BAB84112.1\|  **chalcone synthase (0.511)** | 7.08604E-9 |
|  | **GO:0042398- amino acid derivative biosynthetic process** | gi\|157349388\|emb\|CAO24534.1\|  **5-enol-pyruvylshikimate-phosphate synthase (0.681)** gi\|147775009\|emb\|CAN77208.1\|  **trans-cinnamate 4-hydroxylase (0.681)**  gi\|22266677\|dbj\|BAC07545.1\| **anthocyanidin synthase (0.665)** gi\|122893272\|gb\|ABM67589.1\|  **flavanone 3-hydroxylase (0.656)**  gi\|147837950\|emb\|CAN60561.1\| **dehydroquinate dehydratase shikimate:nadp oxidoreductase (0.616)** gi\|147799448\|emb\|CAN61338.1\| **dihydroflavonol 4-reductase (0.603)** gi\|18376653\|dbj\|BAB84111.1\|  **chalcone synthase (0.598)**  gi\|157329644\|emb\|CAO41079.1\|  **3-deoxy-d-arabino-heptulosonate 7-phosphate synthase (0.546)**  gi\|18376655\|dbj\|BAB84112.1\| | 2.70425E-8 |
|  | **GO:0006575- amino acid derivative metabolic process** | gi\|147801854\|emb\|CAN74850.1\|  **phenylalanine ammonia-lyase (0.686)**  gi\|157349388\|emb\|CAO24534.1\|  **5-enol-pyruvylshikimate-phosphate synthase (0.681)** gi\|147775009\|emb\|CAN77208.1\|  **trans-cinnamate 4-hydroxylase (0.681**) gi\|22266677\|dbj\|BAC07545.1\|  **anthocyanidin synthase (0.665)** gi\|122893272\|gb\|ABM67589.1\|  **flavanone 3-hydroxylase (0.656)**  gi\|147837950\|emb\|CAN60561.1\|  **dehydroquinate dehydratase shikimate:nadp oxidoreductase (0.616)** gi\|147799448\|emb\|CAN61338.1\|  **dihydroflavonol 4-reductase (0.603)** gi\|18376653\|dbj\|BAB84111.1\|  **chalcone synthase (0.598)**  gi\|157329644\|emb\|CAO41079.1\|  **3-deoxy-d-arabino-heptulosonate 7-phosphate synthase (0.546)**  gi\|18376655\|dbj\|BAB84112.1\| **chalcone synthase (0.511)** | 3.08122E-8 |
|  | **GO:0019438- aromatic compound biosynthetic process** | gi\|157349388\|emb\|CAO24534.1\|  gi\|147775009\|emb\|CAN77208.1\|  gi\|22266677\|dbj\|BAC07545.1\|  gi\|122893272\|gb\|ABM67589.1\|  gi\|147837950\|emb\|CAN60561.1\| gi\|147799448\|emb\|CAN61338.1\|, gi\|18376653\|dbj\|BAB84111.1\|, gi\|157329644\|emb\|CAO41079.1\|, gi\|18376655\|dbj\|BAB84112.1\| | 1.68278E-8 |
|  | **GO: 0006725- aromatic compound metabolic process** | gi\|147801854\|emb\|CAN74850.1\|, gi\|157349388\|emb\|CAO24534.1\|, gi\|147775009\|emb\|CAN77208.1\|, gi\|22266677\|dbj\|BAC07545.1\|, gi\|122893272\|gb\|ABM67589.1\|, gi\|147837950\|emb\|CAN60561.1\|, gi\|147799448\|emb\|CAN61338.1\|, gi\|18376653\|dbj\|BAB84111.1\|, gi\|157329644\|emb\|CAO41079.1\|, gi\|18376655\|dbj\|BAB84112.1\| | 1.65288E-6 |
|  | **GO: 0006558- L-phenylalanine metabolic process** | gi\|147801854\|emb\|CAN74850.1\|, gi\|157349388\|emb\|CAO24534.1\|, gi\|147775009\|emb\|CAN77208.1\|, gi\|147837950\|emb\|CAN60561.1\|, gi\|157329644\|emb\|CAO41079.1\| | 2.24027E-4 |
|  | **GO:0019748- secondary metabolic process** | gi\|147801854\|emb\|CAN74850.1\|, gi\|147775009\|emb\|CAN77208.1\|, gi\|22266677\|dbj\|BAC07545.1\|, gi\|122893272\|gb\|ABM67589.1\|, gi\|147799448\|emb\|CAN61338.1\|, gi\|18376653\|dbj\|BAB84111.1\|, gi\|18376655\|dbj\|BAB84112.1\| | 7.48791E-6 |
|  | **GO:0006519- amino acid and derivative metabolic process** | gi\|147801854\|emb\|CAN74850.1\|, gi\|157349388\|emb\|CAO24534.1\|, gi\|147775009\|emb\|CAN77208.1\|, gi\|22266677\|dbj\|BAC07545.1\|, gi\|122893272\|gb\|ABM67589.1\|, gi\|147837950\|emb\|CAN60561.1\|, gi\|147799448\|emb\|CAN61338.1\|, gi\|18376653\|dbj\|BAB84111.1\|, gi\|157329644\|emb\|CAO41079.1\|, gi\|18376655\|dbj\|BAB84112.1\| | 1.19575E-4 |
|  | **GO:0009765- photosynthesis, light harvesting** | gi\|37780998\|gb\|AAP35043.1\|  **type iii chlorophyll a b-binding protein (0.642)** gi\|147770824\|emb\|CAN76428.1\|  **chlorophyll a b-binding apoprotein cp26 precursor (0.592)** gi\|147804704\|emb\|CAN71620.1\| **lhcb6 protein (0.590)** | 0.00108478 |
|  | **GO:0009072- aromatic amino acid family metabolic process** | gi\|147801854\|emb\|CAN74850.1\|  **phenylalanine ammonia-lyase (0.686)** gi\|157349388\|emb\|CAO24534.1\| 5-**enol-pyruvylshikimate-phosphate synthase (0.681)** gi\|147775009\|emb\|CAN77208.1\|, gi\|147837950\|emb\|CAN60561.1\|, gi\|157329644\|emb\|CAO41079.1\| | 0.00114995 |
|  | **GO:0016210- naringenin-chalcone synthase activity** | gi\|18376653\|dbj\|BAB84111.1\|  **chalcone synthase (0.598)**  gi\|18376655\|dbj\|BAB84112.1\|  **chalcone synthase (0.511)** | 0.00158664 |
|  | **GO:0016706- oxidoreductase activity, acting on paired donors, with incorporation or reduction of molecular oxygen, 2-oxoglutarate as one donor, and incorporation of one atom each of oxygen into both donors** | gi\|22266677\|dbj\|BAC07545.1\|, gi\|122893272\|gb\|ABM67589.1\| | 0.00158664 |
| **Molecular function** | **GO:0031418- L-ascorbic acid binding** | gi\|22266677\|dbj\|BAC07545.1\|, gi\|122893272\|gb\|ABM67589.1\| | 0.00158664 |
|  | **GO:0005506- iron ion binding** | gi\|147775009\|emb\|CAN77208.1\|, gi\|157337620\|emb\|CAO21966.1\|  **lipoxygenase (0.673)** gi\|22266677\|dbj\|BAC07545.1\|, gi\|122893272\|gb\|ABM67589.1\| | 0.00115822 |
|  | **GO:0016705- oxidoreductase activity, acting on paired donors, with incorporation or reduction of molecular oxygen** | gi\|147775009\|emb\|CAN77208.1\|, gi\|22266677\|dbj\|BAC07545.1\|, gi\|122893272\|gb\|ABM67589.1\| | 5.87645E-5 |
