# Supplementary material for: iTRAQ-based protein profiling provides insights into the central metabolism changes driving grape berry development and ripening
Source: BMC Plant Biol. 2013 Oct 24;13:167. doi: 10.1186/1471-2229-13-167 (PMC4016569; doi:10.1186/1471-2229-13-167)
Supplement: Additional file 8 — The GO terms enriched and sequences annotated with such terms in the up- or down-regulated subsets of proteins in each grape berry developmental stage step analyzed. [file 1471-2229-13-167-S8.zip › Additional file 8/set to 4mm- up.doc]

| **Cellular component** | **GO:0043232- intracellular non-membrane-bound organelle**  **GO:0043228- non-membrane-bound organelle** | 0.00263824 | gi\|147815159\|emb\|CAN72332.1\|, gi\|147781362\|emb\|CAN67222.1\|, gi\|147802483\|emb\|CAN77412.1\|, gi\|78191412\|gb\|ABB29927.1\|, gi\|157335152\|emb\|CAO60982.1\|, gi\|157337736\|emb\|CAO22082.1\|, gi\|157330475\|emb\|CAO46843.1\|, gi\|33590374\|gb\|AAQ22726.1\|, gi\|157345652\|emb\|CAO14950.1\|, gi\|147819028\|emb\|CAN64894.1\|, gi\|3024017\|sp\|P56331.2\|IF1A_ONOVI, gi\|156322163\|ref\|XP_001618301.1\|, gi\|125572544\|gb\|EAZ14059.1\|, gi\|147841321\|emb\|CAN60181.1\|, gi\|147815192\|emb\|CAN74433.1\|, gi\|147835694\|emb\|CAN75201.1\|, gi\|62286683\|sp\|Q9M531.1\|H2A_EUPES, gi\|157359909\|emb\|CAO68645.1\|, gi\|61807078\|gb\|AAX55706.1\|, gi\|157357599\|emb\|CAO64572.1\| |
| --- | --- | --- | --- |
|  | **GO:0005856- cytoskeleton** | 0.00254146 | gi\|147802483\|emb\|CAN77412.1\|, gi\|157335152\|emb\|CAO60982.1\|, gi\|157337736\|emb\|CAO22082.1\|, gi\|147819028\|emb\|CAN64894.1\| |
|  | **GO:0000786-nucleosome**  **GO: 0000785- chromatin** | 9.33116E-4 | gi\|147781362\|emb\|CAN67222.1\| **histone h2b (6.61)**  gi\|156322163\|ref\|XP_001618301.1\| **protein (2.50)**  gi\|62286683\|sp\|Q9M531.1\|H2A_EUPES **histone h2a (1.94)**  gi\|147841321\|emb\|CAN60181.1\|  **histone h2a (2.24)** |
|  | **GO: 0005874- microtubule** | 2.05615E-4 | gi\|147802483\|emb\|CAN77412.1\|  **alpha tubulin 1 (4.80)**  gi\|157335152\|emb\|CAO60982.1\|  **beta-tubulin (3.99)**  gi\|157337736\|emb\|CAO22082.1\|  **alpha-tubulin (3.79)**  gi\|147819028\|emb\|CAN64894.1\|  **beta-6 tubulin (3.18)** |
|  | **GO: 0044430- cytoskeletal part**  **GO: 0015630 microtubule citoskeleton** | 9.33 E-04 | gi\|147802483\|emb\|CAN77412.1\|, gi\|157335152\|emb\|CAO60982.1\|, gi\|157337736\|emb\|CAO22082.1\|, gi\|147819028\|emb\|CAN64894.1\| |
|  | **GO:0044427- chromosomal part**  **GO:0005694- chromosome** | 9.33116E-4 | gi\|147781362\|emb\|CAN67222.1\|, gi\|156322163\|ref\|XP_001618301.1\|, gi\|147841321\|emb\|CAN60181.1\|, gi\|62286683\|sp\|Q9M531.1\|H2A_EUPES |
| **Biological process** | **GO:0016043- cellular component organization and biogenesis** | 1.43136E-4 | gi\|147815159\|emb\|CAN72332.1\|  gi\|147781362\|emb\|CAN67222.1\|, gi\|157347894\|emb\|CAO22481.1\|, gi\|147802483\|emb\|CAN77412.1\|, gi\|78191412\|gb\|ABB29927.1\|  **60s ribosomal protein l35 (4.67)** gi\|157335141\|emb\|CAO60971.1\|  **heat shock protein 90 (4.40)** gi\|157339297\|emb\|CAO43838.1\|  **ran binding protein (4.30)**  gi\|157335152\|emb\|CAO60982.1\|  **beta-tubulin (3.99)** gi\|157337736\|emb\|CAO22082.1\|, gi\|157330475\|emb\|CAO46843.1\|, gi\|33590374\|gb\|AAQ22726.1\|, gi\|157345652\|emb\|CAO14950.1\|, gi\|147819028\|emb\|CAN64894.1\|, gi\|3024017\|sp\|P56331.2\|IF1A_ONOVI  **eukaryotic translation initiation factor 1a (3.08)** gi\|156322163\|ref\|XP_001618301.1\|, gi\|58333698\|emb\|CAF22243.1\|, gi\|125572544\|gb\|EAZ14059.1\|, gi\|147841321\|emb\|CAN60181.1\|, gi\|147815192\|emb\|CAN74433.1\|, gi\|147835694\|emb\|CAN75201.1\|, gi\|62286683\|sp\|Q9M531.1\|H2A_EUPES, gi\|157348710\|emb\|CAO23602.1\|, gi\|157359909\|emb\|CAO68645.1\|, gi\|61807078\|gb\|AAX55706.1\|, gi\|157340561\|emb\|CAO47366.1\|, gi\|157357599\|emb\|CAO64572.1\| |
|  | **GO:0007010- cytoskeleton organization and biogenesis** | 1.70851E-5 | gi\|157347894\|emb\|CAO22481.1\|  **ran gtpase activating protein 1 protein (5.46)**  gi\|147802483\|emb\|CAN77412.1\|  **alpha tubulin 1 (4.80)** gi\|157335152\|emb\|CAO60982.1\|  **beta-tubulin (3.99)** gi\|157337736\|emb\|CAO22082.1\|  **alpha-tubulin (3.79)** gi\|147819028\|emb\|CAN64894.1\|  **beta-6 tubulin (3.18)**  gi\|157348710\|emb\|CAO23602.1\|  **villin 2 protein (1.80)** |
|  | **GO:0006996- organelle organization and biogenesis** | 8.17446E-5 | gi\|147815159\|emb\|CAN72332.1\|  **40s ribosomal protein s24 (6.66**) gi\|147781362\|emb\|CAN67222.1\|  **histone h2b (6.61)**  gi\|157347894\|emb\|CAO22481.1\|, gi\|147802483\|emb\|CAN77412.1\|, gi\|78191412\|gb\|ABB29927.1\|, gi\|157335152\|emb\|CAO60982.1\|, gi\|157337736\|emb\|CAO22082.1\|, gi\|157330475\|emb\|CAO46843.1\|  **60s ribosomal protein l5 (3.59)** gi\|33590374\|gb\|AAQ22726.1\|  **ribosomal protein s25 (3.37)** gi\|157345652\|emb\|CAO14950.1\|  **40s ribosomal protein s9 (3.23)** gi\|147819028\|emb\|CAN64894.1\|  **beta-6 tubulin (3.18)** gi\|156322163\|ref\|XP_001618301.1\|  **protein (2.50)**  gi\|125572544\|gb\|EAZ14059.1\|  **40s ribosomal protein s23 (2.29)** gi\|147815192\|emb\|CAN74433.1\|  **histone h2a (2.24)** gi\|147835694\|emb\|CAN75201.1\|  **60s ribosomal protein l34 (1.95)**  gi\|62286683\|sp\|Q9M531.1\|H2A_EUPES  **histone h2a (1.94)** gi\|157348710\|emb\|CAO23602.1\|  **villin 2 protein (1.80)** gi\|157359909\|emb\|CAO68645.1\|  **ribosomal protein s6 (1.72)** gi\|61807078\|gb\|AAX55706.1\|  **ribosomal protein s3a (1.58)** gi\|157357599\|emb\|CAO64572.1\| 60s **ribosomal proteinbbc1 protein (1.54)** |
|  | **GO:0006334- nucleosome assembly**  **GO:0065004- protein-DNA complex assembly** | 9.33116E-4 | gi\|147781362\|emb\|CAN67222.1\|  gi\|156322163\|ref\|XP_001618301.1\|  gi\|147841321\|emb\|CAN60181.1\|  gi\|62286683\|sp\|Q9M531.1\|H2A_EUPES |
|  | **GO:0007018- microtubule based- movement**  **GO: 0030705-cytoskeleton-dependent intracellular transport**  **GO: 0051258- protein polymerization** | 2.05615E-4 | gi\|147802483\|emb\|CAN77412.1\|, gi\|157335152\|emb\|CAO60982.1\|, gi\|157337736\|emb\|CAO22082.1\|, gi\|147819028\|emb\|CAN64894.1\| |
|  | **GO:0007017- microtubule-based process** | 2.37403E-5 | gi\|157347894\|emb\|CAO22481.1\|, gi\|147802483\|emb\|CAN77412.1\|, gi\|157335152\|emb\|CAO60982.1\|, gi\|157337736\|emb\|CAO22082.1\|, gi\|147819028\|emb\|CAN64894.1\| |
|  | **GO:0051649- establishment of cellular localization**  **GO:0051641- cellular localization** | 0.00256991 | gi\|157347894\|emb\|CAO22481.1\|, gi\|147802483\|emb\|CAN77412.1\|, gi\|157335141\|emb\|CAO60971.1\|, gi\|157339297\|emb\|CAO43838.1\|, gi\|157335152\|emb\|CAO60982.1\|, gi\|157337736\|emb\|CAO22082.1\|, gi\|157330475\|emb\|CAO46843.1\|, gi\|147819028\|emb\|CAN64894.1\| |
| **Molecular function** | **GO:0016798- hydrolase activity, acting on glycosyl bonds** | 1.28943E-4 | gi\|157351694\|emb\|CAO42683.1\|  **vacuolar invertase (2.69)** gi\|157328892\|emb\|CAO23301.1\|  **atagal2 (arabidopsis thaliana alpha-galactosidase 2) alpha-galactosidase (2.30)**  gi\|157343385\|emb\|CAO67436.1\|  **glycosyl hydrolase family 3 n terminal domain containingexpressed (2.13)**  gi\|157359301\|emb\|CAO67237.1\|  **glycosylhydrolase 1 (1.80)** gi\|157335911\|emb\|CAO61741.1\|  **glycosyl hydrolase family 3 protein (1.61)** |
|  | **GO:0004553- hydrolase activity, hydrolyzing O-glycosyl compounds** | 2.05615E-4 | gi\|157351694\|emb\|CAO42683.1\|, gi\|157328892\|emb\|CAO23301.1\|, gi\|157343385\|emb\|CAO67436.1\|, gi\|157335911\|emb\|CAO61741.1\| |
